# Genome-Wide Host-Pathogen Interaction Unveiled by Transcriptomic Response of Diamondback Moth to Fungal Infection

**DOI:** 10.1371/journal.pone.0152908

**Published:** 2016-04-04

**Authors:** Zhen-Jian Chu, Yu-Jun Wang, Sheng-Hua Ying, Xiao-Wei Wang, Ming-Guang Feng

**Affiliations:** 1 Institute of Microbiology, College of Life Sciences, Zhejiang University, Hangzhou 310058, China; 2 Institute of Insect Science, College of Agriculture and Biotechnology, Zhejiang University, Hangzhou 310058, China; Louisiana State University & LSU AgCenter, UNITED STATES

## Abstract

Genome-wide insight into insect pest response to the infection of *Beauveria bassiana* (fungal insect pathogen) is critical for genetic improvement of fungal insecticides but has been poorly explored. We constructed three pairs of transcriptomes of *Plutella xylostella* larvae at 24, 36 and 48 hours post treatment of infection (hpt_I_) and of control (hpt_C_) for insight into the host-pathogen interaction at genomic level. There were 2143, 3200 and 2967 host genes differentially expressed at 24, 36 and 48 hpt_I_/hpt_C_ respectively. These infection-responsive genes (~15% of the host genome) were enriched in various immune processes, such as complement and coagulation cascades, protein digestion and absorption, and drug metabolism-cytochrome P450. Fungal penetration into cuticle and host defense reaction began at 24 hpt_I_, followed by most intensive host immune response at 36 hpt_I_ and attenuated immunity at 48 hpt_I_. Contrastingly, 44% of fungal genes were differentially expressed in the infection course and enriched in several biological processes, such as antioxidant activity, peroxidase activity and proteolysis. There were 1636 fungal genes co-expressed during 24–48 hpt_I_, including 116 encoding putative secretion proteins. Our results provide novel insights into the insect-pathogen interaction and help to probe molecular mechanisms involved in the fungal infection to the global pest.

## Introduction

The diamond-back moth *Plutella xylostella* (Lepidoptera: Yponomeutidae) coevolves with crucifer [1] and hence is one of most devastating insect pests threatening economically important crops, such as cabbage, cauliflower and rapeseed [2]. The pest damage results in an annual economic loss of 4–5 billion US dollars worldwide [3,4]. This insect pest is also notorious with its high resistance to almost all classes of chemical insecticides [5,6] and crystal toxins of *Bacillus thuringiensis* [7]. Thus, it is critical to develop alternative strategies for the pest control [4].

One of alternative strategies against the pest is to make use of filamentous fungal insect pathogens, such as *Beauveria bassiana* and *Metarhizium anisopliae* which have been widely used as fungal insecticides [8–10]. Such fungi start infection by conidial adhesion to the insect integument, followed by germination and hyphal penetration through the host cuticle [11,12] by means of the action of various cuticle-degrading enzymes [13–17]. Upon entry into the host hemocoel, penetrating multicellular hyphae turn into unicellular blastospores, which must overcome high osmolarity and defensive immunity-derived oxidation encountered in the host hemolymph for yeast-like budding propagation until the host dies from mycosis [18–20]. Subsequently, the blastospores turn back into hyphae to penetrate the integument again for outgrowth and produce conidia on the cadaver surface for the initiation of a new infection cycle. This infection mode endows the insect pathogens with an ability to kill not only piercing insects but also chewing pests, such as *P*. *xylostella* [21]. The fungal lethal action is a process of host-pathogen interaction involved in cuticular penetration, blastospore propagation and host death due to the depletion of hemolymph nutrition. This process is namely a latent period of several days varying with insect species and body size, making the fungal lethal action slower than the action of a chemical insecticide. There has been no recorded insect resistance to fungal insect pathogens, perhaps a consequence of the slower lethal action.

The major disadvantage of fungal insecticides has been alleviated by rapid progress in entomopathogenic fungal biotechnology. Fungal virulence has been enhanced in transgenic fungal strains expressing exogenous chitinase, hybrid chitinase or protease (Pr1A) in *B*. *bassiana* [22–24]. The integration of a scorpion neurotoxin into fungal candidate strains has resulted in a great increase of toxicity to several lepidoptera pests [25–27] and malaria parasites within mosquitoes [28]. In spite of the normal route of cuticular penetration, fungal strains can acquire *per os* virulence for expansion of target pest spectrum by the integration of an insect midgut-specific toxin, such as the vegetative insecticidal protein Vip3Aa1 from *B*. *thuringiensis* [29–31]. A fungal insecticide based on a Vip3Aa1-expressing strain can compete with a chemical insecticide to protect a cabbage crop from full-season damages caused by an insect pest complex comprising *P*. *xylostella*, caterpillars and aphids [32]. Overexpression of an endogenous Mn^2+^-cofactored superoxide dismutase (MnSOD) in a *B*. *bassiana* strain resulted in not only enhanced virulence but increased tolerance to oxidative stress and UV-B irradiation [33]. Since the transgenic strains improved by exogenous gene expression are subject to strict safety evaluation prior to registration, it is ideal to explore more endogenous genes, such as the MnSOD gene, for use in genetic improvement of fungal insecticides. However, this is impeded by a lack of deep insight into the host-pathogen interaction due to unavailability for genomic information of fungal insect pathogens and many important insect pests a few years ago.

The annotated genomes of *B*. *bassiana* [34] and *P*. *xylostella* [35] provide an excellent opportunity for a genome-wide insight into the host-pathogen interaction by means of next generation sequencing (NGS) technology [36,37]. This study seeks to study the transcriptomic response of *P*. *xylostella* to the *B*. *bassiana* infection by analyzing digital gene expression (DGE) libraries (transcriptomes) of total RNAs derived respectively from the *P*. *xylostella* larvae at 24, 36 and 48 hours post treatment of infection (hpt_I_) and of control (hpt_C_). We identified a large number of differentially expressed genes (DEGs) in the pest response to the infection of *B*. *bassiana* and many fungal DEGs involved in the course of host infection.

## Materials and Methods

### Insect stock

The *P*. *xylostella* strain Fuzhou-S, which was used in the *P*. *xylostella* genome sequencing project [35], was maintained on caged cabbage plants at 25 ± 1°C in a light/dark cycle of 14:10 h under a fluctuating relative humidity of 65–85%. The third-instar larvae from the plants were prepared for the following use.

### Treatment of larvae with conidial suspension

The wild-type strain *B*. *bassiana* ARSEF 2860, which attacks many piercing and chewing insects and hence has been subjected to genome sequencing and annotation [34], was cultivated for full conidiation on Sabouraud dextrose agar (4% glucose, 1% peptone and 1.5% agar) plus 1% yeast extract at 25°C in a light/dark cycle of 12:12 h. Conidia harvested from the culture were suspended in 0.02% Tween 80 and standardized to 1 × 10^8^ conidia/ml. Three cohorts (replicates) of ~35 larvae on cabbage leaf discs (~10 cm in diameter) were separately exposed to an equal-volume (1 ml) spray of conidial suspension (treatment) or 0.02% Tween 80 (control) from the top nozzle of an Automatic Potters Spray Tower (Burkard Scientific Ltd, Uxbridge, UK) at a uniform working pressure of 0.7 kg/cm^2^. The sprayed larvae were reared *in situ* in large Petri dishes at 25°C under a photoperiod of 14:10 h and monitored daily for mortality records, which were corrected based on the background mortality in the control. Fresh leaf discs were supplied for their feeding whenever necessary during the period of rearing.

### Samples of infected and uninfected larvae for RNA extraction

The mortality curve of the larvae after spray was used to prepare insect samples for RNA extraction. Several cohorts of larvae were sprayed with the conidial suspension (treatment) or 0.02% Tween 80 (control) and reared as above. Samples of 50 surviving larvae were collected from the fungal treatment and the control at 24, 36 and 48 h respectively, forming three pairs of hpt_I_ and hpt_C_ samples.

### Construction and analysis of DGE libraries

Each sample of 50 larvae was immediately ground in liquid nitrogen. Total RNA was extracted from each ground sample with an RNAiso^TM^ Plus Reagent (TaKaRa, Dalian, China), purified with a Qiagen RNeasy Mini Kit (Qiagen, Germantown, MD, USA) plus on-column treatment with DNase I, and reversely transcribed into cDNA under the action of a PrimeScript RT^®^ Reagent kit (TaKaRa). The resultant cDNA was separated into large and small parts. The large part was sequenced at Beijing Genomics Institute (Shenzhen, China) for constructing a DGE library by means of an Illumina HiSeq™ 2000 platform (San Diego, CA, USA). The small part was used as a template to verify the sequencing by assessing transcript levels of selected genes through quantitative real-time PCR (qRT-PCR) experiments.

All raw DGE reads gained by sequencing the cDNA samples were filtered to generate clean tags, which were mapped with SOAP2 program [38] to the respective genome databases of *P*. *xylostella* [35] and *B*. *bassiana* [34] at the level of no more than five-base mismatching. All the data were normalized as reads per kilobase transcriptome per million mapped reads (RPKM). The whole DGE library database was registered in Gene Expression Omnibus (GEO) and available under the accession code GSE72383.

All *P*. *xylostella* DEGs were identified by horizontal comparison of paired DGE libraries (i.e., 24, 36 and 48 hpt_I_ versus 24, 36 and 48 hpt_C_ respectively) or longitudinal comparison between each pair of the libraries at two sequential time points (i.e., 36 versus 24 hpt_I_ or 48 versus 36 hpt_I_) based on the standards of *P* ≤ 0.001, FDR ≤ 0.001 and |log_2_ ratio| ≥ 1. All identified host DEGs were functionally annotated with known or putative gene information in the non-redundant NCBI protein databases and subjected to Gene Ontology (GO) analysis (http://www.geneontology.org/) and Kyoto Encyclopedia of Genes and Genomes (KEGG) analysis (http://www.genome.jp/kegg/).

Similarly, all *B*. *bassiana* DEGs were identified through the GO term and KEGG enrichment analyses of the fungal DGE libraries at 36/24 hpt_I_ and 48/36 hpt_I_ respectively.

### Validation of DGE libraries

Seven DEGs were arbitrarily taken from each of the host DGE libraries at the three time points. Their transcript levels in the 10-fold dilution samples of the same cDNAs used for sequencing were quantified in a Mastercycler^®^ Ep-Realplex (Eppendorf, Hamburg, Germany) cycler via qRT-PCR with SRBR^®^ Premix Ex Taq^TM^ (TaKaRa) and paired primers (Table A in S1 File). A gene (Px008022.1) encoding 60S ribosomal protein L32 was used as an internal reference. The mean log_2_ R of each gene in each cDNA from the infected larvae over that in the cDNA from the control was calculated and compared with the value in the corresponding pair of DGE libraries to judge a validity of the constructed libraries. Three samples of each cDNA were used as replicates in the qRT-PCR experiment.

## Results and Discussion

### Survival trend of *P*. *xylostella* larvae heavily infected by *B*. *bassiana*

**Fig 1:**
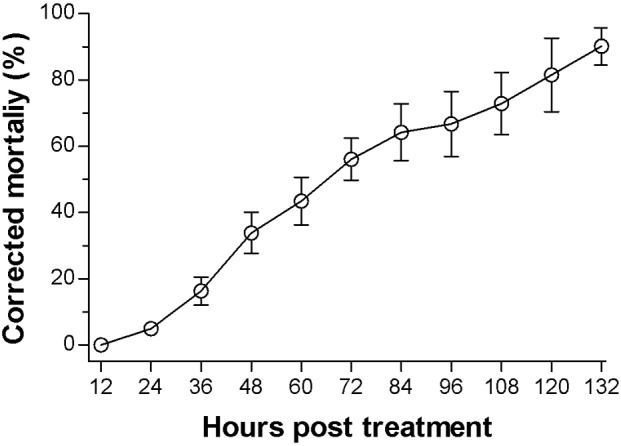
Corrected mortality trend of third-instar *P*. *xylostella* larvae infected by *B*. *bassiana*. Error bars: SD of the mean from three replicates.

The *B*. *bassiana* strain was assayed for its virulence to the third-instar larvae of *P*. *xylostella* by spraying the highly concentrated suspension (1×10^8^ conidia/ml) to cohorts of ~35 larvae in the spray tower. This spray was expected to maximize both the fungal infection to the larvae and the fungal mass accumulation in the infected larvae, which were then used for total RNA extraction. As illustrated in Fig 1, the spray resulted in a corrected mortality of ~5%, 33% and 56% at 24, 48 and 72 hpt_I_, respectively, and reached 90% at 132 hpt_I_. The mortality trend of the sprayed larvae suggests a critical period of 24–48 hpt_I_ for the host-pathogen interaction.

### Features of DGE libraries

Total RNAs were extracted from three pairs of 50 *P*. *xylostella* larvae surviving at 24, 36 and 48 hpt_I_ and hpt_C_ respectively and sequenced by means of the NGS technique, resulting in a total number of 65 million raw tags (Table 1). Percent distribution for the copy number of tags in each sequenced sample (Figure A in S1 File) coincides well with the reported feature of mRNA distribution [39–41]. All clean tags from each sequenced sample were mapped to the genomes of *P*. *xylostella* [35] and *B*. *bassiana* [34]. As a result, paired DGE libraries derived from the infected and uninfected (control) larvae comprised 14166 and 14382 host genes at 24 hpt_I_ and hpt_C_, 14773 and 13995 at 36 hpt_I_ and hpt_C_, and 14837 and 13903 at 48 hpt_I_ and hpt_C_, respectively. Up to 3148, 3613 and 4922 fungal genes were also expressed in the infected larvae at 24, 36 and 48 hpt_I_ respectively, constituting three other DGE libraries specific to the fungal infection.

**Table 1:** Distribution of reads and genes in the DGE libraries. *

Reads mapped to reference genome	24 h		36 h		48 h	
	Count	%	Count	%	Count	%
**Fungal treatment**						
Total reads	65228988	100.0	64624232	100.0	66880206	100.0
Mapped reads	32824512	50.3	34905517	54.0	35485924	53.1
Unique match	29665023	45.5	31800308	49.2	31712757	47.4
Detected host genes	14166	78.4	14773	81.8	14837	82.1
**Control**						
Total reads	65035304	100.0	65464942	100.0	66644942	100.0
Mapped reads	35048892	53.9	35183307	53.7	31841983	47.8
Unique match	31639270	48.7	31695748	48.4	28419774	42.6
Detected host genes	14382	79.6	13995	77.4	13903	76.9

* The percentage (%) for the mapped reads or the unique match is based on the count of total reads while the percentage for the detected host genes is based on the number of *P*. *xylostella* genes (18071).

**Fig 2:**
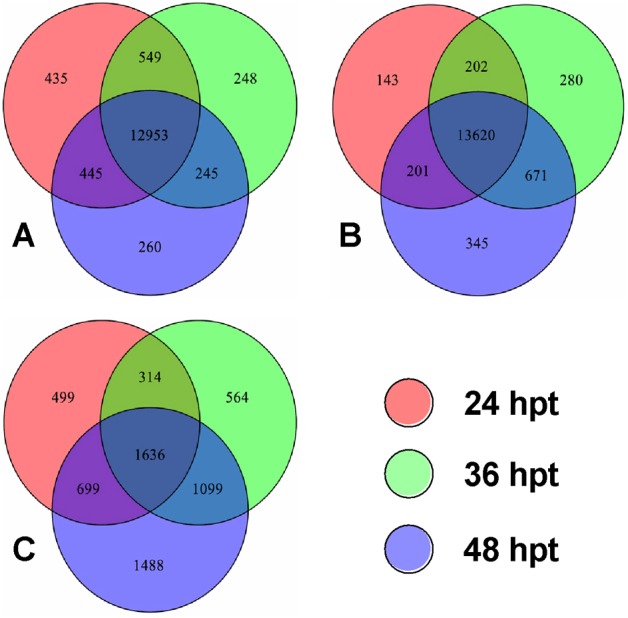
Venn diagrams for gene expression in *P*. *xylostella* and *B*. *bassiana*. (**A, B**) Counts of *P*. *xylostella* genes expressed respectively in the control (uninfected) and infected larvae at 24, 36 and 48 h post treatment (hpt). (**C**) Counts of *B*. *bassiana* genes expressed in the infected *P*. *xylostella* larvae at the three time points.

The counts of host genes consistently detected in the DGE libraries at 24, 36 and 48 h were up to 12953 and 13620 in the respective control (Fig 2A) and fungal treatment (Fig 2B). At the three time points, 996 host genes were expressed only in the infected larvae while other 328 genes were expressed only in the uninfected larvae. There were 1950 fungal genes co-expressed in the infected larvae at 24 and 36 hpt_I_, 2735 at 36 and 48 hpt_I_, and 1636 at the three time points (Fig 2C), indicating potential roles of these genes in the fungal response to the host immune defense during the critical period of infection.

### Host genes identified from the DGE libraries

**Fig 3:**
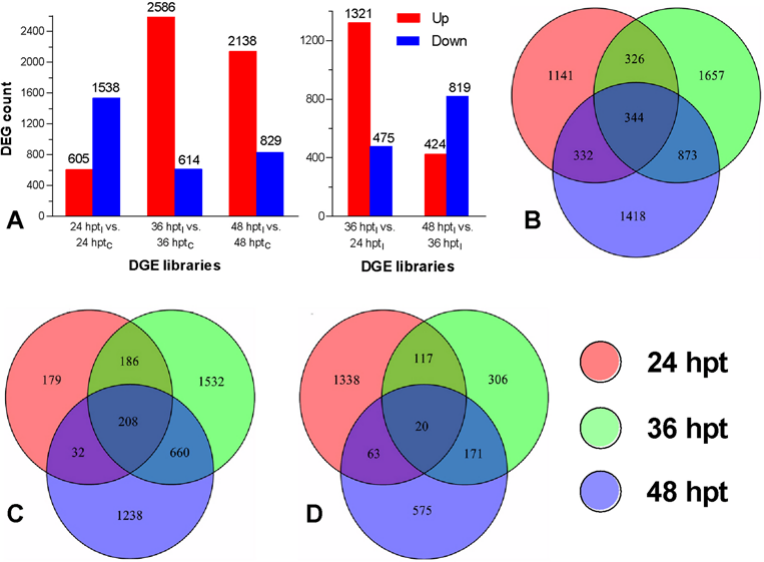
Identification of *P*. *xylostella* DEGs at 24, 36 and 48 h post treatment of infection (hpt_I_) and control (hpt_C_). (**A**) Counts of DEGs in the DGE libraries of 24 hpt_I_/24 hpt_C_, 36 hpt_I_/36 hpt_C_ and 48 hpt_I_/48 hpt_C_ (left) or of 36/24 hpt_I_ and 48/36 hpt_I_ (right). (**B–D**) Venn diagrams for the total counts of host genes differentially expressed and those upregulated and downregulated at 24, 36 and 48 hpt_I_ respectively. Note the counts of those co-upregulated (C) or co-downregulated (D) at the two time points of 36/24 and 48/36 hpt_I_ and at the three time points.

To identify host genes responsive to the fungal infection, the DGE libraries from the infected larvae were horizontally compared with the control counterparts based on the log_2_ ratios of host genes (|log_2_ R| ≥ 1) in each pair of DGE libraries and also longitudinally compared at 36/24 hpt_I_ and 48/36 hpt_I_. As illustrated in Fig 3A (left), 605, 2586 and 2138 host genes were upregulated at 24, 36, and 48 hpt_I_/hpt_C_, respectively, while 1538, 614 and 829 host genes were downregulated at the three time points. In the longitudinal comparison, 1321 host genes were specifically upregulated at 36/24 hpt_I_, contrasting to 424 host genes upregulated at 48/36 hpt_I_ (Fig 3A, right). The horizontal comparisons revealed that 344 host DEGs were consistently present at the three time points (Fig 3B), including 208 co-upregulated (Fig 3C) and 20 co-downregulated genes (Fig 3D). The host genes co-up- and co-downregulated at 24 and 36 hpt_I_ reached 394 and 137, and the counts increased to 868 and 191 at 36 and 48 hpt_I_, respectively. These data implicate that each stage of infection has its specific requirements and interferes with specific biological processes in the host. Previously, The first cuticle penetration of *B*. *bassiana* was observed to occur in intersegmental membrane of *Solenopsis invicta* at 18 h after inoculation, and the penetration expanded on to the insect cuticle around setae at 24 h after inoculation and on to thorax, abdomen and legs soon after that [42]. Unicellular blastospores (hyphal bodies) were present in the hemolymph of *Spodoptera exigua* at 36 h after *B*. *bassiana* infection [43]. Therefore, we speculate that at 24 hpt_I_ in this study, the fungal conidia adhered to the host integument could have germinated to initiate cuticular penetration, which could induce the host reaction with the early upregulated genes, followed by many more host genes activated in the defense response to the fungal penetration through the cuticle for entry into the hemocoel at 36 and 48 hpt_I_.

To gain insight into the quality of the constructed DGE libraries, we assessed transcript levels of three sets of seven host DEGs taken at each time point via qRT-PCR and compared these transcripts with the corresponding transcripts in the DGE libraries following previous studies [41,44,45]. As a result, the up- or downregulated trend of each set of gene transcripts assessed by qRT-PCR was well in agreement with the corresponding expression trend of the selected genes in the libraries. This comparison confirmed the validity of the DGE libraries constructed in the present study.

### Host DEGs enriched in KEGG pathways

**Fig 4:**
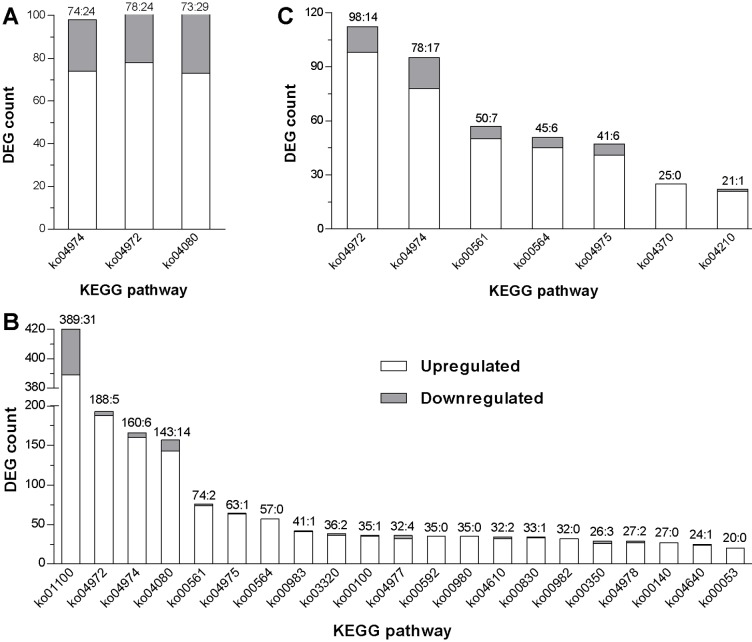
KEGG pathways enriched with *P*. *xylostella* DEGs. (**A–C**) Distribution of host DEGs in enriched KEGG pathways at 24, 36 and 48 h post treatment (hpt) of *B*. *bassiana* infection versus control respectively. All identified DEGS and enriched KEGG pathways are detailed in Tables B–D in S1 File.

All the DEGs horizontally compared at the three time points were mapped to KEGG pathways at the significant levels of *P* < 0.01 and *Q* < 0.01 [41,46]. The host DEGs at 24 hpt_I_ were significantly enriched only in three KEGG pathways (Fig 4A; detailed in Table B in S1 File), including protein digestion and absorption, pancreatic secretion and neuroactive ligand-receptor interaction. Interestingly, 74% of those host genes in the pathways were upregulated by the fungal infection, which apparently triggered the early response of *P*. *xylostella* at 24 hpt_I_.

The host DEGs at 36 hpt_I_ were enriched in the aforementioned and 18 other KEGG pathways, including immunity- and detoxification-related pathways (such as complement and coagulation cascades, drug metabolism-other enzymes, metabolism of xenobiotics by cytochrome P450) and metabolism-related pathways (such as glycerolipid metabolism and alpha-linolenic acid metabolism) (Fig 4B; detailed in Table C in S1 File). Previously, such pathways have been shown to play important roles in the response of *Ostrinia furnacalis* and *Bombyx mori* to microbial challenge [47,48]. Intriguingly, up to 91% of the enriched host genes were upregulated at 36 hpt_I_, indicating their importance for *P*. *xylostella* to cope with the fungal infection at 36 hpt_I_.

Only were seven KEGG pathways were enriched by the host DEGs at 48 hpt_I_ (Fig 4C; detailed in Table D in S1 File). Several immunity- or detoxification-related pathways activated at 36 hpt_I_ disappeared at 48 hpt_I_, including drug metabolism-other enzymes, complement and coagulation, metabolism of xenobiotics by cytochrome P450, and drug metabolism-cytochrome P450. The number and proportion of upregulated genes also decreased drastically at 48 hpt_I_ versus 36 hpt_I_. For instance, those upregulated genes in the pathways of protein digestion/absorption and pancreatic secretion decreased to 78 and 98 at 48 hpt_I_ from 160 and 188 at 48 hpt_I_, respectively. The host apoptosis pathway was significantly activated only at 48 hpt_I_.

**Fig 5:**
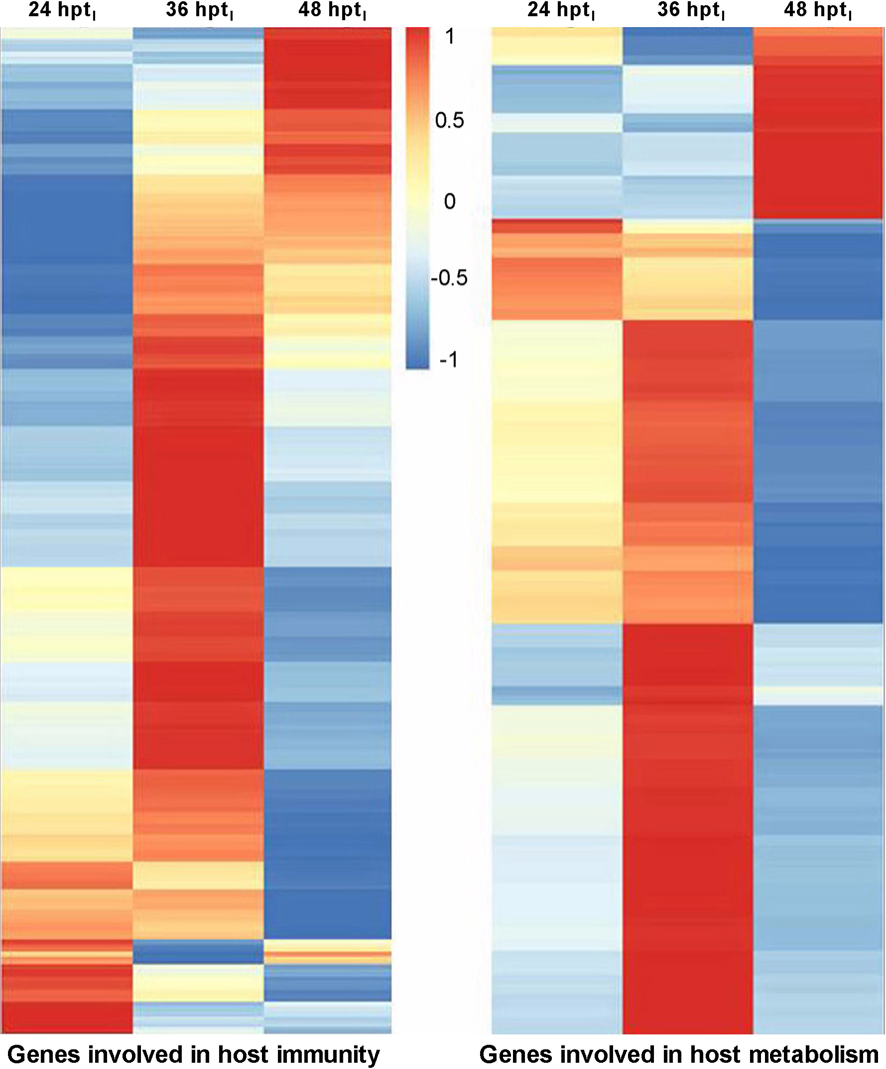
Heatmap for the distribution of *P*. *xylostella* immunity- and metabolism-related genes expressed at 24, 36 and 48 h after the *B*. *bassiana* infection. Scale bar: Gene expression level in the fungal treatment versus control.

In the paired DGE libraries at 36/24 hpt_I_, many DEGs were enriched in eight immunity- and metabolism-related KEGG pathways (*P* < 0.05), such as complement and coagulation cascade, Jak-STAT signaling, alpha-linolenic acid metabolism and glycerophospholipid metabolism (Table E in S1 File). Almost all of these DEGs were upregulated, and many more were present at 36 hpt_I_ than at 24 hpt_I_. In contrast, an analysis of the paired libraries at 48/36 hpt_I_ resulted in 22 immunity- and metabolism-related pathways (Table F in S1 File). During this 12 h period, most of the detected host genes were downregulated in all the immunity-related pathways, such as protein digestion and absorption, drug metabolism-other enzymes, complement and coagulation cascades, and lysosome. This implicates that the host immune system could have been largely impaired at 48 hpt_I_, coinciding well with the subsequent increase of host death (Fig 1). Other enriched pathways were all associated with metabolism, and most genes in the pathways were also downregulated. Up to 505 genes involved in the immune pathways and 211 in the metabolism pathways are illustrated as a heatmap (Fig 5) to show expressional changes at 24, 36 and 48 hpt_I_ respectively. Additionally, up to 996 host genes were co-expressed only in the infected larvae at 24, 36 and 48 hpt_I_ and enriched in the immunity-related complement and coagulation cascades (*P* < 0.05) whereas only 328 host genes were co-expressed in the uninfected larvae at the three time points but not enriched in any immunity-related pathway (Table G in S1 File).

Taken together, the immune defense response of *P*. *xylostella* to the infection of *B*. *bassiana* increased drastically during a short period of cuticular penetration from 24 to 36 hpt_I_ and was largely overcome by the fungal pathogen that could have entered the host hemocoel at 48 hpt_I_. In other words, the short period from 24 to 36 hpt_I_ was critical for a success of the fungal infection to the insect pest.

### Host genes crucial for immune defense response to fungal infection

In the pathway of protein digestion and absorption, many more host genes were upregulated than downregulated at all the concerned time points (Fig 4). The number of the genes upregulated at log_2_ R ≥ 2 in this pathway (see Tables B–D in S1 File) reached 20 at 24 hpt_I_ (log_2_ R_max_ = 10.9), increased to 117 at 36 hpt_I_ (log_2_ R_max_ = 10.4) and then decreased to only 22 at 48 hpt_I_ (log_2_ R_max_ = 3.1). Among those upregulated, trypsin genes took a large proportion, followed by the genes encoding serine proteinases and carboxypeptidases. Apparently, these genes play critical roles in the host defense reaction to the fungal infection. Previously, serine proteinases in *P*. *xylostella* were considered to circumvent insecticidal plant protease inhibitors through differential expression in response to different plant hosts [35]. The pest defense system could make use of the serine proteinases against either the inhibitors or the fungal infection, which may trigger many more genes for the host defense response.

The complement and coagulation systems are proteolytic cascades composed of the serine proteases in the chymotrypsin family and play important roles in host-pathogen interactions. Complement and coagulation are activated at the respective sites of infection and bleeding in vertebrates [49]. We found 32 upregulated genes and two downregulated genes significantly enriched in the pathway of complement and coagulation cascades at 36 hpt_I_ (Fig 4B; detailed in Table C in S1 File). Intriguingly, most of the upregulated host genes encode serine proteinases, coagulation factors and hemocytin. In particular, three genes encoding transmembrane serine proteases were upregulated by >1000-fold at the time point (log_2_ R: 10.3–13.5). Previously, *Drosophila* complement and clotting systems were shown to be similar in structure and function, and serine proteases could be the ancestor of both [50,51]. The coagulation factors and hemocytin are known to play vital roles in vertebrate and insect immunity [49,52]. Several upregulated genes encoding coagulation factors and hemocytin at 36 hpt_I_ implicate that they may involve in the host immune defense against the fungal entry into the host hemocoel, warranting future studies.

Moreover, up to 42 detoxification-related genes have been found in the *P*. *xylostella* genome, including glutathione S-transferases (GST), cytochrome P450 monooxygenases (P450) and carboxylesterases (COE) [35]. Of those, two were upregulated and six were downregulated at 24 hpt_I_. The upregulated genes increased to 30 at 36 hpt_I_ but dropped to 10 at 48 hpt_I_ (Table H in S1 File). These highlight a significance of the GST, P450 and COE genes for the pest defense response during the fungal infection. Several other genes encoding UDP glucosyltransferases (UGT), sulfatase-modifying factors (SUMF) and glucosinolate sulphatases (GSS) are also on the list of the detoxification-related genes [35], and some of them were also upregulated at 36 or 48 hpt_I_, suggesting them to be involved in the pest defense response to the fungal infection

The GO annotation at *P* < 0.05 resulted in a classification of 45 DEGs to four GO terms (intracellular ligand-gated calcium channel activity, ryanodine-sensitive calcium-release channel activity, calcium ion transmembrane transporter activity and calcium channel activity) at 24 hpt_I_ and of 1346 DEGs to six GO terms (peptidase activity, hydrolase activity, catalytic activity, serine-type peptidase activity, serine hydrolase activity and peptidase activity) at 36 hpt_I_ (Table 2; detailed in Tables I and J in S1 File). Most of involved genes in the GO terms at 24 hpt_I_ were downregulated, hinting that the host calcium channels could have been impaired by the early fungal attack for cuticular penetration. All the GO terms at 36 hpt_I_ were linked to the activities of various enzymes, of which most genes were upregulated. These imply that the upregulated genes could play vital roles for the insect immunity defense against *B*. *bassiana* at 36 hpt_I_, which was a critical time point for the fungus-insect interaction. However, no meaningful GO terms were enriched for the DEGs at 48 hpt_I_ perhaps due to the immune defense system collapsed by the fungal attack at that time.

**Table 2:** GO terms for *P*. *xylostella* DEGs significantly enriched to 'Molecular Function' categories.

GO ID	GO Term	DEGs	No. genes	*P* value
**24 hpt**_**I**_**/hpt**_**C**_				
GO:0005218	intracellular ligand-gated calcium channel activity	6	7	0.0186
GO:0005219	ryanodine-sensitive calcium-release channel activity	6	7	0.0186
GO:0015085	calcium ion transmembrane transporter activity	18	50	0.0298
GO:0005262	calcium channel activity	15	38	0.0354
**36 hpt**_**I**_**/hpt**_**C**_				
GO:0008233	peptidase activity	101	305	<0.0001
GO:0016787	hydrolase activity	356	1541	<0.0001
GO:0003824	catalytic activity	755	3774	<0.0001
GO:0008236	serine-type peptidase activity	33	66	<0.0001
GO:0017171	serine hydrolase activity	33	66	<0.0001
GO:0070011	peptidase activity, acting on L-amino acid peptides	68	225	0.0010

### Crucial genes involved in the *B*. *bassiana* infection to *P*. *xylostella*

Up- and downregulated *B*. *bassiana* genes (|log_2_ R| ≥ 1) reached 1934 and 1871 in the DGE libraries at 36/24 hpt_I_, and 3497 and 1071 at 48/36 hpt_I_, respectively. Up to 556 fungal genes were co-upregulated at the three time points, contrasting to only 149 co-suppressed genes. The fungal genes were enriched in 9, 11 and 10 KEGG pathways at 24, 36 and 48 hpt_I_ (*P* < 0.01; Table 3) respectively. Of those, five were co-enriched at the three time points, including energy metabolism and substance biosynthesis (oxidative phosphorylation and TCA cycle), while two or three were specifically enriched at each time point, such as pyruvate metabolism and glyoxylate and dicarboxylate metabolism only at 24 hpt_I_, aminoacyl-tRNA biosynthesis, protein export and RNA polymerase only at 36 hpt_I_, and ribosome biogenesis and 2-oxocarboxylic acid metabolism only at 48 hpt_I_.

**Table 3:** KEGG enrichment analysis of *B*. *bassiana* genes.

Pathway ID	Description	*P* value		
		24 hpt_I_	36 hpt_I_	48 hpt_I_
ko03010	Ribosome	1.29E-18	7.50E-20	1.98E-15
ko00190	Oxidative phosphorylation	6.33E-08	1.71E-06	3.99E-07
ko03050	Proteasome	3.33E-05	2.78E-04	1.45E-03
ko01200	Carbon metabolism	8.23E-05		6.13E-05
ko00020	Citrate cycle (TCA cycle)	9.58E-04	3.76E-03	1.10E-04
ko04141	Protein processing in endoplasmic reticulum	1.56E-03	2.81E-05	5.06E-03
ko00620	Pyruvate metabolism	2.74E-03		
ko00010	Glycolysis / Gluconeogenesis	3.07E-03	8.02E-04	
ko00630	Glyoxylate and dicarboxylate metabolism	5.74E-03		
ko00970	Aminoacyl-tRNA biosynthesis		5.12E-03	
ko03008	Ribosome biogenesis in eukaryotes			7.44E-05
ko01230	Biosynthesis of amino acids		4.15E-03	1.13E-06
ko03013	RNA transport		5.35E-04	8.00E-04
ko03060	Protein export		6.59E-03	
ko03020	RNA polymerase		8.48E-03	
ko01210	2-Oxocarboxylic acid metabolism			3.07E-03

The detected fungal genes were enriched in 91, 116 and 101 GO terms at 24, 36 and 48 hpt_I_ (*P* < 0.01; Tables K–M in S1 File), respectively. Two terms specifically enriched at 24 hpt_I_ were antioxidant activity and peroxidase activity, which are relevant to 10 antioxidant genes and seven peroxidase genes and indicate an importance of antioxidation for the fungal infection to the pest at the early stage of cuticular penetration. The host insect is assumed to generate superoxide anions harmful to the fungus via its immune defense response to the fungal infection. The expression of antioxidant and peroxidase genes could help the fungus to overcome the harmful effect [33,53–55]. Additionally, 24 fungal genes were enriched in proteolysis at 36 hpt_I_, a time point approaching to the completion of cuticular penetration. Apparently, these genes could be likely involved in the degradation of host cuticle and defense proteins.

Secreted proteins are considered to be crucial effectors likely involved in fungal invasion and virulence [56–58]. In this study, our DGE libraries contained up to 246 fungal genes encoding putative secretion proteins that were co-expressed at two or three time points. Among those, 116 were co-expressed at the three time points (Table N in S1 File) and hence could be likely involved in the cuticular penetration, intrahemocoel proliferation and lethal action of *B*. *bassiana*.

### Conclusive remarks

Thousands (44%) of *B*. *bassiana* genes were differentially expressed at 24–48 hpt_I_ for successful infection to *P*. *xylostella*. In contrast, the host genes were differentially expressed at a much lower proportion (~15% only) in response to the fungal infection despite a variation over the infection time. The unbalanced low proportion of the host genes expressed in the immune defense against the fungal attack obviously determines the fate of the host-pathogen interaction, i.e., collapse of the host defense systems by *B*. *bassiana* and subsequent increase of the host death. This fact highlights a genome-wide background for *B*. *bassiana* to attack not only *P*. *xylostella* but many more insects and mites [8] and hence for its great potential against global arthropod pests. Our transcriptomic analyses provide the first insight into a genome-wide interaction between *B*. *bassiana* and *P*. *xylostella* and many clues to further explore molecular mechanisms involved in the host-pathogen interactions.

## Supporting Information

S1 File **Figure A and Tables A**–N.
(PDF)
