# Supplementary material for: Genome-Wide Host-Pathogen Interaction Unveiled by Transcriptomic Response of Diamondback Moth to Fungal Infection
Source: PLoS One. 2016 Apr 4;11(4):e0152908. doi: 10.1371/journal.pone.0152908 (PMC4820269; doi:10.1371/journal.pone.0152908)
Supplement: S1 File — Figure A and Tables A–N. (PDF) [file pone.0152908.s001.pdf]

PLoS ONE

Supporting Information

Genome-Wide Host-Pathogen Interaction Unveiled by Transcriptomic Response of Diamondback Moth to Fungal Infection

Zhen-Jian Chu[1], Yu-Jun Wang[2], Sheng-Hua Ying[1], Xiao-Wei Wang[2*] and Ming-Guang Feng[1*]

[1] Institute of Microbiology, College of Life Sciences; [2] Institute of Insect Science, College of Agriculture and Biotechnology, Zhejiang University, Hangzhou 310058, China

*Corresponding to: M.-G. Feng (mgfeng@zju.edu.cn) or X.-W. Wamg (xwwang@zju.edu.cn)

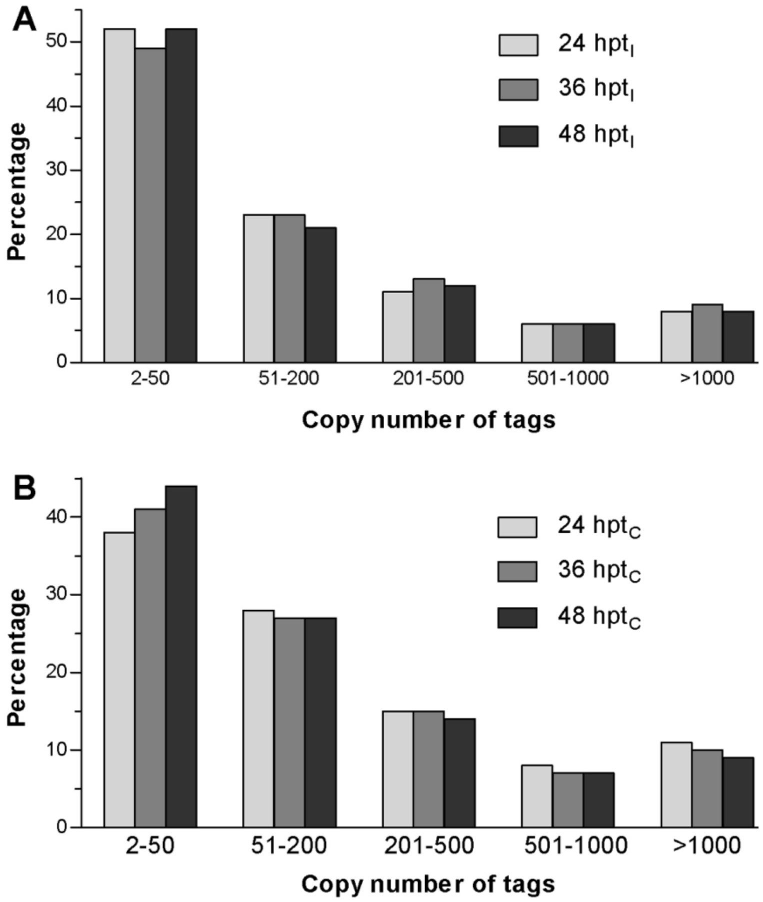

Figure A. Distribution of copy number of tags in the transcriptomoes of third-instar larvae of *P. xylostella* at 24, 36 and 48 h post treatment of *B. bassiana* infection (A; hpt$_I$) and control (B; hpt$_C$).

Table A. Comparison of $\log_2$ R values for the genes randomly selected from the DGE libraries of *P. xylostella* and detected via qRT-PCR.

Gene ID	qPCR $\log_2$ R	DGE $\log_2$ R	Annotation	
**24 hpt_I/24 hpt_C**				
Px009419.1	3.70 ± 0.23	4.12	Seminal fluid protein HACP044	AGACTCACCGCCAGAATC / AATTGGTTATTGACAGTGTTTCCT
Px000119.2	3.23 ± 0.17	3.97	Inositol hexakisphosphate and diphosphoinositol-pentakisphosphate kinase	TGTTATACCGCAGGAATACG / TTCTTCAGCAGTGGAGTG
Px010113.1	4.80 ± 0.45	3.95	Moricin-like peptide C4	GAAGGGCGGAAGAGTTAT / CGTGGTTGTACACTTCAT
Px001312.1	4.76 ± 0.31	3.76	Peptidoglycan-recognition protein LB	CAACACATCGGCATCCAC / GAATCTCCAATCTCCAATAAGACA
Px012904.1	-3.02 ± 0.43	-1.92	Serine/threonine-protein phosphatase 2A catalytic subunit alpha isoform	TATGCCTCAGATATAGAC / ACCGATAAGGAATAAGAT
Px009109.1	-4.41 ± 0.86	-1.90	Cuticular protein CPG4	TTACAGTCACTACTCCTCTC / GTTCACCACCTTGTTCAC
Px010053.1	-1.32 ± 0.09	-1.88	Eukaryotic translation initiation factor 5	TGATGAGGAGGATGAAGAGGAT / TTGGACGGCTTGGTGTATG
**36 hpt_I/36 hpt_C**				
Px006831.1	2.49 ± 0.36	4.83	Putative uncharacterized protein	CACTGGAGATGGAATTAG / CGTGTTTGTCTTCTATGA
Px008276.1	0.41 ± 0.06	4.61	Membrane alanyl aminopeptidase	AATACTCAGATTGCCTTC / GGAGGAGATGTAGGATAA
Px000995.2	2.48 ± 0.55	4.39	Carboxypeptidase A4	CTATGGCAATGGAACACT / CTTCTTAACAGCATCAATGG
Px004749.1	2.40 ± 0.24	4.25	Lipoprotein lipase	CTTTACACCAGGAGGAAC / AAGTTGGAGTTGTTGATG
Px001065.1	-5.18 ± 0.97	-3.91	Cuticle protein	TACTCCTCTGGCTACTCT / TCAGTAGTATCCACCGTG
Px008653.1	-3.97 ± 0.28	-3.33	Cuticle protein	AGTATTCGCTCTCCTGGT / TCCGTATGAGAAGCTGTTG
Px014536.1	-2.59 ± 0.38	-2.91	Unknown function	ATACGCTGCCTCCTACAT / AGTATGAGTACGAGTACGAAGT
**48 hpt_I/48 hpt_C**				
Px008089.1	5.82 ± 1.05	3.92	Pancreatic lipase-related protein 2	TATGGTATCAATGGTGACGAAT / ACCGAAGCCAACTATGTG
Px010113.1	2.66 ± 0.66	3.38	Moricin-like peptide C4	GAAGGGCGGAAGAGTTAT / CGTGGTTGTACACTTCAT
Px013480.1	2.83 ± 0.21	3.04	Hemolin	AACTAACGATAACTCACAAC / GCTTCACATCCATAATACC
Px001312.1	4.73 ± 0.63	3.02	Peptidoglycan-recognition protein LB	CAACACATCGGCATCCAC / GAATCTCCAATCTCCAATAAGACA
Px017384.1	-2.99 ± 0.35	-3.62	Putative cuticle protein	GGTCGGACGCACAGTAAG / TTGCTCAATACCTTATGGTAGACT
Px011723.1	-0.06 ± 0.03	-3.34	Unknown function	GGTAATGTCAATCAGGATA / TTCACTCAGTTCTTCTTC
Px010443.2	-0.8 ± 0.26	-3.20	Putative uncharacterized protein	AGTGGATTCAATGCTGATG / CCGTAGGAACCAAGTGTA
Px008022. 1			60S ribosomal protein L32	CAATCAGGCCAATTTACCGC / CTGGGTTTACGCCAGTTACG

Table B. A list of DEGs enriched in the pathways of *P. xylostella* 24 h post treatment of infection (hpt_I) versus 24 h post treatment of control (hpt_C).

Gene ID	log_2 R	P-value	FDR	Annotation
**Involved in Protein digestion and absorption**				
Px015280.1	10.8687	2.87E-10	1.76E-09	Trypsin CFT-1; similar with TRYP_CHOFU
Px007357.2	6.6517	0.00E+00	0.00E+00	Serine proteinase stubble; similar with STUB_DROME
Px006254.1	4.4212	6.36E-06	2.52E-05	Chymotrypsin-2; similar with CTR2_ANOGA
Px007620.1	3.6327	1.75E-08	9.13E-08	Trypsin CFT-1; similar with TRYP_CHOFU
Px016055.1	3.2151	6.89E-29	1.03E-27	Trypsin, alkaline C; similar with TRYC_MANSE
Px003546.1	3.1733	2.80E-04	8.56E-04	Trypsin, alkaline C; similar with TRYC_MANSE
Px010386.1	2.6772	0.00E+00	0.00E+00	Trypsin CFT-1; similar with TRYP_CHOFU
Px015277.1	2.5730	1.28E-54	3.19E-53	Trypsin CFT-1; similar with TRYP_CHOFU
Px015276.1	2.5579	1.86E-30	2.90E-29	Trypsin CFT-1; similar with TRYP_CHOFU
Px007619.2	2.3494	0.00E+00	0.00E+00	Trypsin, alkaline A; similar with TRYA_MANSE
Px014769.1	2.3190	3.12E-54	7.70E-53	Serine proteinase stubble; similar with STUB_DROME
Px005241.1	2.3004	9.73E-13	7.17E-12	Trypsin CFT-1; similar with TRYP_CHOFU
Px009569.1	2.2896	2.53E-216	1.66E-214	Trypsin CFT-1; similar with TRYP_CHOFU
Px010975.1	2.2277	1.85E-08	9.63E-08	Trypsin, alkaline C; similar with TRYC_MANSE
Px014256.1	2.1988	9.73E-09	5.21E-08	Unknown function
Px011887.1	2.1323	9.63E-147	4.78E-145	Trypsin, alkaline C; similar with TRYC_MANSE
Px009488.1	2.0714	3.01E-06	1.24E-05	Chymotrypsin-like serine proteinase
Px009088.1	2.0671	8.40E-275	6.89E-273	Mastin; similar with TRYM_CANFA
Px007621.1	2.0663	1.43E-07	6.93E-07	Trypsin CFT-1; similar with TRYP_CHOFU
Px003670.2	2.0614	0.00E+00	0.00E+00	Serine protease easter; similar with EAST_DROME
Px002441.1	1.9871	4.10E-39	7.84E-38	Trypsin, alkaline B; similar with TRYB_MANSE
Px006571.1	1.9668	0.00E+00	0.00E+00	Trypsin, alkaline B; similar with TRYB_MANSE
Px008538.1	1.9379	5.30E-72	1.61E-70	Trypsin, alkaline B; similar with TRYB_MANSE
Px011231.1	1.8761	1.79E-17	1.77E-16	Protein unc-50 homolog; similar with UNC50_DROME
Px015278.1	1.8699	3.08E-11	2.04E-10	Trypsin, alkaline A; similar with TRYA_MANSE
Px014830.1	1.8574	0.00E+00	0.00E+00	Tryptase; similar with TRYB1_MOUSE
Px009568.1	1.8431	6.70E-05	2.27E-04	Trypsin, alkaline C; similar with TRYC_MANSE
Px001990.2	1.7922	1.40E-22	1.73E-21	Carboxypeptidase B; similar with CBPB_ASTFL
Px011499.2	1.7910	6.11E-19	6.55E-18	Serine protease persephone; similar with PSH_DROME
Px001833.1	1.7864	0.00E+00	0.00E+00	Serine protease easter; similar with EAST_DROME
Px011737.1	1.7654	0.00E+00	0.00E+00	Neurofilament heavy polypeptide; similar with NFH_MOUSE
Px017515.1	1.7186	1.09E-80	3.60E-79	Trypsin, alkaline C; similar with TRYC_MANSE
Px007598.1	1.6692	9.57E-31	1.50E-29	Chymotrypsin-1; similar with CTR1_ANOGA
Px005240.1	1.6409	7.98E-17	7.63E-16	Trypsin, alkaline A; similar with TRYA_MANSE
Px005242.1	1.6327	5.68E-10	3.38E-09	Trypsin CFT-1; similar with TRYP_CHOFU
Px000107.1	1.5952	7.26E-250	5.34E-248	Trypsin, alkaline C; similar with TRYC_MANSE
Px014882.1	1.5792	1.59E-27	2.28E-26	Trypsin, alkaline C; similar with TRYC_MANSE
Px015275.1	1.5751	0.00E+00	0.00E+00	Trypsin CFT-1; similar with TRYP_CHOFU
Px007616.1	1.5713	2.75E-83	9.34E-82	Trypsin CFT-1; similar with TRYP_CHOFU
Px006572.1	1.5612	0.00E+00	0.00E+00	Trypsin; similar with TRYP_PHACE
Px013377.1	1.5480	1.88E-43	3.82E-42	Trypsin; similar with TRYP_PHACE
Px016057.1	1.5292	2.72E-23	3.45E-22	Trypsin, alkaline B; similar with TRYB_MANSE
Px012048.1	1.5055	2.98E-12	2.12E-11	Chymotrypsin BI; similar with CTRB1_LITVA
Px005936.2	1.5008	1.12E-40	2.19E-39	Trypsin, alkaline C; similar with TRYC_MANSE
Px000996.2	1.4897	7.66E-31	1.21E-29	Carboxypeptidase A2; similar with CBPA2_MOUSE
Px005243.1	1.4820	0.00E+00	0.00E+00	Trypsin, alkaline A; similar with TRYA_MANSE
Px012568.1	1.4572	4.65E-123	2.06E-121	Trypsin CFT-1; similar with TRYP_CHOFU
Px005765.1	1.4516	5.48E-18	5.59E-17	Vitellin-degrading protease; similar with VDP_BOMMO
Px001649.1	1.4424	1.08E-21	1.30E-20	MAM domain-containing glycosylphosphatidylinositol anchor protein 1

Px002440.1	1.4335	9.19E-09	4.93E-08	Trypsin, alkaline B; similar with TRYB_MANSE
Px014534.1	1.4237	7.49E-32	1.21E-30	Trypsin CFT-1; similar with TRYP_CHOFU
Px016058.1	1.3964	2.20E-207	1.39E-205	Trypsin, alkaline C; similar with TRYC_MANSE
Px003671.1	1.3824	8.09E-185	4.69E-183	Serine protease easter; similar with EAST_DROME
Px009638.1	1.3782	0.00E+00	0.00E+00	Trypsin, alkaline B; similar with TRYB_MANSE
Px014831.2	1.3474	0.00E+00	0.00E+00	Serine proteinase stubble; similar with STUB_DROME
Px000991.6	1.3435	4.35E-64	1.21E-62	Carboxypeptidase O; similar with CBPO_BOVIN
Px016054.1	1.3362	5.17E-08	2.60E-07	Trypsin; similar with TRYP_PHACE
Px015049.1	1.3171	5.14E-10	3.08E-09	Venom dipeptidyl peptidase 4; similar with VDDP4_VESVU
Px011428.1	1.2988	8.65E-06	3.36E-05	Peptide transporter family 1; similar with PEPT1_DROME
Px010627.1	1.2976	5.21E-112	2.15E-110	Wing disc-specific protein; similar with Q8WPH2_BOMMO
Px007900.1	1.2849	0.00E+00	0.00E+00	Chymotrypsin BI; similar with CTRB1_LITVA
Px009639.1	1.2774	0.00E+00	0.00E+00	Trypsin CFT-1; similar with TRYP_CHOFU
Px005328.1	1.2421	2.94E-22	3.60E-21	Tubulin-specific chaperone cofactor E-like protein
Px002588.1	1.2360	9.87E-07	4.33E-06	Trypsin, alkaline C; similar with TRYC_MANSE
Px009395.1	1.1916	1.32E-05	5.00E-05	Proclotting enzyme; similar with PCE_TACTR
Px011886.1	1.1422	3.65E-31	5.79E-30	Trypsin CFT-1; similar with TRYP_CHOFU
Px016056.1	1.1375	0.00E+00	0.00E+00	Trypsin, alkaline A; similar with TRYA_MANSE
Px013299.1	1.1172	3.69E-82	1.25E-80	Serine proteinase stubble; similar with STUB_DROME
Px000402.2	1.1008	8.17E-19	8.68E-18	Serine protease persephone; similar with PSH_DROME
Px012329.1	1.0969	2.64E-32	4.31E-31	Serine protease persephone; similar with PSH_DROME
Px007599.1	1.0927	0.00E+00	0.00E+00	Chymotrypsin-1; similar with CTR1_ANOGA
Px000993.1	1.0428	2.30E-209	1.47E-207	Carboxypeptidase A2; similar with CBPA2_RAT
Px007600.1	1.0192	1.33E-52	3.21E-51	Chymotrypsin-1; similar with CTR1_ANOGA
Px012191.1	1.0108	5.91E-126	2.65E-124	Sodium/potassium-transporting ATPase subunit alpha-1
Px013146.1	-9.6079	4.14E-05	1.46E-04	Trypsin; similar with TRYP_PHACE
Px012497.1	-9.3373	1.47E-04	4.73E-04	Trypsin-4; similar with TRY4_ANOGA
Px010996.1	-9.2004	6.17E-06	2.46E-05	Excitatory amino acid transporter; similar with EAA1_CAEEL
Px017492.1	-8.9602	3.86E-08	1.96E-07	Trypsin CFT-1; similar with TRYP_CHOFU
Px000188.1	-8.4578	1.16E-05	4.44E-05	Inactive dipeptidyl peptidase 10; similar with DPP10_HUMAN
Px000082.7	-3.4897	4.66E-08	2.35E-07	Collagen alpha-1(XVIII) chain; similar with COIA1_MOUSE
Px008406.1	-2.8856	5.90E-05	2.02E-04	Serine proteinase stubble; similar with STUB_DROME
Px013802.1	-2.8624	2.15E-07	1.03E-06	Sodium/potassium-transporting ATPase subunit alpha-2
Px009218.1	-2.2069	4.12E-21	4.84E-20	Protein BCL9 homolog; similar with BCL9_DROME
Px013807.1	-2.1804	2.38E-06	1.00E-05	Proton-coupled amino acid transporter 4; similar with S36A4_XENLA
Px011954.1	-2.1036	5.94E-08	2.97E-07	Sodium/hydrogen exchanger 3; similar with SL9A3_HUMAN
Px011754.1	-2.0240	6.17E-28	9.02E-27	Proton-coupled amino acid transporter 1; similar with S36A1_HUMAN
Px013747.1	-1.9944	1.35E-32	2.23E-31	Proton-coupled amino acid transporter 1; similar with S36A1_HUMAN
Px011097.1	-1.8357	1.53E-15	1.35E-14	Serine proteinase stubble; similar with STUB_DROME
Px005208.1	-1.6792	2.19E-06	9.23E-06	Inactive dipeptidyl peptidase 10; similar with DPP10_HUMAN
Px015790.1	-1.6407	8.72E-18	8.82E-17	Sodium/potassium-transporting ATPase subunit beta-2
Px005341.1	-1.4819	1.93E-101	7.46E-100	Collagenase; similar with COGS_HYPLI
Px012129.1	-1.2904	2.16E-19	2.36E-18	Lysosomal Pro-X carboxypeptidase; similar with PCP_PONAB
Px008897.1	-1.2522	5.06E-13	3.79E-12	B(0,+)-type amino acid transporter 1; similar with BAT1_HUMAN
Px004622.1	-1.2131	1.22E-41	2.45E-40	Proton-coupled amino acid transporter 4; similar with S36A4_XENLA
Px011827.1	-1.2043	3.63E-15	3.15E-14	Proton-coupled amino acid transporter 4; similar with S36A4_XENLA
Px008960.1	-1.1092	1.75E-41	3.49E-40	Peptide transporter family 1; similar with PEPT1_DROME
Px003113.1	-1.0084	1.25E-04	4.04E-04	Collagen alpha-1(XV) chain; similar with COFA1_HUMAN
Px001239.1	-1.0036	9.68E-09	5.19E-08	Serine protease P162; similar with D2A5Y8_TRICA

Involved in influenza A

Px015280.1	10.8687	2.87E-10	1.76E-09	Trypsin CFT-1; similar with TRYP_CHOFU
Px007357.2	6.6517	0.00E+00	0.00E+00	Serine proteinase stubble; similar with STUB_DROME
Px006254.1	4.4212	6.36E-06	2.52E-05	Chymotrypsin-2; similar with CTR2_ANOGA

Px007620.1	3.6327	1.75E-08	9.13E-08	Trypsin CFT-1; similar with TRYP_CHOFU
Px016055.1	3.2151	6.89E-29	1.03E-27	Trypsin, alkaline C; similar with TRYC_MANSE
Px003546.1	3.1733	2.80E-04	8.56E-04	Trypsin, alkaline C; similar with TRYC_MANSE
Px010386.1	2.6772	0.00E+00	0.00E+00	Trypsin CFT-1; similar with TRYP_CHOFU
Px015277.1	2.5730	1.28E-54	3.19E-53	Trypsin CFT-1; similar with TRYP_CHOFU
Px015276.1	2.5579	1.86E-30	2.90E-29	Trypsin CFT-1; similar with TRYP_CHOFU
Px015514.1	2.3827	1.35E-06	5.80E-06	Serine/threonine-protein kinase pelle; similar with KPEL_DROME
Px007619.2	2.3494	0.00E+00	0.00E+00	Trypsin, alkaline A; similar with TRYA_MANSE
Px014769.1	2.3190	3.12E-54	7.70E-53	Serine proteinase stubble; similar with STUB_DROME
Px005241.1	2.3004	9.73E-13	7.17E-12	Trypsin CFT-1; similar with TRYP_CHOFU
Px009569.1	2.2896	2.53E-216	1.66E-214	Trypsin CFT-1; similar with TRYP_CHOFU
Px010975.1	2.2277	1.85E-08	9.63E-08	Trypsin, alkaline C; similar with TRYC_MANSE
Px011887.1	2.1323	9.63E-147	4.78E-145	Trypsin, alkaline C; similar with TRYC_MANSE
Px009488.1	2.0714	3.01E-06	1.24E-05	Chymotrypsin-like serine proteinase; similar with CTRL_HALRU
Px009088.1	2.0671	8.40E-275	6.89E-273	Mastin; similar with TRYM_CANFA
Px007621.1	2.0663	1.43E-07	6.93E-07	Trypsin CFT-1; similar with TRYP_CHOFU
Px003670.2	2.0614	0.00E+00	0.00E+00	Serine protease easter; similar with EAST_DROME
Px002441.1	1.9871	4.10E-39	7.84E-38	Trypsin, alkaline B; similar with TRYB_MANSE
Px006571.1	1.9668	0.00E+00	0.00E+00	Trypsin, alkaline B; similar with TRYB_MANSE
Px008538.1	1.9379	5.30E-72	1.61E-70	Trypsin, alkaline B; similar with TRYB_MANSE
Px011231.1	1.8761	1.79E-17	1.77E-16	Protein unc-50 homolog; similar with UNC50_DROME
Px015278.1	1.8699	3.08E-11	2.04E-10	Trypsin, alkaline A; similar with TRYA_MANSE
Px014830.1	1.8574	0.00E+00	0.00E+00	Tryptase; similar with TRYB1_MOUSE
Px015420.2	1.8484	1.34E-11	9.04E-11	Putative uncharacterized protein; similar with Q171T2_AEDAE
Px009568.1	1.8431	6.70E-05	2.27E-04	Trypsin, alkaline C; similar with TRYC_MANSE
Px011499.2	1.7910	6.11E-19	6.55E-18	Serine protease persephone; similar with PSH_DROME
Px001833.1	1.7864	0.00E+00	0.00E+00	Serine protease easter; similar with EAST_DROME
Px017515.1	1.7186	1.09E-80	3.60E-79	Trypsin, alkaline C; similar with TRYC_MANSE
Px007598.1	1.6692	9.57E-31	1.50E-29	Chymotrypsin-1; similar with CTR1_ANOGA
Px002457.1	1.6591	3.55E-58	9.23E-57	Protein dpy-19 homolog 1; similar with D19L1_HUMAN
Px005240.1	1.6409	7.98E-17	7.63E-16	Trypsin, alkaline A; similar with TRYA_MANSE
Px005242.1	1.6327	5.68E-10	3.38E-09	Trypsin CFT-1; similar with TRYP_CHOFU
Px000107.1	1.5952	7.26E-250	5.34E-248	Trypsin, alkaline C; similar with TRYC_MANSE
Px014882.1	1.5792	1.59E-27	2.28E-26	Trypsin, alkaline C; similar with TRYC_MANSE
Px015275.1	1.5751	0.00E+00	0.00E+00	Trypsin CFT-1; similar with TRYP_CHOFU
Px007616.1	1.5713	2.75E-83	9.34E-82	Trypsin CFT-1; similar with TRYP_CHOFU
Px006837.1	1.5701	7.52E-08	3.72E-07	Serine/threonine-protein kinase pelle; similar with KPEL_DROME
Px006572.1	1.5612	0.00E+00	0.00E+00	Trypsin; similar with TRYP_PHACE
Px013377.1	1.5480	1.88E-43	3.82E-42	Trypsin; similar with TRYP_PHACE
Px016057.1	1.5292	2.72E-23	3.45E-22	Trypsin, alkaline B; similar with TRYB_MANSE
Px005936.2	1.5008	1.12E-40	2.19E-39	Trypsin, alkaline C; similar with TRYC_MANSE
Px005243.1	1.4820	0.00E+00	0.00E+00	Trypsin, alkaline A; similar with TRYA_MANSE
Px012568.1	1.4572	4.65E-123	2.06E-121	Trypsin CFT-1; similar with TRYP_CHOFU
Px005765.1	1.4516	5.48E-18	5.59E-17	Vitellin-degrading protease; similar with VDP_BOMMO
Px002440.1	1.4335	9.19E-09	4.93E-08	Trypsin, alkaline B; similar with TRYB_MANSE
Px010499.1	1.4285	6.21E-12	4.30E-11	Serine/threonine-protein kinase pelle; similar with KPEL_DROME
Px014534.1	1.4237	7.49E-32	1.21E-30	Trypsin CFT-1; similar with TRYP_CHOFU
Px016058.1	1.3964	2.20E-207	1.39E-205	Trypsin, alkaline C; similar with TRYC_MANSE
Px003671.1	1.3824	8.09E-185	4.69E-183	Serine protease easter; similar with EAST_DROME
Px009638.1	1.3782	0.00E+00	0.00E+00	Trypsin, alkaline B; similar with TRYB_MANSE
Px014831.2	1.3474	0.00E+00	0.00E+00	Serine proteinase stubble; similar with STUB_DROME
Px016054.1	1.3362	5.17E-08	2.60E-07	Trypsin; similar with TRYP_PHACE
Px003725.1	1.3049	1.92E-25	2.59E-24	Interleukin-1 receptor-associated kinase 4; similar with IRAK4_BOVIN
Px009639.1	1.2774	0.00E+00	0.00E+00	Trypsin CFT-1; similar with TRYP_CHOFU

Px002588.1	1.2360	9.87E-07	4.33E-06	Trypsin, alkaline C; similar with TRYC_MANSE
Px010215.1	1.1967	4.17E-08	2.11E-07	GK12675; similar with B4N3B5_DROWI
Px009395.1	1.1916	1.32E-05	5.00E-05	Proclotting enzyme; similar with PCE_TACTR
Px011886.1	1.1422	3.65E-31	5.79E-30	Trypsin CFT-1; similar with TRYP_CHOFU
Px016056.1	1.1375	0.00E+00	0.00E+00	Trypsin, alkaline A; similar with TRYA_MANSE
Px007552.1	1.1328	6.14E-24	7.92E-23	Paramyosin, putative; similar with E0VGP7_PEDHC
Px013299.1	1.1172	3.69E-82	1.25E-80	Serine proteinase stubble; similar with STUB_DROME
Px011525.1	1.1167	5.60E-05	1.93E-04	Programmed cell death protein 4; similar with PDCD4_CHICK
Px000402.2	1.1008	8.17E-19	8.68E-18	Serine protease persephone; similar with PSH_DROME
Px012329.1	1.0969	2.64E-32	4.31E-31	Serine protease persephone; similar with PSH_DROME
Px007599.1	1.0927	0.00E+00	0.00E+00	Chymotrypsin-1; similar with CTR1_ANOGA
Px008682.1	1.0369	3.84E-06	1.57E-05	Coagulation factor XI; similar with FA11_HUMAN
Px007600.1	1.0192	1.33E-52	3.21E-51	Chymotrypsin-1; similar with CTR1_ANOGA
Px013146.1	-9.6079	4.14E-05	1.46E-04	Trypsin; similar with TRYP_PHACE
Px012497.1	-9.3373	1.47E-04	4.73E-04	Trypsin-4; similar with TRY4_ANOGA
Px006973.1	-9.2055	1.16E-05	4.44E-05	N-chimaerin; similar with CHIN_MOUSE
Px017492.1	-8.9602	3.86E-08	1.96E-07	Trypsin CFT-1; similar with TRYP_CHOFU
Px001873.1	-3.7809	4.04E-07	1.86E-06	Probable palmitoyltransferase ZDHHC8; similar with ZDHC8_MOUSE
Px008406.1	-2.8856	5.90E-05	2.02E-04	Serine proteinase stubble; similar with STUB_DROME
Px016566.1	-2.8663	2.04E-12	1.47E-11	Stress-activated protein kinase JNK; similar with JNK_DROME
Px017197.1	-2.4334	9.91E-10	5.78E-09	Importin subunit alpha-2; similar with IMA2_XENLA
Px006373.1	-2.0343	3.88E-11	2.54E-10	Exportin-6; similar with XPO6_HUMAN
Px008253.1	-2.0121	2.22E-15	1.95E-14	Dual specificity mitogen-activated protein kinase kinase
Px016551.1	-1.9820	6.52E-11	4.20E-10	ADP,ATP carrier protein; similar with ADT_DROME
Px007415.2	-1.9501	8.10E-19	8.60E-18	Furin-like protease 1, isoforms 1/1-X/2; similar with FUR11_DROME
Px015661.1	-1.8624	5.93E-05	2.03E-04	Putative uncharacterized protein; similar with D6WEM6_TRICA
Px011097.1	-1.8357	1.53E-15	1.35E-14	Serine proteinase stubble; similar with STUB_DROME
Px009586.1	-1.6809	2.27E-12	1.63E-11	Phosphatidylinositol 3-kinase regulatory subunit gamma
Px008563.1	-1.6326	5.35E-37	9.82E-36	Interferon-inducible double stranded RNA-dependent protein kinase activator A
Px005341.1	-1.4819	1.93E-101	7.46E-100	Collagenase; similar with COGS_HYPLI
Px007700.1	-1.4344	2.81E-13	2.14E-12	Nuclear RNA export factor 1; similar with NXF1_DROME
Px008372.1	-1.4221	6.98E-10	4.12E-09	Phosphatidylinositol-4,5-bisphosphate 3-kinase catalytic subunit beta isoform
Px011968.1	-1.3855	8.91E-08	4.39E-07	Serine/threonine-protein kinase gcn2; similar with GCN2_SCHPO
Px014044.1	-1.2823	1.15E-58	3.02E-57	Putative uncharacterized protein; similar with C4JWW1_UNCRE
Px016832.3	-1.2819	2.88E-10	1.77E-09	Stress-activated protein kinase JNK; similar with JNK_DROME
Px008701.1	-1.2418	6.12E-09	3.33E-08	Nuclear RNA export factor 1; similar with NXF1_BOVIN
Px017224.1	-1.2107	1.29E-11	8.76E-11	Chaoptin; similar with CHAO_DROME
Px001125.2	-1.2097	1.33E-10	8.38E-10	
Px007910.1	-1.1600	6.95E-41	1.37E-39	Glycogen synthase kinase-3 beta; similar with GSK3B_MOUSE
Px005271.1	-1.0149	8.65E-23	1.08E-21	Putative cuticle protein; similar with C0H6P8_BOMMO
Px001239.1	-1.0036	9.68E-09	5.19E-08	Serine protease P162; similar with D2A5Y8_TRICA
Px001973.2	-1.0031	2.49E-05	9.07E-05	Tetratricopeptide repeat protein 5; similar with TTC5_HUMAN
**Involved in pancreatic secretion**				
Px015280.1	10.8687	2.87E-10	1.76E-09	Trypsin CFT-1; similar with TRYP_CHOFU
Px007357.2	6.6517	0.00E+00	0.00E+00	Serine proteinase stubble; similar with STUB_DROME
Px006254.1	4.4212	6.36E-06	2.52E-05	Chymotrypsin-2; similar with CTR2_ANOGA
Px007620.1	3.6327	1.75E-08	9.13E-08	Trypsin CFT-1; similar with TRYP_CHOFU
Px016055.1	3.2151	6.89E-29	1.03E-27	Trypsin, alkaline C; similar with TRYC_MANSE
Px003546.1	3.1733	2.80E-04	8.56E-04	Trypsin, alkaline C; similar with TRYC_MANSE
Px005387.1	2.8362	1.64E-04	5.24E-04	85 kDa calcium-independent phospholipase A2
Px010386.1	2.6772	0.00E+00	0.00E+00	Trypsin CFT-1; similar with TRYP_CHOFU
Px015277.1	2.5730	1.28E-54	3.19E-53	Trypsin CFT-1; similar with TRYP_CHOFU
Px015276.1	2.5579	1.86E-30	2.90E-29	Trypsin CFT-1; similar with TRYP_CHOFU

Px006441.1	2.4123	0.00E+00	0.00E+00	Lipase member H; similar with LIPH_RAT
Px007619.2	2.3494	0.00E+00	0.00E+00	Trypsin, alkaline A; similar with TRYA_MANSE
Px014769.1	2.3190	3.12E-54	7.70E-53	Serine proteinase stubble; similar with STUB_DROME
Px005241.1	2.3004	9.73E-13	7.17E-12	Trypsin CFT-1; similar with TRYP_CHOFU
Px009569.1	2.2896	2.53E-216	1.66E-214	Trypsin CFT-1; similar with TRYP_CHOFU
Px010975.1	2.2277	1.85E-08	9.63E-08	Trypsin, alkaline C; similar with TRYC_MANSE
Px011887.1	2.1323	9.63E-147	4.78E-145	Trypsin, alkaline C; similar with TRYC_MANSE
Px009488.1	2.0714	3.01E-06	1.24E-05	Chymotrypsin-like serine proteinase; similar with CTRL_HALRU
Px009088.1	2.0671	8.40E-275	6.89E-273	Mastin; similar with TRYM_CANFA
Px007621.1	2.0663	1.43E-07	6.93E-07	Trypsin CFT-1; similar with TRYP_CHOFU
Px003670.2	2.0614	0.00E+00	0.00E+00	Serine protease easter; similar with EAST_DROME
Px002441.1	1.9871	4.10E-39	7.84E-38	Trypsin, alkaline B; similar with TRYB_MANSE
Px006571.1	1.9668	0.00E+00	0.00E+00	Trypsin, alkaline B; similar with TRYB_MANSE
Px008538.1	1.9379	5.30E-72	1.61E-70	Trypsin, alkaline B; similar with TRYB_MANSE
Px011231.1	1.8761	1.79E-17	1.77E-16	Protein unc-50 homolog; similar with UNC50_DROME
Px015278.1	1.8699	3.08E-11	2.04E-10	Trypsin, alkaline A; similar with TRYA_MANSE
Px014830.1	1.8574	0.00E+00	0.00E+00	Tryptase; similar with TRYB1_MOUSE
Px009568.1	1.8431	6.70E-05	2.27E-04	Trypsin, alkaline C; similar with TRYC_MANSE
Px012011.2	1.8280	7.99E-205	5.04E-203	Pancreatic triacylglycerol lipase; similar with LIPP_MYOCO
Px001990.2	1.7922	1.40E-22	1.73E-21	Carboxypeptidase B; similar with CBPB_ASTFL
Px011499.2	1.7910	6.11E-19	6.55E-18	Serine protease persephone; similar with PSH_DROME
Px001833.1	1.7864	0.00E+00	0.00E+00	Serine protease easter; similar with EAST_DROME
Px017515.1	1.7186	1.09E-80	3.60E-79	Trypsin, alkaline C; similar with TRYC_MANSE
Px007598.1	1.6692	9.57E-31	1.50E-29	Chymotrypsin-1; similar with CTR1_ANOGA
Px005240.1	1.6409	7.98E-17	7.63E-16	Trypsin, alkaline A; similar with TRYA_MANSE
Px005242.1	1.6327	5.68E-10	3.38E-09	Trypsin CFT-1; similar with TRYP_CHOFU
Px000107.1	1.5952	7.26E-250	5.34E-248	Trypsin, alkaline C; similar with TRYC_MANSE
Px000104.1	1.5842	4.20E-11	2.75E-10	Pancreatic lipase-related protein 2; similar with LIPR2_CAVPO
Px014882.1	1.5792	1.59E-27	2.28E-26	Trypsin, alkaline C; similar with TRYC_MANSE
Px015275.1	1.5751	0.00E+00	0.00E+00	Trypsin CFT-1; similar with TRYP_CHOFU
Px007616.1	1.5713	2.75E-83	9.34E-82	Trypsin CFT-1; similar with TRYP_CHOFU
Px006572.1	1.5612	0.00E+00	0.00E+00	Trypsin; similar with TRYP_PHACE
Px013377.1	1.5480	1.88E-43	3.82E-42	Trypsin; similar with TRYP_PHACE
Px016057.1	1.5292	2.72E-23	3.45E-22	Trypsin, alkaline B; similar with TRYB_MANSE
Px012048.1	1.5055	2.98E-12	2.12E-11	Chymotrypsin BI; similar with CTRB1_LITVA
Px005936.2	1.5008	1.12E-40	2.19E-39	Trypsin, alkaline C; similar with TRYC_MANSE
Px000996.2	1.4897	7.66E-31	1.21E-29	Carboxypeptidase A2; similar with CBPA2_MOUSE
Px005243.1	1.4820	0.00E+00	0.00E+00	Trypsin, alkaline A; similar with TRYA_MANSE
Px012568.1	1.4572	4.65E-123	2.06E-121	Trypsin CFT-1; similar with TRYP_CHOFU
Px005765.1	1.4516	5.48E-18	5.59E-17	Vitellin-degrading protease; similar with VDP_BOMMO
Px002440.1	1.4335	9.19E-09	4.93E-08	Trypsin, alkaline B; similar with TRYB_MANSE
Px014534.1	1.4237	7.49E-32	1.21E-30	Trypsin CFT-1; similar with TRYP_CHOFU
Px016058.1	1.3964	2.20E-207	1.39E-205	Trypsin, alkaline C; similar with TRYC_MANSE
Px003671.1	1.3824	8.09E-185	4.69E-183	Serine protease easter; similar with EAST_DROME
Px009638.1	1.3782	0.00E+00	0.00E+00	Trypsin, alkaline B; similar with TRYB_MANSE
Px014831.2	1.3474	0.00E+00	0.00E+00	Serine proteinase stubble; similar with STUB_DROME
Px000991.6	1.3435	4.35E-64	1.21E-62	Carboxypeptidase O; similar with CBPO_BOVIN
Px016054.1	1.3362	5.17E-08	2.60E-07	Trypsin; similar with TRYP_PHACE
Px007900.1	1.2849	0.00E+00	0.00E+00	Chymotrypsin BI; similar with CTRB1_LITVA
Px009639.1	1.2774	0.00E+00	0.00E+00	Trypsin CFT-1; similar with TRYP_CHOFU
Px002588.1	1.2360	9.87E-07	4.33E-06	Trypsin, alkaline C; similar with TRYC_MANSE
Px006190.1	1.2352	0.00E+00	0.00E+00	Lipase member H-A; similar with LIPHA_XENLA
Px004751.1	1.2094	5.26E-200	3.23E-198	Pancreatic triacylglycerol lipase; similar with LIPP_HUMAN
Px004750.1	1.2088	0.00E+00	0.00E+00	Pancreatic lipase-related protein 2; similar with LIPR2_RAT

Px009395.1	1.1916	1.32E-05	5.00E-05	Proclotting enzyme; similar with PCE_TACTR
Px003352.1	1.1571	6.22E-06	2.47E-05	Venom carboxylesterase-6; similar with EST6_APIME
Px011886.1	1.1422	3.65E-31	5.79E-30	Trypsin CFT-1; similar with TRYP_CHOFU
Px016056.1	1.1375	0.00E+00	0.00E+00	Trypsin, alkaline A; similar with TRYA_MANSE
Px007013.1	1.1364	1.76E-22	2.18E-21	Group XIIA secretory phospholipase A2; similar with PG12A_MOUSE
Px013299.1	1.1172	3.69E-82	1.25E-80	Serine proteinase stubble; similar with STUB_DROME
Px000402.2	1.1008	8.17E-19	8.68E-18	Serine protease persephone; similar with PSH_DROME
Px012329.1	1.0969	2.64E-32	4.31E-31	Serine protease persephone; similar with PSH_DROME
Px007599.1	1.0927	0.00E+00	0.00E+00	Chymotrypsin-1; similar with CTR1_ANOGA
Px004815.1	1.0516	7.04E-07	3.16E-06	Gut esterase 1; similar with EST1_CAEEL
Px000993.1	1.0428	2.30E-209	1.47E-207	Carboxypeptidase A2; similar with CBPA2_RAT
Px012176.1	1.0278	5.64E-07	2.56E-06	JmjC domain-containing protein 7; similar with JMJD7_MOUSE
Px007600.1	1.0192	1.33E-52	3.21E-51	Chymotrypsin-1; similar with CTR1_ANOGA
Px012191.1	1.0108	5.91E-126	2.65E-124	Sodium/potassium-transporting ATPase subunit alpha-
Px013146.1	-9.6079	4.14E-05	1.46E-04	Trypsin; similar with TRYP_PHACE
Px012497.1	-9.3373	1.47E-04	4.73E-04	Trypsin-4; similar with TRY4_ANOGA
Px017492.1	-8.9602	3.86E-08	1.96E-07	Trypsin CFT-1; similar with TRYP_CHOFU
Px008406.1	-2.8856	5.90E-05	2.02E-04	Serine proteinase stubble; similar with STUB_DROME
Px013802.1	-2.8624	2.15E-07	1.03E-06	Sodium/potassium-transporting ATPase subunit alpha-
Px003127.1	-2.3413	4.59E-06	1.86E-05	Adenylate cyclase type 3; similar with ADCY3_HUMAN
Px002439.1	-2.2418	7.00E-05	2.37E-04	85 kDa calcium-independent phospholipase
Px004992.1	-1.9311	2.49E-05	9.07E-05	Bumetanide-sensitive sodium-(potassium)-chloride cotransporte
Px003969.1	-1.8898	4.45E-12	3.12E-11	Plasma membrane calcium-transporting ATPase
Px005825.1	-1.8565	1.12E-05	4.31E-05	1-phosphatidylinositol-4,5-bisphosphate phosphodiesteras
Px011097.1	-1.8357	1.53E-15	1.35E-14	Serine proteinase stubble; similar with STUB_DROME
Px005762.1	-1.7861	3.28E-16	3.03E-15	Guanine nucleotide-binding protein G(q) subunit alph
Px010907.1	-1.7829	6.97E-137	3.29E-135	Putative uncharacterized protein; similar with D6WJ01_TRICA
Px015790.1	-1.6407	8.72E-18	8.82E-17	Sodium/potassium-transporting ATPase subunit beta-
Px003776.1	-1.4897	2.57E-06	1.07E-05	TBC1 domain family member 30; similar with TBC30_XENTR
Px005341.1	-1.4819	1.93E-101	7.46E-100	Collagenase; similar with COGS_HYPLI
Px003970.1	-1.4540	0.00E+00	0.00E+00	Plasma membrane calcium-transporting ATPase
Px001942.1	-1.3215	3.99E-50	9.31E-49	Carbonic anhydrase 2; similar with CAH2_MOUSE
Px000263.1	-1.2220	3.11E-07	1.45E-06	Ras-related protein Rac1; similar with RAC1_DROME
Px016332.1	-1.2199	0.00E+00	0.00E+00	Calcium-transporting ATPase sarcoplasmic/endoplasmic reticulum typ
Px011757.1	-1.1422	5.15E-16	4.70E-15	Esterase FE4; similar with ESTF_MYZPE
Px005612.4	-1.1210	6.66E-20	7.47E-19	Calcium-activated potassium channel slowpoke; similar with SLO_DROME
Px004044.1	-1.0654	5.33E-21	6.26E-20	Juvenile hormone esterase; similar with ESTJ_HELVI
Px001239.1	-1.0036	9.68E-09	5.19E-08	Serine protease P162; similar with D2A5Y8_TRICA

Involved in neuroactive ligand-receptor interaction

Px015280.1	10.8687	2.87E-10	1.76E-09	Trypsin CFT-1; similar with TRYP_CHOFU
Px014235.1	8.8173	2.67E-08	1.37E-07	Putative chemosensory ionotropic receptor IR41a
Px007357.2	6.6517	0.00E+00	0.00E+00	Serine proteinase stubble; similar with STUB_DROME
Px006831.1	6.1494	0.00E+00	0.00E+00	Putative uncharacterized protein; similar with D6X0U7_TRICA
Px006254.1	4.4212	6.36E-06	2.52E-05	Chymotrypsin-2; similar with CTR2_ANOGA
Px008696.1	4.2465	3.05E-26	4.22E-25	Osiris 9; similar with B6DXB0_BOMMO
Px007620.1	3.6327	1.75E-08	9.13E-08	Trypsin CFT-1; similar with TRYP_CHOFU
Px016055.1	3.2151	6.89E-29	1.03E-27	Trypsin, alkaline C; similar with TRYC_MANSE
Px003546.1	3.1733	2.80E-04	8.56E-04	Trypsin, alkaline C; similar with TRYC_MANSE
Px000336.1	2.9575	1.37E-12	9.98E-12	Osiris 20; similar with B6DXA7_BOMMO
Px010386.1	2.6772	0.00E+00	0.00E+00	Trypsin CFT-1; similar with TRYP_CHOFU
Px006629.1	2.6327	9.17E-05	3.04E-04	Retrotransposable element Tf2 155 kDa protein type 3
Px015277.1	2.5730	1.28E-54	3.19E-53	Trypsin CFT-1; similar with TRYP_CHOFU
Px015276.1	2.5579	1.86E-30	2.90E-29	Trypsin CFT-1; similar with TRYP_CHOFU

Px008529.1	2.4352	3.69E-39	7.07E-38	Thyrotropin-releasing hormone receptor; similar with TRFR_CHICK
Px011101.1	2.3787	5.60E-77	1.78E-75	Coagulation factor X; similar with FA10_RAT
Px007619.2	2.3494	0.00E+00	0.00E+00	Trypsin, alkaline A; similar with TRYA_MANSE
Px014769.1	2.3190	3.12E-54	7.70E-53	Serine proteinase stubble; similar with STUB_DROME
Px005241.1	2.3004	9.73E-13	7.17E-12	Trypsin CFT-1; similar with TRYP_CHOFU
Px009569.1	2.2896	2.53E-216	1.66E-214	Trypsin CFT-1; similar with TRYP_CHOFU
Px010975.1	2.2277	1.85E-08	9.63E-08	Trypsin, alkaline C; similar with TRYC_MANSE
Px016100.1	2.1921	3.26E-21	3.85E-20	Coagulation factor IX; similar with FA9_RABIT
Px011887.1	2.1323	9.63E-147	4.78E-145	Trypsin, alkaline C; similar with TRYC_MANSE
Px009488.1	2.0714	3.01E-06	1.24E-05	Chymotrypsin-like serine proteinase; similar with CTRL_HALRU
Px009088.1	2.0671	8.40E-275	6.89E-273	Mastin; similar with TRYM_CANFA
Px007621.1	2.0663	1.43E-07	6.93E-07	Trypsin CFT-1; similar with TRYP_CHOFU
Px003670.2	2.0614	0.00E+00	0.00E+00	Serine protease easter; similar with EAST_DROME
Px002441.1	1.9871	4.10E-39	7.84E-38	Trypsin, alkaline B; similar with TRYB_MANSE
Px006571.1	1.9668	0.00E+00	0.00E+00	Trypsin, alkaline B; similar with TRYB_MANSE
Px008538.1	1.9379	5.30E-72	1.61E-70	Trypsin, alkaline B; similar with TRYB_MANSE
Px011231.1	1.8761	1.79E-17	1.77E-16	Protein unc-50 homolog; similar with UNC50_DROME
Px015278.1	1.8699	3.08E-11	2.04E-10	Trypsin, alkaline A; similar with TRYA_MANSE
Px014830.1	1.8574	0.00E+00	0.00E+00	Tryptase; similar with TRYB1_MOUSE
Px009568.1	1.8431	6.70E-05	2.27E-04	Trypsin, alkaline C; similar with TRYC_MANSE
Px011499.2	1.7910	6.11E-19	6.55E-18	Serine protease persephone; similar with PSH_DROME
Px001833.1	1.7864	0.00E+00	0.00E+00	Serine protease easter; similar with EAST_DROME
Px007383.1	1.7582	1.80E-04	5.69E-04	PDF receptor; similar with PDFR_DROME
Px017515.1	1.7186	1.09E-80	3.60E-79	Trypsin, alkaline C; similar with TRYC_MANSE
Px007598.1	1.6692	9.57E-31	1.50E-29	Chymotrypsin-1; similar with CTR1_ANOGA
Px005240.1	1.6409	7.98E-17	7.63E-16	Trypsin, alkaline A; similar with TRYA_MANSE
Px005242.1	1.6327	5.68E-10	3.38E-09	Trypsin CFT-1; similar with TRYP_CHOFU
Px000107.1	1.5952	7.26E-250	5.34E-248	Trypsin, alkaline C; similar with TRYC_MANSE
Px014882.1	1.5792	1.59E-27	2.28E-26	Trypsin, alkaline C; similar with TRYC_MANSE
Px015275.1	1.5751	0.00E+00	0.00E+00	Trypsin CFT-1; similar with TRYP_CHOFU
Px007616.1	1.5713	2.75E-83	9.34E-82	Trypsin CFT-1; similar with TRYP_CHOFU
Px006572.1	1.5612	0.00E+00	0.00E+00	Trypsin; similar with TRYP_PHACE
Px013377.1	1.5480	1.88E-43	3.82E-42	Trypsin; similar with TRYP_PHACE
Px016057.1	1.5292	2.72E-23	3.45E-22	Trypsin, alkaline B; similar with TRYB_MANSE
Px005936.2	1.5008	1.12E-40	2.19E-39	Trypsin, alkaline C; similar with TRYC_MANSE
Px005243.1	1.4820	0.00E+00	0.00E+00	Trypsin, alkaline A; similar with TRYA_MANSE
Px012568.1	1.4572	4.65E-123	2.06E-121	Trypsin CFT-1; similar with TRYP_CHOFU
Px005765.1	1.4516	5.48E-18	5.59E-17	Vitellin-degrading protease; similar with VDP_BOMMO
Px002440.1	1.4335	9.19E-09	4.93E-08	Trypsin, alkaline B; similar with TRYB_MANSE
Px000584.1	1.4237	7.49E-32	1.21E-30	Coagulation factor X; similar with FA10_RAT
Px014534.1	1.4237	7.49E-32	1.21E-30	Trypsin CFT-1; similar with TRYP_CHOFU
Px016058.1	1.3964	2.20E-207	1.39E-205	Trypsin, alkaline C; similar with TRYC_MANSE
Px003671.1	1.3824	8.09E-185	4.69E-183	Serine protease easter; similar with EAST_DROME
Px009638.1	1.3782	0.00E+00	0.00E+00	Trypsin, alkaline B; similar with TRYB_MANSE
Px014831.2	1.3474	0.00E+00	0.00E+00	Serine proteinase stubble; similar with STUB_DROME
Px016054.1	1.3362	5.17E-08	2.60E-07	Trypsin; similar with TRYP_PHACE
Px008729.3	1.3325	1.21E-05	4.60E-05	Retrotransposable element Tf2 155 kDa protein type
Px009639.1	1.2774	0.00E+00	0.00E+00	Trypsin CFT-1; similar with TRYP_CHOFU
Px005349.1	1.2454	7.44E-11	4.78E-10	G-protein coupled receptor moody; similar with MOODY_DROME
Px002588.1	1.2360	9.87E-07	4.33E-06	Trypsin, alkaline C; similar with TRYC_MANSE
Px005447.1	1.2327	3.07E-47	6.74E-46	Gonadotropin-releasing hormone receptor; similar with GNRHR_OCTVU
Px009395.1	1.1916	1.32E-05	5.00E-05	Proclotting enzyme; similar with PCE_TACTR
Px011886.1	1.1422	3.65E-31	5.79E-30	Trypsin CFT-1; similar with TRYP_CHOFU
Px016056.1	1.1375	0.00E+00	0.00E+00	Trypsin, alkaline A; similar with TRYA_MANSE

Px013299.1	1.1172	3.69E-82	1.25E-80	Serine proteinase stubble; similar with STUB_DROME
Px000402.2	1.1008	8.17E-19	8.68E-18	Serine protease persephone; similar with PSH_DROME
Px012329.1	1.0969	2.64E-32	4.31E-31	Serine protease persephone; similar with PSH_DROME
Px007599.1	1.0927	0.00E+00	0.00E+00	Chymotrypsin-1; similar with CTR1_ANOGA
Px007600.1	1.0192	1.33E-52	3.21E-51	Chymotrypsin-1; similar with CTR1_ANOGA
Px013146.1	-9.6079	4.14E-05	1.46E-04	Trypsin; similar with TRYP_PHACE
Px012497.1	-9.3373	1.47E-04	4.73E-04	Trypsin-4; similar with TRY4_ANOGA
Px017492.1	-8.9602	3.86E-08	1.96E-07	Trypsin CFT-1; similar with TRYP_CHOFU
Px007162.1	-8.7968	1.16E-05	4.44E-05	5-hydroxytryptamine receptor 2B; similar with 5HT2B_TETFL
Px011547.1	-3.3292	4.37E-07	2.00E-06	Putative uncharacterized protein; similar with D6X0T4_TRICA
Px008406.1	-2.8856	5.90E-05	2.02E-04	Serine proteinase stubble; similar with STUB_DROME
Px014294.1	-2.8267	3.64E-07	1.69E-06	FMRFamide receptor; similar with FMAR_DROME
Px015638.2	-2.7012	2.86E-04	8.73E-04	Transposon Ty3-I Gag-Pol polyprotein; similar with YI31B_YEAST
Px002356.2	-2.5272	1.32E-04	4.25E-04	Glutamate receptor, ionotropic kainate 2; similar with GRIK2_MOUSE
Px003831.1	-2.5272	1.32E-04	4.25E-04	Transposon Ty3-G Gag-Pol polyprotein; similar with YG31B_YEAST
Px011383.1	-2.5148	7.29E-09	3.95E-08	Glutamate receptor, ionotropic kainate 3; similar with GRIK3_HUMAN
Px002244.1	-2.4897	2.31E-23	2.93E-22	Transposon Ty3-I Gag-Pol polyprotein; similar with YI31B_YEAST
Px006189.1	-2.4203	2.06E-22	2.53E-21	5-hydroxytryptamine receptor 1; similar with 5HT1R_DROME
Px008288.1	-1.9967	3.27E-10	1.99E-09	Glutamate [NMDA] receptor subunit 3A; similar with NMD3A_HUMAN
Px011097.1	-1.8357	1.53E-15	1.35E-14	Serine proteinase stubble; similar with STUB_DROME
Px000929.1	-1.6126	1.03E-08	5.48E-08	Octopamine receptor beta-2R; similar with OCTB2_DROME
Px011491.5	-1.5234	4.16E-14	3.35E-13	Rhodopsin, GQ-coupled; similar with OPSD1_MIZYE
Px005341.1	-1.4819	1.93E-101	7.46E-100	Collagenase; similar with COGS_HYPLI
Px004913.1	-1.3923	3.26E-212	2.11E-210	Putative uncharacterized protein; similar with D6W9I4_TRICA
Px003827.2	-1.3590	4.00E-12	2.81E-11	Cell surface glycoprotein 1; similar with SLAP1_CLOTH
Px001307.1	-1.3337	2.77E-04	8.50E-04	Dehydrogenase/reductase SDR family member 1
Px010138.1	-1.3242	6.26E-12	4.33E-11	Probable G-protein coupled receptor B0563.6; similar with YT66_CAEEL
Px010923.3	-1.3232	1.15E-05	4.41E-05	Putative uncharacterized protein; similar with Q17GL6_AEDAE
Px002306.1	-1.2862	1.81E-09	1.03E-08	Calcitonin receptor; similar with CALCR_RABIT
Px009076.2	-1.1289	1.13E-10	7.15E-10	Exportin-7; similar with XPO7_CHICK
Px004308.1	-1.1024	4.07E-05	1.43E-04	Uncharacterized protein K02A2.6; similar with YRD6_CAEEL
Px016265.1	-1.0491	1.67E-04	5.31E-04	Glutamate receptor, ionotropic kainate 3; similar with GRIK3_MACFA
Px015204.1	-1.0183	0.00E+00	0.00E+00	Transposon Ty3-I Gag-Pol polyprotein; similar with YI31B_YEAST
Px001239.1	-1.0036	9.68E-09	5.19E-08	Serine protease P162; similar with D2A5Y8_TRICA

Table C. A list of DEGs enriched in the *P. xylostella* pathways at 36 hpt_I versus 36 hpt_C.

Gene ID	log_2 R	P-value	FDR	Annotation
**Involved in Pancreatic secretion**				
Px000644.1	12.4260	4.78E-37	4.70E-36	Pancreatic triacylglycerol lipase; similar with LIPP_MOUSE
Px012560.1	10.5968	1.26E-07	4.83E-07	Lipase member H-B; similar with LIPHB_XENLA
Px007620.1	10.3950	8.15E-10	3.63E-09	Trypsin CFT-1; similar with TRYP_CHOFU
Px002495.1	10.1815	7.08E-09	2.96E-08	Carbonic anhydrase 5A, mitochondrial; similar with CAH5A_RAT
Px006069.1	10.0136	2.26E-06	7.79E-06	Trypsin CFT-1; similar with TRYP_CHOFU
Px013802.1	8.5527	1.46E-08	5.93E-08	Sodium/potassium-transporting ATPase subunit alpha-2
Px008830.1	6.6433	1.43E-111	2.90E-110	Trypsin, alkaline C; similar with TRYC_MANSE
Px001804.1	6.5119	2.83E-51	3.39E-50	Trypsin, alkaline B; similar with TRYB_MANSE
Px007670.1	6.2472	5.75E-63	7.92E-62	Proclotting enzyme; similar with PCE_TACTR
Px007357.2	5.3379	0.00E+00	0.00E+00	Serine proteinase stubble; similar with STUB_DROME
Px010971.1	5.2472	1.05E-10	4.92E-10	Trypsin CFT-1; similar with TRYP_CHOFU
Px005240.1	5.1310	8.24E-124	1.79E-122	Trypsin, alkaline A; similar with TRYA_MANSE
Px014882.1	4.8506	4.25E-162	1.13E-160	Trypsin, alkaline C; similar with TRYC_MANSE
Px005554.1	4.6008	8.20E-07	2.93E-06	Chymotrypsin-1; similar with CTR1_ANOGA
Px016055.1	4.5497	4.52E-56	5.70E-55	Trypsin, alkaline C; similar with TRYC_MANSE
Px002864.1	4.5298	1.55E-103	3.01E-102	Trypsin, alkaline B; similar with TRYB_MANSE
Px000995.2	4.3915	0.00E+00	0.00E+00	Carboxypeptidase A4; similar with CBPA4_HUMAN
Px005241.1	4.3794	1.43E-53	1.74E-52	Trypsin CFT-1; similar with TRYP_CHOFU
Px007598.1	4.3759	1.54E-119	3.27E-118	Chymotrypsin-1; similar with CTR1_ANOGA
Px016053.1	4.2124	5.01E-59	6.53E-58	Trypsin; similar with TRYP_PHACE
Px011791.1	4.1145	0.00E+00	0.00E+00	Lipase member H; similar with LIPH_XENTR
Px009637.1	4.1028	1.35E-27	1.13E-26	Trypsin CFT-1; similar with TRYP_CHOFU
Px012011.2	4.0620	0.00E+00	0.00E+00	Pancreatic triacylglycerol lipase; similar with LIPP_MYOCO
Px002586.1	4.0373	5.30E-58	6.84E-57	Trypsin CFT-1; similar with TRYP_CHOFU
Px013377.1	4.0330	2.06E-219	6.86E-218	Trypsin; similar with TRYP_PHACE
Px015276.1	4.0315	9.55E-65	1.33E-63	Trypsin CFT-1; similar with TRYP_CHOFU
Px005243.1	3.9876	0.00E+00	0.00E+00	Trypsin, alkaline A; similar with TRYA_MANSE
Px015277.1	3.9698	7.98E-158	2.09E-156	Trypsin CFT-1; similar with TRYP_CHOFU
Px004815.1	3.9017	3.38E-20	2.32E-19	Gut esterase 1; similar with EST1_CAEEL
Px007180.1	3.8846	2.52E-32	2.29E-31	Juvenile hormone esterase; similar with ESTJ_HELVI
Px010313.2	3.7896	2.43E-106	4.78E-105	Serine proteinase stubble; similar with STUB_DROME
Px006571.1	3.7885	0.00E+00	0.00E+00	Trypsin, alkaline B; similar with TRYB_MANSE
Px012813.1	3.6622	2.98E-06	1.02E-05	Serine proteinase stubble; similar with STUB_DROME
Px005239.1	3.6181	0.00E+00	0.00E+00	Trypsin, alkaline A; similar with TRYA_MANSE
Px014201.1	3.6008	7.33E-11	3.48E-10	Sodium/potassium-transporting ATPase subunit alpha-2
Px000992.1	3.6006	0.00E+00	0.00E+00	Carboxypeptidase A1; similar with CBPA1_BOVIN
Px007617.1	3.5958	0.00E+00	0.00E+00	Trypsin CFT-1; similar with TRYP_CHOFU
Px006572.1	3.4867	0.00E+00	0.00E+00	Trypsin; similar with TRYP_PHACE
Px005765.1	3.4325	1.33E-62	1.82E-61	Vitellin-degrading protease; similar with VDP_BOMMO
Px016056.1	3.4297	0.00E+00	0.00E+00	Trypsin, alkaline A; similar with TRYA_MANSE
Px009940.2	3.4221	6.33E-52	7.64E-51	Acetylcholinesterase; similar with ACES_CULPI
Px008538.1	3.4202	3.15E-138	7.39E-137	Trypsin, alkaline B; similar with TRYB_MANSE
Px012048.1	3.3901	4.29E-64	5.96E-63	Chymotrypsin BI; similar with CTRB1_LITVA
Px007621.1	3.3847	6.61E-21	4.65E-20	Trypsin CFT-1; similar with TRYP_CHOFU
Px014592.1	3.3707	9.04E-90	1.58E-88	Trypsin CFT-1; similar with TRYP_CHOFU
Px005238.1	3.3700	4.57E-59	5.96E-58	Trypsin, alkaline B; similar with TRYB_MANSE
Px009046.1	3.3378	8.69E-24	6.54E-23	Carbonic anhydrase 7; similar with CAH7_MOUSE
Px009569.1	3.3179	0.00E+00	0.00E+00	Trypsin CFT-1; similar with TRYP_CHOFU
Px007901.1	3.2912	0.00E+00	0.00E+00	Chymotrypsin BI; similar with CTRB1_LITVA
Px001998.2	3.2879	2.48E-118	5.21E-117	Carboxypeptidase B; similar with CBPB_ASTFL

Px000993.1	3.2755	0.00E+00	0.00E+00	Carboxypeptidase A2; similar with CBPA2_RAT
Px000991.6	3.2679	0.00E+00	0.00E+00	Carboxypeptidase O; similar with CBPO_BOVIN
Px007599.1	3.2567	0.00E+00	0.00E+00	Chymotrypsin-1; similar with CTR1_ANOGA
Px006767.1	3.2472	1.38E-04	3.92E-04	Chymotrypsin-1; similar with E2BNV4_9HYME
Px015279.1	3.2472	1.79E-18	1.17E-17	Trypsin, alkaline A; similar with TRYA_MANSE
Px004168.1	3.2472	2.59E-13	1.38E-12	Plasma kallikrein; similar with KLKB1_HUMAN
Px012568.1	3.2398	0.00E+00	0.00E+00	Trypsin CFT-1; similar with TRYP_CHOFU
Px007900.1	3.2235	0.00E+00	0.00E+00	Chymotrypsin BI; similar with CTRB1_LITVA
Px015278.1	3.1962	2.10E-27	1.75E-26	Trypsin, alkaline A; similar with TRYA_MANSE
Px003352.1	3.1927	1.50E-30	1.32E-29	Venom carboxylesterase-6; similar with EST6_APIME
Px010386.1	3.1714	0.00E+00	0.00E+00	Trypsin CFT-1; similar with TRYP_CHOFU
Px016054.1	3.1536	3.24E-37	3.20E-36	Trypsin; similar with TRYP_PHACE
Px007619.2	3.0951	0.00E+00	0.00E+00	Trypsin, alkaline A; similar with TRYA_MANSE
Px002861.1	3.0431	0.00E+00	0.00E+00	Trypsin, alkaline C; similar with TRYC_MANSE
Px009639.1	3.0376	0.00E+00	0.00E+00	Trypsin CFT-1; similar with TRYP_CHOFU
Px001997.1	3.0350	2.53E-37	2.50E-36	Carboxypeptidase B; similar with CBPB_ASTFL
Px007903.1	2.9954	0.00E+00	0.00E+00	Collagenase; similar with COGS_HYPLI
Px005244.1	2.9796	7.91E-29	6.74E-28	Trypsin CFT-1; similar with TRYP_CHOFU
Px004847.1	2.9467	8.27E-18	5.24E-17	Putative carbonic anhydrase 5; similar with CAH5_CAEEL
Px011683.2	2.9306	0.00E+00	0.00E+00	Venom carboxylesterase-6; similar with EST6_APIME
Px015280.1	2.9138	7.05E-10	3.15E-09	Trypsin CFT-1; similar with TRYP_CHOFU
Px006570.1	2.8972	0.00E+00	0.00E+00	Trypsin, alkaline B; similar with TRYB_MANSE
Px000996.2	2.8881	3.06E-71	4.53E-70	Carboxypeptidase A2; similar with CBPA2_MOUSE
Px008089.1	2.8762	0.00E+00	0.00E+00	Pancreatic lipase-related protein 2; similar with LIPR2_CAVPO
Px006190.1	2.8647	0.00E+00	0.00E+00	Lipase member H-A; similar with LIPHA_XENLA
Px000994.1	2.8631	0.00E+00	0.00E+00	Zinc carboxypeptidase A 1; similar with CBPA1_DROPS
Px016057.1	2.8626	2.00E-77	3.18E-76	Trypsin, alkaline B; similar with TRYB_MANSE
Px009638.1	2.8523	0.00E+00	0.00E+00	Trypsin, alkaline B; similar with TRYB_MANSE
Px011788.2	2.8400	3.24E-222	1.09E-220	Pancreatic lipase-related protein 2; similar with LIPR2_BOVIN
Px002587.1	2.7903	0.00E+00	0.00E+00	Trypsin CFT-1; similar with TRYP_CHOFU
Px005373.1	2.7565	1.52E-39	1.56E-38	Carboxypeptidase B; similar with CBPB_ASTFL
Px017996.1	2.7512	1.00E-177	2.87E-176	Acetylcholinesterase; similar with ACES_HUMAN
Px000107.1	2.7335	0.00E+00	0.00E+00	Trypsin, alkaline C; similar with TRYC_MANSE
Px007618.1	2.7222	1.13E-272	4.41E-271	Trypsin CFT-1; similar with TRYP_CHOFU
Px011887.1	2.7196	0.00E+00	0.00E+00	Trypsin, alkaline C; similar with TRYC_MANSE
Px009286.1	2.7141	0.00E+00	0.00E+00	Trypsin, alkaline C; similar with TRYC_MANSE
Px005936.2	2.7085	3.12E-86	5.29E-85	Trypsin, alkaline C; similar with TRYC_MANSE
Px000089.1	2.6887	2.89E-87	4.95E-86	Acetylcholinesterase; similar with ACES_FELCA
Px002442.1	2.6772	1.51E-16	9.17E-16	Trypsin, alkaline C; similar with TRYC_MANSE
Px002865.1	2.6726	0.00E+00	0.00E+00	Trypsin 5G1; similar with TRY5_AEDAE
Px007902.1	2.6449	5.66E-27	4.65E-26	Collagenase; similar with COGS_HYPLI
Px002441.1	2.6127	4.36E-75	6.75E-74	Trypsin, alkaline B; similar with TRYB_MANSE
Px018074.1	2.6056	3.91E-24	2.97E-23	Chymotrypsin-like elastase family member 2A
Px002735.1	2.6010	1.66E-257	6.15E-256	Esterase FE4; similar with ESTF_MYZPE
Px000395.1	2.6001	0.00E+00	0.00E+00	Alpha-amylase 4N; similar with AM4N_DROAN
Px005193.2	2.5958	2.33E-141	5.61E-140	Pancreatic lipase-related protein 2; similar with LIPR2_MYOCO
Px011889.1	2.5691	1.52E-21	1.09E-20	Achelase-1; similar with ACH1_LONAC
Px007600.1	2.5619	1.13E-226	3.92E-225	Chymotrypsin-1; similar with CTR1_ANOGA
Px007674.2	2.5239	0.00E+00	0.00E+00	Collagenase; similar with COGS_HYPLI
Px007675.1	2.5233	0.00E+00	0.00E+00	Collagenase; similar with COGS_HYPLI
Px006345.1	2.5102	0.00E+00	0.00E+00	Pancreatic lipase-related protein 2; similar with LIPR2_MYOCO
Px002860.1	2.5102	4.25E-17	2.64E-16	Achelase-1; similar with ACH1_LONAC
Px011049.1	2.5090	4.88E-35	4.62E-34	Acetylcholinesterase; similar with ACES_FELCA
Px004750.1	2.4896	0.00E+00	0.00E+00	Pancreatic lipase-related protein 2; similar with LIPR2_RAT

Px002736.1	2.4868	0.00E+00	0.00E+00	Venom carboxylesterase-6; similar with EST6_APIME
Px007677.1	2.4787	0.00E+00	0.00E+00	Trypsin-7; similar with TRY7_ANOGA
Px012582.1	2.4724	2.13E-127	4.68E-126	Collagenase; similar with COGS_HYPLI
Px009488.1	2.4558	6.08E-05	1.81E-04	Chymotrypsin-like serine proteinase; similar with CTRL_HALRU
Px016058.1	2.4185	0.00E+00	0.00E+00	Trypsin, alkaline C; similar with TRYC_MANSE
Px002588.1	2.4151	4.81E-14	2.66E-13	Trypsin, alkaline C; similar with TRYC_MANSE
Px010489.1	2.4085	6.89E-66	9.73E-65	Trypsin, alkaline C; similar with TRYC_MANSE
Px006430.1	2.4038	1.48E-302	6.22E-301	Esterase FE4; similar with ESTF_MYZPE
Px009287.1	2.4027	1.27E-279	5.09E-278	Trypsin CFT-1; similar with TRYP_CHOFU
Px009395.1	2.3871	1.03E-17	6.47E-17	Proclotting enzyme; similar with PCE_TACTR
Px007616.1	2.3746	1.09E-158	2.87E-157	Trypsin CFT-1; similar with TRYP_CHOFU
Px005242.1	2.3668	2.59E-19	1.73E-18	Trypsin CFT-1; similar with TRYP_CHOFU
Px004752.1	2.3527	0.00E+00	0.00E+00	Pancreatic lipase-related protein 2; similar with LIPR2_BOVIN
Px002589.1	2.3435	3.45E-96	6.29E-95	Trypsin, alkaline A; similar with TRYA_MANSE
Px004753.1	2.3410	9.82E-208	3.12E-206	Pancreatic lipase-related protein 2; similar with LIPR2_MYOCO
Px015275.1	2.3286	0.00E+00	0.00E+00	Trypsin CFT-1; similar with TRYP_CHOFU
Px006441.1	2.3113	0.00E+00	0.00E+00	Lipase member H; similar with LIPH_RAT
Px007601.1	2.2954	3.63E-31	3.23E-30	Chymotrypsin-1; similar with CTR1_ANOGA
Px012592.1	2.2775	0.00E+00	0.00E+00	Juvenile hormone esterase; similar with ESTJ_HELVI
Px017515.1	2.2558	2.50E-108	4.98E-107	Trypsin, alkaline C; similar with TRYC_MANSE
Px009567.1	2.2552	3.00E-104	5.83E-103	Trypsin, alkaline B; similar with TRYB_MANSE
Px001990.2	2.2289	2.92E-104	5.69E-103	Carboxypeptidase B; similar with CBPB_ASTFL
Px009566.1	2.2251	1.29E-259	4.84E-258	Trypsin, alkaline C; similar with TRYC_MANSE
Px000771.1	2.1857	1.04E-117	2.18E-116	Trypsin CFT-1; similar with TRYP_CHOFU
Px011886.1	2.1750	1.89E-125	4.14E-124	Trypsin CFT-1; similar with TRYP_CHOFU
Px001239.1	2.1600	2.79E-38	2.80E-37	Serine protease P162; similar with D2A5Y8_TRICA
Px002296.1	2.1474	1.41E-169	3.92E-168	Pancreatic triacylglycerol lipase; similar with LIPP_HUMAN
Px003649.1	2.1363	0.00E+00	0.00E+00	Esterase FE4; similar with ESTF_MYZPE
Px003776.1	2.1246	9.72E-09	4.02E-08	TBC1 domain family member 30; similar with TBC30_XENTR
Px003273.1	2.1129	6.40E-16	3.82E-15	Pancreatic lipase-related protein 2; similar with LIPR2_CAVPO
Px000744.1	2.0773	1.67E-04	4.72E-04	Lipase member H-A; similar with LIPHA_XENLA
Px001833.1	2.0603	0.00E+00	0.00E+00	Serine protease easter; similar with EAST_DROME
Px004167.1	2.0469	1.15E-06	4.06E-06	Plasma kallikrein; similar with KLKB1_MOUSE
Px012569.1	2.0285	0.00E+00	0.00E+00	Trypsin, alkaline C; similar with TRYC_MANSE
Px002440.1	2.0145	2.30E-21	1.64E-20	Trypsin, alkaline B; similar with TRYB_MANSE
Px000515.1	1.9774	1.03E-98	1.91E-97	Esterase FE4; similar with ESTF_MYZPE
Px012570.1	1.9654	0.00E+00	0.00E+00	Trypsin CFT-1; similar with TRYP_CHOFU
Px009775.1	1.9517	8.35E-05	2.44E-04	Two pore calcium channel protein 1; similar with TPC1_RAT
Px011097.1	1.9460	2.65E-10	1.22E-09	Serine proteinase stubble; similar with STUB_DROME
Px004751.1	1.9293	0.00E+00	0.00E+00	Pancreatic triacylglycerol lipase; similar with LIPP_HUMAN
Px002512.1	1.9231	4.29E-29	3.68E-28	Brachyurin; similar with COGS_UCAPU
Px010975.1	1.8944	5.90E-09	2.48E-08	Trypsin, alkaline C; similar with TRYC_MANSE
Px000394.1	1.8666	1.72E-210	5.53E-209	Alpha-amylase 4N; similar with AM4N_DROAN
Px002866.1	1.8658	5.28E-09	2.23E-08	Trypsin, alkaline A; similar with TRYA_MANSE
Px008406.1	1.8639	2.77E-08	1.10E-07	Serine proteinase stubble; similar with STUB_DROME
Px006640.1	1.8615	2.23E-04	6.15E-04	Putative carbonic anhydrase 5; similar with CAH5_CAEEL
Px013665.3	1.8475	2.46E-37	2.43E-36	Collagenase; similar with COGS_HYPLI
Px012329.1	1.7693	5.39E-84	8.95E-83	Serine protease persephone; similar with PSH_DROME
Px002295.1	1.7684	0.00E+00	0.00E+00	Pancreatic lipase-related protein 2; similar with LIPR2_BOVIN
Px011231.1	1.7453	1.76E-27	1.46E-26	Protein unc-50 homolog; similar with UNC50_DROME
Px010017.1	1.7418	0.00E+00	0.00E+00	Carboxypeptidase B; similar with CBPB_ASTFL
Px011755.1	1.7284	1.92E-39	1.96E-38	Antennal esterase CXE4; similar with D3GDL5_SPOLI
Px004235.2	1.7053	1.90E-19	1.28E-18	Putative uncharacterized protein; similar with Q178N6_AEDAE
Px000402.2	1.6821	5.77E-83	9.50E-82	Serine protease persephone; similar with PSH_DROME

Px009088.1	1.6795	5.48E-174	1.55E-172	Mastin; similar with TRYM_CANFA
Px003712.1	1.6490	1.05E-19	7.17E-19	Acetylcholinesterase; similar with ACES_CULPI
Px005342.1	1.6408	3.80E-156	9.82E-155	Collagenase; similar with COGS_HYPLI
Px003251.1	1.6237	1.02E-19	6.98E-19	Putative cuticle protein; similar with C0H6E8_BOMMO
Px017997.1	1.6216	3.59E-04	9.62E-04	Esterase FE4; similar with ESTF_MYZPE
Px007904.1	1.6124	0.00E+00	0.00E+00	Collagenase; similar with COGS_HYPLI
Px001556.1	1.6061	8.94E-117	1.85E-115	Serine protease easter; similar with EAST_DROME
Px013162.1	1.5740	7.41E-151	1.88E-149	Serine protease snake; similar with SNAK_DROME
Px005340.1	1.5499	1.24E-139	2.94E-138	Collagenase; similar with COGS_HYPLI
Px004846.1	1.5437	4.25E-52	5.15E-51	Putative carbonic anhydrase 5; similar with CAH5_CAEEL
Px007772.1	1.5076	2.80E-66	3.96E-65	Esterase B1; similar with EST1_CULPI
Px014702.1	1.5035	1.75E-04	4.92E-04	Lysine-specific demethylase 8; similar with KDM8_RAT
Px014831.2	1.4467	0.00E+00	0.00E+00	Serine proteinase stubble; similar with STUB_DROME
Px012191.1	1.4028	2.38E-208	7.61E-207	Sodium/potassium-transporting ATPase subunit alpha-1
Px009568.1	1.3992	1.23E-04	3.53E-04	Trypsin, alkaline C; similar with TRYC_MANSE
Px005341.1	1.3783	1.92E-45	2.13E-44	Collagenase; similar with COGS_HYPLI
Px005947.1	1.3627	2.45E-06	8.39E-06	Serine proteinase stubble; similar with STUB_DROME
Px000146.1	1.3503	5.61E-05	1.68E-04	Adenylate cyclase type 9; similar with ADCY9_HUMAN
Px007906.2	1.3236	0.00E+00	0.00E+00	Collagenase; similar with COGS_HYPLI
Px012207.1	1.2928	3.22E-34	3.02E-33	Transmembrane protein 205; similar with TM205_MOUSE
Px011499.2	1.1964	1.89E-18	1.23E-17	Serine protease persephone; similar with PSH_DROME
Px011153.3	1.1683	1.07E-26	8.76E-26	Complement factor I; similar with CFAI_RAT
Px011888.1	1.1647	3.48E-08	1.38E-07	Chymotrypsin-like elastase family member 2B
Px014830.1	1.1646	9.91E-167	2.71E-165	Tryptase; similar with TRYB1_MOUSE
Px003670.2	1.1636	0.00E+00	0.00E+00	Serine protease easter; similar with EAST_DROME
Px004131.5	1.1474	6.20E-26	4.92E-25	Calcium-independent phospholipase A2-gamma
Px010855.1	1.1350	3.54E-11	1.72E-10	Trypsin, alkaline A; similar with TRYA_MANSE
Px012098.2	1.0475	2.15E-05	6.71E-05	Lipase member I; similar with LIPI_HUMAN
Px011757.1	1.0401	5.69E-11	2.72E-10	Esterase FE4; similar with ESTF_MYZPE
Px016983.3	1.0379	6.64E-09	2.78E-08	Serine proteinase stubble; similar with STUB_DROME
Px003126.1	-3.3822	3.42E-05	1.05E-04	Adenylate cyclase type 3; similar with ADCY3_MOUSE
Px014636.1	-1.5417	2.28E-04	6.26E-04	Trypsin-1; similar with TRY1_ANOGA
Px016332.1	-1.5166	0.00E+00	0.00E+00	Calcium-transporting ATPase sarcoplasmic/endoplasmic reticulum
Px000220.2	-1.1220	7.78E-07	2.79E-06	Bumetanide-sensitive sodium-(potassium)-chloride cotransporter
Px012289.1	-1.0288	3.16E-06	1.07E-05	Liver carboxylesterase; similar with EST1_MESAU

Involved in protein digestion and absorption

Px007620.1	10.3950	8.15E-10	3.63E-09	Trypsin CFT-1; similar with TRYP_CHOFU
Px006069.1	10.0136	2.26E-06	7.79E-06	Trypsin CFT-1; similar with TRYP_CHOFU
Px013802.1	8.5527	1.46E-08	5.93E-08	Sodium/potassium-transporting ATPase subunit alpha-2
Px008830.1	6.6433	1.43E-111	2.90E-110	Trypsin, alkaline C; similar with TRYC_MANSE
Px001804.1	6.5119	2.83E-51	3.39E-50	Trypsin, alkaline B; similar with TRYB_MANSE
Px007670.1	6.2472	5.75E-63	7.92E-62	Proclotting enzyme; similar with PCE_TACTR
Px007357.2	5.3379	0.00E+00	0.00E+00	Serine proteinase stubble; similar with STUB_DROME
Px010971.1	5.2472	1.05E-10	4.92E-10	Trypsin CFT-1; similar with TRYP_CHOFU
Px005240.1	5.1310	8.24E-124	1.79E-122	Trypsin, alkaline A; similar with TRYA_MANSE
Px014256.1	4.9352	1.34E-08	5.46E-08	Unknown functio
Px014882.1	4.8506	4.25E-162	1.13E-160	Trypsin, alkaline C; similar with TRYC_MANSE
Px005554.1	4.6008	8.20E-07	2.93E-06	Chymotrypsin-1; similar with CTR1_ANOGA
Px016055.1	4.5497	4.52E-56	5.70E-55	Trypsin, alkaline C; similar with TRYC_MANSE
Px002864.1	4.5298	1.55E-103	3.01E-102	Trypsin, alkaline B; similar with TRYB_MANSE
Px000995.2	4.3915	0.00E+00	0.00E+00	Carboxypeptidase A4; similar with CBPA4_HUMAN
Px005241.1	4.3794	1.43E-53	1.74E-52	Trypsin CFT-1; similar with TRYP_CHOFU
Px007598.1	4.3759	1.54E-119	3.27E-118	Chymotrypsin-1; similar with CTR1_ANOGA

Px016053.1	4.2124	5.01E-59	6.53E-58	Trypsin; similar with TRYP_PHACE
Px009637.1	4.1028	1.35E-27	1.13E-26	Trypsin CFT-1; similar with TRYP_CHOFU
Px002586.1	4.0373	5.30E-58	6.84E-57	Trypsin CFT-1; similar with TRYP_CHOFU
Px013377.1	4.0330	2.06E-219	6.86E-218	Trypsin; similar with TRYP_PHACE
Px015276.1	4.0315	9.55E-65	1.33E-63	Trypsin CFT-1; similar with TRYP_CHOFU
Px005243.1	3.9876	0.00E+00	0.00E+00	Trypsin, alkaline A; similar with TRYA_MANSE
Px015277.1	3.9698	7.98E-158	2.09E-156	Trypsin CFT-1; similar with TRYP_CHOFU
Px010313.2	3.7896	2.43E-106	4.78E-105	Serine proteinase stubble; similar with STUB_DROME
Px006571.1	3.7885	0.00E+00	0.00E+00	Trypsin, alkaline B; similar with TRYB_MANSE
Px012813.1	3.6622	2.98E-06	1.02E-05	Serine proteinase stubble; similar with STUB_DROME
Px001649.1	3.6353	5.51E-68	7.90E-67	MAM domain-containing glycosylphosphatidylinositol anchor protein 1
Px005239.1	3.6181	0.00E+00	0.00E+00	Trypsin, alkaline A; similar with TRYA_MANSE
Px014201.1	3.6008	7.33E-11	3.48E-10	Sodium/potassium-transporting ATPase subunit alpha-2
Px000992.1	3.6006	0.00E+00	0.00E+00	Carboxypeptidase A1; similar with CBPA1_BOVIN
Px007617.1	3.5958	0.00E+00	0.00E+00	Trypsin CFT-1; similar with TRYP_CHOFU
Px006572.1	3.4867	0.00E+00	0.00E+00	Trypsin; similar with TRYP_PHACE
Px005765.1	3.4325	1.33E-62	1.82E-61	Vitellin-degrading protease; similar with VDP_BOMMO
Px016056.1	3.4297	0.00E+00	0.00E+00	Trypsin, alkaline A; similar with TRYA_MANSE
Px008538.1	3.4202	3.15E-138	7.39E-137	Trypsin, alkaline B; similar with TRYB_MANSE
Px011428.1	3.4151	1.82E-19	1.23E-18	Peptide transporter family 1; similar with PEPT1_DROME
Px012048.1	3.3901	4.29E-64	5.96E-63	Chymotrypsin BI; similar with CTRB1_LITVA
Px007621.1	3.3847	6.61E-21	4.65E-20	Trypsin CFT-1; similar with TRYP_CHOFU
Px014592.1	3.3707	9.04E-90	1.58E-88	Trypsin CFT-1; similar with TRYP_CHOFU
Px005238.1	3.3700	4.57E-59	5.96E-58	Trypsin, alkaline B; similar with TRYB_MANSE
Px009569.1	3.3179	0.00E+00	0.00E+00	Trypsin CFT-1; similar with TRYP_CHOFU
Px007901.1	3.2912	0.00E+00	0.00E+00	Chymotrypsin BI; similar with CTRB1_LITVA
Px001998.2	3.2879	2.48E-118	5.21E-117	Carboxypeptidase B; similar with CBPB_ASTFL
Px013646.1	3.2808	1.40E-59	1.84E-58	Proton-coupled amino acid transporter 4
Px000993.1	3.2755	0.00E+00	0.00E+00	Carboxypeptidase A2; similar with CBPA2_RAT
Px000991.6	3.2679	0.00E+00	0.00E+00	Carboxypeptidase O; similar with CBPO_BOVIN
Px007599.1	3.2567	0.00E+00	0.00E+00	Chymotrypsin-1; similar with CTR1_ANOGA
Px006767.1	3.2472	1.38E-04	3.92E-04	Chymotrypsin-1; similar with E2BNV4_9HYME
Px015279.1	3.2472	1.79E-18	1.17E-17	Trypsin, alkaline A; similar with TRYA_MANSE
Px004168.1	3.2472	2.59E-13	1.38E-12	Plasma kallikrein; similar with KLKB1_HUMAN
Px002648.1	3.2472	2.32E-06	7.97E-06	Tachykinins; similar with TACHY_APIME
Px012568.1	3.2398	0.00E+00	0.00E+00	Trypsin CFT-1; similar with TRYP_CHOFU
Px007900.1	3.2235	0.00E+00	0.00E+00	Chymotrypsin BI; similar with CTRB1_LITVA
Px015278.1	3.1962	2.10E-27	1.75E-26	Trypsin, alkaline A; similar with TRYA_MANSE
Px010386.1	3.1714	0.00E+00	0.00E+00	Trypsin CFT-1; similar with TRYP_CHOFU
Px016054.1	3.1536	3.24E-37	3.20E-36	Trypsin; similar with TRYP_PHACE
Px007619.2	3.0951	0.00E+00	0.00E+00	Trypsin, alkaline A; similar with TRYA_MANSE
Px002861.1	3.0431	0.00E+00	0.00E+00	Trypsin, alkaline C; similar with TRYC_MANSE
Px009639.1	3.0376	0.00E+00	0.00E+00	Trypsin CFT-1; similar with TRYP_CHOFU
Px001997.1	3.0350	2.53E-37	2.50E-36	Carboxypeptidase B; similar with CBPB_ASTFL
Px007903.1	2.9954	0.00E+00	0.00E+00	Collagenase; similar with COGS_HYPLI
Px014424.1	2.9841	1.63E-06	5.68E-06	Proton-coupled amino acid transporter 4
Px005244.1	2.9796	7.91E-29	6.74E-28	Trypsin CFT-1; similar with TRYP_CHOFU
Px015049.1	2.9693	5.52E-35	5.22E-34	Venom dipeptidyl peptidase 4; similar with VDDP4_VESVU
Px015280.1	2.9138	7.05E-10	3.15E-09	Trypsin CFT-1; similar with TRYP_CHOFU
Px006570.1	2.8972	0.00E+00	0.00E+00	Trypsin, alkaline B; similar with TRYB_MANSE
Px000996.2	2.8881	3.06E-71	4.53E-70	Carboxypeptidase A2; similar with CBPA2_MOUSE
Px000994.1	2.8631	0.00E+00	0.00E+00	Zinc carboxypeptidase A 1; similar with CBPA1_DROPS
Px016057.1	2.8626	2.00E-77	3.18E-76	Trypsin, alkaline B; similar with TRYB_MANSE
Px009638.1	2.8523	0.00E+00	0.00E+00	Trypsin, alkaline B; similar with TRYB_MANSE

Px002587.1	2.7903	0.00E+00	0.00E+00	Trypsin CFT-1; similar with TRYP_CHOFU
Px002993.1	2.7717	0.00E+00	0.00E+00	Zinc metalloproteinase nas-4; similar with NAS4_CAEEL
Px005373.1	2.7565	1.52E-39	1.56E-38	Carboxypeptidase B; similar with CBPB_ASTFL
Px000107.1	2.7335	0.00E+00	0.00E+00	Trypsin, alkaline C; similar with TRYC_MANSE
Px007618.1	2.7222	1.13E-272	4.41E-271	Trypsin CFT-1; similar with TRYP_CHOFU
Px011887.1	2.7196	0.00E+00	0.00E+00	Trypsin, alkaline C; similar with TRYC_MANSE
Px009286.1	2.7141	0.00E+00	0.00E+00	Trypsin, alkaline C; similar with TRYC_MANSE
Px005936.2	2.7085	3.12E-86	5.29E-85	Trypsin, alkaline C; similar with TRYC_MANSE
Px002442.1	2.6772	1.51E-16	9.17E-16	Trypsin, alkaline C; similar with TRYC_MANSE
Px002865.1	2.6726	0.00E+00	0.00E+00	Trypsin 5G1; similar with TRY5_AEDAE
Px007902.1	2.6449	5.66E-27	4.65E-26	Collagenase; similar with COGS_HYPLI
Px004330.1	2.6327	2.73E-97	5.00E-96	Proton-coupled amino acid transporter 1
Px002441.1	2.6127	4.36E-75	6.75E-74	Trypsin, alkaline B; similar with TRYB_MANSE
Px018074.1	2.6056	3.91E-24	2.97E-23	Chymotrypsin-like elastase family member 2A
Px011889.1	2.5691	1.52E-21	1.09E-20	Achelase-1; similar with ACH1_LONAC
Px007600.1	2.5619	1.13E-226	3.92E-225	Chymotrypsin-1; similar with CTR1_ANOGA
Px007674.2	2.5239	0.00E+00	0.00E+00	Collagenase; similar with COGS_HYPLI
Px007675.1	2.5233	0.00E+00	0.00E+00	Collagenase; similar with COGS_HYPLI
Px002860.1	2.5102	4.25E-17	2.64E-16	Achelase-1; similar with ACH1_LONAC
Px009766.1	2.4923	6.69E-06	2.20E-05	Lysosomal Pro-X carboxypeptidase; similar with PCP_PONAB
Px007677.1	2.4787	0.00E+00	0.00E+00	Trypsin-7; similar with TRY7_ANOGA
Px012582.1	2.4724	2.13E-127	4.68E-126	Collagenase; similar with COGS_HYPLI
Px009488.1	2.4558	6.08E-05	1.81E-04	Chymotrypsin-like serine proteinase; similar with CTRL_HALRU
Px016058.1	2.4185	0.00E+00	0.00E+00	Trypsin, alkaline C; similar with TRYC_MANSE
Px002588.1	2.4151	4.81E-14	2.66E-13	Trypsin, alkaline C; similar with TRYC_MANSE
Px010489.1	2.4085	6.89E-66	9.73E-65	Trypsin, alkaline C; similar with TRYC_MANSE
Px009287.1	2.4027	1.27E-279	5.09E-278	Trypsin CFT-1; similar with TRYP_CHOFU
Px009395.1	2.3871	1.03E-17	6.47E-17	Proclotting enzyme; similar with PCE_TACTR
Px013212.4	2.3763	4.42E-145	1.09E-143	Inactive dipeptidyl peptidase 10; similar with DPP10_HUMAN
Px007616.1	2.3746	1.09E-158	2.87E-157	Trypsin CFT-1; similar with TRYP_CHOFU
Px005242.1	2.3668	2.59E-19	1.73E-18	Trypsin CFT-1; similar with TRYP_CHOFU
Px002589.1	2.3435	3.45E-96	6.29E-95	Trypsin, alkaline A; similar with TRYA_MANSE
Px015275.1	2.3286	0.00E+00	0.00E+00	Trypsin CFT-1; similar with TRYP_CHOFU
Px007601.1	2.2954	3.63E-31	3.23E-30	Chymotrypsin-1; similar with CTR1_ANOGA
Px017515.1	2.2558	2.50E-108	4.98E-107	Trypsin, alkaline C; similar with TRYC_MANSE
Px009567.1	2.2552	3.00E-104	5.83E-103	Trypsin, alkaline B; similar with TRYB_MANSE
Px001990.2	2.2289	2.92E-104	5.69E-103	Carboxypeptidase B; similar with CBPB_ASTFL
Px009566.1	2.2251	1.29E-259	4.84E-258	Trypsin, alkaline C; similar with TRYC_MANSE
Px000771.1	2.1857	1.04E-117	2.18E-116	Trypsin CFT-1; similar with TRYP_CHOFU
Px001878.1	2.1781	2.23E-21	1.59E-20	Lysosomal Pro-X carboxypeptidase; similar with PCP_PONAB
Px011886.1	2.1750	1.89E-125	4.14E-124	Trypsin CFT-1; similar with TRYP_CHOFU
Px001239.1	2.1600	2.79E-38	2.80E-37	Serine protease P162; similar with D2A5Y8_TRICA
Px001833.1	2.0603	0.00E+00	0.00E+00	Serine protease easter; similar with EAST_DROME
Px004167.1	2.0469	1.15E-06	4.06E-06	Plasma kallikrein; similar with KLKB1_MOUSE
Px012569.1	2.0285	0.00E+00	0.00E+00	Trypsin, alkaline C; similar with TRYC_MANSE
Px002440.1	2.0145	2.30E-21	1.64E-20	Trypsin, alkaline B; similar with TRYB_MANSE
Px012570.1	1.9654	0.00E+00	0.00E+00	Trypsin CFT-1; similar with TRYP_CHOFU
Px011097.1	1.9460	2.65E-10	1.22E-09	Serine proteinase stubble; similar with STUB_DROME
Px002512.1	1.9231	4.29E-29	3.68E-28	Brachyurin; similar with COGS_UCAPU
Px010975.1	1.8944	5.90E-09	2.48E-08	Trypsin, alkaline C; similar with TRYC_MANSE
Px002866.1	1.8658	5.28E-09	2.23E-08	Trypsin, alkaline A; similar with TRYA_MANSE
Px008406.1	1.8639	2.77E-08	1.10E-07	Serine proteinase stubble; similar with STUB_DROME
Px013665.3	1.8475	2.46E-37	2.43E-36	Collagenase; similar with COGS_HYPLI
Px012329.1	1.7693	5.39E-84	8.95E-83	Serine protease persephone; similar with PSH_DROME

Px011231.1	1.7453	1.76E-27	1.46E-26	Protein unc-50 homolog; similar with UNC50_DROME
Px010017.1	1.7418	0.00E+00	0.00E+00	Carboxypeptidase B; similar with CBPB_ASTFL
Px000402.2	1.6821	5.77E-83	9.50E-82	Serine protease persephone; similar with PSH_DROME
Px009088.1	1.6795	5.48E-174	1.55E-172	Mastin; similar with TRYM_CANFA
Px005342.1	1.6408	3.80E-156	9.82E-155	Collagenase; similar with COGS_HYPLI
Px007904.1	1.6124	0.00E+00	0.00E+00	Collagenase; similar with COGS_HYPLI
Px001556.1	1.6061	8.94E-117	1.85E-115	Serine protease easter; similar with EAST_DROME
Px013162.1	1.5740	7.41E-151	1.88E-149	Serine protease snake; similar with SNAK_DROME
Px011754.1	1.5564	1.54E-08	6.25E-08	Proton-coupled amino acid transporter 1
Px005340.1	1.5499	1.24E-139	2.94E-138	Collagenase; similar with COGS_HYPLI
Px005328.1	1.5414	2.32E-48	2.68E-47	Tubulin-specific chaperone cofactor E-like protein
Px002809.2	1.5031	7.52E-18	4.78E-17	WD repeat domain phosphoinositide-interacting protein 4
Px012067.1	1.4726	4.54E-10	2.06E-09	Proton-coupled amino acid transporter 4; similar with S36A4_XENLA
Px014831.2	1.4467	0.00E+00	0.00E+00	Serine proteinase stubble; similar with STUB_DROME
Px000703.2	1.4219	2.78E-12	1.41E-11	Y+L amino acid transporter 2; similar with YLAT2_MOUSE
Px010627.1	1.4167	1.53E-153	3.89E-152	Wing disc-specific protein; similar with Q8WPH2_BOMMO
Px007681.2	1.4129	6.79E-05	2.01E-04	Monocarboxylate transporter 2; similar with MOT2_RAT
Px012191.1	1.4028	2.38E-208	7.61E-207	Sodium/potassium-transporting ATPase subunit alpha-1
Px009568.1	1.3992	1.23E-04	3.53E-04	Trypsin, alkaline C; similar with TRYC_MANSE
Px005341.1	1.3783	1.92E-45	2.13E-44	Collagenase; similar with COGS_HYPLI
Px005947.1	1.3627	2.45E-06	8.39E-06	Serine proteinase stubble; similar with STUB_DROME
Px011737.1	1.3544	0.00E+00	0.00E+00	Neurofilament heavy polypeptide; similar with NFH_MOUSE
Px007906.2	1.3236	0.00E+00	0.00E+00	Collagenase; similar with COGS_HYPLI
Px008882.1	1.2542	1.19E-128	2.65E-127	Unknown functio
Px011499.2	1.1964	1.89E-18	1.23E-17	Serine protease persephone; similar with PSH_DROME
Px011153.3	1.1683	1.07E-26	8.76E-26	Complement factor I; similar with CFAI_RAT
Px011888.1	1.1647	3.48E-08	1.38E-07	Chymotrypsin-like elastase family member 2B
Px014830.1	1.1646	9.91E-167	2.71E-165	Tryptase; similar with TRYB1_MOUSE
Px003670.2	1.1636	0.00E+00	0.00E+00	Serine protease easter; similar with EAST_DROME
Px013919.1	1.1490	0.00E+00	0.00E+00	Collagen alpha-4(VI) chain; similar with CO6A4_MOUSE
Px010855.1	1.1350	3.54E-11	1.72E-10	Trypsin, alkaline A; similar with TRYA_MANSE
Px002792.1	1.0827	4.51E-25	3.50E-24	Unknown functio
Px008960.1	1.0459	7.21E-30	6.29E-29	Peptide transporter family 1; similar with PEPT1_DROME
Px016983.3	1.0379	6.64E-09	2.78E-08	Serine proteinase stubble; similar with STUB_DROME
Px005604.1	1.0184	4.78E-11	2.29E-10	Monocarboxylate transporter 10; similar with MOT10_MOUSE
Px011876.1	-8.7166	3.46E-04	9.33E-04	Armadillo repeat-containing protein 2; similar with ARMC2_HUMAN
Px015142.1	-1.8073	1.28E-05	4.08E-05	Proton-coupled amino acid transporter 1; similar with S36A1_HUMAN
Px014636.1	-1.5417	2.28E-04	6.26E-04	Trypsin-1; similar with TRY1_ANOGA
Px005208.1	-1.3118	2.13E-04	5.89E-04	Inactive dipeptidyl peptidase 10; similar with DPP10_HUMAN
Px012129.1	-1.2949	1.78E-33	1.64E-32	Lysosomal Pro-X carboxypeptidase; similar with PCP_PONAB
Px009791.1	-1.1980	0.00E+00	0.00E+00	Cuticle collagen bli-1; similar with BLI1_CAEEL

Involved in Influenza A

Px008374.1	10.7904	2.99E-08	1.19E-07	Phosphatidylinositol-4,5-bisphosphate 3-kinase catalytic subunit delta isoform
Px007620.1	10.3950	8.15E-10	3.63E-09	Trypsin CFT-1; similar with TRYP_CHOFU
Px006069.1	10.0136	2.26E-06	7.79E-06	Trypsin CFT-1; similar with TRYP_CHOFU
Px008830.1	6.6433	1.43E-111	2.90E-110	Trypsin, alkaline C; similar with TRYC_MANSE
Px001804.1	6.5119	2.83E-51	3.39E-50	Trypsin, alkaline B; similar with TRYB_MANSE
Px007670.1	6.2472	5.75E-63	7.92E-62	Proclotting enzyme; similar with PCE_TACTR
Px007357.2	5.3379	0.00E+00	0.00E+00	Serine proteinase stubble; similar with STUB_DROME
Px010971.1	5.2472	1.05E-10	4.92E-10	Trypsin CFT-1; similar with TRYP_CHOFU
Px005240.1	5.1310	8.24E-124	1.79E-122	Trypsin, alkaline A; similar with TRYA_MANSE
Px014882.1	4.8506	4.25E-162	1.13E-160	Trypsin, alkaline C; similar with TRYC_MANSE
Px005554.1	4.6008	8.20E-07	2.93E-06	Chymotrypsin-1; similar with CTR1_ANOGA

Px016055.1	4.5497	4.52E-56	5.70E-55	Trypsin, alkaline C; similar with TRYC_MANSE
Px002864.1	4.5298	1.55E-103	3.01E-102	Trypsin, alkaline B; similar with TRYB_MANSE
Px005241.1	4.3794	1.43E-53	1.74E-52	Trypsin CFT-1; similar with TRYP_CHOFU
Px007598.1	4.3759	1.54E-119	3.27E-118	Chymotrypsin-1; similar with CTR1_ANOGA
Px015514.1	4.2671	1.44E-18	9.38E-18	Serine/threonine-protein kinase pelle; similar with KPEL_DROME
Px016053.1	4.2124	5.01E-59	6.53E-58	Trypsin; similar with TRYP_PHACE
Px009637.1	4.1028	1.35E-27	1.13E-26	Trypsin CFT-1; similar with TRYP_CHOFU
Px002586.1	4.0373	5.30E-58	6.84E-57	Trypsin CFT-1; similar with TRYP_CHOFU
Px013377.1	4.0330	2.06E-219	6.86E-218	Trypsin; similar with TRYP_PHACE
Px015276.1	4.0315	9.55E-65	1.33E-63	Trypsin CFT-1; similar with TRYP_CHOFU
Px005243.1	3.9876	0.00E+00	0.00E+00	Trypsin, alkaline A; similar with TRYA_MANSE
Px015277.1	3.9698	7.98E-158	2.09E-156	Trypsin CFT-1; similar with TRYP_CHOFU
Px010313.2	3.7896	2.43E-106	4.78E-105	Serine proteinase stubble; similar with STUB_DROME
Px006571.1	3.7885	0.00E+00	0.00E+00	Trypsin, alkaline B; similar with TRYB_MANSE
Px004048.1	3.7053	2.38E-30	2.09E-29	Toll-like receptor 6; similar with TLR6_BOVIN
Px012813.1	3.6622	2.98E-06	1.02E-05	Serine proteinase stubble; similar with STUB_DROME
Px005239.1	3.6181	0.00E+00	0.00E+00	Trypsin, alkaline A; similar with TRYA_MANSE
Px007617.1	3.5958	0.00E+00	0.00E+00	Trypsin CFT-1; similar with TRYP_CHOFU
Px006572.1	3.4867	0.00E+00	0.00E+00	Trypsin; similar with TRYP_PHACE
Px005765.1	3.4325	1.33E-62	1.82E-61	Vitellin-degrading protease; similar with VDP_BOMMO
Px016056.1	3.4297	0.00E+00	0.00E+00	Trypsin, alkaline A; similar with TRYA_MANSE
Px008538.1	3.4202	3.15E-138	7.39E-137	Trypsin, alkaline B; similar with TRYB_MANSE
Px007621.1	3.3847	6.61E-21	4.65E-20	Trypsin CFT-1; similar with TRYP_CHOFU
Px014592.1	3.3707	9.04E-90	1.58E-88	Trypsin CFT-1; similar with TRYP_CHOFU
Px005238.1	3.3700	4.57E-59	5.96E-58	Trypsin, alkaline B; similar with TRYB_MANSE
Px009569.1	3.3179	0.00E+00	0.00E+00	Trypsin CFT-1; similar with TRYP_CHOFU
Px007901.1	3.2912	0.00E+00	0.00E+00	Chymotrypsin BI; similar with CTRB1_LITVA
Px007599.1	3.2567	0.00E+00	0.00E+00	Chymotrypsin-1; similar with CTR1_ANOGA
Px006767.1	3.2472	1.38E-04	3.92E-04	Chymotrypsin-1; similar with E2BNV4_9HYME
Px015279.1	3.2472	1.79E-18	1.17E-17	Trypsin, alkaline A; similar with TRYA_MANSE
Px012568.1	3.2398	0.00E+00	0.00E+00	Trypsin CFT-1; similar with TRYP_CHOFU
Px015278.1	3.1962	2.10E-27	1.75E-26	Trypsin, alkaline A; similar with TRYA_MANSE
Px010386.1	3.1714	0.00E+00	0.00E+00	Trypsin CFT-1; similar with TRYP_CHOFU
Px016054.1	3.1536	3.24E-37	3.20E-36	Trypsin; similar with TRYP_PHACE
Px007619.2	3.0951	0.00E+00	0.00E+00	Trypsin, alkaline A; similar with TRYA_MANSE
Px002861.1	3.0431	0.00E+00	0.00E+00	Trypsin, alkaline C; similar with TRYC_MANSE
Px009639.1	3.0376	0.00E+00	0.00E+00	Trypsin CFT-1; similar with TRYP_CHOFU
Px002457.1	2.9802	1.14E-158	2.99E-157	Protein dpy-19 homolog 1; similar with D19L1_HUMAN
Px005244.1	2.9796	7.91E-29	6.74E-28	Trypsin CFT-1; similar with TRYP_CHOFU
Px015280.1	2.9138	7.05E-10	3.15E-09	Trypsin CFT-1; similar with TRYP_CHOFU
Px006570.1	2.8972	0.00E+00	0.00E+00	Trypsin, alkaline B; similar with TRYB_MANSE
Px004108.2	2.8846	2.77E-66	3.92E-65	Heat shock protein 68; similar with HSP68_DROME
Px008604.1	2.8677	2.19E-157	5.71E-156	AGAP011476-PA; similar with Q7Q343_ANOGA
Px016057.1	2.8626	2.00E-77	3.18E-76	Trypsin, alkaline B; similar with TRYB_MANSE
Px009638.1	2.8523	0.00E+00	0.00E+00	Trypsin, alkaline B; similar with TRYB_MANSE
Px002587.1	2.7903	0.00E+00	0.00E+00	Trypsin CFT-1; similar with TRYP_CHOFU
Px000107.1	2.7335	0.00E+00	0.00E+00	Trypsin, alkaline C; similar with TRYC_MANSE
Px007618.1	2.7222	1.13E-272	4.41E-271	Trypsin CFT-1; similar with TRYP_CHOFU
Px011887.1	2.7196	0.00E+00	0.00E+00	Trypsin, alkaline C; similar with TRYC_MANSE
Px009286.1	2.7141	0.00E+00	0.00E+00	Trypsin, alkaline C; similar with TRYC_MANSE
Px005936.2	2.7085	3.12E-86	5.29E-85	Trypsin, alkaline C; similar with TRYC_MANSE
Px002442.1	2.6772	1.51E-16	9.17E-16	Trypsin, alkaline C; similar with TRYC_MANSE
Px002865.1	2.6726	0.00E+00	0.00E+00	Trypsin 5G1; similar with TRY5_AEDAE
Px002441.1	2.6127	4.36E-75	6.75E-74	Trypsin, alkaline B; similar with TRYB_MANSE

Px011889.1	2.5691	1.52E-21	1.09E-20	Achelase-1; similar with ACH1_LONAC
Px007600.1	2.5619	1.13E-226	3.92E-225	Chymotrypsin-1; similar with CTR1_ANOGA
Px007675.1	2.5233	0.00E+00	0.00E+00	Collagenase; similar with COGS_HYPLI
Px011248.1	2.5102	3.46E-05	1.06E-04	Radial spoke head protein 4 homolog A; similar with RSH4A_HUMAN
Px002860.1	2.5102	4.25E-17	2.64E-16	Achelase-1; similar with ACH1_LONAC
Px007677.1	2.4787	0.00E+00	0.00E+00	Trypsin-7; similar with TRY7_ANOGA
Px012582.1	2.4724	2.13E-127	4.68E-126	Collagenase; similar with COGS_HYPLI
Px007552.1	2.4696	1.06E-167	2.91E-166	Paramyosin, putative; similar with E0VGP7_PEDHC
Px009488.1	2.4558	6.08E-05	1.81E-04	Chymotrypsin-like serine proteinase; similar with CTRL_HALRU
Px016058.1	2.4185	0.00E+00	0.00E+00	Trypsin, alkaline C; similar with TRYC_MANSE
Px002588.1	2.4151	4.81E-14	2.66E-13	Trypsin, alkaline C; similar with TRYC_MANSE
Px010489.1	2.4085	6.89E-66	9.73E-65	Trypsin, alkaline C; similar with TRYC_MANSE
Px009287.1	2.4027	1.27E-279	5.09E-278	Trypsin CFT-1; similar with TRYP_CHOFU
Px009395.1	2.3871	1.03E-17	6.47E-17	Proclotting enzyme; similar with PCE_TACTR
Px007616.1	2.3746	1.09E-158	2.87E-157	Trypsin CFT-1; similar with TRYP_CHOFU
Px005242.1	2.3668	2.59E-19	1.73E-18	Trypsin CFT-1; similar with TRYP_CHOFU
Px002589.1	2.3435	3.45E-96	6.29E-95	Trypsin, alkaline A; similar with TRYA_MANSE
Px015275.1	2.3286	0.00E+00	0.00E+00	Trypsin CFT-1; similar with TRYP_CHOFU
Px010499.1	2.3073	3.86E-59	5.04E-58	Serine/threonine-protein kinase pelle; similar with KPEL_DROME
Px007601.1	2.2954	3.63E-31	3.23E-30	Chymotrypsin-1; similar with CTR1_ANOGA
Px013205.1	2.2558	4.92E-55	6.10E-54	Putative uncharacterized protein; similar with D6WHY1_TRICA
Px017515.1	2.2558	2.50E-108	4.98E-107	Trypsin, alkaline C; similar with TRYC_MANSE
Px009567.1	2.2552	3.00E-104	5.83E-103	Trypsin, alkaline B; similar with TRYB_MANSE
Px009566.1	2.2251	1.29E-259	4.84E-258	Trypsin, alkaline C; similar with TRYC_MANSE
Px000771.1	2.1857	1.04E-117	2.18E-116	Trypsin CFT-1; similar with TRYP_CHOFU
Px011886.1	2.1750	1.89E-125	4.14E-124	Trypsin CFT-1; similar with TRYP_CHOFU
Px001239.1	2.1600	2.79E-38	2.80E-37	Serine protease P162; similar with D2A5Y8_TRICA
Px001833.1	2.0603	0.00E+00	0.00E+00	Serine protease easter; similar with EAST_DROME
Px004167.1	2.0469	1.15E-06	4.06E-06	Plasma kallikrein; similar with KLKB1_MOUSE
Px012569.1	2.0285	0.00E+00	0.00E+00	Trypsin, alkaline C; similar with TRYC_MANSE
Px009870.1	2.0232	0.00E+00	0.00E+00	Inter-alpha-trypsin inhibitor heavy chain H5-like protein
Px002440.1	2.0145	2.30E-21	1.64E-20	Trypsin, alkaline B; similar with TRYB_MANSE
Px006985.1	1.9682	0.00E+00	0.00E+00	Putative uncharacterized protein; similar with B1NLD7_HELAM
Px012570.1	1.9654	0.00E+00	0.00E+00	Trypsin CFT-1; similar with TRYP_CHOFU
Px003725.1	1.9489	6.60E-74	1.01E-72	Interleukin-1 receptor-associated kinase 4; similar with IRAK4_BOVIN
Px011097.1	1.9460	2.65E-10	1.22E-09	Serine proteinase stubble; similar with STUB_DROME
Px007201.5	1.9364	7.77E-18	4.93E-17	Tyrosine recombinase; similar with Q68SM2_TRICA
Px008682.1	1.9277	7.20E-14	3.94E-13	Coagulation factor XI; similar with FA11_HUMAN
Px010975.1	1.8944	5.90E-09	2.48E-08	Trypsin, alkaline C; similar with TRYC_MANSE
Px002866.1	1.8658	5.28E-09	2.23E-08	Trypsin, alkaline A; similar with TRYA_MANSE
Px008406.1	1.8639	2.77E-08	1.10E-07	Serine proteinase stubble; similar with STUB_DROME
Px013665.3	1.8475	2.46E-37	2.43E-36	Collagenase; similar with COGS_HYPLI
Px012329.1	1.7693	5.39E-84	8.95E-83	Serine protease persephone; similar with PSH_DROME
Px015180.1	1.7466	2.09E-49	2.45E-48	ADP,ATP carrier protein; similar with ADT_DROME
Px011231.1	1.7453	1.76E-27	1.46E-26	Protein unc-50 homolog; similar with UNC50_DROME
Px000402.2	1.6821	5.77E-83	9.50E-82	Serine protease persephone; similar with PSH_DROME
Px004977.1	1.6799	1.55E-260	5.85E-259	Transcription factor AP-1; similar with JRA_DROME
Px009088.1	1.6795	5.48E-174	1.55E-172	Mastin; similar with TRYM_CANFA
Px013101.1	1.6622	3.21E-06	1.09E-05	Nose resistant to fluoxetine protein 6; similar with NRF6_CAEEL
Px005342.1	1.6408	3.80E-156	9.82E-155	Collagenase; similar with COGS_HYPLI
Px001556.1	1.6061	8.94E-117	1.85E-115	Serine protease easter; similar with EAST_DROME
Px005490.1	1.5819	5.31E-62	7.19E-61	Inhibitor of nuclear factor kappa-B kinase subunit beta
Px005960.1	1.5748	5.55E-09	2.34E-08	Putative uncharacterized protein; similar with D6W9B6_TRICA
Px013162.1	1.5740	7.41E-151	1.88E-149	Serine protease snake; similar with SNAK_DROME

Px005340.1	1.5499	1.24E-139	2.94E-138	Collagenase; similar with COGS_HYPLI
Px014831.2	1.4467	0.00E+00	0.00E+00	Serine proteinase stubble; similar with STUB_DROME
Px009011.1	1.4129	6.79E-05	2.01E-04	Tyrosine-protein kinase hopscotch; similar with JAK_DROME
Px009568.1	1.3992	1.23E-04	3.53E-04	Trypsin, alkaline C; similar with TRYC_MANSE
Px005341.1	1.3783	1.92E-45	2.13E-44	Collagenase; similar with COGS_HYPLI
Px004105.1	1.3645	1.30E-23	9.72E-23	Heat shock protein 68; similar with HSP68_DROME
Px005947.1	1.3627	2.45E-06	8.39E-06	Serine proteinase stubble; similar with STUB_DROME
Px015363.1	1.2365	5.17E-05	1.55E-04	Slit homolog 3 protein; similar with SLIT3_MOUSE
Px005175.1	1.2331	1.71E-19	1.16E-18	Uncharacterized protein KIAA1109; similar with K1109_MOUSE
Px011499.2	1.1964	1.89E-18	1.23E-17	Serine protease persephone; similar with PSH_DROME
Px017224.1	1.1915	1.34E-11	6.59E-11	Chaoptin; similar with CHAO_DROME
Px014830.1	1.1646	9.91E-167	2.71E-165	Tryptase; similar with TRYB1_MOUSE
Px003670.2	1.1636	0.00E+00	0.00E+00	Serine protease easter; similar with EAST_DROME
Px010855.1	1.1350	3.54E-11	1.72E-10	Trypsin, alkaline A; similar with TRYA_MANSE
Px011525.1	1.0996	2.63E-07	9.80E-07	Programmed cell death protein 4; similar with PDCD4_CHICK
Px006234.1	1.0631	1.55E-14	8.69E-14	Chaoptin; similar with CHAO_DROME
Px008341.1	1.0613	2.09E-24	1.59E-23	Mitochondrial fission 1 protein; similar with FIS1_HUMAN
Px001401.1	1.0591	1.02E-04	2.94E-04	Apolipophorins; similar with APLP_MANSE
Px016983.3	1.0379	6.64E-09	2.78E-08	Serine proteinase stubble; similar with STUB_DROME
Px001873.1	-2.5077	3.78E-06	1.28E-05	Probable palmitoyltransferase ZDHHC8
Px009322.1	-1.8126	2.19E-185	6.48E-184	Unknown functio
Px014636.1	-1.5417	2.28E-04	6.26E-04	Trypsin-1; similar with TRY1_ANOGA
Px013456.1	-1.4780	9.11E-21	6.38E-20	Actin, clone 211; similar with ACT2_ARTSX
Px003076.1	-1.3663	2.09E-05	6.52E-05	Chaoptin; similar with CHAO_DROME
Px003840.1	-1.3269	0.00E+00	0.00E+00	Actin, muscle; similar with ACT_MANSE
Px014679.1	-1.2335	5.49E-08	2.14E-07	Neuromedin-B receptor; similar with NMBR_MOUSE
Px009400.1	-1.1378	3.13E-04	8.48E-04	Protein IWS1 homolog; similar with IWS1_RAT
Px012579.1	-1.0801	1.12E-29	9.70E-29	mRNA export factor; similar with RAEL1_PIG
Px003808.1	-1.0231	0.00E+00	0.00E+00	Actin, muscle; similar with ACT_MANSE
Px006532.1	-1.0165	1.80E-78	2.87E-77	Eukaryotic translation initiation factor 2 subunit 1

Involved in neuroactive ligand-receptor interaction

Px007620.1	10.3950	8.15E-10	3.63E-09	Trypsin CFT-1; similar with TRYP_CHOFU
Px006069.1	10.0136	2.26E-06	7.79E-06	Trypsin CFT-1; similar with TRYP_CHOFU
Px008696.1	8.2447	0.00E+00	0.00E+00	Osiris 9; similar with B6DXB0_BOMMO
Px006417.1	7.5446	3.51E-04	9.44E-04	Glutamate receptor 1; similar with GLK1_DROME
Px000336.1	6.9841	2.20E-107	4.36E-106	Osiris 20; similar with B6DXA7_BOMMO
Px008830.1	6.6433	1.43E-111	2.90E-110	Trypsin, alkaline C; similar with TRYC_MANSE
Px001804.1	6.5119	2.83E-51	3.39E-50	Trypsin, alkaline B; similar with TRYB_MANSE
Px007670.1	6.2472	5.75E-63	7.92E-62	Proclotting enzyme; similar with PCE_TACTR
Px007357.2	5.3379	0.00E+00	0.00E+00	Serine proteinase stubble; similar with STUB_DROME
Px010971.1	5.2472	1.05E-10	4.92E-10	Trypsin CFT-1; similar with TRYP_CHOFU
Px012645.1	5.1711	0.00E+00	0.00E+00	Neurofilament heavy polypeptide; similar with NFH_RAT
Px005240.1	5.1310	8.24E-124	1.79E-122	Trypsin, alkaline A; similar with TRYA_MANSE
Px014882.1	4.8506	4.25E-162	1.13E-160	Trypsin, alkaline C; similar with TRYC_MANSE
Px006831.1	4.8288	0.00E+00	0.00E+00	Putative uncharacterized protein; similar with D6X0U7_TRICA
Px008162.1	4.7017	7.16E-20	4.90E-19	Putative uncharacterized protein; similar with Q16SC6_AEDAE
Px005554.1	4.6008	8.20E-07	2.93E-06	Chymotrypsin-1; similar with CTR1_ANOGA
Px016100.1	4.5930	1.02E-46	1.15E-45	Coagulation factor IX; similar with FA9_RABIT
Px016055.1	4.5497	4.52E-56	5.70E-55	Trypsin, alkaline C; similar with TRYC_MANSE
Px002864.1	4.5298	1.55E-103	3.01E-102	Trypsin, alkaline B; similar with TRYB_MANSE
Px005241.1	4.3794	1.43E-53	1.74E-52	Trypsin CFT-1; similar with TRYP_CHOFU
Px007598.1	4.3759	1.54E-119	3.27E-118	Chymotrypsin-1; similar with CTR1_ANOGA
Px016053.1	4.2124	5.01E-59	6.53E-58	Trypsin; similar with TRYP_PHACE

Px009637.1	4.1028	1.35E-27	1.13E-26	Trypsin CFT-1; similar with TRYP_CHOFU
Px002586.1	4.0373	5.30E-58	6.84E-57	Trypsin CFT-1; similar with TRYP_CHOFU
Px013377.1	4.0330	2.06E-219	6.86E-218	Trypsin; similar with TRYP_PHACE
Px015276.1	4.0315	9.55E-65	1.33E-63	Trypsin CFT-1; similar with TRYP_CHOFU
Px005243.1	3.9876	0.00E+00	0.00E+00	Trypsin, alkaline A; similar with TRYA_MANSE
Px015277.1	3.9698	7.98E-158	2.09E-156	Trypsin CFT-1; similar with TRYP_CHOFU
Px010313.2	3.7896	2.43E-106	4.78E-105	Serine proteinase stubble; similar with STUB_DROME
Px006571.1	3.7885	0.00E+00	0.00E+00	Trypsin, alkaline B; similar with TRYB_MANSE
Px012813.1	3.6622	2.98E-06	1.02E-05	Serine proteinase stubble; similar with STUB_DROME
Px005239.1	3.6181	0.00E+00	0.00E+00	Trypsin, alkaline A; similar with TRYA_MANSE
Px007617.1	3.5958	0.00E+00	0.00E+00	Trypsin CFT-1; similar with TRYP_CHOFU
Px006572.1	3.4867	0.00E+00	0.00E+00	Trypsin; similar with TRYP_PHACE
Px004597.2	3.4696	2.05E-05	6.43E-05	Thyrotropin-releasing hormone receptor; similar with TRFR_RAT
Px005765.1	3.4325	1.33E-62	1.82E-61	Vitellin-degrading protease; similar with VDP_BOMMO
Px016056.1	3.4297	0.00E+00	0.00E+00	Trypsin, alkaline A; similar with TRYA_MANSE
Px008538.1	3.4202	3.15E-138	7.39E-137	Trypsin, alkaline B; similar with TRYB_MANSE
Px007621.1	3.3847	6.61E-21	4.65E-20	Trypsin CFT-1; similar with TRYP_CHOFU
Px014592.1	3.3707	9.04E-90	1.58E-88	Trypsin CFT-1; similar with TRYP_CHOFU
Px005238.1	3.3700	4.57E-59	5.96E-58	Trypsin, alkaline B; similar with TRYB_MANSE
Px009569.1	3.3179	0.00E+00	0.00E+00	Trypsin CFT-1; similar with TRYP_CHOFU
Px007901.1	3.2912	0.00E+00	0.00E+00	Chymotrypsin BI; similar with CTRB1_LITVA
Px008319.1	3.2789	4.00E-10	1.82E-09	Glutamate receptor 1; similar with GLR1_CAEEL
Px007599.1	3.2567	0.00E+00	0.00E+00	Chymotrypsin-1; similar with CTR1_ANOGA
Px002306.1	3.2472	7.34E-34	6.82E-33	Calcitonin receptor; similar with CALCR_RABIT
Px006767.1	3.2472	1.38E-04	3.92E-04	Chymotrypsin-1; similar with E2BNV4_9HYME
Px015279.1	3.2472	1.79E-18	1.17E-17	Trypsin, alkaline A; similar with TRYA_MANSE
Px012568.1	3.2398	0.00E+00	0.00E+00	Trypsin CFT-1; similar with TRYP_CHOFU
Px015278.1	3.1962	2.10E-27	1.75E-26	Trypsin, alkaline A; similar with TRYA_MANSE
Px010386.1	3.1714	0.00E+00	0.00E+00	Trypsin CFT-1; similar with TRYP_CHOFU
Px016054.1	3.1536	3.24E-37	3.20E-36	Trypsin; similar with TRYP_PHACE
Px007383.1	3.1028	1.03E-11	5.14E-11	PDF receptor; similar with PDFR_DROME
Px007619.2	3.0951	0.00E+00	0.00E+00	Trypsin, alkaline A; similar with TRYA_MANSE
Px012032.1	3.0773	4.83E-07	1.76E-06	Transposon Ty3-G Gag-Pol polyprotein; similar with YG31B_YEAST
Px002861.1	3.0431	0.00E+00	0.00E+00	Trypsin, alkaline C; similar with TRYC_MANSE
Px009639.1	3.0376	0.00E+00	0.00E+00	Trypsin CFT-1; similar with TRYP_CHOFU
Px006830.1	3.0315	8.88E-07	3.17E-06	Putative uncharacterized protein; similar with Q16SC6_AEDAE
Px005244.1	2.9796	7.91E-29	6.74E-28	Trypsin CFT-1; similar with TRYP_CHOFU
Px015280.1	2.9138	7.05E-10	3.15E-09	Trypsin CFT-1; similar with TRYP_CHOFU
Px006570.1	2.8972	0.00E+00	0.00E+00	Trypsin, alkaline B; similar with TRYB_MANSE
Px016057.1	2.8626	2.00E-77	3.18E-76	Trypsin, alkaline B; similar with TRYB_MANSE
Px009638.1	2.8523	0.00E+00	0.00E+00	Trypsin, alkaline B; similar with TRYB_MANSE
Px002587.1	2.7903	0.00E+00	0.00E+00	Trypsin CFT-1; similar with TRYP_CHOFU
Px010727.3	2.7386	4.24E-97	7.77E-96	Transposon Ty3-I Gag-Pol polyprotein; similar with YI31B_YEAST
Px000107.1	2.7335	0.00E+00	0.00E+00	Trypsin, alkaline C; similar with TRYC_MANSE
Px007618.1	2.7222	1.13E-272	4.41E-271	Trypsin CFT-1; similar with TRYP_CHOFU
Px011887.1	2.7196	0.00E+00	0.00E+00	Trypsin, alkaline C; similar with TRYC_MANSE
Px009286.1	2.7141	0.00E+00	0.00E+00	Trypsin, alkaline C; similar with TRYC_MANSE
Px005936.2	2.7085	3.12E-86	5.29E-85	Trypsin, alkaline C; similar with TRYC_MANSE
Px002442.1	2.6772	1.51E-16	9.17E-16	Trypsin, alkaline C; similar with TRYC_MANSE
Px002865.1	2.6726	0.00E+00	0.00E+00	Trypsin 5G1; similar with TRY5_AEDAE
Px002441.1	2.6127	4.36E-75	6.75E-74	Trypsin, alkaline B; similar with TRYB_MANSE
Px011889.1	2.5691	1.52E-21	1.09E-20	Achelase-1; similar with ACH1_LONAC
Px007600.1	2.5619	1.13E-226	3.92E-225	Chymotrypsin-1; similar with CTR1_ANOGA
Px008529.1	2.5323	1.56E-49	1.83E-48	Thyrotropin-releasing hormone receptor; similar with TRFR_CHICK

Px007675.1	2.5233	0.00E+00	0.00E+00	Collagenase; similar with COGS_HYPLI
Px002860.1	2.5102	4.25E-17	2.64E-16	Achelase-1; similar with ACH1_LONAC
Px007677.1	2.4787	0.00E+00	0.00E+00	Trypsin-7; similar with TRY7_ANOGA
Px012582.1	2.4724	2.13E-127	4.68E-126	Collagenase; similar with COGS_HYPLI
Px009488.1	2.4558	6.08E-05	1.81E-04	Chymotrypsin-like serine proteinase; similar with CTRL_HALRU
Px016058.1	2.4185	0.00E+00	0.00E+00	Trypsin, alkaline C; similar with TRYC_MANSE
Px002588.1	2.4151	4.81E-14	2.66E-13	Trypsin, alkaline C; similar with TRYC_MANSE
Px010489.1	2.4085	6.89E-66	9.73E-65	Trypsin, alkaline C; similar with TRYC_MANSE
Px009287.1	2.4027	1.27E-279	5.09E-278	Trypsin CFT-1; similar with TRYP_CHOFU
Px009395.1	2.3871	1.03E-17	6.47E-17	Proclotting enzyme; similar with PCE_TACTR
Px007616.1	2.3746	1.09E-158	2.87E-157	Trypsin CFT-1; similar with TRYP_CHOFU
Px005803.1	2.3700	5.55E-94	9.94E-93	Allatostatin-A receptor; similar with AR_BOMMO
Px005242.1	2.3668	2.59E-19	1.73E-18	Trypsin CFT-1; similar with TRYP_CHOFU
Px002589.1	2.3435	3.45E-96	6.29E-95	Trypsin, alkaline A; similar with TRYA_MANSE
Px015275.1	2.3286	0.00E+00	0.00E+00	Trypsin CFT-1; similar with TRYP_CHOFU
Px007601.1	2.2954	3.63E-31	3.23E-30	Chymotrypsin-1; similar with CTR1_ANOGA
Px017515.1	2.2558	2.50E-108	4.98E-107	Trypsin, alkaline C; similar with TRYC_MANSE
Px009567.1	2.2552	3.00E-104	5.83E-103	Trypsin, alkaline B; similar with TRYB_MANSE
Px009566.1	2.2251	1.29E-259	4.84E-258	Trypsin, alkaline C; similar with TRYC_MANSE
Px010612.1	2.1991	5.97E-76	9.32E-75	Serine/arginine repetitive matrix protein 5
Px000584.1	2.1984	3.86E-96	7.02E-95	Coagulation factor X; similar with FA10_RAT
Px000771.1	2.1857	1.04E-117	2.18E-116	Trypsin CFT-1; similar with TRYP_CHOFU
Px011886.1	2.1750	1.89E-125	4.14E-124	Trypsin CFT-1; similar with TRYP_CHOFU
Px001239.1	2.1600	2.79E-38	2.80E-37	Serine protease P162; similar with D2A5Y8_TRICA
Px010923.3	2.1086	9.06E-29	7.72E-28	Putative uncharacterized protein; similar with Q17GL6_AEDAE
Px004263.3	2.0773	1.72E-18	1.12E-17	Retrotransposable element Tf2 155 kDa protein type 1
Px001833.1	2.0603	0.00E+00	0.00E+00	Serine protease easter; similar with EAST_DROME
Px004167.1	2.0469	1.15E-06	4.06E-06	Plasma kallikrein; similar with KLKB1_MOUSE
Px012569.1	2.0285	0.00E+00	0.00E+00	Trypsin, alkaline C; similar with TRYC_MANSE
Px002440.1	2.0145	2.30E-21	1.64E-20	Trypsin, alkaline B; similar with TRYB_MANSE
Px012570.1	1.9654	0.00E+00	0.00E+00	Trypsin CFT-1; similar with TRYP_CHOFU
Px011097.1	1.9460	2.65E-10	1.22E-09	Serine proteinase stubble; similar with STUB_DROME
Px010975.1	1.8944	5.90E-09	2.48E-08	Trypsin, alkaline C; similar with TRYC_MANSE
Px002866.1	1.8658	5.28E-09	2.23E-08	Trypsin, alkaline A; similar with TRYA_MANSE
Px008406.1	1.8639	2.77E-08	1.10E-07	Serine proteinase stubble; similar with STUB_DROME
Px013665.3	1.8475	2.46E-37	2.43E-36	Collagenase; similar with COGS_HYPLI
Px005124.5	1.8306	2.07E-24	1.58E-23	Transposon Ty3-I Gag-Pol polyprotein; similar with YI31B_YEAST
Px012329.1	1.7693	5.39E-84	8.95E-83	Serine protease persephone; similar with PSH_DROME
Px011231.1	1.7453	1.76E-27	1.46E-26	Protein unc-50 homolog; similar with UNC50_DROME
Px014054.1	1.7402	9.14E-05	2.66E-04	Retrovirus-related Pol polyprotein from transposon 297
Px000402.2	1.6821	5.77E-83	9.50E-82	Serine protease persephone; similar with PSH_DROME
Px009088.1	1.6795	5.48E-174	1.55E-172	Mastin; similar with TRYM_CANFA
Px008995.1	1.6622	2.29E-04	6.29E-04	Uncharacterized protein K02A2.6; similar with YRD6_CAEEL
Px005342.1	1.6408	3.80E-156	9.82E-155	Collagenase; similar with COGS_HYPLI
Px001556.1	1.6061	8.94E-117	1.85E-115	Serine protease easter; similar with EAST_DROME
Px016723.1	1.5918	1.63E-04	4.61E-04	Calcitonin receptor; similar with CALCR_CAVPO
Px013974.1	1.5808	3.97E-38	3.98E-37	Leucine-rich repeat transmembrane neuronal protein 4
Px013162.1	1.5740	7.41E-151	1.88E-149	Serine protease snake; similar with SNAK_DROME
Px007239.2	1.5579	9.66E-11	4.55E-10	Diuretic hormone receptor; similar with DIHR_MANSE
Px005340.1	1.5499	1.24E-139	2.94E-138	Collagenase; similar with COGS_HYPLI
Px014831.2	1.4467	0.00E+00	0.00E+00	Serine proteinase stubble; similar with STUB_DROME
Px009568.1	1.3992	1.23E-04	3.53E-04	Trypsin, alkaline C; similar with TRYC_MANSE
Px005341.1	1.3783	1.92E-45	2.13E-44	Collagenase; similar with COGS_HYPLI
Px005947.1	1.3627	2.45E-06	8.39E-06	Serine proteinase stubble; similar with STUB_DROME

Px011101.1	1.3474	1.36E-56	1.73E-55	Coagulation factor X; similar with FA10_RAT
Px013795.1	1.2837	8.08E-05	2.37E-04	Putative neuropeptide Y receptor 11; similar with NPR11_CAEEL
Px015363.1	1.2365	5.17E-05	1.55E-04	Slit homolog 3 protein; similar with SLIT3_MOUSE
Px000103.2	1.2217	3.55E-133	8.06E-132	Prothrombin; similar with THRB_MOUSE
Px012531.1	1.2148	7.44E-05	2.19E-04	Transposon Ty3-I Gag-Pol polyprotein; similar with YI31B_YEAST
Px011499.2	1.1964	1.89E-18	1.23E-17	Serine protease persephone; similar with PSH_DROME
Px014830.1	1.1646	9.91E-167	2.71E-165	Tryptase; similar with TRYB1_MOUSE
Px003670.2	1.1636	0.00E+00	0.00E+00	Serine protease easter; similar with EAST_DROME
Px010855.1	1.1350	3.54E-11	1.72E-10	Trypsin, alkaline A; similar with TRYA_MANSE
Px009755.1	1.0920	5.93E-41	6.20E-40	Seminal fluid protein HACP060; similar with D9HQ61_9NEOP
Px016983.3	1.0379	6.64E-09	2.78E-08	Serine proteinase stubble; similar with STUB_DROME
Px000816.1	1.0221	1.94E-04	5.41E-04	Retrotransposable element Tf2 155 kDa protein type 1
Px002244.1	1.0207	2.03E-06	7.05E-06	Transposon Ty3-I Gag-Pol polyprotein; similar with YI31B_YEAST
Px013175.1	#######	1.24E-05	3.96E-05	Alpha-2B adrenergic receptor; similar with ADA2B_DANRE
Px001248.1	-8.6456	1.78E-04	4.98E-04	Orexin receptor type 2; similar with OX2R_CANFA
Px011383.1	-2.1912	1.92E-07	7.25E-07	Glutamate receptor, ionotropic kainate 3; similar with GRIK3_HUMAN
Px014636.1	-1.5417	2.28E-04	6.26E-04	Trypsin-1; similar with TRY1_ANOGA
Px004913.1	-1.4985	4.34E-286	1.75E-284	Putative uncharacterized protein; similar with D6W9I4_TRICA
Px002218.1	-1.4791	3.99E-08	1.58E-07	Beta-1 adrenergic receptor; similar with ADRB1_SHEEP
Px014434.1	-1.3509	4.77E-159	1.26E-157	Retrotransposable element Tf2 155 kDa protein type 1
Px008527.1	-1.3308	2.46E-09	1.06E-08	Retrotransposable element Tf2 155 kDa protein type 1
Px014679.1	-1.2335	5.49E-08	2.14E-07	Neuromedin-B receptor; similar with NMBR_MOUSE
Px010171.1	-1.2264	2.92E-19	1.95E-18	Retrovirus-related Pol polyprotein from transposon opus
Px002358.1	-1.1094	9.76E-12	4.86E-11	Glutamate receptor, ionotropic kainate 3; similar with GRIK3_HUMAN
Px000929.1	-1.0927	2.38E-04	6.54E-04	Octopamine receptor beta-2R; similar with OCTB2_DROME
Px015204.1	-1.0896	0.00E+00	0.00E+00	Transposon Ty3-I Gag-Pol polyprotein; similar with YI31B_YEAST
Px002507.1	-1.0338	1.62E-05	5.14E-05	Glutamate receptor, ionotropic kainate 2; similar with GRIK2_HUMAN

Involved in glycerolipid metabolism

Px000644.1	12.4260	4.78E-37	4.70E-36	Pancreatic triacylglycerol lipase; similar with LIPP_MOUSE
Px012560.1	10.5968	1.26E-07	4.83E-07	Lipase member H-B; similar with LIPHB_XENLA
Px004749.1	4.2513	0.00E+00	0.00E+00	Lipoprotein lipase; similar with LIPL_CAVPO
Px000948.1	4.2472	2.43E-05	7.55E-05	Gastric triacylglycerol lipase; similar with LIPG_RAT
Px012694.3	4.1304	9.29E-40	9.57E-39	Lipase 1; similar with LIP1_DROME
Px011791.1	4.1145	0.00E+00	0.00E+00	Lipase member H; similar with LIPH_XENTR
Px012011.2	4.0620	0.00E+00	0.00E+00	Pancreatic triacylglycerol lipase; similar with LIPP_MYOCO
Px004815.1	3.9017	3.38E-20	2.32E-19	Gut esterase 1; similar with EST1_CAEEL
Px007180.1	3.8846	2.52E-32	2.29E-31	Juvenile hormone esterase; similar with ESTJ_HELVI
Px005805.1	3.8398	5.29E-43	5.67E-42	Lipase 1; similar with LIP1_DROME
Px005804.1	3.4363	2.24E-142	5.42E-141	Lipase 1; similar with LIP1_DROME
Px009940.2	3.4221	6.33E-52	7.64E-51	Acetylcholinesterase; similar with ACES_CULPI
Px011477.2	3.1975	0.00E+00	0.00E+00	Lipase 1; similar with LIP1_DROME
Px003352.1	3.1927	1.50E-30	1.32E-29	Venom carboxylesterase-6; similar with EST6_APIME
Px001526.1	3.1070	4.54E-37	4.47E-36	Lipase 1; similar with LIP1_DROME
Px000371.1	3.0476	0.00E+00	0.00E+00	Lipase 1; similar with LIP1_DROME
Px012695.1	2.9423	3.93E-150	9.92E-149	Lipase 1; similar with LIP1_DROME
Px011683.2	2.9306	0.00E+00	0.00E+00	Venom carboxylesterase-6; similar with EST6_APIME
Px008089.1	2.8762	0.00E+00	0.00E+00	Pancreatic lipase-related protein 2; similar with LIPR2_CAVPO
Px006190.1	2.8647	0.00E+00	0.00E+00	Lipase member H-A; similar with LIPHA_XENLA
Px011788.2	2.8400	3.24E-222	1.09E-220	Pancreatic lipase-related protein 2; similar with LIPR2_BOVIN
Px008088.2	2.8173	0.00E+00	0.00E+00	Lipoprotein lipase; similar with LIPL_CAVPO
Px001527.1	2.7935	7.50E-09	3.13E-08	Lipase 1; similar with LIP1_DROME
Px017996.1	2.7512	1.00E-177	2.87E-176	Acetylcholinesterase; similar with ACES_HUMAN
Px000089.1	2.6887	2.89E-87	4.95E-86	Acetylcholinesterase; similar with ACES_FELCA

Px002735.1	2.6010	1.66E-257	6.15E-256	Esterase FE4; similar with ESTF_MYZPE
Px005193.2	2.5958	2.33E-141	5.61E-140	Pancreatic lipase-related protein 2; similar with LIPR2_MYOCO
Px009691.1	2.5676	5.36E-19	3.56E-18	Lipase 1; similar with LIP1_DROME
Px006345.1	2.5102	0.00E+00	0.00E+00	Pancreatic lipase-related protein 2; similar with LIPR2_MYOCO
Px011049.1	2.5090	4.88E-35	4.62E-34	Acetylcholinesterase; similar with ACES_FELCA
Px004750.1	2.4896	0.00E+00	0.00E+00	Pancreatic lipase-related protein 2; similar with LIPR2_RAT
Px002736.1	2.4868	0.00E+00	0.00E+00	Venom carboxylesterase-6; similar with EST6_APIME
Px001913.1	2.4737	1.20E-150	3.03E-149	Gastric triacylglycerol lipase; similar with LIPG_RAT
Px006430.1	2.4038	1.48E-302	6.22E-301	Esterase FE4; similar with ESTF_MYZPE
Px011475.1	2.3683	0.00E+00	0.00E+00	Lipase 3; similar with LIP3_DROME
Px004752.1	2.3527	0.00E+00	0.00E+00	Pancreatic lipase-related protein 2; similar with LIPR2_BOVIN
Px004753.1	2.3410	9.82E-208	3.12E-206	Pancreatic lipase-related protein 2; similar with LIPR2_MYOCO
Px006441.1	2.3113	0.00E+00	0.00E+00	Lipase member H; similar with LIPH_RAT
Px005876.1	2.3107	8.18E-70	1.20E-68	Patatin-like phospholipase domain-containing protein 2
Px012592.1	2.2775	0.00E+00	0.00E+00	Juvenile hormone esterase; similar with ESTJ_HELVI
Px001456.1	2.2249	2.79E-169	7.74E-168	Gastric triacylglycerol lipase; similar with LIPG_RAT
Px002296.1	2.1474	1.41E-169	3.92E-168	Pancreatic triacylglycerol lipase; similar with LIPP_HUMAN
Px003649.1	2.1363	0.00E+00	0.00E+00	Esterase FE4; similar with ESTF_MYZPE
Px002964.1	2.1362	2.49E-07	9.29E-07	Putative uncharacterized protein; similar with E9H4D7_DAPPU
Px003273.1	2.1129	6.40E-16	3.82E-15	Pancreatic lipase-related protein 2; similar with LIPR2_CAVPO
Px000744.1	2.0773	1.67E-04	4.72E-04	Lipase member H-A; similar with LIPHA_XENLA
Px006440.1	2.0668	1.56E-48	1.81E-47	Phospholipase A1 member A; similar with PLA1A_HUMAN
Px004200.1	2.0610	1.11E-81	1.81E-80	Putative phosphatidate phosphatase; similar with WUN_DROME
Px012696.1	2.0159	2.21E-135	5.07E-134	Lipase 3; similar with LIP3_DROME
Px000515.1	1.9774	1.03E-98	1.91E-97	Esterase FE4; similar with ESTF_MYZPE
Px004751.1	1.9293	0.00E+00	0.00E+00	Pancreatic triacylglycerol lipase; similar with LIPP_HUMAN
Px016338.1	1.9089	9.52E-63	1.31E-61	Lipase 1; similar with LIP1_DROME
Px011727.1	1.7734	6.73E-64	9.35E-63	Protein GCY; similar with GCY_YEAST
Px002295.1	1.7684	0.00E+00	0.00E+00	Pancreatic lipase-related protein 2; similar with LIPR2_BOVIN
Px000123.1	1.7598	0.00E+00	0.00E+00	Putative aldehyde dehydrogenase family 7 member A1 homolog
Px010260.3	1.7301	6.29E-25	4.87E-24	Alcohol dehydrogenase [NADP+] A; similar with A1A1A_DANRE
Px011755.1	1.7284	1.92E-39	1.96E-38	Antennal esterase CXE4; similar with D3GDL5_SPOLI
Px011476.1	1.7230	8.10E-19	5.34E-18	Lipase 3; similar with LIP3_DROME
Px003712.1	1.6490	1.05E-19	7.17E-19	Acetylcholinesterase; similar with ACES_CULPI
Px017997.1	1.6216	3.59E-04	9.62E-04	Esterase FE4; similar with ESTF_MYZPE
Px000132.1	1.5866	3.04E-79	4.87E-78	FGGY carbohydrate kinase domain-containing protein
Px007772.1	1.5076	2.80E-66	3.96E-65	Esterase B1; similar with EST1_CULPI
Px016066.1	1.4923	1.12E-13	6.06E-13	Lipase member M; similar with LIPM_MOUSE
Px011550.1	1.4749	3.05E-11	1.48E-10	Glycerate kinase; similar with GLCTK_RAT
Px006720.1	1.4070	1.32E-07	5.02E-07	Glycerol-3-phosphate acyltransferase 4; similar with GPAT4_PONAB
Px004571.1	1.3081	2.26E-185	6.68E-184	Aldose reductase; similar with ALDR_BOVIN
Px001937.2	1.2673	3.55E-115	7.31E-114	Phosphatidate phosphatase LPIN2; similar with LPIN2_MOUSE
Px002733.2	1.2093	0.00E+00	0.00E+00	Aldehyde dehydrogenase, dimeric NADP-preferring
Px000042.3	1.1085	4.45E-27	3.66E-26	Alcohol dehydrogenase [NADP+]; similar with AK1A1_BOVIN
Px016558.1	1.0773	3.20E-04	8.66E-04	Putative glycerol kinase 5; similar with GLPK5_DANRE
Px013239.1	1.0562	3.46E-07	1.28E-06	Putative glycerol kinase 3; similar with GLPK3_HUMAN
Px012098.2	1.0475	2.15E-05	6.71E-05	Lipase member I; similar with LIPI_HUMAN
Px011757.1	1.0401	5.69E-11	2.72E-10	Esterase FE4; similar with ESTF_MYZPE
Px015882.1	1.0071	2.32E-110	4.67E-109	Alcohol dehydrogenase [NADP+] A; similar with A1A1A_DANRE
Px012289.1	-1.0288	3.16E-06	1.07E-05	Liver carboxylesterase; similar with EST1_MESAU
Px004110.1	-1.0281	8.36E-07	2.99E-06	Aldose reductase; similar with ALDR_RABIT

Involved in fat digestion and absorption

Px000644.1	12.4260	4.78E-37	4.70E-36	Pancreatic triacylglycerol lipase; similar with LIPP_MOUSE

Px012560.1	10.5968	1.26E-07	4.83E-07	Lipase member H-B; similar with LIPHB_XENLA
Px000948.1	4.2472	2.43E-05	7.55E-05	Gastric triacylglycerol lipase; similar with LIPG_RAT
Px012694.3	4.1304	9.29E-40	9.57E-39	Lipase 1; similar with LIP1_DROME
Px011791.1	4.1145	0.00E+00	0.00E+00	Lipase member H; similar with LIPH_XENTR
Px012011.2	4.0620	0.00E+00	0.00E+00	Pancreatic triacylglycerol lipase; similar with LIPP_MYOCO
Px004815.1	3.9017	3.38E-20	2.32E-19	Gut esterase 1; similar with EST1_CAEEL
Px007180.1	3.8846	2.52E-32	2.29E-31	Juvenile hormone esterase; similar with ESTJ_HELVI
Px005805.1	3.8398	5.29E-43	5.67E-42	Lipase 1; similar with LIP1_DROME
Px008783.1	3.5367	1.08E-05	3.49E-05	Long-chain fatty acid transport protein 1; similar with S27A1_MOUSE
Px009940.2	3.4221	6.33E-52	7.64E-51	Acetylcholinesterase; similar with ACES_CULPI
Px003352.1	3.1927	1.50E-30	1.32E-29	Venom carboxylesterase-6; similar with EST6_APIME
Px011683.2	2.9306	0.00E+00	0.00E+00	Venom carboxylesterase-6; similar with EST6_APIME
Px008089.1	2.8762	0.00E+00	0.00E+00	Pancreatic lipase-related protein 2; similar with LIPR2_CAVPO
Px006190.1	2.8647	0.00E+00	0.00E+00	Lipase member H-A; similar with LIPHA_XENLA
Px011788.2	2.8400	3.24E-222	1.09E-220	Pancreatic lipase-related protein 2; similar with LIPR2_BOVIN
Px017996.1	2.7512	1.00E-177	2.87E-176	Acetylcholinesterase; similar with ACES_HUMAN
Px012537.1	2.7402	3.03E-04	8.22E-04	Apolipophorins; similar with APLP_LOCMI
Px000089.1	2.6887	2.89E-87	4.95E-86	Acetylcholinesterase; similar with ACES_FELCA
Px002735.1	2.6010	1.66E-257	6.15E-256	Esterase FE4; similar with ESTF_MYZPE
Px005193.2	2.5958	2.33E-141	5.61E-140	Pancreatic lipase-related protein 2; similar with LIPR2_MYOCO
Px009691.1	2.5676	5.36E-19	3.56E-18	Lipase 1; similar with LIP1_DROME
Px005199.1	2.5327	1.44E-159	3.81E-158	Long-chain fatty acid transport protein 4; similar with S27A4_MACFA
Px006345.1	2.5102	0.00E+00	0.00E+00	Pancreatic lipase-related protein 2; similar with LIPR2_MYOCO
Px011049.1	2.5090	4.88E-35	4.62E-34	Acetylcholinesterase; similar with ACES_FELCA
Px004750.1	2.4896	0.00E+00	0.00E+00	Pancreatic lipase-related protein 2; similar with LIPR2_RAT
Px002736.1	2.4868	0.00E+00	0.00E+00	Venom carboxylesterase-6; similar with EST6_APIME
Px013824.1	2.4844	6.34E-19	4.20E-18	Sensory neuron membrane protein 1; similar with SNMP1_PEDHC
Px006430.1	2.4038	1.48E-302	6.22E-301	Esterase FE4; similar with ESTF_MYZPE
Px004752.1	2.3527	0.00E+00	0.00E+00	Pancreatic lipase-related protein 2; similar with LIPR2_BOVIN
Px004753.1	2.3410	9.82E-208	3.12E-206	Pancreatic lipase-related protein 2; similar with LIPR2_MYOCO
Px006441.1	2.3113	0.00E+00	0.00E+00	Lipase member H; similar with LIPH_RAT
Px001337.1	2.2940	0.00E+00	0.00E+00	Putative uncharacterized protein; similar with A9NYL2_PICSI
Px012592.1	2.2775	0.00E+00	0.00E+00	Juvenile hormone esterase; similar with ESTJ_HELVI
Px001456.1	2.2249	2.79E-169	7.74E-168	Gastric triacylglycerol lipase; similar with LIPG_RAT
Px002296.1	2.1474	1.41E-169	3.92E-168	Pancreatic triacylglycerol lipase; similar with LIPP_HUMAN
Px003649.1	2.1363	0.00E+00	0.00E+00	Esterase FE4; similar with ESTF_MYZPE
Px003273.1	2.1129	6.40E-16	3.82E-15	Pancreatic lipase-related protein 2; similar with LIPR2_CAVPO
Px000744.1	2.0773	1.67E-04	4.72E-04	Lipase member H-A; similar with LIPHA_XENLA
Px004200.1	2.0610	1.11E-81	1.81E-80	Putative phosphatidate phosphatase; similar with WUN_DROME
Px012696.1	2.0159	2.21E-135	5.07E-134	Lipase 3; similar with LIP3_DROME
Px000515.1	1.9774	1.03E-98	1.91E-97	Esterase FE4; similar with ESTF_MYZPE
Px004751.1	1.9293	0.00E+00	0.00E+00	Pancreatic triacylglycerol lipase; similar with LIPP_HUMAN
Px008150.1	1.7972	2.12E-17	1.33E-16	Scavenger receptor class B member 1; similar with SCRB1_PIG
Px001510.4	1.7749	2.77E-85	4.66E-84	Scavenger receptor class B member 1; similar with SCRB1_PIG
Px002295.1	1.7684	0.00E+00	0.00E+00	Pancreatic lipase-related protein 2; similar with LIPR2_BOVIN
Px011335.1	1.7641	1.16E-13	6.27E-13	Scavenger receptor class B member 1; similar with SCRB1_BOVIN
Px012163.2	1.7447	5.09E-26	4.05E-25	Protein croquemort; similar with CRQ_DROME
Px011755.1	1.7284	1.92E-39	1.96E-38	Antennal esterase CXE4; similar with D3GDL5_SPOLI
Px003712.1	1.6490	1.05E-19	7.17E-19	Acetylcholinesterase; similar with ACES_CULPI
Px017997.1	1.6216	3.59E-04	9.62E-04	Esterase FE4; similar with ESTF_MYZPE
Px007772.1	1.5076	2.80E-66	3.96E-65	Esterase B1; similar with EST1_CULPI
Px014702.1	1.5035	1.75E-04	4.92E-04	Lysine-specific demethylase 8; similar with KDM8_RAT
Px011529.1	1.4501	6.50E-117	1.35E-115	Apolipophorins; similar with APLP_LOCMI
Px017838.1	1.3328	7.63E-25	5.89E-24	ATP-binding cassette sub-family A member 12

Px016733.1	1.2758	1.82E-09	7.92E-09	Acyl-CoA synthetase family member 3, mitochondrial
Px010271.1	1.2456	5.38E-14	2.96E-13	2-acylglycerol O-acyltransferase 1; similar with MOGT1_XENTR
Px004131.5	1.1474	6.20E-26	4.92E-25	Calcium-independent phospholipase A2-gamma
Px008080.1	1.0773	1.70E-05	5.37E-05	Lysosome membrane protein 2; similar with SCRB2_HUMAN
Px012098.2	1.0475	2.15E-05	6.71E-05	Lipase member I; similar with LIPI_HUMAN
Px005198.1	1.0445	2.21E-21	1.58E-20	Long-chain fatty acid transport protein 4; similar with S27A4_MACFA
Px011757.1	1.0401	5.69E-11	2.72E-10	Esterase FE4; similar with ESTF_MYZPE
Px004270.2	1.0203	2.08E-14	1.16E-13	Microsomal triglyceride transfer protein large subunit
Px012289.1	-1.0288	3.16E-06	1.07E-05	Liver carboxylesterase; similar with EST1_MESAU
**Involved in amoebiasis**				
Px005278.1	11.8601	5.93E-18	3.78E-17	Unknown functio
Px005318.1	11.3994	8.15E-10	3.63E-09	Cuticle protein 19; similar with CU19_LOCMI
Px008374.1	10.7904	2.99E-08	1.19E-07	Phosphatidylinositol-4,5-bisphosphate 3-kinase catalytic subunit delta isoform
Px012733.3	10.3612	1.64E-14	9.22E-14	Putative uncharacterized protein; similar with D6W9U7_TRICA
Px009397.1	10.2656	1.26E-07	4.83E-07	Translation initiation factor IF-2; similar with IF2_CHLPD
Px003372.1	9.1648	8.30E-05	2.43E-04	Putative uncharacterized protein; similar with Q16FD0_AEDAE
Px008029.1	9.1359	8.30E-05	2.43E-04	cAMP-dependent protein kinase catalytic subunit alpha
Px001530.1	8.7932	5.30E-259	1.98E-257	Neurofilament heavy polypeptide; similar with NFH_MOUSE
Px004419.1	7.3760	0.00E+00	0.00E+00	Putative uncharacterized protein GLEAN_08369
Px017104.1	6.1858	1.57E-99	2.94E-98	Neurofilament heavy polypeptide; similar with NFH_MOUSE
Px004755.2	6.1713	0.00E+00	0.00E+00	Putative uncharacterized protein; similar with D6W7K2_TRICA
Px000337.1	6.0185	2.92E-157	7.59E-156	Osiris 19; similar with B6DXA8_BOMMO
Px012644.1	5.1718	5.01E-218	1.66E-216	Filamentous hemagglutinin; similar with FHAB_BORPE
Px003684.1	4.9599	1.89E-62	2.57E-61	Cuticle protein 19; similar with CU19_LOCMI
Px001327.2	4.7211	1.68E-26	1.36E-25	Unknown functio
Px016161.1	4.6340	0.00E+00	0.00E+00	Neurofilament heavy polypeptide; similar with NFH_RAT
Px007897.1	4.2538	1.12E-103	2.16E-102	Peritrophic matrix insect intestinal mucin; similar with Q8ISS2_PLUXY
Px013975.1	4.1505	1.23E-71	1.83E-70	Cuticlin-1; similar with E2BP69_9HYME
Px005436.1	3.8846	3.51E-04	9.44E-04	Protein yellow; similar with YELL_DROSU
Px001082.1	3.7444	0.00E+00	0.00E+00	Unknown functio
Px008031.1	3.6920	1.06E-11	5.27E-11	cAMP-dependent protein kinase catalytic subunit alpha
Px002960.1	3.6078	1.03E-44	1.13E-43	Transforming growth factor-beta-induced protein ig-h3
Px013635.1	3.5312	9.71E-216	3.19E-214	Extracellular domains-containing protein CG31004
Px007895.2	3.4975	0.00E+00	0.00E+00	Peritrophic matrix insect intestinal mucin; similar with Q8ISS2_PLUXY
Px005969.1	3.4592	0.00E+00	0.00E+00	Apolipoprotein D; similar with APOD_MACFA
Px002615.1	3.4179	7.59E-94	1.36E-92	C-type lectin, galactose-binding (AGAP010196-PA)
Px000651.1	3.3574	8.65E-121	1.85E-119	Extracellular domains-containing protein CG31004
Px000745.1	3.3391	1.06E-92	1.88E-91	Tenascin-X; similar with TENX_HUMAN
Px001443.1	3.2620	0.00E+00	0.00E+00	L-ascorbate oxidase; similar with ASO_CUCPM
Px006951.2	3.2472	4.09E-08	1.62E-07	Putative uncharacterized protein; similar with D6WTK7_TRICA
Px013918.1	3.2126	0.00E+00	0.00E+00	Putative uncharacterized protein; similar with B0XCG6_CULQU
Px007274.1	3.2065	2.52E-47	2.88E-46	Serpin B9; similar with SPB9_HUMAN
Px010970.1	3.1720	7.67E-57	9.77E-56	Cuticle protein; similar with CU01_ANOGA
Px001081.1	3.1539	0.00E+00	0.00E+00	Unknown functio
Px009152.1	3.1248	6.67E-86	1.13E-84	Innexin inx7; similar with INX7_DROME
Px003835.1	3.0608	1.01E-94	1.81E-93	Putative uncharacterized protein; similar with B0WNP5_CULQU
Px005321.1	3.0407	2.97E-08	1.18E-07	Cuticle protein 19; similar with CU19_LOCMI
Px005323.1	2.9253	4.27E-26	3.40E-25	Cuticle protein 19; similar with CU19_LOCMI
Px009987.1	2.8978	1.47E-38	1.48E-37	Caspase-1; similar with CASP1_DROME
Px008604.1	2.8677	2.19E-157	5.71E-156	AGAP011476-PA; similar with Q7Q343_ANOGA
Px008030.1	2.8370	3.71E-26	2.97E-25	cAMP-dependent protein kinase catalytic subunit
Px006536.1	2.8353	0.00E+00	0.00E+00	HMG176; similar with Q0MTA5_HELAM
Px005322.1	2.8116	0.00E+00	0.00E+00	Cuticle protein 19; similar with CU19_LOCMI

Px009784.1	2.6561	0.00E+00	0.00E+00	Ribosome-binding protein 1; similar with RRBP1_HUMAN
Px009883.1	2.6507	1.03E-20	7.20E-20	Putative uncharacterized protein GLEAN_08340
Px003497.1	2.6461	4.06E-15	2.34E-14	Putative uncharacterized protein; similar with E9J4L9_SOLIN
Px001080.1	2.6021	0.00E+00	0.00E+00	Unknown functio
Px001671.1	2.5627	1.09E-09	4.81E-09	Putative cuticle protein; similar with C0H6D6_BOMMO
Px014252.1	2.4415	3.67E-69	5.34E-68	Putative uncharacterized protein; similar with D6WG20_TRICA
Px010337.1	2.3427	6.05E-128	1.34E-126	Cuticular protein CPR77; similar with B2DBI2_9NEOP
Px015898.1	2.3215	0.00E+00	0.00E+00	Putative farnesoic acid O-methyl transferase
Px001077.1	2.3052	0.00E+00	0.00E+00	Unknown functio
Px002650.1	2.2907	5.34E-43	5.71E-42	Chitin binding protein; similar with B0WJ01_CULQU
Px002795.1	2.2574	1.02E-55	1.28E-54	Laccase-4; similar with LAC4_TRAVE
Px003776.1	2.1246	9.72E-09	4.02E-08	TBC1 domain family member 30; similar with TBC30_XENTR
Px014841.1	2.1217	5.76E-83	9.49E-82	Unknown functio
Px015340.1	2.1102	2.20E-89	3.82E-88	Putative uncharacterized protein; similar with D6WEC2_TRICA
Px002424.1	1.9989	4.37E-161	1.16E-159	Serpin B3; similar with SPB3_HUMAN
Px012786.1	1.9892	0.00E+00	0.00E+00	GH11122; similar with B4JCY6_DROGR
Px001076.1	1.9838	0.00E+00	0.00E+00	Fibrohexamerin; similar with SI25_GALME
Px009301.1	1.9550	1.51E-125	3.31E-124	Beta-1,3-glucan-binding protein; similar with BGBP_BOMMO
Px015087.3	1.8241	0.00E+00	0.00E+00	Glia-derived nexin; similar with GDN_HUMAN
Px000622.1	1.8077	2.17E-08	8.71E-08	Protein spaetzle; similar with E2CAB2_9HYME
Px011577.1	1.8036	1.23E-82	2.03E-81	Plasminogen activator inhibitor 1; similar with PAI1_PIG
Px008680.1	1.7601	5.23E-07	1.90E-06	Putative dsRNase; similar with B7VCC3_SPOLI
Px015090.1	1.7115	0.00E+00	0.00E+00	Serpin B6; similar with SPB6_HUMAN
Px004235.2	1.7053	1.90E-19	1.28E-18	Putative uncharacterized protein; similar with Q178N6_AEDAE
Px015730.1	1.7021	0.00E+00	0.00E+00	Apolipophorins; similar with APLP_MANSE
Px017059.1	1.6853	1.12E-190	3.37E-189	Serpin B6; similar with SPB6_HUMAN
Px012136.3	1.6501	2.67E-51	3.20E-50	Transferrin; similar with TRF_BLADI
Px001047.1	1.6266	1.35E-135	3.11E-134	Heat shock protein 67B2; similar with HS6B_DROME
Px003251.1	1.6237	1.02E-19	6.98E-19	Putative cuticle protein; similar with C0H6E8_BOMMO
Px002015.1	1.6107	7.78E-06	2.55E-05	AGAP008123-PA; similar with Q7Q401_ANOGA
Px017058.1	1.5999	3.21E-34	3.01E-33	Serpin B6; similar with SPB6_MOUSE
Px015729.1	1.5960	2.06E-13	1.10E-12	Vinculin; similar with VINC_DROME
Px007271.1	1.5855	0.00E+00	0.00E+00	Serpin B9; similar with SPB9_HUMAN
Px007272.1	1.5808	3.47E-77	5.48E-76	Leukocyte elastase inhibitor; similar with ILEU_BOVIN
Px001494.2	1.5662	0.00E+00	0.00E+00	Alaserpin; similar with SERA_MANSE
Px013987.1	1.5512	3.40E-05	1.04E-04	Putative uncharacterized transposon-derived protein F52C9.6
Px002809.2	1.5031	7.52E-18	4.78E-17	WD repeat domain phosphoinositide-interacting protein 4
Px011646.1	1.4885	1.68E-10	7.79E-10	GA10261; similar with Q29B54_DROPS
Px010627.1	1.4167	1.53E-153	3.89E-152	Wing disc-specific protein; similar with Q8WPH2_BOMMO
Px011466.1	1.3882	2.99E-14	1.66E-13	Putative uncharacterized protein; similar with F0Y4G7_9STRA
Px013252.1	1.3766	0.00E+00	0.00E+00	Calexcitin-2; similar with CEX2_CAEEL
Px015383.1	1.3727	8.33E-05	2.43E-04	Latrophilin-like receptor; similar with D7EJP1_TRICA
Px011737.1	1.3544	0.00E+00	0.00E+00	Neurofilament heavy polypeptide; similar with NFH_MOUSE
Px015923.1	1.3371	5.16E-07	1.88E-06	OTU domain-containing protein 1; similar with OTUD1_HUMAN
Px015089.1	1.3270	0.00E+00	0.00E+00	Serpin B9; similar with SPB9_HUMAN
Px007816.1	1.2997	3.29E-04	8.88E-04	AGAP010146-PA; similar with Q7Q0V0_ANOGA
Px003823.1	1.2692	4.31E-175	1.22E-173	Plasminogen activator inhibitor 1; similar with PAI1_MUSVI
Px013298.1	1.2577	3.36E-51	4.02E-50	Serine proteinase-like protein; similar with Q1HPQ5_BOMMO
Px008882.1	1.2542	1.19E-128	2.65E-127	Unknown functio
Px007273.1	1.2484	5.02E-34	4.68E-33	Plasminogen activator inhibitor 1; similar with PAI1_HUMAN
Px005957.1	1.2387	4.85E-06	1.62E-05	LIM and SH3 domain protein Lasp; similar with LASP1_DROME
Px015363.1	1.2365	5.17E-05	1.55E-04	Slit homolog 3 protein; similar with SLIT3_MOUSE
Px001430.1	1.1554	0.00E+00	0.00E+00	Chitin deacetylase 1; similar with B2ZGH5_9NEOP
Px013919.1	1.1490	0.00E+00	0.00E+00	Collagen alpha-4(VI) chain; similar with CO6A4_MOUSE

Px015683.1	1.1458	8.18E-148	2.04E-146	Protein yellow; similar with YELL_DROYA
Px002792.1	1.0827	4.51E-25	3.50E-24	Unknown functio
Px008178.7	1.0520	8.69E-16	5.15E-15	Vacuolar protein sorting-associated protein 13A
Px007399.1	1.0453	1.62E-14	9.12E-14	Putative uncharacterized protein; similar with D6WDQ4_TRICA
Px012264.1	1.0389	7.52E-138	1.76E-136	Ejaculatory bulb-specific protein 3; similar with PEB3_DROME
Px011876.1	-8.7166	3.46E-04	9.33E-04	Armadillo repeat-containing protein 2; similar with ARMC2_HUMAN
Px008648.1	-4.7573	9.33E-102	1.78E-100	Larval/pupal rigid cuticle protein 66; similar with CU66_HYACE
Px001065.1	-3.9118	4.45E-217	1.47E-215	Cuticle protein; similar with CU01_ANOGA
Px008500.1	-3.8767	0.00E+00	0.00E+00	Pupal cuticle protein; similar with CUPP_BOMMO
Px008653.1	-3.3337	5.01E-149	1.26E-147	Cuticle protein; similar with CU01_ANOGA
Px014536.1	-2.9107	4.25E-133	9.63E-132	Unknown functio
Px001066.1	-2.8804	7.21E-117	1.50E-115	Cuticle protein; similar with CU01_ANOGA
Px016485.1	-2.7098	3.20E-167	8.79E-166	Pupal cuticle protein; similar with CUPP_BOMMO
Px008652.2	-2.6586	0.00E+00	0.00E+00	Cuticle protein; similar with CU01_ANOGA
Px007290.1	-2.5885	0.00E+00	0.00E+00	Cuticle protein; similar with CU01_ANOGA
Px003252.2	-2.5383	0.00E+00	0.00E+00	Larval cuticle protein LCP-30; similar with CU30_BOMMO
Px003249.1	-2.5279	0.00E+00	0.00E+00	Larval cuticle protein LCP-22; similar with CU22_BOMMO
Px008651.1	-2.3378	2.32E-17	1.45E-16	Cuticle protein; similar with CU01_ANOGA
Px001064.1	-2.2939	0.00E+00	0.00E+00	Pupal cuticle protein Edg-84A; similar with CUP8_DROME
Px014265.1	-2.2155	7.66E-13	4.01E-12	Unknown functio
Px014262.1	-2.1613	8.02E-226	2.76E-224	Putative cuticle protein; similar with C0H6H1_BOMMO
Px008650.1	-2.1060	0.00E+00	0.00E+00	Cuticle protein; similar with CU01_ANOGA
Px005288.1	-2.0903	2.09E-66	2.97E-65	Putative cuticle protein; similar with Q9U504_MANSE
Px014261.1	-1.7691	0.00E+00	0.00E+00	Putative cuticle protein; similar with C0H6H2_BOMMO
Px003250.1	-1.6890	8.64E-94	1.54E-92	Larval cuticle protein LCP-30; similar with CU30_BOMMO
Px001656.1	-1.6562	2.78E-23	2.07E-22	Vitellogenin; similar with VIT_BOMMO
Px013556.1	-1.6442	0.00E+00	0.00E+00	Flexible cuticle protein 12; similar with CU12_HYACE
Px013555.1	-1.6367	0.00E+00	0.00E+00	Flexible cuticle protein 12; similar with CU12_HYACE
Px008655.1	-1.5915	6.52E-163	1.74E-161	Cuticle protein; similar with CU01_ANOGA
Px002974.3	-1.5617	0.00E+00	0.00E+00	Myofilin variant A; similar with C3UZ70_BOMMA
Px014245.1	-1.5602	2.08E-05	6.52E-05	Unknown functio
Px000548.1	-1.4120	0.00E+00	0.00E+00	Alpha-actinin, sarcomeric; similar with ACTN_DROME
Px008654.1	-1.3873	7.28E-49	8.45E-48	Pupal cuticle protein Edg-84A; similar with CUP8_DROME
Px002640.1	-1.2858	0.00E+00	0.00E+00	Alpha-actinin, sarcomeric; similar with ACTN_DROME
Px001199.1	-1.2619	0.00E+00	0.00E+00	Fibroin light chain; similar with FIBL_GALME
Px003254.3	-1.2597	0.00E+00	0.00E+00	Larval cuticle protein LCP-17; similar with CU17_BOMMO
Px014247.3	-1.2526	0.00E+00	0.00E+00	Cuticular protein CPR77; similar with B2DBI2_9NEOP
Px011301.2	-1.1671	2.31E-91	4.07E-90	Putative uncharacterized protein; similar with D6WH87_TRICA
Px014818.1	-1.0682	2.80E-04	7.64E-04	Ecdysteroid 22-phosphate; similar with Q0PCR8_BOMMO
Px003245.1	-1.0530	8.39E-301	3.51E-299	Larval cuticle protein LCP-22; similar with CU22_BOMMO
Px003255.1	-1.0353	0.00E+00	0.00E+00	Larval cuticle protein LCP-17; similar with CU17_BOMMO
Px010466.1	-1.0231	0.00E+00	0.00E+00	Repetitive proline-rich cell wall protein 2; similar with PRP2_MEDTR
Px013606.1	-1.0228	0.00E+00	0.00E+00	Unknown functio

Involved in vibrio cholerae infection

Px005278.1	11.8601	5.93E-18	3.78E-17	Unknown functio
Px005318.1	11.3994	8.15E-10	3.63E-09	Cuticle protein 19; similar with CU19_LOCMI
Px012733.3	10.3612	1.64E-14	9.22E-14	Putative uncharacterized protein; similar with D6W9U7_TRICA
Px009397.1	10.2656	1.26E-07	4.83E-07	Translation initiation factor IF-2; similar with IF2_CHLPD
Px003372.1	9.1648	8.30E-05	2.43E-04	Putative uncharacterized protein; similar with Q16FD0_AEDAE
Px008029.1	9.1359	8.30E-05	2.43E-04	cAMP-dependent protein kinase catalytic subunit alpha
Px001530.1	8.7932	5.30E-259	1.98E-257	Neurofilament heavy polypeptide; similar with NFH_MOUSE
Px004419.1	7.3760	0.00E+00	0.00E+00	Putative uncharacterized protein GLEAN_08369
Px017104.1	6.1858	1.57E-99	2.94E-98	Neurofilament heavy polypeptide; similar with NFH_MOUSE

Px004755.2	6.1713	0.00E+00	0.00E+00	Putative uncharacterized protein; similar with D6W7K2_TRICA
Px000337.1	6.0185	2.92E-157	7.59E-156	Osiris 19; similar with B6DXA8_BOMMO
Px012644.1	5.1718	5.01E-218	1.66E-216	Filamentous hemagglutinin; similar with FHAB_BORPE
Px003684.1	4.9599	1.89E-62	2.57E-61	Cuticle protein 19; similar with CU19_LOCMI
Px001327.2	4.7211	1.68E-26	1.36E-25	Unknown functio
Px016161.1	4.6340	0.00E+00	0.00E+00	Neurofilament heavy polypeptide; similar with NFH_RAT
Px007897.1	4.2538	1.12E-103	2.16E-102	Peritrophic matrix insect intestinal mucin; similar with Q8ISS2_PLUXY
Px013975.1	4.1505	1.23E-71	1.83E-70	Cuticlin-1; similar with E2BP69_9HYME
Px005436.1	3.8846	3.51E-04	9.44E-04	Protein yellow; similar with YELL_DROSU
Px001082.1	3.7444	0.00E+00	0.00E+00	Unknown functio
Px008031.1	3.6920	1.06E-11	5.27E-11	cAMP-dependent protein kinase catalytic subunit alpha
Px002960.1	3.6078	1.03E-44	1.13E-43	Transforming growth factor-beta-induced protein ig-h3
Px013635.1	3.5312	9.71E-216	3.19E-214	Extracellular domains-containing protein CG31004
Px007895.2	3.4975	0.00E+00	0.00E+00	Peritrophic matrix insect intestinal mucin; similar with Q8ISS2_PLUXY
Px005969.1	3.4592	0.00E+00	0.00E+00	Apolipoprotein D; similar with APOD_MACFA
Px002615.1	3.4179	7.59E-94	1.36E-92	C-type lectin, galactose-binding (AGAP010196-PA)
Px000651.1	3.3574	8.65E-121	1.85E-119	Extracellular domains-containing protein CG31004
Px001443.1	3.2620	0.00E+00	0.00E+00	L-ascorbate oxidase; similar with ASO_CUCPM
Px006951.2	3.2472	4.09E-08	1.62E-07	Putative uncharacterized protein; similar with D6WTK7_TRICA
Px013918.1	3.2126	0.00E+00	0.00E+00	Putative uncharacterized protein; similar with B0XCG6_CULQU
Px010970.1	3.1720	7.67E-57	9.77E-56	Cuticle protein; similar with CU01_ANOGA
Px001081.1	3.1539	0.00E+00	0.00E+00	Unknown functio
Px009152.1	3.1248	6.67E-86	1.13E-84	Innexin inx7; similar with INX7_DROME
Px009080.1	3.0773	6.20E-26	4.92E-25	Vacuolar ATP synthase subunit S1, putative
Px003835.1	3.0608	1.01E-94	1.81E-93	Putative uncharacterized protein; similar with B0WNP5_CULQU
Px005321.1	3.0407	2.97E-08	1.18E-07	Cuticle protein 19; similar with CU19_LOCMI
Px005323.1	2.9253	4.27E-26	3.40E-25	Cuticle protein 19; similar with CU19_LOCMI
Px008604.1	2.8677	2.19E-157	5.71E-156	AGAP011476-PA; similar with Q7Q343_ANOGA
Px008030.1	2.8370	3.71E-26	2.97E-25	cAMP-dependent protein kinase catalytic subunit
Px006536.1	2.8353	0.00E+00	0.00E+00	HMG176; similar with Q0MTA5_HELAM
Px005322.1	2.8116	0.00E+00	0.00E+00	Cuticle protein 19; similar with CU19_LOCMI
Px009784.1	2.6561	0.00E+00	0.00E+00	Ribosome-binding protein 1; similar with RRBP1_HUMAN
Px009883.1	2.6507	1.03E-20	7.20E-20	Putative uncharacterized protein GLEAN_08340
Px003497.1	2.6461	4.06E-15	2.34E-14	Putative uncharacterized protein; similar with E9J4L9_SOLIN
Px001080.1	2.6021	0.00E+00	0.00E+00	Unknown functio
Px001671.1	2.5627	1.09E-09	4.81E-09	Putative cuticle protein; similar with C0H6D6_BOMMO
Px014252.1	2.4415	3.67E-69	5.34E-68	Putative uncharacterized protein; similar with D6WG20_TRICA
Px010337.1	2.3427	6.05E-128	1.34E-126	Cuticular protein CPR77; similar with B2DBI2_9NEOP
Px015898.1	2.3215	0.00E+00	0.00E+00	Putative farnesoic acid O-methyl transferase
Px001077.1	2.3052	0.00E+00	0.00E+00	Unknown functio
Px002650.1	2.2907	5.34E-43	5.71E-42	Chitin binding protein; similar with B0WJ01_CULQU
Px002795.1	2.2574	1.02E-55	1.28E-54	Laccase-4; similar with LAC4_TRAVE
Px007927.1	2.2154	4.79E-60	6.34E-59	V-type proton ATPase subunit G; similar with VATG_MANSE
Px003776.1	2.1246	9.72E-09	4.02E-08	TBC1 domain family member 30; similar with TBC30_XENTR
Px014841.1	2.1217	5.76E-83	9.49E-82	Unknown functio
Px015340.1	2.1102	2.20E-89	3.82E-88	Putative uncharacterized protein; similar with D6WEC2_TRICA
Px012786.1	1.9892	0.00E+00	0.00E+00	GH11122; similar with B4JCY6_DROGR
Px001076.1	1.9838	0.00E+00	0.00E+00	Fibrohexamerin; similar with SI25_GALME
Px009301.1	1.9550	1.51E-125	3.31E-124	Beta-1,3-glucan-binding protein; similar with BGBP_BOMMO
Px000622.1	1.8077	2.17E-08	8.71E-08	Protein spaetzle; similar with E2CAB2_9HYME
Px008680.1	1.7601	5.23E-07	1.90E-06	Putative dsRNase; similar with B7VCC3_SPOLI
Px004235.2	1.7053	1.90E-19	1.28E-18	Putative uncharacterized protein; similar with Q178N6_AEDAE
Px015730.1	1.7021	0.00E+00	0.00E+00	Apolipophorins; similar with APLP_MANSE
Px012136.3	1.6501	2.67E-51	3.20E-50	Transferrin; similar with TRF_BLADI

Px001047.1	1.6266	1.35E-135	3.11E-134	Heat shock protein 67B2; similar with HS6B_DROME
Px002015.1	1.6107	7.78E-06	2.55E-05	AGAP008123-PA; similar with Q7Q401_ANOGA
Px011646.1	1.4885	1.68E-10	7.79E-10	GA10261; similar with Q29B54_DROPS
Px011466.1	1.3882	2.99E-14	1.66E-13	Putative uncharacterized protein; similar with F0Y4G7_9STRA
Px013252.1	1.3766	0.00E+00	0.00E+00	Calexcitin-2; similar with CEX2_CAEEL
Px015383.1	1.3727	8.33E-05	2.43E-04	Latrophilin-like receptor; similar with D7EJP1_TRICA
Px015301.3	1.3558	5.98E-18	3.81E-17	V-type proton ATPase subunit S1; similar with VAS1_MOUSE
Px000146.1	1.3503	5.61E-05	1.68E-04	Adenylate cyclase type 9; similar with ADCY9_HUMAN
Px015923.1	1.3371	5.16E-07	1.88E-06	OTU domain-containing protein 1; similar with OTUD1_HUMAN
Px007816.1	1.2997	3.29E-04	8.88E-04	AGAP010146-PA; similar with Q7Q0V0_ANOGA
Px013298.1	1.2577	3.36E-51	4.02E-50	Serine proteinase-like protein; similar with Q1HPQ5_BOMMO
Px005957.1	1.2387	4.85E-06	1.62E-05	LIM and SH3 domain protein Lasp; similar with LASP1_DROME
Px001430.1	1.1554	0.00E+00	0.00E+00	Chitin deacetylase 1; similar with B2ZGH5_9NEOP
Px015683.1	1.1458	8.18E-148	2.04E-146	Protein yellow; similar with YELL_DROYA
Px008178.7	1.0520	8.69E-16	5.15E-15	Vacuolar protein sorting-associated protein 13A
Px007399.1	1.0453	1.62E-14	9.12E-14	Putative uncharacterized protein; similar with D6WDQ4_TRICA
Px012264.1	1.0389	7.52E-138	1.76E-136	Ejaculatory bulb-specific protein 3; similar with PEB3_DROME
Px005056.1	1.0223	1.78E-132	4.01E-131	V-type proton ATPase subunit e 2; similar with VA0E2_HUMAN
Px008648.1	-4.7573	9.33E-102	1.78E-100	Larval/pupal rigid cuticle protein 66; similar with CU66_HYACE
Px001065.1	-3.9118	4.45E-217	1.47E-215	Cuticle protein; similar with CU01_ANOGA
Px008500.1	-3.8767	0.00E+00	0.00E+00	Pupal cuticle protein; similar with CUPP_BOMMO
Px003126.1	-3.3822	3.42E-05	1.05E-04	Adenylate cyclase type 3; similar with ADCY3_MOUSE
Px008653.1	-3.3337	5.01E-149	1.26E-147	Cuticle protein; similar with CU01_ANOGA
Px014536.1	-2.9107	4.25E-133	9.63E-132	Unknown functio
Px001066.1	-2.8804	7.21E-117	1.50E-115	Cuticle protein; similar with CU01_ANOGA
Px016485.1	-2.7098	3.20E-167	8.79E-166	Pupal cuticle protein; similar with CUPP_BOMMO
Px008652.2	-2.6586	0.00E+00	0.00E+00	Cuticle protein; similar with CU01_ANOGA
Px007290.1	-2.5885	0.00E+00	0.00E+00	Cuticle protein; similar with CU01_ANOGA
Px003252.2	-2.5383	0.00E+00	0.00E+00	Larval cuticle protein LCP-30; similar with CU30_BOMMO
Px003249.1	-2.5279	0.00E+00	0.00E+00	Larval cuticle protein LCP-22; similar with CU22_BOMMO
Px008651.1	-2.3378	2.32E-17	1.45E-16	Cuticle protein; similar with CU01_ANOGA
Px001064.1	-2.2939	0.00E+00	0.00E+00	Pupal cuticle protein Edg-84A; similar with CUP8_DROME
Px014265.1	-2.2155	7.66E-13	4.01E-12	Unknown functio
Px014262.1	-2.1613	8.02E-226	2.76E-224	Putative cuticle protein; similar with C0H6H1_BOMMO
Px008650.1	-2.1060	0.00E+00	0.00E+00	Cuticle protein; similar with CU01_ANOGA
Px005288.1	-2.0903	2.09E-66	2.97E-65	Putative cuticle protein; similar with Q9U504_MANSE
Px014261.1	-1.7691	0.00E+00	0.00E+00	Putative cuticle protein; similar with C0H6H2_BOMMO
Px003250.1	-1.6890	8.64E-94	1.54E-92	Larval cuticle protein LCP-30; similar with CU30_BOMMO
Px001656.1	-1.6562	2.78E-23	2.07E-22	Vitellogenin; similar with VIT_BOMMO
Px013556.1	-1.6442	0.00E+00	0.00E+00	Flexible cuticle protein 12; similar with CU12_HYACE
Px013555.1	-1.6367	0.00E+00	0.00E+00	Flexible cuticle protein 12; similar with CU12_HYACE
Px008655.1	-1.5915	6.52E-163	1.74E-161	Cuticle protein; similar with CU01_ANOGA
Px002974.3	-1.5617	0.00E+00	0.00E+00	Myofilin variant A; similar with C3UZ70_BOMMA
Px014245.1	-1.5602	2.08E-05	6.52E-05	Unknown functio
Px013456.1	-1.4780	9.11E-21	6.38E-20	Actin, clone 211; similar with ACT2_ARTSX
Px008654.1	-1.3873	7.28E-49	8.45E-48	Pupal cuticle protein Edg-84A; similar with CUP8_DROME
Px003840.1	-1.3269	0.00E+00	0.00E+00	Actin, muscle; similar with ACT_MANSE
Px001199.1	-1.2619	0.00E+00	0.00E+00	Fibroin light chain; similar with FIBL_GALME
Px003254.3	-1.2597	0.00E+00	0.00E+00	Larval cuticle protein LCP-17; similar with CU17_BOMMO
Px014247.3	-1.2526	0.00E+00	0.00E+00	Cuticular protein CPR77; similar with B2DBI2_9NEOP
Px011301.2	-1.1671	2.31E-91	4.07E-90	Putative uncharacterized protein; similar with D6WH87_TRICA
Px000220.2	-1.1220	7.78E-07	2.79E-06	Bumetanide-sensitive sodium-(potassium)-chloride cotransporter
Px014818.1	-1.0682	2.80E-04	7.64E-04	Ecdysteroid 22-phosphate; similar with Q0PCR8_BOMMO
Px003245.1	-1.0530	8.39E-301	3.51E-299	Larval cuticle protein LCP-22; similar with CU22_BOMMO

Px003255.1	-1.0353	0.00E+00	0.00E+00	Larval cuticle protein LCP-17; similar with CU17_BOMMO
Px003808.1	-1.0231	0.00E+00	0.00E+00	Actin, muscle; similar with ACT_MANSE
Px010466.1	-1.0231	0.00E+00	0.00E+00	Repetitive proline-rich cell wall protein 2
Px013606.1	-1.0228	0.00E+00	0.00E+00	Unknown functio

Involved in steroid biosynthesis

Px000948.1	4.2472	2.43E-05	7.55E-05	Gastric triacylglycerol lipase; similar with LIPG_RAT
Px012694.3	4.1304	9.29E-40	9.57E-39	Lipase 1; similar with LIP1_DROME
Px004815.1	3.9017	3.38E-20	2.32E-19	Gut esterase 1; similar with EST1_CAEEL
Px007180.1	3.8846	2.52E-32	2.29E-31	Juvenile hormone esterase; similar with ESTJ_HELVI
Px005805.1	3.8398	5.29E-43	5.67E-42	Lipase 1; similar with LIP1_DROME
Px005804.1	3.4363	2.24E-142	5.42E-141	Lipase 1; similar with LIP1_DROME
Px009940.2	3.4221	6.33E-52	7.64E-51	Acetylcholinesterase; similar with ACES_CULPI
Px011477.2	3.1975	0.00E+00	0.00E+00	Lipase 1; similar with LIP1_DROME
Px003352.1	3.1927	1.50E-30	1.32E-29	Venom carboxylesterase-6; similar with EST6_APIME
Px000371.1	3.0476	0.00E+00	0.00E+00	Lipase 1; similar with LIP1_DROME
Px012695.1	2.9423	3.93E-150	9.92E-149	Lipase 1; similar with LIP1_DROME
Px011683.2	2.9306	0.00E+00	0.00E+00	Venom carboxylesterase-6; similar with EST6_APIME
Px001527.1	2.7935	7.50E-09	3.13E-08	Lipase 1; similar with LIP1_DROME
Px017996.1	2.7512	1.00E-177	2.87E-176	Acetylcholinesterase; similar with ACES_HUMAN
Px000089.1	2.6887	2.89E-87	4.95E-86	Acetylcholinesterase; similar with ACES_FELCA
Px002735.1	2.6010	1.66E-257	6.15E-256	Esterase FE4; similar with ESTF_MYZPE
Px003400.1	2.5938	6.18E-17	3.82E-16	EF-hand calcium-binding domain-containing protein 1
Px009691.1	2.5676	5.36E-19	3.56E-18	Lipase 1; similar with LIP1_DROME
Px011049.1	2.5090	4.88E-35	4.62E-34	Acetylcholinesterase; similar with ACES_FELCA
Px002736.1	2.4868	0.00E+00	0.00E+00	Venom carboxylesterase-6; similar with EST6_APIME
Px001913.1	2.4737	1.20E-150	3.03E-149	Gastric triacylglycerol lipase; similar with LIPG_RAT
Px006430.1	2.4038	1.48E-302	6.22E-301	Esterase FE4; similar with ESTF_MYZPE
Px011475.1	2.3683	0.00E+00	0.00E+00	Lipase 3; similar with LIP3_DROME
Px012592.1	2.2775	0.00E+00	0.00E+00	Juvenile hormone esterase; similar with ESTJ_HELVI
Px001456.1	2.2249	2.79E-169	7.74E-168	Gastric triacylglycerol lipase; similar with LIPG_RAT
Px003649.1	2.1363	0.00E+00	0.00E+00	Esterase FE4; similar with ESTF_MYZPE
Px012696.1	2.0159	2.21E-135	5.07E-134	Lipase 3; similar with LIP3_DROME
Px000515.1	1.9774	1.03E-98	1.91E-97	Esterase FE4; similar with ESTF_MYZPE
Px016338.1	1.9089	9.52E-63	1.31E-61	Lipase 1; similar with LIP1_DROME
Px011755.1	1.7284	1.92E-39	1.96E-38	Antennal esterase CXE4; similar with D3GDL5_SPOLI
Px003712.1	1.6490	1.05E-19	7.17E-19	Acetylcholinesterase; similar with ACES_CULPI
Px017997.1	1.6216	3.59E-04	9.62E-04	Esterase FE4; similar with ESTF_MYZPE
Px007772.1	1.5076	2.80E-66	3.96E-65	Esterase B1; similar with EST1_CULPI
Px016066.1	1.4923	1.12E-13	6.06E-13	Lipase member M; similar with LIPM_MOUSE
Px011757.1	1.0401	5.69E-11	2.72E-10	Esterase FE4; similar with ESTF_MYZPE
Px012289.1	-1.0288	3.16E-06	1.07E-05	Liver carboxylesterase; similar with EST1_MESAU

Involved in complement and coagulation cascades

Px015905.1	13.4712	1.77E-71	2.63E-70	Transmembrane protease serine 6; similar with TMPS6_MOUSE
Px003000.1	12.5364	2.28E-42	2.43E-41	Transmembrane protease serine 6; similar with TMPS6_MOUSE
Px006453.1	10.3391	8.15E-10	3.63E-09	Transmembrane protease serine 6; similar with TMPS6_MOUSE
Px006790.1	5.8207	8.90E-106	1.74E-104	Hemocytin; similar with HMCT_BOMMO
Px007357.2	5.3379	0.00E+00	0.00E+00	Serine proteinase stubble; similar with STUB_DROME
Px016100.1	4.5930	1.02E-46	1.15E-45	Coagulation factor IX; similar with FA9_RABIT
Px016549.2	4.5691	2.48E-12	1.26E-11	Glia-derived nexin; similar with GDN_MOUSE
Px009539.1	4.4251	0.00E+00	0.00E+00	Hemocytin; similar with HMCT_BOMMO
Px016053.1	4.2124	5.01E-59	6.53E-58	Trypsin; similar with TRYP_PHACE
Px006789.3	3.3612	0.00E+00	0.00E+00	Hemocytin; similar with HMCT_BOMMO

Px004168.1	3.2472	2.59E-13	1.38E-12	Plasma kallikrein; similar with KLKB1_HUMAN
Px001997.1	3.0350	2.53E-37	2.50E-36	Carboxypeptidase B; similar with CBPB_ASTFL
Px007757.1	2.7947	2.34E-29	2.01E-28	Putative trypsin Inhibitor like cysteine rich domain protein
Px018074.1	2.6056	3.91E-24	2.97E-23	Chymotrypsin-like elastase family member 2A
Px000584.1	2.1984	3.86E-96	7.02E-95	Coagulation factor X; similar with FA10_RAT
Px001833.1	2.0603	0.00E+00	0.00E+00	Serine protease easter; similar with EAST_DROME
Px004167.1	2.0469	1.15E-06	4.06E-06	Plasma kallikrein; similar with KLKB1_MOUSE
Px011577.1	1.8036	1.23E-82	2.03E-81	Plasminogen activator inhibitor 1; similar with PAI1_PIG
Px012329.1	1.7693	5.39E-84	8.95E-83	Serine protease persephone; similar with PSH_DROME
Px009087.2	1.6281	1.83E-240	6.53E-239	Coagulation factor XII; similar with FA12_CAVPO
Px001556.1	1.6061	8.94E-117	1.85E-115	Serine protease easter; similar with EAST_DROME
Px007271.1	1.5855	0.00E+00	0.00E+00	Serpin B9; similar with SPB9_HUMAN
Px003184.1	1.3818	6.82E-52	8.23E-51	Unknown functio
Px005947.1	1.3627	2.45E-06	8.39E-06	Serine proteinase stubble; similar with STUB_DROME
Px011101.1	1.3474	1.36E-56	1.73E-55	Coagulation factor X; similar with FA10_RAT
Px006975.2	1.3342	0.00E+00	0.00E+00	Serine protease gd; similar with GD_DROME
Px007273.1	1.2484	5.02E-34	4.68E-33	Plasminogen activator inhibitor 1; similar with PAI1_HUMAN
Px000103.2	1.2217	3.55E-133	8.06E-132	Prothrombin; similar with THRB_MOUSE
Px014830.1	1.1646	9.91E-167	2.71E-165	Tryptase; similar with TRYB1_MOUSE
Px003670.2	1.1636	0.00E+00	0.00E+00	Serine protease easter; similar with EAST_DROME
Px010253.1	1.1621	1.06E-107	2.11E-106	Unknown functio
Px016983.3	1.0379	6.64E-09	2.78E-08	Serine proteinase stubble; similar with STUB_DROME
Px005668.1	-2.2447	8.13E-05	2.38E-04	NGFI-A-binding protein homolog; similar with NAB_DROME
Px013792.1	-1.1047	6.26E-31	5.54E-30	Thrombospondin type-1 domain-containing protein 7A

Involved in alpha-linolenic acid metabolism

Px012560.1	10.5968	1.26E-07	4.83E-07	Lipase member H-B; similar with LIPHB_XENLA
Px004749.1	4.2513	0.00E+00	0.00E+00	Lipoprotein lipase; similar with LIPL_CAVPO
Px000948.1	4.2472	2.43E-05	7.55E-05	Gastric triacylglycerol lipase; similar with LIPG_RAT
Px012694.3	4.1304	9.29E-40	9.57E-39	Lipase 1; similar with LIP1_DROME
Px005805.1	3.8398	5.29E-43	5.67E-42	Lipase 1; similar with LIP1_DROME
Px005804.1	3.4363	2.24E-142	5.42E-141	Lipase 1; similar with LIP1_DROME
Px011477.2	3.1975	0.00E+00	0.00E+00	Lipase 1; similar with LIP1_DROME
Px001526.1	3.1070	4.54E-37	4.47E-36	Lipase 1; similar with LIP1_DROME
Px000371.1	3.0476	0.00E+00	0.00E+00	Lipase 1; similar with LIP1_DROME
Px012695.1	2.9423	3.93E-150	9.92E-149	Lipase 1; similar with LIP1_DROME
Px011788.2	2.8400	3.24E-222	1.09E-220	Pancreatic lipase-related protein 2; similar with LIPR2_BOVIN
Px001527.1	2.7935	7.50E-09	3.13E-08	Lipase 1; similar with LIP1_DROME
Px005193.2	2.5958	2.33E-141	5.61E-140	Pancreatic lipase-related protein 2; similar with LIPR2_MYOCO
Px009691.1	2.5676	5.36E-19	3.56E-18	Lipase 1; similar with LIP1_DROME
Px006345.1	2.5102	0.00E+00	0.00E+00	Pancreatic lipase-related protein 2; similar with LIPR2_MYOCO
Px004750.1	2.4896	0.00E+00	0.00E+00	Pancreatic lipase-related protein 2; similar with LIPR2_RAT
Px001913.1	2.4737	1.20E-150	3.03E-149	Gastric triacylglycerol lipase; similar with LIPG_RAT
Px011475.1	2.3683	0.00E+00	0.00E+00	Lipase 3; similar with LIP3_DROME
Px004752.1	2.3527	0.00E+00	0.00E+00	Pancreatic lipase-related protein 2; similar with LIPR2_BOVIN
Px004753.1	2.3410	9.82E-208	3.12E-206	Pancreatic lipase-related protein 2; similar with LIPR2_MYOCO
Px002296.1	2.1474	1.41E-169	3.92E-168	Pancreatic triacylglycerol lipase; similar with LIPP_HUMAN
Px003273.1	2.1129	6.40E-16	3.82E-15	Pancreatic lipase-related protein 2; similar with LIPR2_CAVPO
Px006500.1	2.0492	9.92E-42	1.05E-40	Peroxisomal acyl-coenzyme A oxidase 3
Px006763.1	2.0407	1.02E-05	3.30E-05	Peroxisomal acyl-coenzyme A oxidase 3
Px012696.1	2.0159	2.21E-135	5.07E-134	Lipase 3; similar with LIP3_DROME
Px004751.1	1.9293	0.00E+00	0.00E+00	Pancreatic triacylglycerol lipase; similar with LIPP_HUMAN
Px016338.1	1.9089	9.52E-63	1.31E-61	Lipase 1; similar with LIP1_DROME
Px002295.1	1.7684	0.00E+00	0.00E+00	Pancreatic lipase-related protein 2; similar with LIPR2_BOVIN

Px011476.1	1.7230	8.10E-19	5.34E-18	Lipase 3; similar with LIP3_DROME
Px001531.1	1.5708	7.84E-60	1.04E-58	Probable peroxisomal acyl-coenzyme A oxidase 1
Px007047.2	1.5134	9.55E-05	2.77E-04	Probable peroxisomal acyl-coenzyme A oxidase 1
Px014702.1	1.5035	1.75E-04	4.92E-04	Lysine-specific demethylase 8; similar with KDM8_RAT
Px016066.1	1.4923	1.12E-13	6.06E-13	Lipase member M; similar with LIPM_MOUSE
Px004131.5	1.1474	6.20E-26	4.92E-25	Calcium-independent phospholipase A2-gamma
Px007883.1	1.1153	4.95E-21	3.49E-20	Probable medium-chain specific acyl-CoA dehydrogenase, mitochondrial

Involved in glycerophospholipid metabolism

Px012560.1	10.5968	1.26E-07	4.83E-07	Lipase member H-B; similar with LIPHB_XENLA
Px005646.1	4.6071	1.01E-58	1.31E-57	Nose resistant to fluoxetine protein 6; similar with NRF6_CAEEL
Px004749.1	4.2513	0.00E+00	0.00E+00	Lipoprotein lipase; similar with LIPL_CAVPO
Px000948.1	4.2472	2.43E-05	7.55E-05	Gastric triacylglycerol lipase; similar with LIPG_RAT
Px012694.3	4.1304	9.29E-40	9.57E-39	Lipase 1; similar with LIP1_DROME
Px003528.3	3.9017	3.38E-20	2.32E-19	Nose resistant to fluoxetine protein 6; similar with NRF6_CAEEL
Px005805.1	3.8398	5.29E-43	5.67E-42	Lipase 1; similar with LIP1_DROME
Px002585.2	3.5836	3.76E-53	4.58E-52	Nose resistant to fluoxetine protein 6; similar with NRF6_CAEEL
Px005804.1	3.4363	2.24E-142	5.42E-141	Lipase 1; similar with LIP1_DROME
Px009940.2	3.4221	6.33E-52	7.64E-51	Acetylcholinesterase; similar with ACES_CULPI
Px009569.1	3.3179	0.00E+00	0.00E+00	Trypsin CFT-1; similar with TRYP_CHOFU
Px011477.2	3.1975	0.00E+00	0.00E+00	Lipase 1; similar with LIP1_DROME
Px003182.1	3.1217	2.62E-07	9.75E-07	Acyl-CoA:lysophosphatidylglycerol acyltransferase 1
Px001526.1	3.1070	4.54E-37	4.47E-36	Lipase 1; similar with LIP1_DROME
Px000371.1	3.0476	0.00E+00	0.00E+00	Lipase 1; similar with LIP1_DROME
Px012695.1	2.9423	3.93E-150	9.92E-149	Lipase 1; similar with LIP1_DROME
Px011788.2	2.8400	3.24E-222	1.09E-220	Pancreatic lipase-related protein 2; similar with LIPR2_BOVIN
Px005417.1	2.8142	7.10E-18	4.51E-17	Nose resistant to fluoxetine protein 6; similar with NRF6_CAEEL
Px001527.1	2.7935	7.50E-09	3.13E-08	Lipase 1; similar with LIP1_DROME
Px015388.1	2.7618	1.35E-08	5.50E-08	Nose resistant to fluoxetine protein 6; similar with NRF6_CAEEL
Px005418.1	2.6622	6.87E-07	2.48E-06	Nose resistant to fluoxetine protein 6; similar with NRF6_CAEEL
Px005193.2	2.5958	2.33E-141	5.61E-140	Pancreatic lipase-related protein 2; similar with LIPR2_MYOCO
Px009691.1	2.5676	5.36E-19	3.56E-18	Lipase 1; similar with LIP1_DROME
Px006345.1	2.5102	0.00E+00	0.00E+00	Pancreatic lipase-related protein 2; similar with LIPR2_MYOCO
Px011049.1	2.5090	4.88E-35	4.62E-34	Acetylcholinesterase; similar with ACES_FELCA
Px004750.1	2.4896	0.00E+00	0.00E+00	Pancreatic lipase-related protein 2; similar with LIPR2_RAT
Px001913.1	2.4737	1.20E-150	3.03E-149	Gastric triacylglycerol lipase; similar with LIPG_RAT
Px011475.1	2.3683	0.00E+00	0.00E+00	Lipase 3; similar with LIP3_DROME
Px004752.1	2.3527	0.00E+00	0.00E+00	Pancreatic lipase-related protein 2; similar with LIPR2_BOVIN
Px004753.1	2.3410	9.82E-208	3.12E-206	Pancreatic lipase-related protein 2; similar with LIPR2_MYOCO
Px008913.1	2.1926	0.00E+00	0.00E+00	Acetylcholinesterase; similar with ACES_CULPI
Px002296.1	2.1474	1.41E-169	3.92E-168	Pancreatic triacylglycerol lipase; similar with LIPP_HUMAN
Px002964.1	2.1362	2.49E-07	9.29E-07	Putative uncharacterized protein; similar with E9H4D7_DAPPU
Px003273.1	2.1129	6.40E-16	3.82E-15	Pancreatic lipase-related protein 2; similar with LIPR2_CAVPO
Px004200.1	2.0610	1.11E-81	1.81E-80	Putative phosphatidate phosphatase; similar with WUN_DROME
Px012696.1	2.0159	2.21E-135	5.07E-134	Lipase 3; similar with LIP3_DROME
Px004751.1	1.9293	0.00E+00	0.00E+00	Pancreatic triacylglycerol lipase; similar with LIPP_HUMAN
Px016338.1	1.9089	9.52E-63	1.31E-61	Lipase 1; similar with LIP1_DROME
Px012937.2	1.8846	1.37E-05	4.37E-05	Phospholipase D1; similar with PLD1_HUMAN
Px002295.1	1.7684	0.00E+00	0.00E+00	Pancreatic lipase-related protein 2; similar with LIPR2_BOVIN
Px011476.1	1.7230	8.10E-19	5.34E-18	Lipase 3; similar with LIP3_DROME
Px013101.1	1.6622	3.21E-06	1.09E-05	Nose resistant to fluoxetine protein 6; similar with NRF6_CAEEL
Px003712.1	1.6490	1.05E-19	7.17E-19	Acetylcholinesterase; similar with ACES_CULPI
Px017997.1	1.6216	3.59E-04	9.62E-04	Esterase FE4; similar with ESTF_MYZPE
Px011624.1	1.5666	4.24E-07	1.55E-06	Nose resistant to fluoxetine protein 6; similar with NRF6_CAEEL

Px014702.1	1.5035	1.75E-04	4.92E-04	Lysine-specific demethylase 8; similar with KDM8_RAT
Px016066.1	1.4923	1.12E-13	6.06E-13	Lipase member M; similar with LIPM_MOUSE
Px006720.1	1.4070	1.32E-07	5.02E-07	Glycerol-3-phosphate acyltransferase 4
Px017196.1	1.3834	3.80E-22	2.75E-21	Choline/ethanolamine kinase; similar with CHKB_HUMAN
Px014079.1	1.2713	4.69E-10	2.13E-09	Nose resistant to fluoxetine protein 6; similar with NRF6_CAEEL
Px001937.2	1.2673	3.55E-115	7.31E-114	Phosphatidate phosphatase LPIN2; similar with LPIN2_MOUSE
Px006231.1	1.2299	8.55E-52	1.03E-50	Protein B602L; similar with VF602_ASFB7
Px007406.1	1.2241	1.13E-04	3.24E-04	Transmembrane protein nessy; similar with NESSY_DROME
Px001894.8	1.1880	5.51E-25	4.27E-24	Ethanolamine-phosphate cytidylyltransferase
Px004131.5	1.1474	6.20E-26	4.92E-25	Calcium-independent phospholipase A2-gamma
Px010099.1	1.1453	2.76E-19	1.85E-18	Phospholipase D1; similar with PLD1_RAT
Px011234.1	1.0051	2.16E-04	5.97E-04	Nose resistant to fluoxetine protein 6; similar with NRF6_CAEEL

Involved in steroid hormone biosynthesis

Px009293.1	11.1045	9.55E-06	3.10E-05	Probable cytochrome P450 304a1; similar with CP304_DROME
Px009572.1	9.2901	5.34E-07	1.94E-06	Cytochrome P450 6B1; similar with CP6B1_PAPPO
Px000114.1	3.5971	6.55E-42	6.92E-41	Sulfotransferase 1C4; similar with ST1C4_HUMAN
Px002723.1	2.9848	0.00E+00	0.00E+00	Cytochrome P450 6B7; similar with CP6B7_HELAM
Px015642.1	2.8052	4.74E-10	2.15E-09	Estradiol 17-beta-dehydrogenase 8; similar with DHB8_CANFA
Px014883.1	2.5298	7.38E-32	6.64E-31	Ecdysteroid UDP-glucosyltransferase; similar with UDPE_NPVSL
Px013884.1	2.4758	5.37E-18	3.43E-17	Probable cytochrome P450 9f2; similar with CP9F2_DROME
Px014981.1	2.4259	0.00E+00	0.00E+00	Cytochrome P450 9e2; similar with CP9E2_BLAGE
Px005902.4	2.1318	2.80E-119	5.89E-118	Cytochrome P450 6B6; similar with CP6B6_HELAM
Px004854.1	2.1011	1.06E-15	6.27E-15	Ecdysteroid UDP-glucosyltransferase; similar with UDPE_NPVSL
Px014216.1	1.9094	1.32E-40	1.38E-39	Cytochrome P450 6B6; similar with CP6B6_HELAM
Px000872.2	1.8756	1.82E-09	7.93E-09	Ecdysteroid UDP-glucosyltransferase; similar with UDPE_NPVSL
Px000647.3	1.8232	6.21E-280	2.49E-278	UDP-glucuronosyltransferase 2B7; similar with UD2B7_HUMAN
Px011727.1	1.7734	6.73E-64	9.35E-63	Protein GCY; similar with GCY_YEAST
Px001113.1	1.6873	1.59E-11	7.83E-11	Ecdysteroid UDP-glucosyltransferase; similar with UDPE_NPVSL
Px004643.1	1.5882	6.58E-06	2.17E-05	Cytochrome P450 6B6; similar with CP6B6_HELAM
Px013958.1	1.5211	6.83E-145	1.68E-143	Cytochrome P450 6k1; similar with CP6K1_BLAGE
Px014982.1	1.4635	0.00E+00	0.00E+00	Probable cytochrome P450 9f2; similar with CP9F2_DROME
Px013203.1	1.4517	2.86E-06	9.77E-06	UDP-glucuronosyltransferase 2B2; similar with UD2B2_RAT
Px006292.1	1.2679	2.20E-13	1.18E-12	Ecdysteroid UDP-glucosyltransferase; similar with UDPE_NPVSL
Px017253.1	1.2169	4.53E-37	4.47E-36	Cytochrome P450 6B2; similar with CP6B2_HELAM
Px000039.1	1.1965	1.40E-231	4.93E-230	UDP-glucuronosyltransferase 1-8; similar with UD18_RAT
Px001164.1	1.1390	6.83E-117	1.42E-115	UDP-glucuronosyltransferase; similar with UGT3_PLEPL
Px009675.1	1.1334	1.86E-96	3.40E-95	UDP-glucuronosyltransferase 1-2; similar with UD12_MOUSE
Px015080.1	1.1199	3.36E-06	1.14E-05	Ecdysteroid UDP-glucosyltransferase; similar with UDPE_NPVMB
Px005900.1	1.0773	1.30E-16	7.94E-16	Cytochrome P450 6B2; similar with CP6B2_HELAM
Px011285.1	1.0624	2.26E-250	8.22E-249	UDP-glucuronosyltransferase 2B4; similar with UD2B4_HUMAN

Involved in PPAR signaling pathway

Px004749.1	4.2513	0.00E+00	0.00E+00	Lipoprotein lipase; similar with LIPL_CAVPO
Px008783.1	3.5367	1.08E-05	3.49E-05	Long-chain fatty acid transport protein 1
Px008088.2	2.8173	0.00E+00	0.00E+00	Lipoprotein lipase; similar with LIPL_CAVPO
Px017039.1	2.6501	0.00E+00	0.00E+00	Putative acyl-CoA-binding protein; similar with ACBP_HYPDU
Px005193.2	2.5958	2.33E-141	5.61E-140	Pancreatic lipase-related protein 2; similar with LIPR2_MYOCO
Px005199.1	2.5327	1.44E-159	3.81E-158	Long-chain fatty acid transport protein 4
Px004750.1	2.4896	0.00E+00	0.00E+00	Pancreatic lipase-related protein 2; similar with LIPR2_RAT
Px004752.1	2.3527	0.00E+00	0.00E+00	Pancreatic lipase-related protein 2; similar with LIPR2_BOVIN
Px004753.1	2.3410	9.82E-208	3.12E-206	Pancreatic lipase-related protein 2; similar with LIPR2_MYOCO
Px006441.1	2.3113	0.00E+00	0.00E+00	Lipase member H; similar with LIPH_RAT
Px001337.1	2.2940	0.00E+00	0.00E+00	Putative uncharacterized protein; similar with A9NYL2_PICSI

Px007360.3	2.2294	2.18E-223	7.44E-222	3-phosphoinositide-dependent protein kinase 1
Px000744.1	2.0773	1.67E-04	4.72E-04	Lipase member H-A; similar with LIPHA_XENLA
Px006440.1	2.0668	1.56E-48	1.81E-47	Phospholipase A1 member A; similar with PLA1A_HUMAN
Px006500.1	2.0492	9.92E-42	1.05E-40	Peroxisomal acyl-coenzyme A oxidase 3
Px006763.1	2.0407	1.02E-05	3.30E-05	Peroxisomal acyl-coenzyme A oxidase 3
Px004751.1	1.9293	0.00E+00	0.00E+00	Pancreatic triacylglycerol lipase; similar with LIPP_HUMAN
Px015380.1	1.9245	1.43E-39	1.47E-38	Acyl-CoA Delta(11) desaturase; similar with ACO11_TRINI
Px002295.1	1.7684	0.00E+00	0.00E+00	Pancreatic lipase-related protein 2; similar with LIPR2_BOVIN
Px000844.1	1.7136	3.72E-14	2.07E-13	Ribosomal protein S6 kinase delta-1; similar with KS6C1_HUMAN
Px001605.1	1.5936	1.14E-246	4.14E-245	Putative acyl-CoA-binding protein; similar with ACBP_HYPDU
Px000132.1	1.5866	3.04E-79	4.87E-78	FGGY carbohydrate kinase domain-containing protein
Px011340.1	1.5860	0.00E+00	0.00E+00	Non-specific lipid-transfer protein; similar with NLTP_CHICK
Px001531.1	1.5708	7.84E-60	1.04E-58	Probable peroxisomal acyl-coenzyme A oxidase 1
Px000524.1	1.5210	2.27E-74	3.48E-73	Putative acyl-CoA-binding protein; similar with ACBP_HYPDU
Px007047.2	1.5134	9.55E-05	2.77E-04	Probable peroxisomal acyl-coenzyme A oxidase 1
Px016733.1	1.2758	1.82E-09	7.92E-09	Acyl-CoA synthetase family member 3, mitochondrial
Px013837.1	1.2663	1.45E-04	4.10E-04	Carnitine O-palmitoyltransferase 1, muscle isoform
Px010887.1	1.1902	3.16E-21	2.24E-20	Phosphoenolpyruvate carboxykinase [GTP]
Px015376.1	1.1200	1.05E-146	2.61E-145	Phosphoenolpyruvate carboxykinase [GTP]
Px007883.1	1.1153	4.95E-21	3.49E-20	Probable medium-chain specific acyl-CoA dehydrogenase, mitochondrial
Px008080.1	1.0773	1.70E-05	5.37E-05	Lysosome membrane protein 2; similar with SCRB2_HUMAN
Px016558.1	1.0773	3.20E-04	8.66E-04	Putative glycerol kinase 5; similar with GLPK5_DANRE
Px013239.1	1.0562	3.46E-07	1.28E-06	Putative glycerol kinase 3; similar with GLPK3_HUMAN
Px005198.1	1.0445	2.21E-21	1.58E-20	Long-chain fatty acid transport protein 4
Px008837.1	1.0217	1.55E-94	2.77E-93	Long-chain-fatty-acid--CoA ligase ACSBG2
Px013405.1	-1.5636	1.01E-18	6.66E-18	CAP isoform A; similar with C9DTM4_BOMMO
Px017708.4	-1.4493	1.01E-40	1.05E-39	Long-chain-fatty-acid--CoA ligase 1; similar with ACSL1_RAT

Involved in vitamin digestion and absorption

Px000644.1	12.4260	4.78E-37	4.70E-36	Pancreatic triacylglycerol lipase; similar with LIPP_MOUSE
Px012560.1	10.5968	1.26E-07	4.83E-07	Lipase member H-B; similar with LIPHB_XENLA
Px009713.1	7.3544	7.08E-09	2.96E-08	Cubilin; similar with CUBN_CANFA
Px011791.1	4.1145	0.00E+00	0.00E+00	Lipase member H; similar with LIPH_XENTR
Px012011.2	4.0620	0.00E+00	0.00E+00	Pancreatic triacylglycerol lipase; similar with LIPP_MYOCO
Px014012.2	3.4448	3.32E-59	4.34E-58	Proton-coupled folate transporter; similar with PCFT_XENLA
Px008089.1	2.8762	0.00E+00	0.00E+00	Pancreatic lipase-related protein 2; similar with LIPR2_CAVPO
Px006190.1	2.8647	0.00E+00	0.00E+00	Lipase member H-A; similar with LIPHA_XENLA
Px011788.2	2.8400	3.24E-222	1.09E-220	Pancreatic lipase-related protein 2; similar with LIPR2_BOVIN
Px012537.1	2.7402	3.03E-04	8.22E-04	Apolipophorins; similar with APLP_LOCMI
Px013824.1	2.4844	6.34E-19	4.20E-18	Sensory neuron membrane protein 1; similar with SNMP1_PEDHC
Px004752.1	2.3527	0.00E+00	0.00E+00	Pancreatic lipase-related protein 2; similar with LIPR2_BOVIN
Px006441.1	2.3113	0.00E+00	0.00E+00	Lipase member H; similar with LIPH_RAT
Px009761.1	2.2693	1.37E-127	3.02E-126	Plasma glutamate carboxypeptidase; similar with PGCP_XENLA
Px002296.1	2.1474	1.41E-169	3.92E-168	Pancreatic triacylglycerol lipase; similar with LIPP_HUMAN
Px003273.1	2.1129	6.40E-16	3.82E-15	Pancreatic lipase-related protein 2; similar with LIPR2_CAVPO
Px004751.1	1.9293	0.00E+00	0.00E+00	Pancreatic triacylglycerol lipase; similar with LIPP_HUMAN
Px008150.1	1.7972	2.12E-17	1.33E-16	Scavenger receptor class B member 1; similar with SCRB1_PIG
Px001510.4	1.7749	2.77E-85	4.66E-84	Scavenger receptor class B member 1; similar with SCRB1_PIG
Px002295.1	1.7684	0.00E+00	0.00E+00	Pancreatic lipase-related protein 2; similar with LIPR2_BOVIN
Px011335.1	1.7641	1.16E-13	6.27E-13	Scavenger receptor class B member 1; similar with SCRB1_BOVIN
Px007238.1	1.7626	1.98E-23	1.48E-22	Solute carrier family 46 member 3; similar with S46A3_CHICK
Px011641.3	1.7499	0.00E+00	0.00E+00	Vanin-like protein 2; similar with VNNL2_DROME
Px012163.2	1.7447	5.09E-26	4.05E-25	Protein croquemort; similar with CRQ_DROME
Px015615.1	1.7053	2.16E-07	8.10E-07	Proton-coupled folate transporter; similar with PCFT_DANRE

Px002809.2	1.5031	7.52E-18	4.78E-17	WD repeat domain phosphoinositide-interacting protein 4
Px011529.1	1.4501	6.50E-117	1.35E-115	Apolipophorins; similar with APLP_LOCMI
Px014014.2	1.2587	6.46E-21	4.55E-20	Proton-coupled folate transporter; similar with PCFT_DANRE
Px014013.2	1.2197	1.13E-07	4.34E-07	Proton-coupled folate transporter; similar with PCFT_DANRE
Px014019.1	1.1874	2.13E-08	8.57E-08	Solute carrier family 46 member 3; similar with S46A3_CHICK
Px015324.1	1.1450	2.99E-40	3.10E-39	Proton-coupled folate transporter; similar with PCFT_XENLA
Px012098.2	1.0475	2.15E-05	6.71E-05	Lipase member I; similar with LIPI_HUMAN
Px009302.2	-1.5524	1.01E-125	2.21E-124	Putative uncharacterized protein; similar with D7GYL0_TRICA
Px006184.1	-1.5077	3.41E-07	1.26E-06	Neuropilin and tolloid-like protein 1
Px014011.1	-1.2447	1.09E-04	3.13E-04	Proton-coupled folate transporter; similar with PCFT_XENLA
Px016584.2	-1.1902	1.13E-07	4.34E-07	Solute carrier family 23 member 2; similar with S23A2_XENTR

Involved in retinol metabolism

Px009293.1	11.1045	9.55E-06	3.10E-05	Probable cytochrome P450 304a1; similar with CP304_DROME
Px009572.1	9.2901	5.34E-07	1.94E-06	Cytochrome P450 6B1; similar with CP6B1_PAPPO
Px002723.1	2.9848	0.00E+00	0.00E+00	Cytochrome P450 6B7; similar with CP6B7_HELAM
Px006696.1	2.6893	0.00E+00	0.00E+00	Short-chain dehydrogenase/reductase family 16C member 6
Px014883.1	2.5298	7.38E-32	6.64E-31	Ecdysteroid UDP-glucosyltransferase; similar with UDPE_NPVSL
Px013884.1	2.4758	5.37E-18	3.43E-17	Probable cytochrome P450 9f2; similar with CP9F2_DROME
Px014981.1	2.4259	0.00E+00	0.00E+00	Cytochrome P450 9e2; similar with CP9E2_BLAGE
Px006319.1	2.1734	2.37E-82	3.89E-81	Retinol dehydrogenase 11; similar with RDH11_HUMAN
Px005902.4	2.1318	2.80E-119	5.89E-118	Cytochrome P450 6B6; similar with CP6B6_HELAM
Px004854.1	2.1011	1.06E-15	6.27E-15	Ecdysteroid UDP-glucosyltransferase; similar with UDPE_NPVSL
Px014216.1	1.9094	1.32E-40	1.38E-39	Cytochrome P450 6B6; similar with CP6B6_HELAM
Px000872.2	1.8756	1.82E-09	7.93E-09	Ecdysteroid UDP-glucosyltransferase; similar with UDPE_NPVSL
Px010853.1	1.8586	7.49E-18	4.76E-17	Retinol dehydrogenase 11; similar with RDH11_MOUSE
Px000647.3	1.8232	6.21E-280	2.49E-278	UDP-glucuronosyltransferase 2B7; similar with UD2B7_HUMAN
Px005070.1	1.7143	1.14E-73	1.74E-72	Glucose 1-dehydrogenase 2; similar with DHG2_BACME
Px001113.1	1.6873	1.59E-11	7.83E-11	Ecdysteroid UDP-glucosyltransferase; similar with UDPE_NPVSL
Px004643.1	1.5882	6.58E-06	2.17E-05	Cytochrome P450 6B6; similar with CP6B6_HELAM
Px008706.2	1.5512	3.40E-05	1.04E-04	WW domain-containing oxidoreductase
Px013958.1	1.5211	6.83E-145	1.68E-143	Cytochrome P450 6k1; similar with CP6K1_BLAGE
Px014982.1	1.4635	0.00E+00	0.00E+00	Probable cytochrome P450 9f2; similar with CP9F2_DROME
Px013203.1	1.4517	2.86E-06	9.77E-06	UDP-glucuronosyltransferase 2B2; similar with UD2B2_RAT
Px006694.1	1.3215	6.61E-31	5.85E-30	Epidermal retinol dehydrogenase 2; similar with RDHE2_MOUSE
Px006292.1	1.2679	2.20E-13	1.18E-12	Ecdysteroid UDP-glucosyltransferase; similar with UDPE_NPVSL
Px017253.1	1.2169	4.53E-37	4.47E-36	Cytochrome P450 6B2; similar with CP6B2_HELAM
Px000039.1	1.1965	1.40E-231	4.93E-230	UDP-glucuronosyltransferase 1-8; similar with UD18_RAT
Px013246.1	1.1587	3.16E-98	5.83E-97	Dehydrogenase/reductase SDR family member 4
Px001164.1	1.1390	6.83E-117	1.42E-115	UDP-glucuronosyltransferase; similar with UGT3_PLEPL
Px009675.1	1.1334	1.86E-96	3.40E-95	UDP-glucuronosyltransferase 1-2; similar with UD12_MOUSE
Px015080.1	1.1199	3.36E-06	1.14E-05	Ecdysteroid UDP-glucosyltransferase; similar with UDPE_NPVMB
Px005900.1	1.0773	1.30E-16	7.94E-16	Cytochrome P450 6B2; similar with CP6B2_HELAM
Px011285.1	1.0624	2.26E-250	8.22E-249	UDP-glucuronosyltransferase 2B4; similar with UD2B4_HUMAN
Px015569.1	1.0487	2.19E-22	1.60E-21	3-oxoacyl-[acyl-carrier-protein] reductase FabG
Px010797.1	1.0393	1.27E-07	4.84E-07	Epidermal retinol dehydrogenase 2; similar with RDHE2_MOUSE
Px007377.1	-1.9772	8.53E-07	3.05E-06	Glucose 1-dehydrogenase 1; similar with DHG1_BACME

Involved in drug metabolism - other enzymes

Px009572.1	9.2901	5.34E-07	1.94E-06	Cytochrome P450 6B1; similar with CP6B1_PAPPO
Px014859.2	3.9352	1.13E-07	4.34E-07	Uridine phosphorylase 1; similar with UPP1_MOUSE
Px004815.1	3.9017	3.38E-20	2.32E-19	Gut esterase 1; similar with EST1_CAEEL
Px009019.1	3.7997	1.15E-15	6.76E-15	Uridine phosphorylase 1; similar with UPP1_MOUSE
Px005119.1	3.7777	1.08E-09	4.79E-09	Retrovirus-related Pol polyprotein from transposon TNT 1-94

Px003352.1	3.1927	1.50E-30	1.32E-29	Venom carboxylesterase-6; similar with EST6_APIME
Px002723.1	2.9848	0.00E+00	0.00E+00	Cytochrome P450 6B7; similar with CP6B7_HELAM
Px017996.1	2.7512	1.00E-177	2.87E-176	Acetylcholinesterase; similar with ACES_HUMAN
Px000089.1	2.6887	2.89E-87	4.95E-86	Acetylcholinesterase; similar with ACES_FELCA
Px002735.1	2.6010	1.66E-257	6.15E-256	Esterase FE4; similar with ESTF_MYZPE
Px014883.1	2.5298	7.38E-32	6.64E-31	Ecdysteroid UDP-glucosyltransferase; similar with UDPE_NPVSL
Px011049.1	2.5090	4.88E-35	4.62E-34	Acetylcholinesterase; similar with ACES_FELCA
Px002736.1	2.4868	0.00E+00	0.00E+00	Venom carboxylesterase-6; similar with EST6_APIME
Px013884.1	2.4758	5.37E-18	3.43E-17	Probable cytochrome P450 9f2; similar with CP9F2_DROME
Px014981.1	2.4259	0.00E+00	0.00E+00	Cytochrome P450 9e2; similar with CP9E2_BLAGE
Px006430.1	2.4038	1.48E-302	6.22E-301	Esterase FE4; similar with ESTF_MYZPE
Px005687.1	2.2408	8.49E-30	7.38E-29	Venom carboxylesterase-6; similar with EST6_APIME
Px003649.1	2.1363	0.00E+00	0.00E+00	Esterase FE4; similar with ESTF_MYZPE
Px005902.4	2.1318	2.80E-119	5.89E-118	Cytochrome P450 6B6; similar with CP6B6_HELAM
Px004854.1	2.1011	1.06E-15	6.27E-15	Ecdysteroid UDP-glucosyltransferase; similar with UDPE_NPVSL
Px000515.1	1.9774	1.03E-98	1.91E-97	Esterase FE4; similar with ESTF_MYZPE
Px014216.1	1.9094	1.32E-40	1.38E-39	Cytochrome P450 6B6; similar with CP6B6_HELAM
Px000872.2	1.8756	1.82E-09	7.93E-09	Ecdysteroid UDP-glucosyltransferase; similar with UDPE_NPVSL
Px000647.3	1.8232	6.21E-280	2.49E-278	UDP-glucuronosyltransferase 2B7; similar with UD2B7_HUMAN
Px001113.1	1.6873	1.59E-11	7.83E-11	Ecdysteroid UDP-glucosyltransferase; similar with UDPE_NPVSL
Px017997.1	1.6216	3.59E-04	9.62E-04	Esterase FE4; similar with ESTF_MYZPE
Px004643.1	1.5882	6.58E-06	2.17E-05	Cytochrome P450 6B6; similar with CP6B6_HELAM
Px013958.1	1.5211	6.83E-145	1.68E-143	Cytochrome P450 6k1; similar with CP6K1_BLAGE
Px007772.1	1.5076	2.80E-66	3.96E-65	Esterase B1; similar with EST1_CULPI
Px014982.1	1.4635	0.00E+00	0.00E+00	Probable cytochrome P450 9f2; similar with CP9F2_DROME
Px013203.1	1.4517	2.86E-06	9.77E-06	UDP-glucuronosyltransferase 2B2; similar with UD2B2_RAT
Px006292.1	1.2679	2.20E-13	1.18E-12	Ecdysteroid UDP-glucosyltransferase; similar with UDPE_NPVSL
Px010076.1	1.2570	6.78E-14	3.72E-13	Carboxyl/choline esterase CCE014a; similar with D5G3E6_HELAM
Px017253.1	1.2169	4.53E-37	4.47E-36	Cytochrome P450 6B2; similar with CP6B2_HELAM
Px000039.1	1.1965	1.40E-231	4.93E-230	UDP-glucuronosyltransferase 1-8; similar with UD18_RAT
Px001164.1	1.1390	6.83E-117	1.42E-115	UDP-glucuronosyltransferase; similar with UGT3_PLEPL
Px009675.1	1.1334	1.86E-96	3.40E-95	UDP-glucuronosyltransferase 1-2; similar with UD12_MOUSE
Px015080.1	1.1199	3.36E-06	1.14E-05	Ecdysteroid UDP-glucosyltransferase; similar with UDPE_NPVMB
Px005900.1	1.0773	1.30E-16	7.94E-16	Cytochrome P450 6B2; similar with CP6B2_HELAM
Px011285.1	1.0624	2.26E-250	8.22E-249	UDP-glucuronosyltransferase 2B4; similar with UD2B4_HUMAN
Px013693.1	1.0094	5.72E-08	2.23E-07	Aldehyde oxidase; similar with ADO_HUMAN
Px012289.1	-1.0288	3.16E-06	1.07E-05	Liver carboxylesterase; similar with EST1_MESAU

Involved in metabolic pathways

Px000644.1	12.4260	4.78E-37	4.70E-36	Pancreatic triacylglycerol lipase; similar with LIPP_MOUSE
Px009293.1	11.1045	9.55E-06	3.10E-05	Probable cytochrome P450 304a1; similar with CP304_DROME
Px003417.1	11.0707	4.48E-16	2.69E-15	Beta-1,3-galactosyltransferase 5; similar with B3GT5_MOUSE
Px006067.1	10.6233	1.24E-12	6.43E-12	Nucleoside diphosphate kinase 7; similar with NDK7_RAT
Px012560.1	10.5968	1.26E-07	4.83E-07	Lipase member H-B; similar with LIPHB_XENLA
Px013527.1	10.4804	7.08E-09	2.96E-08	Carbonyl reductase [NADPH] 1; similar with CBR1_RABIT
Px011676.1	9.9119	4.04E-05	1.22E-04	Alpha-aminoadipic semialdehyde synthase, mitochondrial
Px002969.1	9.6359	1.96E-05	6.16E-05	Adenylate kinase isoenzyme 1; similar with KAD1_RAT
Px009572.1	9.2901	5.34E-07	1.94E-06	Cytochrome P450 6B1; similar with CP6B1_PAPPO
Px002197.1	9.2124	9.55E-06	3.10E-05	Putative malate dehydrogenase 1B; similar with MDH1B_BOVIN
Px013761.3	9.0724	2.99E-08	1.19E-07	Peroxidase; similar with PERO_DROME
Px014880.1	8.9447	4.64E-06	1.55E-05	ATP synthase subunit beta, mitochondrial; similar with ATPB_RAT
Px010354.2	8.4829	6.15E-08	2.39E-07	Envelope fusion protein; similar with ENV_NPVLD
Px015148.1	8.0644	1.96E-05	6.16E-05	ATP synthase subunit beta, mitochondrial; similar with ATPB_RAT
Px010126.1	7.3719	5.77E-48	6.66E-47	DNA-directed RNA polymerase II subunit RPB1

Px000336.1	6.9841	2.20E-107	4.36E-106	Osiris 20; similar with B6DXA7_BOMMO
Px000337.1	6.0185	2.92E-157	7.59E-156	Osiris 19; similar with B6DXA8_BOMMO
Px001707.1	5.4057	1.10E-120	2.34E-119	Membrane alanyl aminopeptidase; similar with AMPM_MANSE
Px007930.1	5.2867	5.24E-11	2.51E-10	Cytochrome c oxidase subunit 4 isoform 2, mitochondrial
Px002027.1	4.7961	1.66E-41	1.74E-40	L-sorbose 1-dehydrogenase; similar with SDH_GLUOX
Px008276.1	4.6077	0.00E+00	0.00E+00	Membrane alanyl aminopeptidase; similar with AMPM_HELVI
Px014796.1	4.3440	9.84E-20	6.71E-19	Apyrase; similar with APY_AEDAE
Px000024.1	4.3252	1.24E-05	3.97E-05	Cuticular protein CPG4; similar with B2DBL4_9NEOP
Px000084.1	4.3183	5.99E-51	7.13E-50	Cysteine sulfinic acid decarboxylase; similar with CSAD_HUMAN
Px004749.1	4.2513	0.00E+00	0.00E+00	Lipoprotein lipase; similar with LIPL_CAVPO
Px000948.1	4.2472	2.43E-05	7.55E-05	Gastric triacylglycerol lipase; similar with LIPG_RAT
Px000702.1	4.1858	2.08E-17	1.30E-16	NADH dehydrogenase [ubiquinone] iron-sulfur protein 2, mitochondrial
Px012694.3	4.1304	9.29E-40	9.57E-39	Lipase 1; similar with LIP1_DROME
Px011791.1	4.1145	0.00E+00	0.00E+00	Lipase member H; similar with LIPH_XENTR
Px000291.1	4.0996	2.97E-16	1.79E-15	Myrosinase 1; similar with MYRO1_BREBR
Px007386.1	4.0773	1.56E-08	6.32E-08	Carbonyl reductase [NADPH] 1; similar with CBR1_PIG
Px003596.1	4.0773	9.29E-05	2.70E-04	Predicted protein; similar with A9V0D7_MONBE
Px012011.2	4.0620	0.00E+00	0.00E+00	Pancreatic triacylglycerol lipase; similar with LIPP_MYOCO
Px000359.2	4.0159	3.40E-22	2.47E-21	Alpha-methyldopa hypersensitive protein
Px004490.1	3.9557	0.00E+00	0.00E+00	Adenosine deaminase CECR1; similar with CECR1_XENLA
Px014859.2	3.9352	1.13E-07	4.34E-07	Uridine phosphorylase 1; similar with UPP1_MOUSE
Px004815.1	3.9017	3.38E-20	2.32E-19	Gut esterase 1; similar with EST1_CAEEL
Px007180.1	3.8846	2.52E-32	2.29E-31	Juvenile hormone esterase; similar with ESTJ_HELVI
Px005805.1	3.8398	5.29E-43	5.67E-42	Lipase 1; similar with LIP1_DROME
Px003323.1	3.8321	4.22E-07	1.55E-06	LINE-1 reverse transcriptase homolog; similar with LIN1_NYCCO
Px009019.1	3.7997	1.15E-15	6.76E-15	Uridine phosphorylase 1; similar with UPP1_MOUSE
Px005119.1	3.7777	1.08E-09	4.79E-09	Retrovirus-related Pol polyprotein from transposon TNT 1-94
Px002634.1	3.7211	1.56E-06	5.44E-06	Beta-1,4-N-acetylgalactosaminyltransferase bre-4
Px000708.1	3.6813	1.01E-65	1.42E-64	Pyruvate kinase; similar with KPYK_DROME
Px016942.1	3.6242	1.71E-40	1.77E-39	NADH dehydrogenase [ubiquinone] 1 beta subcomplex subunit 9
Px011202.1	3.5882	8.25E-143	2.01E-141	Aromatic-L-amino-acid decarboxylase; similar with DDC_MANSE
Px000982.1	3.5851	4.17E-39	4.24E-38	Catalase; similar with CATA_GLARU
Px014253.1	3.5691	1.39E-10	6.48E-10	Unknown functio
Px005431.1	3.5627	7.25E-13	3.80E-12	ATP synthase E chain; similar with C9W1E1_RHISA
Px008598.1	3.5627	7.25E-13	3.80E-12	Phosphatidylinositol glycan anchor biosynthesis class U protein
Px005494.1	3.5545	9.06E-52	1.09E-50	Tubulin polyglutamylase TTLL6; similar with TTLL6_MOUSE
Px014693.1	3.5102	2.59E-12	1.32E-11	Nucleoside diphosphate kinase homolog 5
Px003371.1	3.4542	6.30E-39	6.39E-38	NADH dehydrogenase [ubiquinone] 1 alpha subcomplex subunit 8
Px005804.1	3.4363	2.24E-142	5.42E-141	Lipase 1; similar with LIP1_DROME
Px009940.2	3.4221	6.33E-52	7.64E-51	Acetylcholinesterase; similar with ACES_CULPI
Px006617.2	3.3534	1.05E-31	9.40E-31	Chorion peroxidase; similar with PERC_DROME
Px009020.1	3.3475	1.24E-134	2.82E-133	Protein C9orf138, putative; similar with E0VSG8_PEDHC
Px008277.1	3.3443	1.09E-141	2.62E-140	Membrane alanyl aminopeptidase; similar with AMPM_MANSE
Px007120.2	3.3099	1.50E-19	1.02E-18	ATP synthase subunit beta, mitochondrial
Px013989.1	3.2472	4.09E-08	1.62E-07	NADH dehydrogenase [ubiquinone] flavoprotein 2, mitochondrial
Px007471.1	3.2141	3.12E-82	5.11E-81	Pyruvate kinase; similar with KPYK_DROME
Px012208.1	3.1992	4.12E-29	3.54E-28	Probable flavin-containing monoamine oxidase A
Px011477.2	3.1975	0.00E+00	0.00E+00	Lipase 1; similar with LIP1_DROME
Px004605.1	3.1961	1.06E-135	2.45E-134	Sphingomyelin phosphodiesterase; similar with ASM_HUMAN
Px003352.1	3.1927	1.50E-30	1.32E-29	Venom carboxylesterase-6; similar with EST6_APIME
Px007137.2	3.1853	1.13E-132	2.54E-131	Peroxidase; similar with PERO_DROME
Px005513.1	3.1816	1.98E-25	1.56E-24	Polypeptide N-acetylgalactosaminyltransferase 1
Px017834.1	3.1217	2.62E-07	9.75E-07	NEDD8 ultimate buster 1; similar with NUB1_HUMAN
Px001526.1	3.1070	4.54E-37	4.47E-36	Lipase 1; similar with LIP1_DROME

Px005368.1	3.0773	1.61E-47	1.85E-46	Malate dehydrogenase 2, mitochondrial
Px009080.1	3.0773	6.20E-26	4.92E-25	Vacuolar ATP synthase subunit S1, putative
Px000371.1	3.0476	0.00E+00	0.00E+00	Lipase 1; similar with LIP1_DROME
Px017452.1	3.0407	2.97E-08	1.18E-07	Putative uncharacterized protein; similar with D6WQR2_TRICA
Px002723.1	2.9848	0.00E+00	0.00E+00	Cytochrome P450 6B7; similar with CP6B7_HELAM
Px004698.1	2.9517	1.27E-16	7.77E-16	NADH dehydrogenase [ubiquinone] iron-sulfur protein 8, mitochondrial
Px012695.1	2.9423	3.93E-150	9.92E-149	Lipase 1; similar with LIP1_DROME
Px003660.1	2.9352	2.46E-11	1.20E-10	NADH dehydrogenase [ubiquinone] iron-sulfur protein 7, mitochondrial
Px011683.2	2.9306	0.00E+00	0.00E+00	Venom carboxylesterase-6; similar with EST6_APIME
Px008089.1	2.8762	0.00E+00	0.00E+00	Pancreatic lipase-related protein 2; similar with LIPR2_CAVPO
Px006190.1	2.8647	0.00E+00	0.00E+00	Lipase member H-A; similar with LIPHA_XENLA
Px007275.2	2.8466	2.07E-26	1.67E-25	Glucosylceramidase; similar with GLCM_PIG
Px011788.2	2.8400	3.24E-222	1.09E-220	Pancreatic lipase-related protein 2; similar with LIPR2_BOVIN
Px012332.1	2.8227	3.03E-20	2.09E-19	ATP synthase subunit beta, mitochondrial
Px016752.1	2.8120	1.98E-36	1.92E-35	Glutamate dehydrogenase 2, mitochondrial
Px015642.1	2.8052	4.74E-10	2.15E-09	Estradiol 17-beta-dehydrogenase 8; similar with DHB8_CANFA
Px001527.1	2.7935	7.50E-09	3.13E-08	Lipase 1; similar with LIP1_DROME
Px002047.1	2.7527	1.74E-261	6.60E-260	Uncharacterized family 31 glucosidase KIAA1161
Px017996.1	2.7512	1.00E-177	2.87E-176	Acetylcholinesterase; similar with ACES_HUMAN
Px016235.1	2.7497	1.52E-09	6.70E-09	3-oxoacyl-[acyl-carrier-protein] reductase FabG
Px002926.1	2.7459	2.44E-55	3.05E-54	Choline dehydrogenase, mitochondrial
Px007430.3	2.7402	9.65E-26	7.64E-25	Tektin-2; similar with TEKT2_BOVIN
Px005043.2	2.7402	3.03E-04	8.22E-04	Probable RNA-directed DNA polymerase from transposon BS
Px002274.1	2.7121	0.00E+00	0.00E+00	Phenoloxidase subunit 2; similar with PRP2_BOMMO
Px000089.1	2.6887	2.89E-87	4.95E-86	Acetylcholinesterase; similar with ACES_FELCA
Px003755.1	2.6780	0.00E+00	0.00E+00	Membrane alanyl aminopeptidase; similar with AMPM_MANSE
Px006228.1	2.6731	3.87E-22	2.80E-21	Beta-1,3-galactosyltransferase 5; similar with B3GT5_MOUSE
Px004610.1	2.6440	7.63E-86	1.29E-84	Sphingosine-1-phosphate lyase; similar with SGPL_DROME
Px006940.2	2.6353	0.00E+00	0.00E+00	Cytosolic beta-glucosidase; similar with GBA3_CAVPO
Px002735.1	2.6010	1.66E-257	6.15E-256	Esterase FE4; similar with ESTF_MYZPE
Px000395.1	2.6001	0.00E+00	0.00E+00	Alpha-amylase 4N; similar with AM4N_DROAN
Px005193.2	2.5958	2.33E-141	5.61E-140	Pancreatic lipase-related protein 2; similar with LIPR2_MYOCO
Px003400.1	2.5938	6.18E-17	3.82E-16	EF-hand calcium-binding domain-containing protein 1
Px009691.1	2.5676	5.36E-19	3.56E-18	Lipase 1; similar with LIP1_DROME
Px005277.3	2.5372	0.00E+00	0.00E+00	Lactase-phlorizin hydrolase; similar with LPH_HUMAN
Px003486.1	2.5359	0.00E+00	0.00E+00	Probable maltase H; similar with MAL2_DROME
Px014883.1	2.5298	7.38E-32	6.64E-31	Ecdysteroid UDP-glucosyltransferase; similar with UDPE_NPVSL
Px014887.1	2.5258	3.34E-136	7.70E-135	Dihydropteridine reductase; similar with DHPR_RAT
Px006345.1	2.5102	0.00E+00	0.00E+00	Pancreatic lipase-related protein 2; similar with LIPR2_MYOCO
Px011049.1	2.5090	4.88E-35	4.62E-34	Acetylcholinesterase; similar with ACES_FELCA
Px004489.1	2.5013	7.47E-48	8.61E-47	Adenosine deaminase CECR1; similar with CECR1_PONAB
Px008866.1	2.4968	7.39E-17	4.55E-16	Chorion peroxidase; similar with PERC_DROME
Px002046.1	2.4907	0.00E+00	0.00E+00	Uncharacterized family 31 glucosidase KIAA1161
Px004750.1	2.4896	0.00E+00	0.00E+00	Pancreatic lipase-related protein 2; similar with LIPR2_RAT
Px002736.1	2.4868	0.00E+00	0.00E+00	Venom carboxylesterase-6; similar with EST6_APIME
Px013884.1	2.4758	5.37E-18	3.43E-17	Probable cytochrome P450 9f2; similar with CP9F2_DROME
Px001913.1	2.4737	1.20E-150	3.03E-149	Gastric triacylglycerol lipase; similar with LIPG_RAT
Px012495.1	2.4696	3.21E-04	8.68E-04	Cytochrome c oxidase subunit 4 isoform 2, mitochondrial
Px007691.1	2.4696	3.21E-04	8.67E-04	Polypeptide N-acetylgalactosaminyltransferase 1
Px016078.1	2.4634	5.95E-157	1.54E-155	ACYPI004563 protein; similar with C4WYA2_ACYPI
Px001785.1	2.4629	2.00E-24	1.53E-23	Gamma-glutamyltranspeptidase 1; similar with GGT1_PIG
Px010022.2	2.4600	2.32E-49	2.71E-48	Cytosolic beta-glucosidase; similar with GBA3_CAVPO
Px008848.1	2.4433	2.05E-168	5.68E-167	Myrosinase 1; similar with MYRO1_BREBR
Px014981.1	2.4259	0.00E+00	0.00E+00	Cytochrome P450 9e2; similar with CP9E2_BLAGE

Px006430.1	2.4038	1.48E-302	6.22E-301	Esterase FE4; similar with ESTF_MYZPE
Px007764.1	2.3822	1.77E-173	4.99E-172	Cytochrome c oxidase subunit 6A1, mitochondrial
Px013390.1	2.3727	1.05E-08	4.33E-08	NADH dehydrogenase [ubiquinone] iron-sulfur protein 3, mitochondrial
Px001708.1	2.3708	0.00E+00	0.00E+00	Aminopeptidase N; similar with AMPN_PLUXY
Px011475.1	2.3683	0.00E+00	0.00E+00	Lipase 3; similar with LIP3_DROME
Px004327.1	2.3658	1.41E-24	1.08E-23	Glyceraldehyde-3-phosphate dehydrogenase 2
Px004752.1	2.3527	0.00E+00	0.00E+00	Pancreatic lipase-related protein 2; similar with LIPR2_BOVIN
Px017856.1	2.3523	6.28E-34	5.85E-33	Succinate dehydrogenase cytochrome b560 subunit, mitochondrial
Px004753.1	2.3410	9.82E-208	3.12E-206	Pancreatic lipase-related protein 2; similar with LIPR2_MYOCO
Px001705.1	2.3213	0.00E+00	0.00E+00	Membrane alanyl aminopeptidase; similar with AMPM_MANSE
Px006441.1	2.3113	0.00E+00	0.00E+00	Lipase member H; similar with LIPH_RAT
Px005876.1	2.3107	8.18E-70	1.20E-68	Patatin-like phospholipase domain-containing protein 2
Px004604.5	2.2867	3.97E-06	1.34E-05	Envelope fusion protein; similar with ENV_NPVLD
Px005528.1	2.2789	3.18E-04	8.62E-04	Cytochrome c oxidase copper chaperone
Px012592.1	2.2775	0.00E+00	0.00E+00	Juvenile hormone esterase; similar with ESTJ_HELVI
Px011160.1	2.2730	0.00E+00	0.00E+00	Lactase-phlorizin hydrolase; similar with LPH_HUMAN
Px008278.1	2.2655	4.42E-37	4.36E-36	Membrane alanyl aminopeptidase; similar with AMPM_MANSE
Px008810.1	2.2431	1.22E-23	9.18E-23	Probable tubulin polyglutamylase TTLL1
Px005687.1	2.2408	8.49E-30	7.38E-29	Venom carboxylesterase-6; similar with EST6_APIME
Px001456.1	2.2249	2.79E-169	7.74E-168	Gastric triacylglycerol lipase; similar with LIPG_RAT
Px007927.1	2.2154	4.79E-60	6.34E-59	V-type proton ATPase subunit G; similar with VATG_MANSE
Px005369.1	2.1951	1.27E-26	1.03E-25	Malate dehydrogenase; similar with MDH_EDWI9
Px004946.1	2.1753	3.57E-18	2.30E-17	ATP synthase subunit gamma, mitochondrial
Px006319.1	2.1734	2.37E-82	3.89E-81	Retinol dehydrogenase 11; similar with RDH11_HUMAN
Px018023.1	2.1708	1.08E-51	1.30E-50	Adenylate kinase isoenzyme 1; similar with KAD1_CHICK
Px016488.1	2.1647	1.18E-09	5.22E-09	Glucose dehydrogenase [acceptor]; similar with DHGL_DROME
Px008152.2	2.1559	5.44E-31	4.83E-30	Alpha-1,6-mannosyl-glycoprotein 2-beta-N-acetylglucosaminyltransferase
Px004209.1	2.1558	1.50E-35	1.43E-34	Putative uncharacterized protein; similar with D6WSS5_TRICA
Px002296.1	2.1474	1.41E-169	3.92E-168	Pancreatic triacylglycerol lipase; similar with LIPP_HUMAN
Px010079.1	2.1426	1.30E-95	2.36E-94	Glutamine synthetase 1, mitochondrial
Px003205.1	2.1414	1.92E-12	9.88E-12	Envelope fusion protein; similar with ENV_NPVLD
Px003742.1	2.1377	3.18E-39	3.23E-38	Sphingomyelin phosphodiesterase; similar with ASM_HUMAN
Px003649.1	2.1363	0.00E+00	0.00E+00	Esterase FE4; similar with ESTF_MYZPE
Px006683.1	2.1362	2.49E-07	9.29E-07	Envelope fusion protein; similar with ENV_NPVLD
Px002964.1	2.1362	2.49E-07	9.29E-07	Putative uncharacterized protein; similar with E9H4D7_DAPPU
Px005679.1	2.1335	9.60E-184	2.83E-182	Uncharacterized phosphotransferase yvkC
Px005902.4	2.1318	2.80E-119	5.89E-118	Cytochrome P450 6B6; similar with CP6B6_HELAM
Px003273.1	2.1129	6.40E-16	3.82E-15	Pancreatic lipase-related protein 2; similar with LIPR2_CAVPO
Px008811.1	2.1078	3.17E-18	2.04E-17	Probable tubulin polyglutamylase TTLL1
Px004854.1	2.1011	1.06E-15	6.27E-15	Ecdysteroid UDP-glucosyltransferase; similar with UDPE_NPVSL
Px014748.1	2.0912	9.16E-74	1.40E-72	Unknown functio
Px011501.1	2.0881	7.26E-17	4.47E-16	ATP synthase subunit beta, mitochondrial; similar with ATPB_RAT
Px000744.1	2.0773	1.67E-04	4.72E-04	Lipase member H-A; similar with LIPHA_XENLA
Px011796.5	2.0740	1.05E-51	1.26E-50	Putative aminopeptidase W07G4.4; similar with YH24_CAEEL
Px006440.1	2.0668	1.56E-48	1.81E-47	Phospholipase A1 member A; similar with PLA1A_HUMAN
Px004200.1	2.0610	1.11E-81	1.81E-80	Putative phosphatidate phosphatase; similar with WUN_DROME
Px011066.2	2.0600	8.86E-30	7.71E-29	EF-hand domain-containing family member B
Px006500.1	2.0492	9.92E-42	1.05E-40	Peroxisomal acyl-coenzyme A oxidase 3
Px007402.1	2.0481	1.63E-12	8.38E-12	Dephospho-CoA kinase domain-containing protein
Px006763.1	2.0407	1.02E-05	3.30E-05	Peroxisomal acyl-coenzyme A oxidase 3
Px016492.1	2.0372	2.78E-33	2.57E-32	Luciferin 4-monooxygenase; similar with LUCI_LUCCR
Px011034.1	2.0305	3.34E-62	4.54E-61	Lysosomal alpha-glucosidase; similar with LYAG_PONAB
Px011280.1	2.0291	2.23E-60	2.97E-59	4-coumarate--CoA ligase 1; similar with 4CL1_TOBAC
Px010481.1	2.0283	7.38E-08	2.86E-07	Tctex1 domain-containing protein 3; similar with TC1D3_MOUSE

Px007291.1	2.0198	4.19E-15	2.41E-14	Cytochrome c oxidase subunit 5B, mitochondrial
Px012696.1	2.0159	2.21E-135	5.07E-134	Lipase 3; similar with LIP3_DROME
Px005079.1	1.9962	0.00E+00	0.00E+00	Lactase-phlorizin hydrolase; similar with LPH_HUMAN
Px013789.1	1.9877	3.65E-89	6.34E-88	L-threonine 3-dehydrogenase; similar with TDH_LEGPL
Px000515.1	1.9774	1.03E-98	1.91E-97	Esterase FE4; similar with ESTF_MYZPE
Px012400.1	1.9673	6.21E-38	6.19E-37	Argininosuccinate lyase; similar with ARLY2_ANAPL
Px006941.1	1.9351	4.59E-121	9.83E-120	Myrosinase 1; similar with MYRO1_BREBR
Px008153.1	1.9344	1.29E-24	9.93E-24	Alpha-1,6-mannosyl-glycoprotein 2-beta-N-acetylglucosaminyltransferase
Px004751.1	1.9293	0.00E+00	0.00E+00	Pancreatic triacylglycerol lipase; similar with LIPP_HUMAN
Px003754.1	1.9228	1.67E-147	4.13E-146	Membrane alanyl aminopeptidase; similar with AMPM_MANSE
Px017171.1	1.9108	8.59E-16	5.10E-15	Putative inositol monophosphatase 3; similar with IMPA3_DROME
Px014216.1	1.9094	1.32E-40	1.38E-39	Cytochrome P450 6B6; similar with CP6B6_HELAM
Px016338.1	1.9089	9.52E-63	1.31E-61	Lipase 1; similar with LIP1_DROME
Px016609.1	1.8983	8.66E-07	3.09E-06	Enkurin; similar with ENKUR_HUMAN
Px013937.1	1.8935	1.13E-09	4.99E-09	3-ketoacyl-CoA thiolase, mitochondrial; similar with THIM_HUMAN
Px012937.2	1.8846	1.37E-05	4.37E-05	Phospholipase D1; similar with PLD1_HUMAN
Px000872.2	1.8756	1.82E-09	7.93E-09	Ecdysteroid UDP-glucosyltransferase; similar with UDPE_NPVSL
Px000394.1	1.8666	1.72E-210	5.53E-209	Alpha-amylase 4N; similar with AM4N_DROAN
Px010853.1	1.8586	7.49E-18	4.76E-17	Retinol dehydrogenase 11; similar with RDH11_MOUSE
Px004843.1	1.8517	8.05E-08	3.11E-07	ORF; similar with Q8I7Q1_DROME
Px009920.1	1.8428	8.21E-15	4.67E-14	Branched-chain-amino-acid aminotransferase, mitochondrial
Px000647.3	1.8232	6.21E-280	2.49E-278	UDP-glucuronosyltransferase 2B7; similar with UD2B7_HUMAN
Px010831.1	1.8205	7.51E-09	3.13E-08	Luciferin 4-monooxygenase; similar with LUCI_PHOPE
Px017172.1	1.8142	5.10E-12	2.56E-11	Serine palmitoyltransferase 1; similar with SPTC1_MOUSE
Px002968.1	1.8131	5.34E-41	5.58E-40	Adenylate kinase isoenzyme 1; similar with KAD1_CHICK
Px007409.1	1.8110	8.48E-30	7.38E-29	Succinate dehydrogenase [ubiquinone] iron-sulfur subunit, mitochondrial
Px018019.1	1.8031	1.95E-05	6.12E-05	Putative uncharacterized protein; similar with E2ARC4_9HYME
Px008606.3	1.8031	1.95E-05	6.12E-05	Gamma-interferon-inducible lysosomal thiol reductase
Px009194.1	1.8005	8.55E-179	2.46E-177	Dihydropteridine reductase; similar with DHPR_RAT
Px003540.1	1.7898	3.78E-38	3.78E-37	Hexokinase type 2; similar with HXK2_DROME
Px004684.1	1.7858	0.00E+00	0.00E+00	Tyrosine 3-monooxygenase; similar with TY3H_DROME
Px011697.1	1.7841	4.24E-28	3.56E-27	Bifunctional methylenetetrahydrofolate dehydrogenase/cyclohydrolase, mitochondrial
Px011727.1	1.7734	6.73E-64	9.35E-63	Protein GCY; similar with GCY_YEAST
Px002295.1	1.7684	0.00E+00	0.00E+00	Pancreatic lipase-related protein 2; similar with LIPR2_BOVIN
Px015449.1	1.7626	1.93E-12	9.88E-12	Acetyl-CoA carboxylase 1; similar with ACACA_SHEEP
Px000123.1	1.7598	0.00E+00	0.00E+00	Putative aldehyde dehydrogenase family 7 member A1 homolog
Px003753.1	1.7587	0.00E+00	0.00E+00	Membrane alanyl aminopeptidase; similar with AMPM_HELVI
Px017680.3	1.7524	1.24E-28	1.06E-27	CaiB/baiF CoA-transferase family protein C7orf10 homolog
Px011641.3	1.7499	0.00E+00	0.00E+00	Vanin-like protein 2; similar with VNNL2_DROME
Px014078.1	1.7425	5.92E-30	5.19E-29	Fructose-bisphosphate aldolase; similar with ALF_DROME
Px014054.1	1.7402	9.14E-05	2.66E-04	Retrovirus-related Pol polyprotein from transposon 297
Px002071.1	1.7379	9.28E-30	8.06E-29	Beta-1,3-galactosyltransferase 5; similar with B3GT5_HUMAN
Px010260.3	1.7301	6.29E-25	4.87E-24	Alcohol dehydrogenase [NADP+] A; similar with A1A1A_DANRE
Px011755.1	1.7284	1.92E-39	1.96E-38	Antennal esterase CXE4; similar with D3GDL5_SPOLI
Px000957.1	1.7245	2.09E-58	2.70E-57	Putative lipoyltransferase 2, mitochondrial
Px011476.1	1.7230	8.10E-19	5.34E-18	Lipase 3; similar with LIP3_DROME
Px009343.1	1.7147	2.39E-06	8.20E-06	NADH-ubiquinone oxidoreductase 75 kDa subunit, mitochondrial
Px005070.1	1.7143	1.14E-73	1.74E-72	Glucose 1-dehydrogenase 2; similar with DHG2_BACME
Px011320.1	1.7053	2.69E-04	7.34E-04	Cytochrome b-c1 complex subunit Rieske, mitochondrial
Px009518.1	1.6962	4.26E-05	1.29E-04	Allantoicase; similar with ALLC_DANRE
Px008134.5	1.6910	0.00E+00	0.00E+00	Glucose dehydrogenase [acceptor]; similar with DHGL_DROME
Px001113.1	1.6873	1.59E-11	7.83E-11	Ecdysteroid UDP-glucosyltransferase; similar with UDPE_NPVSL
Px008154.1	1.6773	3.06E-26	2.46E-25	Alpha-1,6-mannosyl-glycoprotein 2-beta-N-acetylglucosaminyltransferase
Px007305.1	1.6562	9.37E-42	9.88E-41	C-1-tetrahydrofolate synthase, cytoplasmic

Px004798.2	1.6515	1.57E-68	2.27E-67	Malate dehydrogenase, mitochondrial; similar with MDHM_PONAB
Px003712.1	1.6490	1.05E-19	7.17E-19	Acetylcholinesterase; similar with ACES_CULPI
Px001047.1	1.6266	1.35E-135	3.11E-134	Heat shock protein 67B2; similar with HS6B_DROME
Px003251.1	1.6237	1.02E-19	6.98E-19	Putative cuticle protein; similar with C0H6E8_BOMMO
Px017997.1	1.6216	3.59E-04	9.62E-04	Esterase FE4; similar with ESTF_MYZPE
Px004065.1	1.6192	1.53E-09	6.71E-09	Mannosyl-oligosaccharide alpha-1,2-mannosidase isoform A
Px016278.1	1.6052	1.76E-11	8.64E-11	Cytoplasmic aconitate hydratase; similar with ACOC_MOUSE
Px004310.2	1.5938	1.11E-09	4.89E-09	Probable trans-2-enoyl-CoA reductase, mitochondrial
Px004643.1	1.5882	6.58E-06	2.17E-05	Cytochrome P450 6B6; similar with CP6B6_HELAM
Px000132.1	1.5866	3.04E-79	4.87E-78	FGGY carbohydrate kinase domain-containing protein
Px011340.1	1.5860	0.00E+00	0.00E+00	Non-specific lipid-transfer protein; similar with NLTP_CHICK
Px016731.1	1.5768	2.81E-27	2.33E-26	Acyl-CoA synthetase family member 3, mitochondrial
Px015306.1	1.5761	1.08E-11	5.34E-11	1,2-dihydroxy-3-keto-5-methylthiopentene dioxygenase
Px017275.1	1.5732	4.40E-10	1.99E-09	Inositol monophosphatase 3; similar with IMPA3_DANRE
Px001531.1	1.5708	7.84E-60	1.04E-58	Probable peroxisomal acyl-coenzyme A oxidase 1
Px016564.1	1.5653	3.09E-27	2.55E-26	Hydroxyacyl-coenzyme A dehydrogenase, mitochondrial
Px009165.1	1.5540	5.35E-37	5.25E-36	Retinol dehydrogenase 14; similar with RDH14_MOUSE
Px008706.2	1.5512	3.40E-05	1.04E-04	WW domain-containing oxidoreductase
Px012743.1	1.5433	6.47E-14	3.55E-13	Probable dolichol-phosphate mannosyltransferase
Px014172.1	1.5367	3.01E-07	1.11E-06	Heparanase; similar with HPSE_BOVIN
Px010498.1	1.5315	2.77E-59	3.62E-58	Fumarylacetoacetate hydrolase domain-containing protein 2
Px002990.1	1.5298	2.84E-05	8.78E-05	LINE-1 reverse transcriptase homolog; similar with LIN1_NYCCO
Px001106.1	1.5290	1.97E-08	7.95E-08	Protein retinal degeneration B; similar with RDGB_DROME
Px013958.1	1.5211	6.83E-145	1.68E-143	Cytochrome P450 6k1; similar with CP6K1_BLAGE
Px007047.2	1.5134	9.55E-05	2.77E-04	Probable peroxisomal acyl-coenzyme A oxidase 1
Px016262.1	1.5084	3.45E-19	2.30E-18	Cysteine sulfinic acid decarboxylase; similar with CSAD_HUMAN
Px007772.1	1.5076	2.80E-66	3.96E-65	Esterase B1; similar with EST1_CULPI
Px014702.1	1.5035	1.75E-04	4.92E-04	Lysine-specific demethylase 8; similar with KDM8_RAT
Px008849.1	1.5018	7.18E-50	8.45E-49	Myrosinase 1; similar with MYRO1_BREBR
Px016066.1	1.4923	1.12E-13	6.06E-13	Lipase member M; similar with LIPM_MOUSE
Px006995.1	1.4923	7.94E-05	2.33E-04	Polypeptide N-acetylgalactosaminyltransferase 1
Px004945.1	1.4848	1.39E-157	3.62E-156	Protein henna; similar with PH4H_DROME
Px011686.1	1.4813	2.69E-20	1.86E-19	5-formyltetrahydrofolate cyclo-ligase; similar with MTHFS_HUMAN
Px016493.1	1.4751	2.83E-29	2.43E-28	Luciferin 4-monooxygenase; similar with LUCI_LUCMI
Px011550.1	1.4749	3.05E-11	1.48E-10	Glycerate kinase; similar with GLCTK_RAT
Px011338.1	1.4635	5.55E-12	2.79E-11	GTP cyclohydrolase 1; similar with GCH1_DROME
Px014982.1	1.4635	0.00E+00	0.00E+00	Probable cytochrome P450 9f2; similar with CP9F2_DROME
Px000965.1	1.4634	3.13E-36	3.03E-35	D-amino-acid oxidase 2; similar with OXDA2_CAEEL
Px009184.1	1.4536	6.85E-11	3.26E-10	Adenosine deaminase-like protein; similar with ADAL_BOVIN
Px013203.1	1.4517	2.86E-06	9.77E-06	UDP-glucuronosyltransferase 2B2; similar with UD2B2_RAT
Px008269.1	1.4462	9.77E-33	8.96E-32	Chondroitin sulfate synthase 1; similar with CHSS1_MOUSE
Px016080.1	1.4117	3.08E-05	9.48E-05	Glucosamine-6-phosphate isomerase; similar with GNPI_AEDAE
Px011844.1	1.4117	3.08E-05	9.48E-05	Adenylate kinase 7; similar with KAD7_MOUSE
Px011611.1	1.4113	0.00E+00	0.00E+00	Probable enoyl-CoA hydratase, mitochondrial
Px006720.1	1.4070	1.32E-07	5.02E-07	Glycerol-3-phosphate acyltransferase 4
Px009407.1	1.4048	3.00E-27	2.47E-26	Glycoprotein-N-acetylgalactosamine 3-beta-galactosyltransferase 1
Px013538.1	1.4047	8.56E-19	5.64E-18	2-oxoglutarate dehydrogenase, mitochondrial
Px017196.1	1.3834	3.80E-22	2.75E-21	Choline/ethanolamine kinase; similar with CHKB_HUMAN
Px001847.1	1.3803	1.09E-180	3.16E-179	Aminoacylase-1; similar with ACY1_MOUSE
Px001370.1	1.3780	6.09E-19	4.04E-18	Aminoacylase-1; similar with ACY1_MOUSE
Px015891.1	1.3621	4.81E-87	8.23E-86	Peroxidase; similar with PERO_DROME
Px015301.3	1.3558	5.98E-18	3.81E-17	V-type proton ATPase subunit S1; similar with VAS1_MOUSE
Px009798.1	1.3537	1.57E-40	1.63E-39	Aminoacylase-1A; similar with ACY1A_RAT
Px010161.1	1.3476	3.12E-26	2.50E-25	GTP cyclohydrolase 1; similar with GCH1_DROME

Px000763.1	1.3380	2.84E-09	1.22E-08	Acetyl-CoA carboxylase; similar with ACAC_CHICK
Px003338.1	1.3380	2.84E-09	1.22E-08	ATP synthase subunit d, mitochondrial
Px012027.3	1.3347	9.75E-93	1.73E-91	Protein 5NUC; similar with 5NTD_LUTLO
Px009776.1	1.3252	2.34E-08	9.36E-08	Mevalonate kinase; similar with KIME_DICDI
Px004571.1	1.3081	2.26E-185	6.68E-184	Aldose reductase; similar with ALDR_BOVIN
Px016456.1	1.3048	0.00E+00	0.00E+00	Lambda-crystallin homolog; similar with CRYL1_HUMAN
Px008217.3	1.3035	8.78E-09	3.64E-08	Phosphatidylinositol 4,5-bisphosphate 5-phosphatase A
Px015448.1	1.2854	3.01E-05	9.28E-05	Acetyl-CoA carboxylase; similar with ACAC_CHICK
Px016834.1	1.2833	2.58E-98	4.76E-97	Probable 3-hydroxyacyl-CoA dehydrogenase B0272.3
Px016733.1	1.2758	1.82E-09	7.92E-09	Acyl-CoA synthetase family member 3, mitochondrial
Px006848.1	1.2745	1.88E-14	1.05E-13	UDP-glucose 6-dehydrogenase; similar with UGDH_DROME
Px006311.1	1.2734	4.37E-18	2.80E-17	Chorion peroxidase; similar with PERC_ANOGA
Px002115.1	1.2726	1.76E-36	1.71E-35	5-formyltetrahydrofolate cyclo-ligase
Px006292.1	1.2679	2.20E-13	1.18E-12	Ecdysteroid UDP-glucosyltransferase; similar with UDPE_NPVSL
Px001937.2	1.2673	3.55E-115	7.31E-114	Phosphatidate phosphatase LPIN2; similar with LPIN2_MOUSE
Px003365.1	1.2639	2.20E-57	2.82E-56	Luciferin 4-monooxygenase; similar with LUCI_PHOPE
Px000372.1	1.2617	2.59E-11	1.26E-10	Putative gamma-glutamyltransferase ywrD
Px013936.1	1.2531	1.39E-28	1.18E-27	3-ketoacyl-CoA thiolase, mitochondrial
Px012993.1	1.2368	4.80E-99	8.92E-98	Multifunctional protein ADE2; similar with PUR6_DROME
Px006054.1	1.2358	6.60E-273	2.59E-271	Myrosinase 1; similar with MYRO1_BREBR
Px014795.1	1.2270	4.25E-09	1.81E-08	5'-nucleotidase; similar with 5NTD_DISOM
Px017253.1	1.2169	4.53E-37	4.47E-36	Cytochrome P450 6B2; similar with CP6B2_HELAM
Px011707.1	1.2143	2.86E-35	2.71E-34	Amidophosphoribosyltransferase; similar with PUR1_DROME
Px007055.2	1.2138	3.00E-67	4.29E-66	Branched-chain-amino-acid aminotransferase, cytosolic
Px001867.3	1.2132	1.80E-11	8.86E-11	AMP deaminase 2; similar with AMPD2_HUMAN
Px012198.1	1.2125	2.46E-68	3.54E-67	Fatty acid synthase; similar with FAS_RAT
Px017168.1	1.2122	2.42E-07	9.06E-07	Iron/zinc purple acid phosphatase-like protein
Px011286.1	1.2119	8.71E-219	2.89E-217	Glutaryl-CoA dehydrogenase, mitochondrial
Px002733.2	1.2093	0.00E+00	0.00E+00	Aldehyde dehydrogenase, dimeric NADP-preferring
Px002532.1	1.2056	1.34E-124	2.93E-123	Peroxidasin; similar with PXDN_DROME
Px002690.2	1.1998	1.95E-17	1.22E-16	Dolichol kinase; similar with DOLK_MOUSE
Px010938.1	1.1997	2.45E-12	1.25E-11	2-oxo-4-hydroxy-4-carboxy-5-ureidoimidazoline decarboxylase
Px000039.1	1.1965	1.40E-231	4.93E-230	UDP-glucuronosyltransferase 1-8; similar with UD18_RAT
Px003013.3	1.1926	3.26E-165	8.82E-164	Lipoyltransferase 1, mitochondrial; similar with LIPT_MOUSE
Px001933.1	1.1912	1.41E-09	6.20E-09	Myrosinase 1; similar with MYRO1_BREBR
Px005839.1	1.1906	1.80E-18	1.17E-17	Mannosyl-oligosaccharide alpha-1,2-mannosidase isoform A
Px010887.1	1.1902	3.16E-21	2.24E-20	Phosphoenolpyruvate carboxykinase [GTP]
Px001894.8	1.1880	5.51E-25	4.27E-24	Ethanolamine-phosphate cytidylyltransferase
Px009877.1	1.1833	6.80E-08	2.64E-07	Ferrochelatase, mitochondrial; similar with HEMH_DROME
Px000954.1	1.1823	1.17E-74	1.80E-73	Succinyl-CoA ligase [GDP-forming] subunit beta, mitochondrial
Px012054.1	1.1758	3.79E-12	1.91E-11	Unknown functio
Px001224.1	1.1741	3.07E-05	9.43E-05	Maleylacetoacetate isomerase; similar with MAAI_MOUSE
Px006815.1	1.1714	0.00E+00	0.00E+00	Regucalcin; similar with RGN_DANRE
Px009624.5	1.1652	5.53E-155	1.42E-153	Peroxisomal multifunctional enzyme type 2
Px003966.1	1.1609	1.72E-15	1.01E-14	Tubulin polyglutamylase ttll6; similar with TTLL6_DANRE
Px006643.3	1.1605	8.63E-35	8.15E-34	Luciferin 4-monooxygenase; similar with LUCI_LUCMI
Px012467.1	1.1592	1.77E-09	7.71E-09	GTP:AMP phosphotransferase, mitochondrial
Px013246.1	1.1587	3.16E-98	5.83E-97	Dehydrogenase/reductase SDR family member 4
Px000720.1	1.1574	2.24E-12	1.15E-11	Galactokinase; similar with GALK1_HUMAN
Px004131.5	1.1474	6.20E-26	4.92E-25	Calcium-independent phospholipase A2-gamma
Px010099.1	1.1453	2.76E-19	1.85E-18	Phospholipase D1; similar with PLD1_RAT
Px011987.1	1.1403	4.97E-07	1.81E-06	Amine oxidase [flavin-containing] B; similar with AOFB_MOUSE
Px001164.1	1.1390	6.83E-117	1.42E-115	UDP-glucuronosyltransferase; similar with UGT3_PLEPL
Px009675.1	1.1334	1.86E-96	3.40E-95	UDP-glucuronosyltransferase 1-2; similar with UD12_MOUSE

Px011027.1	1.1279	4.95E-11	2.37E-10	ORF; similar with Q8I7Q1_DROME
Px015376.1	1.1200	1.05E-146	2.61E-145	Phosphoenolpyruvate carboxykinase [GTP]; similar with PCKG_DROME
Px015080.1	1.1199	3.36E-06	1.14E-05	Ecdysteroid UDP-glucosyltransferase; similar with UDPE_NPVMB
Px002044.1	1.1191	1.32E-04	3.76E-04	Phosphatidylinositol 3-kinase catalytic subunit type 3
Px007883.1	1.1153	4.95E-21	3.49E-20	Probable medium-chain specific acyl-CoA dehydrogenase, mitochondrial
Px005106.1	1.1107	1.62E-25	1.28E-24	Aminopeptidase N; similar with AMPN_CHICK
Px000042.3	1.1085	4.45E-27	3.66E-26	Alcohol dehydrogenase [NADP+]; similar with AK1A1_BOVIN
Px003316.1	1.0981	0.00E+00	0.00E+00	C-1-tetrahydrofolate synthase, cytoplasmic; similar with C1TC_SPOFR
Px005939.1	1.0952	5.25E-05	1.58E-04	ORF; similar with Q8I7Q1_DROME
Px004129.1	1.0899	9.10E-17	5.57E-16	Putative ferric-chelate reductase 1 homolog; similar with FRRS1_DROME
Px008244.2	1.0882	5.98E-89	1.04E-87	Sialic acid synthase; similar with SIAS_HUMAN
Px009265.1	1.0812	1.04E-17	6.55E-17	Sepiapterin reductase; similar with SPRE_XENTR
Px016558.1	1.0773	3.20E-04	8.66E-04	Putative glycerol kinase 5; similar with GLPK5_DANRE
Px005900.1	1.0773	1.30E-16	7.94E-16	Cytochrome P450 6B2; similar with CP6B2_HELAM
Px002103.1	1.0773	2.09E-05	6.54E-05	Dolichol-phosphate mannosyltransferase subunit 3
Px000764.1	1.0743	4.55E-43	4.88E-42	Acetyl-CoA carboxylase; similar with ACAC_CHICK
Px007394.2	1.0691	1.25E-39	1.29E-38	Peroxidasin homolog; similar with PXDN_MOUSE
Px006816.1	1.0638	0.00E+00	0.00E+00	2-oxoisovalerate dehydrogenase subunit alpha, mitochondrial
Px011285.1	1.0624	2.26E-250	8.22E-249	UDP-glucuronosyltransferase 2B4; similar with UD2B4_HUMAN
Px000825.3	1.0612	4.33E-28	3.63E-27	Ferrochelatase, mitochondrial; similar with HEMH_DROME
Px004924.1	1.0611	3.58E-05	1.09E-04	Polypeptide N-acetylgalactosaminyltransferase 3
Px013239.1	1.0562	3.46E-07	1.28E-06	Putative glycerol kinase 3; similar with GLPK3_HUMAN
Px008638.1	1.0552	4.56E-37	4.49E-36	Thioredoxin domain-containing protein 17; similar with TXD17_PONAB
Px016756.1	1.0544	4.92E-12	2.48E-11	Cytoplasmic aconitate hydratase; similar with ACOC_HUMAN
Px015569.1	1.0487	2.19E-22	1.60E-21	3-oxoacyl-[acyl-carrier-protein] reductase FabG
Px012098.2	1.0475	2.15E-05	6.71E-05	Lipase member I; similar with LIPI_HUMAN
Px012834.1	1.0441	9.14E-32	8.21E-31	Phosphopantothenate--cysteine ligase; similar with PPCS_HUMAN
Px011757.1	1.0401	5.69E-11	2.72E-10	Esterase FE4; similar with ESTF_MYZPE
Px011708.1	1.0393	3.52E-26	2.82E-25	Amidophosphoribosyltransferase; similar with PUR1_DROME
Px016062.1	1.0391	1.04E-10	4.90E-10	Prostamide/prostaglandin F synthase; similar with PGFS_RAT
Px007493.1	1.0389	5.06E-106	9.92E-105	Delta-1-pyrroline-5-carboxylate synthase; similar with P5CS_PONAB
Px016651.3	1.0381	6.93E-06	2.28E-05	Polypeptide N-acetylgalactosaminyltransferase 5
Px013730.1	1.0378	8.17E-18	5.18E-17	Myrosinase 1; similar with MYRO1_BREBR
Px004354.1	1.0356	2.73E-18	1.76E-17	UDP-N-acetylhexosamine pyrophosphorylase-like protein
Px003748.1	1.0252	1.80E-24	1.37E-23	Sphingosine-1-phosphate lyase; similar with SGPL_DROME
Px010631.1	1.0225	2.51E-26	2.02E-25	Sphingomyelin phosphodiesterase; similar with ASM_HUMAN
Px005056.1	1.0223	1.78E-132	4.01E-131	V-type proton ATPase subunit e 2; similar with VA0E2_HUMAN
Px008837.1	1.0217	1.55E-94	2.77E-93	Long-chain-fatty-acid--CoA ligase ACSBG2; similar with ACBG2_XENLA
Px010263.1	1.0192	2.96E-04	8.05E-04	Probable methylmalonate-semialdehyde dehydrogenase [acylating], mitochondrial
Px001460.1	1.0189	4.64E-14	2.57E-13	Alpha-N-acetylglucosaminidase; similar with ANAG_HUMAN
Px015217.1	1.0179	5.90E-29	5.04E-28	4-aminobutyrate aminotransferase, mitochondrial; similar with GABT_PIG
Px012551.1	1.0171	6.11E-09	2.57E-08	Uncharacterized protein R102.4; similar with YF64_CAEEL
Px014822.1	1.0107	6.18E-81	1.00E-79	3-ketoacyl-CoA thiolase, mitochondrial; similar with THIM_HUMAN
Px013693.1	1.0094	5.72E-08	2.23E-07	Aldehyde oxidase; similar with ADO_HUMAN
Px003215.1	1.0078	3.27E-10	1.49E-09	N-acetyllactosaminide beta-1,3-N-acetylglucosaminyltransferase
Px015882.1	1.0071	2.32E-110	4.67E-109	Alcohol dehydrogenase [NADP+] A; similar with A1A1A_DANRE
Px010660.1	1.0065	4.71E-17	2.92E-16	Probable general secretion pathway protein D; similar with GSPD_ECOLI
Px009169.1	1.0057	5.15E-06	1.71E-05	Myrosinase 1; similar with MYRO1_BREBR
Px004525.4	1.0036	2.33E-16	1.41E-15	Glyoxylate reductase/hydroxypyruvate reductase
Px013169.1	1.0023	2.92E-07	1.09E-06	Lactase-phlorizin hydrolase; similar with LPH_HUMAN
Px002054.1	-9.5448	1.78E-04	4.98E-04	Uncharacterized oxidoreductase MexAM1_META1p0182
Px017388.1	-8.2367	1.78E-04	4.99E-04	Neither inactivation nor afterpotential protein G
Px000980.1	-3.3822	3.42E-05	1.05E-04	Catalase; similar with CATA_PONAB
Px005914.1	-2.5077	1.83E-04	5.12E-04	Putative uncharacterized protein; similar with D6WG00_TRICA

Px016811.1	-2.2109	3.61E-99	6.74E-98	Retrovirus-related Pol polyprotein LINE-1; similar with POL2_MOUSE
Px006540.1	-2.1451	2.18E-04	6.03E-04	ACYPI009600 protein; similar with C4WT25_ACYPI
Px009473.1	-2.1154	2.05E-08	8.25E-08	DNA-directed RNA polymerases I, II, and III subunit RPABC3
Px002199.1	-2.0641	1.94E-17	1.22E-16	4-coumarate--CoA ligase-like 5; similar with 4CLL5_ARATH
Px007377.1	-1.9772	8.53E-07	3.05E-06	Glucose 1-dehydrogenase 1; similar with DHG1_BACME
Px006557.1	-1.7301	1.15E-06	4.05E-06	Ribonucleoside-diphosphate reductase small chain
Px009302.2	-1.5524	1.01E-125	2.21E-124	Putative uncharacterized protein; similar with D7GYL0_TRICA
Px015198.2	-1.5238	2.14E-20	1.48E-19	NADH dehydrogenase [ubiquinone] 1 alpha subcomplex subunit 8
Px017708.4	-1.4493	1.01E-40	1.05E-39	Long-chain-fatty-acid--CoA ligase 1; similar with ACSL1_RAT
Px010117.1	-1.3859	3.21E-207	1.02E-205	Asparagine synthetase [glutamine-hydrolyzing]; similar with ASNS_RAT
Px007720.1	-1.3396	2.40E-31	2.14E-30	Catalase; similar with CATA_GLARU
Px004229.1	-1.2823	8.45E-31	7.47E-30	Retrovirus-related Pol polyprotein LINE-1; similar with POL2_MOUSE
Px005654.1	-1.2576	5.56E-25	4.31E-24	DNA-directed RNA polymerase III subunit RPC4
Px013191.1	-1.2294	5.69E-11	2.72E-10	Aromatic-L-amino-acid decarboxylase; similar with DDC_RAT
Px007031.1	-1.2120	0.00E+00	0.00E+00	Basic juvenile hormone-suppressible protein 2; similar with BJSB2_TRINI
Px017624.1	-1.2048	5.92E-12	2.97E-11	DNA-directed RNA polymerase I subunit RPA2
Px012350.1	-1.2045	5.28E-49	6.14E-48	Ribonucleoside-diphosphate reductase large subunit
Px002394.3	-1.1661	5.87E-70	8.60E-69	D-beta-hydroxybutyrate dehydrogenase, mitochondrial
Px010954.1	-1.1513	1.34E-08	5.50E-08	Probable DNA-directed RNA polymerases I and III subunit RPAC2
Px012461.1	-1.1191	1.86E-13	9.95E-13	Lethal(2)neighbour of tid protein; similar with NT56_DROME
Px009682.1	-1.0521	0.00E+00	0.00E+00	Cytochrome c oxidase subunit 6A1, mitochondrial
Px001485.1	-1.0439	1.14E-09	5.04E-09	Putative 115 kDa protein in type-1 retrotransposable element R1DM
Px012289.1	-1.0288	3.16E-06	1.07E-05	Liver carboxylesterase; similar with EST1_MESAU
Px004110.1	-1.0281	8.36E-07	2.99E-06	Aldose reductase; similar with ALDR_RABIT
Px012716.1	-1.0128	4.03E-70	5.92E-69	Proline dehydrogenase 1, mitochondrial; similar with PROD_DROME
Px013099.1	-1.0105	3.25E-117	6.80E-116	Unknown functio
Px006040.1	-1.0062	1.31E-04	3.75E-04	RNA-directed DNA polymerase from mobile element jockey

Involved in metabolism of xenobiotics by cytochrome P450

Px009293.1	11.1045	9.55E-06	3.10E-05	Probable cytochrome P450 304a1; similar with CP304_DROME
Px013527.1	10.4804	7.08E-09	2.96E-08	Carbonyl reductase [NADPH] 1; similar with CBR1_RABIT
Px009572.1	9.2901	5.34E-07	1.94E-06	Cytochrome P450 6B1; similar with CP6B1_PAPPO
Px007386.1	4.0773	1.56E-08	6.32E-08	Carbonyl reductase [NADPH] 1; similar with CBR1_PIG
Px002723.1	2.9848	0.00E+00	0.00E+00	Cytochrome P450 6B7; similar with CP6B7_HELAM
Px014883.1	2.5298	7.38E-32	6.64E-31	Ecdysteroid UDP-glucosyltransferase; similar with UDPE_NPVSL
Px013884.1	2.4758	5.37E-18	3.43E-17	Probable cytochrome P450 9f2; similar with CP9F2_DROME
Px009900.2	2.4751	2.96E-29	2.55E-28	Microsomal glutathione S-transferase 1; similar with MGST1_HUMAN
Px015266.1	2.4663	4.55E-17	2.83E-16	Glutathione S-transferase omega-1; similar with GSTO1_HUMAN
Px014981.1	2.4259	0.00E+00	0.00E+00	Cytochrome P450 9e2; similar with CP9E2_BLAGE
Px015631.1	2.2963	1.88E-38	1.89E-37	Glutathione S-transferase 1; similar with GSTT1_MUSDO
Px005902.4	2.1318	2.80E-119	5.89E-118	Cytochrome P450 6B6; similar with CP6B6_HELAM
Px004854.1	2.1011	1.06E-15	6.27E-15	Ecdysteroid UDP-glucosyltransferase; similar with UDPE_NPVSL
Px014216.1	1.9094	1.32E-40	1.38E-39	Cytochrome P450 6B6; similar with CP6B6_HELAM
Px000872.2	1.8756	1.82E-09	7.93E-09	Ecdysteroid UDP-glucosyltransferase; similar with UDPE_NPVSL
Px000647.3	1.8232	6.21E-280	2.49E-278	UDP-glucuronosyltransferase 2B7; similar with UD2B7_HUMAN
Px011727.1	1.7734	6.73E-64	9.35E-63	Protein GCY; similar with GCY_YEAST
Px001113.1	1.6873	1.59E-11	7.83E-11	Ecdysteroid UDP-glucosyltransferase; similar with UDPE_NPVSL
Px004643.1	1.5882	6.58E-06	2.17E-05	Cytochrome P450 6B6; similar with CP6B6_HELAM
Px013958.1	1.5211	6.83E-145	1.68E-143	Cytochrome P450 6k1; similar with CP6K1_BLAGE
Px014982.1	1.4635	0.00E+00	0.00E+00	Probable cytochrome P450 9f2; similar with CP9F2_DROME
Px013203.1	1.4517	2.86E-06	9.77E-06	UDP-glucuronosyltransferase 2B2; similar with UD2B2_RAT
Px006106.1	1.3067	6.05E-183	1.77E-181	Glutathione S-transferase 1; similar with GSTT1_MANSE
Px006292.1	1.2679	2.20E-13	1.18E-12	Ecdysteroid UDP-glucosyltransferase; similar with UDPE_NPVSL
Px017253.1	1.2169	4.53E-37	4.47E-36	Cytochrome P450 6B2; similar with CP6B2_HELAM

Px000039.1	1.1965	1.40E-231	4.93E-230	UDP-glucuronosyltransferase 1-8; similar with UD18_RAT
Px003005.1	1.1775	9.24E-16	5.47E-15	Trans-1,2-dihydrobenzene-1,2-diol dehydrogenase
Px015896.1	1.1494	3.63E-206	1.14E-204	Glutathione S-transferase 1-1; similar with GSTT1_DROME
Px001164.1	1.1390	6.83E-117	1.42E-115	UDP-glucuronosyltransferase; similar with UGT3_PLEPL
Px009675.1	1.1334	1.86E-96	3.40E-95	UDP-glucuronosyltransferase 1-2; similar with UD12_MOUSE
Px010078.3	1.1245	3.16E-85	5.30E-84	Glutathione S-transferase 1; similar with GSTT1_MANSE
Px015080.1	1.1199	3.36E-06	1.14E-05	Ecdysteroid UDP-glucosyltransferase; similar with UDPE_NPVMB
Px005900.1	1.0773	1.30E-16	7.94E-16	Cytochrome P450 6B2; similar with CP6B2_HELAM
Px011285.1	1.0624	2.26E-250	8.22E-249	UDP-glucuronosyltransferase 2B4; similar with UD2B4_HUMAN
Px012288.2	1.0131	1.22E-06	4.30E-06	Glucose-fructose oxidoreductase domain-containing protein 1

Involved in drug metabolism - cytochrome P450

Px009572.1	9.2901	5.34E-07	1.94E-06	Cytochrome P450 6B1; similar with CP6B1_PAPPO
Px012208.1	3.1992	4.12E-29	3.54E-28	Probable flavin-containing monoamine oxidase A
Px002723.1	2.9848	0.00E+00	0.00E+00	Cytochrome P450 6B7; similar with CP6B7_HELAM
Px014883.1	2.5298	7.38E-32	6.64E-31	Ecdysteroid UDP-glucosyltransferase; similar with UDPE_NPVSL
Px013884.1	2.4758	5.37E-18	3.43E-17	Probable cytochrome P450 9f2; similar with CP9F2_DROME
Px009900.2	2.4751	2.96E-29	2.55E-28	Microsomal glutathione S-transferase 1; similar with MGST1_HUMAN
Px015266.1	2.4663	4.55E-17	2.83E-16	Glutathione S-transferase omega-1; similar with GSTO1_HUMAN
Px014981.1	2.4259	0.00E+00	0.00E+00	Cytochrome P450 9e2; similar with CP9E2_BLAGE
Px015631.1	2.2963	1.88E-38	1.89E-37	Glutathione S-transferase 1; similar with GSTT1_MUSDO
Px005902.4	2.1318	2.80E-119	5.89E-118	Cytochrome P450 6B6; similar with CP6B6_HELAM
Px004854.1	2.1011	1.06E-15	6.27E-15	Ecdysteroid UDP-glucosyltransferase; similar with UDPE_NPVSL
Px014216.1	1.9094	1.32E-40	1.38E-39	Cytochrome P450 6B6; similar with CP6B6_HELAM
Px000872.2	1.8756	1.82E-09	7.93E-09	Ecdysteroid UDP-glucosyltransferase; similar with UDPE_NPVSL
Px000647.3	1.8232	6.21E-280	2.49E-278	UDP-glucuronosyltransferase 2B7; similar with UD2B7_HUMAN
Px001113.1	1.6873	1.59E-11	7.83E-11	Ecdysteroid UDP-glucosyltransferase; similar with UDPE_NPVSL
Px004643.1	1.5882	6.58E-06	2.17E-05	Cytochrome P450 6B6; similar with CP6B6_HELAM
Px013958.1	1.5211	6.83E-145	1.68E-143	Cytochrome P450 6k1; similar with CP6K1_BLAGE
Px014982.1	1.4635	0.00E+00	0.00E+00	Probable cytochrome P450 9f2; similar with CP9F2_DROME
Px013203.1	1.4517	2.86E-06	9.77E-06	UDP-glucuronosyltransferase 2B2; similar with UD2B2_RAT
Px006106.1	1.3067	6.05E-183	1.77E-181	Glutathione S-transferase 1; similar with GSTT1_MANSE
Px006292.1	1.2679	2.20E-13	1.18E-12	Ecdysteroid UDP-glucosyltransferase; similar with UDPE_NPVSL
Px017253.1	1.2169	4.53E-37	4.47E-36	Cytochrome P450 6B2; similar with CP6B2_HELAM
Px011620.2	1.2137	5.39E-29	4.60E-28	Flavin-containing monooxygenase FMO GS-OX3
Px000039.1	1.1965	1.40E-231	4.93E-230	UDP-glucuronosyltransferase 1-8; similar with UD18_RAT
Px015896.1	1.1494	3.63E-206	1.14E-204	Glutathione S-transferase 1-1; similar with GSTT1_DROME
Px011987.1	1.1403	4.97E-07	1.81E-06	Amine oxidase [flavin-containing] B; similar with AOFB_MOUSE
Px001164.1	1.1390	6.83E-117	1.42E-115	UDP-glucuronosyltransferase; similar with UGT3_PLEPL
Px009675.1	1.1334	1.86E-96	3.40E-95	UDP-glucuronosyltransferase 1-2; similar with UD12_MOUSE
Px010078.3	1.1245	3.16E-85	5.30E-84	Glutathione S-transferase 1; similar with GSTT1_MANSE
Px015080.1	1.1199	3.36E-06	1.14E-05	Ecdysteroid UDP-glucosyltransferase; similar with UDPE_NPVMB
Px005900.1	1.0773	1.30E-16	7.94E-16	Cytochrome P450 6B2; similar with CP6B2_HELAM
Px011285.1	1.0624	2.26E-250	8.22E-249	UDP-glucuronosyltransferase 2B4; similar with UD2B4_HUMAN

Involved in hematopoietic cell lineage

Px013761.3	9.0724	2.99E-08	1.19E-07	Peroxidase; similar with PERO_DROME
Px010718.1	8.1236	8.30E-05	2.43E-04	B-cell receptor CD22; similar with CD22_HUMAN
Px001707.1	5.4057	1.10E-120	2.34E-119	Membrane alanyl aminopeptidase; similar with AMPM_MANSE
Px008276.1	4.6077	0.00E+00	0.00E+00	Membrane alanyl aminopeptidase; similar with AMPM_HELVI
Px006617.2	3.3534	1.05E-31	9.40E-31	Chorion peroxidase; similar with PERC_DROME
Px008277.1	3.3443	1.09E-141	2.62E-140	Membrane alanyl aminopeptidase; similar with AMPM_MANSE
Px007137.2	3.1853	1.13E-132	2.54E-131	Peroxidase; similar with PERO_DROME
Px003755.1	2.6780	0.00E+00	0.00E+00	Membrane alanyl aminopeptidase; similar with AMPM_MANSE

Px000184.1	2.5516	9.19E-59	1.19E-57	CD9 antigen; similar with CD9_RAT
Px008866.1	2.4968	7.39E-17	4.55E-16	Chorion peroxidase; similar with PERC_DROME
Px006338.1	2.4923	6.30E-36	6.05E-35	Carboxypeptidase N subunit 2; similar with CPN2_MOUSE
Px001708.1	2.3708	0.00E+00	0.00E+00	Aminopeptidase N; similar with AMPN_PLUXY
Px001705.1	2.3213	0.00E+00	0.00E+00	Membrane alanyl aminopeptidase; similar with AMPM_MANSE
Px009761.1	2.2693	1.37E-127	3.02E-126	Plasma glutamate carboxypeptidase; similar with PGCP_XENLA
Px008278.1	2.2655	4.42E-37	4.36E-36	Membrane alanyl aminopeptidase; similar with AMPM_MANSE
Px005645.3	2.2566	2.97E-11	1.44E-10	Integrin alpha-PS3; similar with ITA3_DROME
Px014250.1	2.0028	2.31E-65	3.24E-64	Putative uncharacterized protein; similar with D6WG20_TRICA
Px003754.1	1.9228	1.67E-147	4.13E-146	Membrane alanyl aminopeptidase; similar with AMPM_MANSE
Px003753.1	1.7587	0.00E+00	0.00E+00	Membrane alanyl aminopeptidase; similar with AMPM_HELVI
Px015891.1	1.3621	4.81E-87	8.23E-86	Peroxidase; similar with PERO_DROME
Px002532.1	1.2056	1.34E-124	2.93E-123	Peroxidasin; similar with PXDN_DROME
Px005106.1	1.1107	1.62E-25	1.28E-24	Aminopeptidase N; similar with AMPN_CHICK
Px008080.1	1.0773	1.70E-05	5.37E-05	Lysosome membrane protein 2; similar with SCRB2_HUMAN
Px007394.2	1.0691	1.25E-39	1.29E-38	Peroxidasin homolog; similar with PXDN_MOUSE
Px011170.1	-2.2057	2.29E-18	1.48E-17	Hemicentin-1; similar with HMCN1_HUMAN

Involved in Tyrosine metabolism

Px013761.3	9.0724	2.99E-08	1.19E-07	Peroxidase; similar with PERO_DROME
Px005646.1	4.6071	1.01E-58	1.31E-57	Nose resistant to fluoxetine protein 6; similar with NRF6_CAEEL
Px000359.2	4.0159	3.40E-22	2.47E-21	Alpha-methyldopa hypersensitive protein; similar with L2AM_DROME
Px003528.3	3.9017	3.38E-20	2.32E-19	Nose resistant to fluoxetine protein 6; similar with NRF6_CAEEL
Px011202.1	3.5882	8.25E-143	2.01E-141	Aromatic-L-amino-acid decarboxylase; similar with DDC_MANSE
Px002585.2	3.5836	3.76E-53	4.58E-52	Nose resistant to fluoxetine protein 6; similar with NRF6_CAEEL
Px006617.2	3.3534	1.05E-31	9.40E-31	Chorion peroxidase; similar with PERC_DROME
Px009569.1	3.3179	0.00E+00	0.00E+00	Trypsin CFT-1; similar with TRYP_CHOFU
Px012208.1	3.1992	4.12E-29	3.54E-28	Probable flavin-containing monoamine oxidase A
Px007137.2	3.1853	1.13E-132	2.54E-131	Peroxidase; similar with PERO_DROME
Px005417.1	2.8142	7.10E-18	4.51E-17	Nose resistant to fluoxetine protein 6; similar with NRF6_CAEEL
Px015388.1	2.7618	1.35E-08	5.50E-08	Nose resistant to fluoxetine protein 6; similar with NRF6_CAEEL
Px002274.1	2.7121	0.00E+00	0.00E+00	Phenoloxidase subunit 2; similar with PRP2_BOMMO
Px005418.1	2.6622	6.87E-07	2.48E-06	Nose resistant to fluoxetine protein 6; similar with NRF6_CAEEL
Px008866.1	2.4968	7.39E-17	4.55E-16	Chorion peroxidase; similar with PERC_DROME
Px004684.1	1.7858	0.00E+00	0.00E+00	Tyrosine 3-monooxygenase; similar with TY3H_DROME
Px013101.1	1.6622	3.21E-06	1.09E-05	Nose resistant to fluoxetine protein 6; similar with NRF6_CAEEL
Px011624.1	1.5666	4.24E-07	1.55E-06	Nose resistant to fluoxetine protein 6; similar with NRF6_CAEEL
Px010498.1	1.5315	2.77E-59	3.62E-58	Fumarylacetoacetate hydrolase domain-containing protein 2
Px015891.1	1.3621	4.81E-87	8.23E-86	Peroxidase; similar with PERO_DROME
Px014079.1	1.2713	4.69E-10	2.13E-09	Nose resistant to fluoxetine protein 6; similar with NRF6_CAEEL
Px002532.1	1.2056	1.34E-124	2.93E-123	Peroxidasin; similar with PXDN_DROME
Px001224.1	1.1741	3.07E-05	9.43E-05	Maleylacetoacetate isomerase; similar with MAAI_MOUSE
Px011987.1	1.1403	4.97E-07	1.81E-06	Amine oxidase [flavin-containing] B; similar with AOFB_MOUSE
Px007394.2	1.0691	1.25E-39	1.29E-38	Peroxidasin homolog; similar with PXDN_MOUSE
Px011234.1	1.0051	2.16E-04	5.97E-04	Nose resistant to fluoxetine protein 6; similar with NRF6_CAEEL
Px013191.1	-1.2294	5.69E-11	2.72E-10	Aromatic-L-amino-acid decarboxylase; similar with DDC_RAT
Px007031.1	-1.2120	0.00E+00	0.00E+00	Basic juvenile hormone-suppressible protein 2; similar with BJSB2_TRINI
Px016931.1	-1.0939	3.25E-26	2.60E-25	Methyltransferase-like protein 6; similar with METL6_HUMAN

Involved in mineral absorption

Px013802.1	8.5527	1.46E-08	5.93E-08	Sodium/potassium-transporting ATPase subunit alpha-2
Px010354.2	8.4829	6.15E-08	2.39E-07	Envelope fusion protein; similar with ENV_NPVLD
Px005784.1	3.8214	5.98E-16	3.58E-15	Ferritin, chloroplastic; similar with FRI_MALXI
Px014201.1	3.6008	7.33E-11	3.48E-10	Sodium/potassium-transporting ATPase subunit alpha-2

Px014012.2	3.4448	3.32E-59	4.34E-58	Proton-coupled folate transporter; similar with PCFT_XENLA
Px004813.1	3.1234	8.30E-302	3.49E-300	High affinity copper uptake protein 1; similar with COPT1_HUMAN
Px004604.5	2.2867	3.97E-06	1.34E-05	Envelope fusion protein; similar with ENV_NPVLD
Px003205.1	2.1414	1.92E-12	9.88E-12	Envelope fusion protein; similar with ENV_NPVLD
Px006683.1	2.1362	2.49E-07	9.29E-07	Envelope fusion protein; similar with ENV_NPVLD
Px004843.1	1.8517	8.05E-08	3.11E-07	ORF; similar with Q8I7Q1_DROME
Px018019.1	1.8031	1.95E-05	6.12E-05	Putative uncharacterized protein; similar with E2ARC4_9HYME
Px007238.1	1.7626	1.98E-23	1.48E-22	Solute carrier family 46 member 3; similar with S46A3_CHICK
Px013208.1	1.7432	6.70E-193	2.04E-191	Transferrin; similar with TRF_MANSE
Px015615.1	1.7053	2.16E-07	8.10E-07	Proton-coupled folate transporter; similar with PCFT_DANRE
Px004446.3	1.6970	2.01E-45	2.23E-44	Copper-transporting ATPase 2; similar with ATP7B_MOUSE
Px005491.1	1.5695	1.70E-21	1.22E-20	High affinity copper uptake protein 1; similar with COPT1_HUMAN
Px012191.1	1.4028	2.38E-208	7.61E-207	Sodium/potassium-transporting ATPase subunit alpha-1
Px003168.1	1.3070	0.00E+00	0.00E+00	Soma ferritin; similar with FRIS_LYMST
Px011103.1	1.2919	1.27E-08	5.22E-08	Transferrin; similar with TRF_BLADI
Px003167.1	1.2675	3.51E-157	9.10E-156	Ferritin subunit; similar with FRI_AEDAE
Px014014.2	1.2587	6.46E-21	4.55E-20	Proton-coupled folate transporter; similar with PCFT_DANRE
Px014013.2	1.2197	1.13E-07	4.34E-07	Proton-coupled folate transporter; similar with PCFT_DANRE
Px014019.1	1.1874	2.13E-08	8.57E-08	Solute carrier family 46 member 3; similar with S46A3_CHICK
Px015324.1	1.1450	2.99E-40	3.10E-39	Proton-coupled folate transporter; similar with PCFT_XENLA
Px011027.1	1.1279	4.95E-11	2.37E-10	ORF; similar with Q8I7Q1_DROME
Px005939.1	1.0952	5.25E-05	1.58E-04	ORF; similar with Q8I7Q1_DROME
Px004243.3	1.0071	7.75E-78	1.23E-76	Ferritin subunit; similar with FRI_AEDAE
Px009302.2	-1.5524	1.01E-125	2.21E-124	Putative uncharacterized protein; similar with D7GYL0_TRICA
Px014011.1	-1.2447	1.09E-04	3.13E-04	Proton-coupled folate transporter; similar with PCFT_XENLA

Involved in pertussis

Px015514.1	4.2671	1.44E-18	9.38E-18	Serine/threonine-protein kinase pelle; similar with KPEL_DROME
Px009987.1	2.8978	1.47E-38	1.48E-37	Caspase-1; similar with CASP1_DROME
Px010499.1	2.3073	3.86E-59	5.04E-58	Serine/threonine-protein kinase pelle; similar with KPEL_DROME
Px012279.1	2.1704	1.43E-32	1.30E-31	Caltractin; similar with CATR_SCHDU
Px007006.1	1.9791	1.83E-24	1.40E-23	EF-hand domain-containing protein CG10641; similar with EFHD_DROME
Px003725.1	1.9489	6.60E-74	1.01E-72	Interleukin-1 receptor-associated kinase 4; similar with IRAK4_BOVIN
Px004376.3	1.8321	1.31E-14	7.38E-14	Leucine-rich repeat-containing protein 34; similar with LRC34_MOUSE
Px006496.1	1.7584	1.39E-18	9.08E-18	Cytochrome b5 domain-containing protein 1; similar with CB5D1_HUMAN
Px001400.1	1.7038	5.86E-54	7.19E-53	Calmodulin; similar with CALM_PLECO
Px004977.1	1.6799	1.55E-260	5.85E-259	Transcription factor AP-1; similar with JRA_DROME
Px001556.1	1.6061	8.94E-117	1.85E-115	Serine protease easter; similar with EAST_DROME
Px012395.1	1.2873	6.63E-51	7.90E-50	Integrin beta-PS; similar with ITBX_DROME
Px012604.1	1.2789	4.84E-08	1.90E-07	Integrin beta; similar with E9GI17_DAPPU
Px015363.1	1.2365	5.17E-05	1.55E-04	Slit homolog 3 protein; similar with SLIT3_MOUSE
Px009367.1	1.1816	9.43E-06	3.07E-05	Troponin C, isoform 1; similar with TNNC1_DROME
Px010253.1	1.1621	1.06E-107	2.11E-106	Unknown functio
Px012423.1	1.1613	1.21E-06	4.25E-06	TNF receptor-associated factor 6; similar with TRAF6_XENTR
Px015782.1	1.1185	1.11E-14	6.29E-14	Calmodulin; similar with CALM_PLAFA
Px008096.1	1.0350	1.59E-05	5.03E-05	TNF receptor-associated factor 6-B; similar with TRF6B_XENLA
Px005668.1	-2.2447	8.13E-05	2.38E-04	NGFI-A-binding protein homolog; similar with NAB_DROME
Px011260.1	-1.6757	5.96E-122	1.28E-120	Troponin C, isoform 2; similar with TNNC2_DROME
Px002799.3	-1.4322	0.00E+00	0.00E+00	Troponin C, isoform 1; similar with TNNC1_DROME

Involved in ascorbate and aldarate metabolism

Px014883.1	2.5298	7.38E-32	6.64E-31	Ecdysteroid UDP-glucosyltransferase; similar with UDPE_NPVSL
Px004854.1	2.1011	1.06E-15	6.27E-15	Ecdysteroid UDP-glucosyltransferase; similar with UDPE_NPVSL
Px000872.2	1.8756	1.82E-09	7.93E-09	Ecdysteroid UDP-glucosyltransferase; similar with UDPE_NPVSL

Px000647.3	1.8232	6.21E-280	2.49E-278	UDP-glucuronosyltransferase 2B7; similar with UD2B7_HUMAN
Px000123.1	1.7598	0.00E+00	0.00E+00	Putative aldehyde dehydrogenase family 7 member A1 homolog
Px001113.1	1.6873	1.59E-11	7.83E-11	Ecdysteroid UDP-glucosyltransferase; similar with UDPE_NPVSL
Px013203.1	1.4517	2.86E-06	9.77E-06	UDP-glucuronosyltransferase 2B2; similar with UD2B2_RAT
Px006848.1	1.2745	1.88E-14	1.05E-13	UDP-glucose 6-dehydrogenase; similar with UGDH_DROME
Px006292.1	1.2679	2.20E-13	1.18E-12	Ecdysteroid UDP-glucosyltransferase; similar with UDPE_NPVSL
Px012877.1	1.2309	5.17E-17	3.21E-16	Inositol oxygenase; similar with MIOX_DANRE
Px002733.2	1.2093	0.00E+00	0.00E+00	Aldehyde dehydrogenase, dimeric NADP-preferring
Px000039.1	1.1965	1.40E-231	4.93E-230	UDP-glucuronosyltransferase 1-8; similar with UD18_RAT
Px006815.1	1.1714	0.00E+00	0.00E+00	Regucalcin; similar with RGN_DANRE
Px001164.1	1.1390	6.83E-117	1.42E-115	UDP-glucuronosyltransferase; similar with UGT3_PLEPL
Px012878.1	1.1388	3.00E-40	3.10E-39	Inositol oxygenase; similar with MIOX_MOUSE
Px009675.1	1.1334	1.86E-96	3.40E-95	UDP-glucuronosyltransferase 1-2; similar with UD12_MOUSE
Px015080.1	1.1199	3.36E-06	1.14E-05	Ecdysteroid UDP-glucosyltransferase; similar with UDPE_NPVMB
Px011285.1	1.0624	2.26E-250	8.22E-249	UDP-glucuronosyltransferase 2B4; similar with UD2B4_HUMAN
Px010576.1	1.0580	2.09E-20	1.44E-19	Inositol oxygenase; similar with MIOX_DANRE
Px016791.1	1.0335	1.04E-22	7.66E-22	Inositol oxygenase; similar with MIOX_DANRE

Table D. A list of DEGs enriched in the *P. xylostella* pathways at 48 hpt_I versus 48 hpt_C.

Gene ID	log_2 R	P-value	FDR	Annotation
**Involved in amoebiasis**				
Px007765.1	10.6239	6.00E-05	1.81E-04	Serine protease inhibitor 3/4; similar with SPI3_LONON
Px008374.1	10.1228	1.11E-04	3.21E-04	Phosphatidylinositol-4,5-bisphosphate 3-kinase catalytic subunit delta isoform
Px001465.1	9.3895	9.76E-10	4.84E-09	Putative uncharacterized protein; similar with D6WLC4_TRICA
Px002641.1	4.0559	2.16E-109	6.30E-108	Alpha-actinin-2; similar with ACTN2_BOVIN
Px001917.1	3.8840	8.31E-08	3.47E-07	Receptor-mediated endocytosis protein 6 homolog
Px014766.1	3.1608	9.70E-10	4.81E-09	Metal transporter CNNM4; similar with CNNM4_MOUSE
Px009397.1	2.9247	1.45E-06	5.37E-06	Translation initiation factor IF-2; similar with IF2_CHLPD
Px005596.1	2.7721	1.45E-36	1.92E-35	FERM and PDZ domain-containing protein 4
Px011821.1	2.6934	2.68E-04	7.29E-04	Reticulon-4-interacting protein 1, mitochondrial
Px004755.2	2.6104	2.23E-21	1.98E-20	Putative uncharacterized protein; similar with D6W7K2_TRICA
Px014818.1	2.5961	6.82E-07	2.61E-06	Ecdysteroid 22-phosphate; similar with Q0PCR8_BOMMO
Px007895.2	2.5462	3.78E-150	1.38E-148	Peritrophic matrix insect intestinal mucin; similar with Q8ISS2_PLUXY
Px008372.1	2.4177	3.42E-08	1.48E-07	Phosphatidylinositol-4,5-bisphosphate 3-kinase catalytic subunit beta isoform
Px010041.2	2.3397	1.97E-05	6.34E-05	Circadian clock-controlled protein; similar with CCCP_DROME
Px009784.1	2.2374	0.00E+00	0.00E+00	Ribosome-binding protein 1; similar with RRBP1_HUMAN
Px009987.1	2.1996	6.07E-12	3.49E-11	Caspase-1; similar with CASP1_DROME
Px010627.1	2.1698	0.00E+00	0.00E+00	Wing disc-specific protein; similar with Q8WPH2_BOMMO
Px008413.1	2.1698	2.19E-06	7.93E-06	AGAP011937-PA; similar with Q7PKC7_ANOGA
Px007897.1	2.1651	9.44E-23	8.75E-22	Peritrophic matrix insect intestinal mucin; similar with Q8ISS2_PLUXY
Px014841.1	2.1518	5.47E-57	1.01E-55	Unknown functio
Px005969.1	2.1323	0.00E+00	0.00E+00	Apolipoprotein D; similar with APOD_MACFA
Px002309.1	2.1123	1.77E-05	5.73E-05	Putative uncharacterized protein; similar with E9IM57_SOLIN
Px007274.1	2.0866	3.84E-16	2.78E-15	Serpin B9; similar with SPB9_HUMAN
Px001443.1	2.0338	0.00E+00	0.00E+00	L-ascorbate oxidase; similar with ASO_CUCPM
Px004235.2	1.8977	7.45E-11	4.01E-10	Putative uncharacterized protein; similar with Q178N6_AEDAE
Px007273.1	1.8846	3.63E-31	4.31E-30	Plasminogen activator inhibitor 1; similar with PAI1_HUMAN
Px015898.1	1.8788	0.00E+00	0.00E+00	Putative farnesoic acid O-methyl transferase
Px011646.1	1.8633	2.33E-07	9.35E-07	GA10261; similar with Q29B54_DROPS
Px006878.1	1.7943	2.54E-04	6.97E-04	Protein kinase C, brain isozyme; similar with KPC1_DROME
Px004991.4	1.7588	4.01E-30	4.65E-29	Tyrosine-protein phosphatase non-receptor type 4
Px001831.1	1.7548	1.93E-08	8.52E-08	Headcase protein homolog; similar with HDC_HUMAN
Px011466.1	1.7416	9.61E-19	7.76E-18	Putative uncharacterized protein; similar with F0Y4G7_9STRA
Px000548.1	1.7364	0.00E+00	0.00E+00	Alpha-actinin, sarcomeric; similar with ACTN_DROME
Px003835.1	1.7362	1.33E-13	8.38E-13	Putative uncharacterized protein; similar with B0WNP5_CULQU
Px007399.1	1.6693	1.30E-29	1.49E-28	Putative uncharacterized protein; similar with D6WDQ4_TRICA
Px015729.1	1.6242	3.15E-08	1.37E-07	Vinculin; similar with VINC_DROME
Px009510.1	1.5900	2.13E-12	1.26E-11	Cytosolic carboxypeptidase 1; similar with CBPC1_DANRE
Px013841.1	1.5661	5.52E-39	7.58E-38	Phytanoyl-CoA dioxygenase domain-containing protein 1
Px015087.3	1.5281	0.00E+00	0.00E+00	Glia-derived nexin; similar with GDN_HUMAN
Px008604.1	1.5243	7.49E-30	8.64E-29	AGAP011476-PA; similar with Q7Q343_ANOGA
Px012264.1	1.5180	4.16E-221	2.02E-219	Ejaculatory bulb-specific protein 3; similar with PEB3_DROME
Px011301.2	1.5122	5.33E-93	1.38E-91	Putative uncharacterized protein; similar with D6WH87_TRICA
Px009152.1	1.4996	5.17E-21	4.54E-20	Innexin inx7; similar with INX7_DROME
Px000651.1	1.4970	3.22E-13	1.99E-12	Extracellular domains-containing protein CG31004
Px006536.1	1.4850	0.00E+00	0.00E+00	HMG176; similar with Q0MTA5_HELAM
Px013635.1	1.4584	1.68E-22	1.54E-21	Extracellular domains-containing protein CG31004
Px000072.1	1.4166	5.91E-14	3.80E-13	Protein CLEC16A; similar with CL16A_HUMAN
Px001494.2	1.4053	0.00E+00	0.00E+00	Alaserpin; similar with SERA_MANSE
Px009586.1	1.4046	1.13E-06	4.22E-06	Phosphatidylinositol 3-kinase regulatory subunit gamma
Px001082.1	1.3854	0.00E+00	0.00E+00	Unknown functio

Px013721.1	1.3817	2.56E-15	1.79E-14	Endonuclease-reverse transcriptase; similar with D7F170_BOMMO
Px002670.1	1.3679	0.00E+00	0.00E+00	Apolipophorins; similar with APLP_MANSE
Px007271.1	1.3439	6.03E-169	2.36E-167	Serpin B9; similar with SPB9_HUMAN
Px012786.1	1.3299	0.00E+00	0.00E+00	GH11122; similar with B4JCY6_DROGR
Px013975.1	1.3267	2.86E-07	1.13E-06	Cuticlin-1; similar with E2BP69_9HYME
Px009301.1	1.3241	2.23E-45	3.46E-44	Beta-1,3-glucan-binding protein; similar with BGBP_BOMMO
Px017059.1	1.3144	3.09E-78	7.16E-77	Serpin B6; similar with SPB6_HUMAN
Px011737.1	1.3022	0.00E+00	0.00E+00	Neurofilament heavy polypeptide; similar with NFH_MOUSE
Px002835.1	1.2932	1.92E-04	5.37E-04	Fasciclin-1; similar with FAS1_SCHAM
Px002792.1	1.2920	7.53E-26	7.80E-25	Unknown functio
Px004419.1	1.2908	2.43E-07	9.72E-07	Putative uncharacterized protein GLEAN_08369
Px015492.1	1.2649	5.91E-22	5.35E-21	Protein halfway; similar with HFW1_DROPS
Px005140.1	1.2596	1.46E-06	5.39E-06	Focal adhesion kinase 1; similar with FAK1_RAT
Px002608.1	1.2583	1.73E-12	1.03E-11	Protein kinase DC2; similar with KDC2_DROME
Px008882.1	1.2281	6.01E-123	1.88E-121	Unknown functio
Px008031.1	1.1629	2.57E-04	7.03E-04	cAMP-dependent protein kinase catalytic subunit alpha
Px005739.1	1.1553	1.10E-15	7.78E-15	Putative uncharacterized protein; similar with B0W6E4_CULQU
Px015730.1	1.1397	0.00E+00	0.00E+00	Apolipophorins; similar with APLP_MANSE
Px001430.1	1.1298	0.00E+00	0.00E+00	Chitin deacetylase 1; similar with B2ZGH5_9NEOP
Px001080.1	1.1262	0.00E+00	0.00E+00	Unknown functio
Px005322.1	1.1251	2.50E-46	3.97E-45	Cuticle protein 19; similar with CU19_LOCMI
Px002640.1	1.1212	8.64E-275	4.94E-273	Alpha-actinin, sarcomeric; similar with ACTN_DROME
Px003409.1	1.1173	2.96E-04	8.01E-04	Protein toll; similar with TOLL_DROME
Px008393.1	1.1138	3.51E-06	1.24E-05	Nck-associated protein 5; similar with NCKP5_HUMAN
Px015010.1	1.0950	1.19E-43	1.79E-42	Heat shock protein beta-1; similar with HSPB1_CHICK
Px002572.2	1.0850	2.97E-25	3.01E-24	Large proline-rich protein bag6-A; similar with BAG6A_XENLA
Px015090.1	1.0837	2.60E-268	1.46E-266	Serpin B6; similar with SPB6_HUMAN
Px015092.1	1.0810	2.56E-59	4.92E-58	Unknown functio
Px014947.1	1.0681	1.22E-23	1.17E-22	Putative uncharacterized protein; similar with E2AAT5_9HYME
Px016246.1	-9.0336	3.73E-04	9.91E-04	Unknown functio
Px012920.1	-5.7371	1.31E-14	8.79E-14	Unknown functio
Px003251.1	-5.6346	2.17E-287	1.29E-285	Putative cuticle protein; similar with C0H6E8_BOMMO
Px010970.1	-5.1269	1.33E-210	6.09E-209	Cuticle protein; similar with CU01_ANOGA
Px001530.1	-5.1032	8.45E-92	2.16E-90	Neurofilament heavy polypeptide; similar with NFH_MOUSE
Px001327.2	-4.9271	1.00E-22	9.26E-22	Unknown functio
Px014502.2	-4.9177	1.09E-29	1.26E-28	Putative uncharacterized protein; similar with B0W8F8_CULQU
Px012644.1	-4.8891	1.72E-84	4.13E-83	Filamentous hemagglutinin; similar with FHAB_BORPE
Px008648.1	-4.8302	1.88E-14	1.25E-13	Larval/pupal rigid cuticle protein 66; similar with CU66_HYACE
Px005321.1	-4.6028	2.84E-34	3.61E-33	Cuticle protein 19; similar with CU19_LOCMI
Px014251.1	-4.3113	1.63E-18	1.30E-17	Putative uncharacterized protein; similar with D6WG20_TRICA
Px005318.1	-4.2809	2.97E-43	4.42E-42	Cuticle protein 19; similar with CU19_LOCMI
Px016161.1	-4.1521	1.00E-98	2.73E-97	Neurofilament heavy polypeptide; similar with NFH_RAT
Px010665.1	-3.9457	7.76E-14	4.96E-13	Putative uncharacterized protein; similar with E3WXH7_ANODA
Px017104.1	-3.9233	1.10E-16	8.19E-16	Neurofilament heavy polypeptide; similar with NFH_MOUSE
Px014245.1	-3.5851	1.97E-19	1.63E-18	Unknown functio
Px001674.1	-3.5040	1.12E-30	1.32E-29	Cuticular protein CPG7; similar with B2DBG1_9NEOP
Px012919.2	-3.4152	4.85E-05	1.49E-04	Cuticle protein 19; similar with CU19_LOCMI
Px005323.1	-3.2863	1.82E-23	1.73E-22	Cuticle protein 19; similar with CU19_LOCMI
Px008500.1	-3.0987	1.00E-124	3.20E-123	Pupal cuticle protein; similar with CUPP_BOMMO
Px003249.1	-2.7665	0.00E+00	0.00E+00	Larval cuticle protein LCP-22; similar with CU22_BOMMO
Px008654.1	-2.7571	5.40E-28	5.90E-27	Pupal cuticle protein Edg-84A; similar with CUP8_DROME
Px007290.1	-2.6760	7.93E-134	2.68E-132	Cuticle protein; similar with CU01_ANOGA
Px009321.1	-2.6343	1.76E-19	1.45E-18	Cuticle protein 3; similar with CU03_LONON
Px002841.1	-2.5840	3.11E-15	2.17E-14	Putative uncharacterized protein GLEAN_07427

Px003254.3	-2.4993	0.00E+00	0.00E+00	Larval cuticle protein LCP-17; similar with CU17_BOMMO
Px014252.1	-2.2750	5.84E-15	3.99E-14	Putative uncharacterized protein; similar with D6WG20_TRICA
Px003252.2	-2.2721	0.00E+00	0.00E+00	Larval cuticle protein LCP-30; similar with CU30_BOMMO
Px004340.1	-2.1994	2.01E-05	6.48E-05	Uncharacterized protein; similar with F1SF10_PIG
Px003681.1	-2.1521	5.92E-15	4.05E-14	Cuticle protein 19; similar with CU19_LOCMI
Px013556.1	-1.9665	0.00E+00	0.00E+00	Flexible cuticle protein 12; similar with CU12_HYACE
Px001064.1	-1.9506	2.87E-247	1.54E-245	Pupal cuticle protein Edg-84A; similar with CUP8_DROME
Px008653.1	-1.9082	3.21E-20	2.74E-19	Cuticle protein; similar with CU01_ANOGA
Px005288.1	-1.8733	2.28E-12	1.35E-11	Putative cuticle protein; similar with Q9U504_MANSE
Px003259.1	-1.8425	5.66E-111	1.68E-109	Endocuticle structural glycoprotein SgAbd-2
Px005317.2	-1.8082	1.04E-14	6.99E-14	Cuticle protein 7; similar with CU07_LOCMI
Px010337.1	-1.7509	4.56E-46	7.17E-45	Cuticular protein CPR77; similar with B2DBI2_9NEOP
Px002615.1	-1.7185	7.88E-24	7.62E-23	C-type lectin, galactose-binding (AGAP010196-PA)
Px005891.1	-1.7086	5.24E-10	2.66E-09	Putative cuticle protein; similar with Q9U509_MANSE
Px001065.1	-1.6858	5.33E-19	4.33E-18	Cuticle protein; similar with CU01_ANOGA
Px008954.1	-1.6672	1.06E-274	6.05E-273	Protein MICAL-2; similar with MICA2_HUMAN
Px009941.1	-1.6603	3.34E-04	8.97E-04	Putative uncharacterized protein; similar with E2A5A2_9HYME
Px017593.1	-1.6187	5.41E-41	7.74E-40	Putative uncharacterized protein; similar with D6X1E0_TRICA
Px000919.1	-1.5620	9.67E-48	1.58E-46	Enhancer of split m4 protein; similar with ESM4_DROME
Px014262.1	-1.5611	1.89E-59	3.65E-58	Putative cuticle protein; similar with C0H6H1_BOMMO
Px014536.1	-1.5191	8.05E-25	8.03E-24	Unknown functio
Px001789.1	-1.4859	7.13E-28	7.75E-27	Protein toll; similar with TOLL_DROME
Px011630.1	-1.4823	5.20E-09	2.43E-08	Cuticle protein 7; similar with CU07_LOCMI
Px005320.1	-1.4616	1.11E-148	4.03E-147	Cuticle protein 7; similar with CU07_LOCMI
Px003255.1	-1.4547	0.00E+00	0.00E+00	Larval cuticle protein LCP-17; similar with CU17_BOMMO
Px003245.1	-1.3445	1.84E-227	9.18E-226	Larval cuticle protein LCP-22; similar with CU22_BOMMO
Px013555.1	-1.2894	4.85E-254	2.64E-252	Flexible cuticle protein 12; similar with CU12_HYACE
Px003729.1	-1.2580	4.23E-75	9.56E-74	Venom allergen 3; similar with VA3_SOLIN
Px014247.3	-1.2306	0.00E+00	0.00E+00	Cuticular protein CPR77; similar with B2DBI2_9NEOP
Px008408.1	-1.2190	8.68E-14	5.53E-13	Ras-related protein RabJ; similar with E2C0I8_9HYME
Px008652.2	-1.2135	3.96E-44	6.01E-43	Cuticle protein; similar with CU01_ANOGA
Px010900.1	-1.1915	0.00E+00	0.00E+00	Unknown functio
Px008680.1	-1.1712	2.29E-04	6.33E-04	Putative dsRNase; similar with B7VCC3_SPOLI
Px016485.1	-1.1684	5.49E-15	3.77E-14	Pupal cuticle protein; similar with CUPP_BOMMO
Px001226.2	-1.1578	4.15E-18	3.25E-17	Collagen alpha-2(I) chain; similar with CO1A2_RAT
Px015352.3	-1.1051	3.91E-06	1.38E-05	Putative uncharacterized protein; similar with E3X258_ANODA
Px015226.1	-1.0508	9.46E-128	3.07E-126	GI23113; similar with B4KCJ7_DROMO
Px014261.1	-1.0402	6.99E-38	9.43E-37	Putative cuticle protein; similar with C0H6H2_BOMMO
Px009438.1	-1.0079	2.26E-54	4.06E-53	Arginase, non-hepatic 2; similar with ARGN2_XENLA

Involved in vibrio cholerae infection

Px003126.1	9.7828	1.11E-04	3.21E-04	Adenylate cyclase type 3; similar with ADCY3_MOUSE
Px001465.1	9.3895	9.76E-10	4.84E-09	Putative uncharacterized protein; similar with D6WLC4_TRICA
Px008411.1	8.8650	6.00E-05	1.81E-04	Adenylate cyclase type 3; similar with ADCY3_MOUSE
Px001917.1	3.8840	8.31E-08	3.47E-07	Receptor-mediated endocytosis protein 6 homolog
Px014766.1	3.1608	9.70E-10	4.81E-09	Metal transporter CNNM4; similar with CNNM4_MOUSE
Px009397.1	2.9247	1.45E-06	5.37E-06	Translation initiation factor IF-2; similar with IF2_CHLPD
Px011821.1	2.6934	2.68E-04	7.29E-04	Reticulon-4-interacting protein 1, mitochondrial
Px004755.2	2.6104	2.23E-21	1.98E-20	Putative uncharacterized protein; similar with D6W7K2_TRICA
Px014818.1	2.5961	6.82E-07	2.61E-06	Ecdysteroid 22-phosphate; similar with Q0PCR8_BOMMO
Px007895.2	2.5462	3.78E-150	1.38E-148	Peritrophic matrix insect intestinal mucin
Px010041.2	2.3397	1.97E-05	6.34E-05	Circadian clock-controlled protein; similar with CCCP_DROME
Px009784.1	2.2374	0.00E+00	0.00E+00	Ribosome-binding protein 1; similar with RRBP1_HUMAN
Px008413.1	2.1698	2.19E-06	7.93E-06	AGAP011937-PA; similar with Q7PKC7_ANOGA

Px007897.1	2.1651	9.44E-23	8.75E-22	Peritrophic matrix insect intestinal mucin
Px014841.1	2.1518	5.47E-57	1.01E-55	Unknown functio
Px005969.1	2.1323	0.00E+00	0.00E+00	Apolipoprotein D; similar with APOD_MACFA
Px002309.1	2.1123	1.77E-05	5.73E-05	Putative uncharacterized protein; similar with E9IM57_SOLIN
Px001443.1	2.0338	0.00E+00	0.00E+00	L-ascorbate oxidase; similar with ASO_CUCPM
Px004235.2	1.8977	7.45E-11	4.01E-10	Putative uncharacterized protein; similar with Q178N6_AEDAE
Px015898.1	1.8788	0.00E+00	0.00E+00	Putative farnesoic acid O-methyl transferase
Px016879.1	1.8644	1.62E-08	7.21E-08	Disks large homolog 5; similar with DLG5_HUMAN
Px011646.1	1.8633	2.33E-07	9.35E-07	GA10261; similar with Q29B54_DROPS
Px006878.1	1.7943	2.54E-04	6.97E-04	Protein kinase C, brain isozyme; similar with KPC1_DROME
Px007889.1	1.7872	5.41E-05	1.65E-04	V-type proton ATPase 116 kDa subunit a isoform 1
Px004991.4	1.7588	4.01E-30	4.65E-29	Tyrosine-protein phosphatase non-receptor type 4
Px001831.1	1.7548	1.93E-08	8.52E-08	Headcase protein homolog; similar with HDC_HUMAN
Px011466.1	1.7416	9.61E-19	7.76E-18	Putative uncharacterized protein; similar with F0Y4G7_9STRA
Px003835.1	1.7362	1.33E-13	8.38E-13	Putative uncharacterized protein; similar with B0WNP5_CULQU
Px003751.1	1.6696	1.67E-28	1.85E-27	V-type proton ATPase 116 kDa subunit a isoform 1
Px007399.1	1.6693	1.30E-29	1.49E-28	Putative uncharacterized protein; similar with D6WDQ4_TRICA
Px009510.1	1.5900	2.13E-12	1.26E-11	Cytosolic carboxypeptidase 1; similar with CBPC1_DANRE
Px013582.1	1.5749	3.43E-09	1.62E-08	TBC1 domain family member 24; similar with TBC24_XENTR
Px010270.1	1.5696	0.00E+00	0.00E+00	Putative protein disulfide-isomerase C1F5.02
Px013841.1	1.5661	5.52E-39	7.58E-38	Phytanoyl-CoA dioxygenase domain-containing protein 1
Px008604.1	1.5243	7.49E-30	8.64E-29	AGAP011476-PA; similar with Q7Q343_ANOGA
Px012264.1	1.5180	4.16E-221	2.02E-219	Ejaculatory bulb-specific protein 3; similar with PEB3_DROME
Px011301.2	1.5122	5.33E-93	1.38E-91	Putative uncharacterized protein; similar with D6WH87_TRICA
Px009152.1	1.4996	5.17E-21	4.54E-20	Innexin inx7; similar with INX7_DROME
Px003232.1	1.4984	1.17E-10	6.25E-10	1-phosphatidylinositol-4,5-bisphosphate phosphodiesterase gamma-1
Px000651.1	1.4970	3.22E-13	1.99E-12	Extracellular domains-containing protein CG31004
Px006536.1	1.4850	0.00E+00	0.00E+00	HMG176; similar with Q0MTA5_HELAM
Px013635.1	1.4584	1.68E-22	1.54E-21	Extracellular domains-containing protein CG31004
Px000072.1	1.4166	5.91E-14	3.80E-13	Protein CLEC16A; similar with CL16A_HUMAN
Px001082.1	1.3854	0.00E+00	0.00E+00	Unknown functio
Px002670.1	1.3679	0.00E+00	0.00E+00	Apolipophorins; similar with APLP_MANSE
Px012786.1	1.3299	0.00E+00	0.00E+00	GH11122; similar with B4JCY6_DROGR
Px013975.1	1.3267	2.86E-07	1.13E-06	Cuticlin-1; similar with E2BP69_9HYME
Px009301.1	1.3241	2.23E-45	3.46E-44	Beta-1,3-glucan-binding protein; similar with BGBP_BOMMO
Px002835.1	1.2932	1.92E-04	5.37E-04	Fasciclin-1; similar with FAS1_SCHAM
Px004419.1	1.2908	2.43E-07	9.72E-07	Putative uncharacterized protein GLEAN_08369
Px015492.1	1.2649	5.91E-22	5.35E-21	Protein halfway; similar with HFW1_DROPS
Px002608.1	1.2583	1.73E-12	1.03E-11	Protein kinase DC2; similar with KDC2_DROME
Px008031.1	1.1629	2.57E-04	7.03E-04	cAMP-dependent protein kinase catalytic subunit alpha
Px015730.1	1.1397	0.00E+00	0.00E+00	Apolipophorins; similar with APLP_MANSE
Px001430.1	1.1298	0.00E+00	0.00E+00	Chitin deacetylase 1; similar with B2ZGH5_9NEOP
Px001080.1	1.1262	0.00E+00	0.00E+00	Unknown functio
Px005322.1	1.1251	2.50E-46	3.97E-45	Cuticle protein 19; similar with CU19_LOCMI
Px015092.1	1.0810	2.56E-59	4.92E-58	Unknown functio
Px008625.1	1.0738	1.64E-58	3.13E-57	V-type proton ATPase 116 kDa subunit a isoform 1
Px014947.1	1.0681	1.22E-23	1.17E-22	Putative uncharacterized protein; similar with E2AAT5_9HYME
Px000220.2	1.0629	2.79E-05	8.80E-05	Bumetanide-sensitive sodium-(potassium)-chloride cotransporter
Px016770.1	1.0207	3.65E-12	2.13E-11	V-type proton ATPase subunit C; similar with VATC_MANSE
Px016246.1	-9.0336	3.73E-04	9.91E-04	Unknown functio
Px012920.1	-5.7371	1.31E-14	8.79E-14	Unknown functio
Px010970.1	-5.1269	1.33E-210	6.09E-209	Cuticle protein; similar with CU01_ANOGA
Px001530.1	-5.1032	8.45E-92	2.16E-90	Neurofilament heavy polypeptide; similar with NFH_MOUSE
Px001327.2	-4.9271	1.00E-22	9.26E-22	Unknown functio

Px014502.2	-4.9177	1.09E-29	1.26E-28	Putative uncharacterized protein; similar with B0W8F8_CULQU
Px012644.1	-4.8891	1.72E-84	4.13E-83	Filamentous hemagglutinin; similar with FHAB_BORPE
Px008648.1	-4.8302	1.88E-14	1.25E-13	Larval/pupal rigid cuticle protein 66; similar with CU66_HYACE
Px005321.1	-4.6028	2.84E-34	3.61E-33	Cuticle protein 19; similar with CU19_LOCMI
Px014251.1	-4.3113	1.63E-18	1.30E-17	Putative uncharacterized protein; similar with D6WG20_TRICA
Px005318.1	-4.2809	2.97E-43	4.42E-42	Cuticle protein 19; similar with CU19_LOCMI
Px016161.1	-4.1521	1.00E-98	2.73E-97	Neurofilament heavy polypeptide; similar with NFH_RAT
Px010665.1	-3.9457	7.76E-14	4.96E-13	Putative uncharacterized protein; similar with E3WXH7_ANODA
Px017104.1	-3.9233	1.10E-16	8.19E-16	Neurofilament heavy polypeptide; similar with NFH_MOUSE
Px014245.1	-3.5851	1.97E-19	1.63E-18	Unknown functio
Px012919.2	-3.4152	4.85E-05	1.49E-04	Cuticle protein 19; similar with CU19_LOCMI
Px005323.1	-3.2863	1.82E-23	1.73E-22	Cuticle protein 19; similar with CU19_LOCMI
Px008500.1	-3.0987	1.00E-124	3.20E-123	Pupal cuticle protein; similar with CUPP_BOMMO
Px003249.1	-2.7665	0.00E+00	0.00E+00	Larval cuticle protein LCP-22; similar with CU22_BOMMO
Px008654.1	-2.7571	5.40E-28	5.90E-27	Pupal cuticle protein Edg-84A; similar with CUP8_DROME
Px007290.1	-2.6760	7.93E-134	2.68E-132	Cuticle protein; similar with CU01_ANOGA
Px009321.1	-2.6343	1.76E-19	1.45E-18	Cuticle protein 3; similar with CU03_LONON
Px002841.1	-2.5840	3.11E-15	2.17E-14	Putative uncharacterized protein GLEAN_07427
Px003254.3	-2.4993	0.00E+00	0.00E+00	Larval cuticle protein LCP-17; similar with CU17_BOMMO
Px014252.1	-2.2750	5.84E-15	3.99E-14	Putative uncharacterized protein; similar with D6WG20_TRICA
Px003252.2	-2.2721	0.00E+00	0.00E+00	Larval cuticle protein LCP-30; similar with CU30_BOMMO
Px004340.1	-2.1994	2.01E-05	6.48E-05	Uncharacterized protein; similar with F1SF10_PIG
Px003681.1	-2.1521	5.92E-15	4.05E-14	Cuticle protein 19; similar with CU19_LOCMI
Px013556.1	-1.9665	0.00E+00	0.00E+00	Flexible cuticle protein 12; similar with CU12_HYACE
Px001064.1	-1.9506	2.87E-247	1.54E-245	Pupal cuticle protein Edg-84A; similar with CUP8_DROME
Px008653.1	-1.9082	3.21E-20	2.74E-19	Cuticle protein; similar with CU01_ANOGA
Px005288.1	-1.8733	2.28E-12	1.35E-11	Putative cuticle protein; similar with Q9U504_MANSE
Px003259.1	-1.8425	5.66E-111	1.68E-109	Endocuticle structural glycoprotein SgAbd-2
Px005317.2	-1.8082	1.04E-14	6.99E-14	Cuticle protein 7; similar with CU07_LOCMI
Px010337.1	-1.7509	4.56E-46	7.17E-45	Cuticular protein CPR77; similar with B2DBI2_9NEOP
Px002615.1	-1.7185	7.88E-24	7.62E-23	C-type lectin, galactose-binding (AGAP010196-PA)
Px005891.1	-1.7086	5.24E-10	2.66E-09	Putative cuticle protein; similar with Q9U509_MANSE
Px001065.1	-1.6858	5.33E-19	4.33E-18	Cuticle protein; similar with CU01_ANOGA
Px008954.1	-1.6672	1.06E-274	6.05E-273	Protein MICAL-2; similar with MICA2_HUMAN
Px009941.1	-1.6603	3.34E-04	8.97E-04	Putative uncharacterized protein; similar with E2A5A2_9HYME
Px017593.1	-1.6187	5.41E-41	7.74E-40	Putative uncharacterized protein; similar with D6X1E0_TRICA
Px000919.1	-1.5620	9.67E-48	1.58E-46	Enhancer of split m4 protein; similar with ESM4_DROME
Px014262.1	-1.5611	1.89E-59	3.65E-58	Putative cuticle protein; similar with C0H6H1_BOMMO
Px014536.1	-1.5191	8.05E-25	8.03E-24	Unknown functio
Px011630.1	-1.4823	5.20E-09	2.43E-08	Cuticle protein 7; similar with CU07_LOCMI
Px005320.1	-1.4616	1.11E-148	4.03E-147	Cuticle protein 7; similar with CU07_LOCMI
Px003255.1	-1.4547	0.00E+00	0.00E+00	Larval cuticle protein LCP-17; similar with CU17_BOMMO
Px003245.1	-1.3445	1.84E-227	9.18E-226	Larval cuticle protein LCP-22; similar with CU22_BOMMO
Px013555.1	-1.2894	4.85E-254	2.64E-252	Flexible cuticle protein 12; similar with CU12_HYACE
Px003729.1	-1.2580	4.23E-75	9.56E-74	Venom allergen 3; similar with VA3_SOLIN
Px014247.3	-1.2306	0.00E+00	0.00E+00	Cuticular protein CPR77; similar with B2DBI2_9NEOP
Px008652.2	-1.2135	3.96E-44	6.01E-43	Cuticle protein; similar with CU01_ANOGA
Px010900.1	-1.1915	0.00E+00	0.00E+00	Unknown functio
Px008680.1	-1.1712	2.29E-04	6.33E-04	Putative dsRNase; similar with B7VCC3_SPOLI
Px016485.1	-1.1684	5.49E-15	3.77E-14	Pupal cuticle protein; similar with CUPP_BOMMO
Px015352.3	-1.1051	3.91E-06	1.38E-05	Putative uncharacterized protein; similar with E3X258_ANODA
Px015226.1	-1.0508	9.46E-128	3.07E-126	GI23113; similar with B4KCJ7_DROMO
Px014261.1	-1.0402	6.99E-38	9.43E-37	Putative cuticle protein; similar with C0H6H2_BOMMO

Involved in pancreatic secretion

Px003126.1	9.7828	1.11E-04	3.21E-04	Adenylate cyclase type 3; similar with ADCY3_MOUSE
Px008411.1	8.8650	6.00E-05	1.81E-04	Adenylate cyclase type 3; similar with ADCY3_MOUSE
Px009476.1	4.3986	3.12E-06	1.11E-05	Group 3 secretory phospholipase A2; similar with PA2G3_BOVIN
Px008089.1	3.9186	0.00E+00	0.00E+00	Pancreatic lipase-related protein 2; similar with LIPR2_CAVPO
Px004847.1	3.8702	4.52E-11	2.46E-10	Putative carbonic anhydrase 5; similar with CAH5_CAEEL
Px005242.1	3.0767	6.37E-17	4.80E-16	Trypsin CFT-1; similar with TRYP_CHOFU
Px007621.1	3.0027	5.16E-07	2.00E-06	Trypsin CFT-1; similar with TRYP_CHOFU
Px007357.2	2.9638	0.00E+00	0.00E+00	Serine proteinase stubble; similar with STUB_DROME
Px017492.1	2.9247	3.48E-05	1.08E-04	Trypsin CFT-1; similar with TRYP_CHOFU
Px005241.1	2.8923	3.63E-27	3.87E-26	Trypsin CFT-1; similar with TRYP_CHOFU
Px007617.1	2.8498	1.67E-246	8.96E-245	Trypsin CFT-1; similar with TRYP_CHOFU
Px000992.1	2.7356	0.00E+00	0.00E+00	Carboxypeptidase A1; similar with CBPA1_BOVIN
Px014882.1	2.6696	8.90E-26	9.20E-25	Trypsin, alkaline C; similar with TRYC_MANSE
Px006441.1	2.6454	0.00E+00	0.00E+00	Lipase member H; similar with LIPH_RAT
Px008830.1	2.5145	3.44E-20	2.93E-19	Trypsin, alkaline C; similar with TRYC_MANSE
Px001804.1	2.4772	1.61E-09	7.81E-09	Trypsin, alkaline B; similar with TRYB_MANSE
Px011683.2	2.4698	9.12E-191	3.91E-189	Venom carboxylesterase-6; similar with EST6_APIME
Px005243.1	2.4179	0.00E+00	0.00E+00	Trypsin, alkaline A; similar with TRYA_MANSE
Px009569.1	2.3697	7.48E-240	3.88E-238	Trypsin CFT-1; similar with TRYP_CHOFU
Px000104.1	2.3695	6.34E-13	3.87E-12	Pancreatic lipase-related protein 2; similar with LIPR2_CAVPO
Px007616.1	2.3535	1.02E-74	2.30E-73	Trypsin CFT-1; similar with TRYP_CHOFU
Px007618.1	2.2331	1.62E-143	5.75E-142	Trypsin CFT-1; similar with TRYP_CHOFU
Px000402.2	2.2104	2.09E-62	4.14E-61	Serine protease persephone; similar with PSH_DROME
Px014592.1	2.1944	1.03E-24	1.02E-23	Trypsin CFT-1; similar with TRYP_CHOFU
Px002512.1	2.1941	1.00E-35	1.30E-34	Brachyurin; similar with COGS_UCAPU
Px006571.1	2.1935	0.00E+00	0.00E+00	Trypsin, alkaline B; similar with TRYB_MANSE
Px016057.1	2.1333	4.30E-39	5.93E-38	Trypsin, alkaline B; similar with TRYB_MANSE
Px011788.2	2.1319	2.40E-67	5.04E-66	Pancreatic lipase-related protein 2; similar with LIPR2_BOVIN
Px002309.1	2.1123	1.77E-05	5.73E-05	Putative uncharacterized protein; similar with E9IM57_SOLIN
Px005612.4	2.0853	4.92E-69	1.05E-67	Calcium-activated potassium channel slowpoke
Px001239.1	2.0005	4.17E-33	5.19E-32	Serine protease P162; similar with D2A5Y8_TRICA
Px010386.1	1.9598	3.91E-233	1.99E-231	Trypsin CFT-1; similar with TRYP_CHOFU
Px015275.1	1.9447	0.00E+00	0.00E+00	Trypsin CFT-1; similar with TRYP_CHOFU
Px005244.1	1.9247	6.69E-08	2.82E-07	Trypsin CFT-1; similar with TRYP_CHOFU
Px002492.1	1.9068	7.27E-05	2.17E-04	Carbonic anhydrase 3; similar with CAH3_BOVIN
Px004235.2	1.8977	7.45E-11	4.01E-10	Putative uncharacterized protein; similar with Q178N6_AEDAE
Px015277.1	1.8808	1.20E-25	1.23E-24	Trypsin CFT-1; similar with TRYP_CHOFU
Px013377.1	1.8477	2.46E-41	3.55E-40	Trypsin; similar with TRYP_PHACE
Px006878.1	1.7943	2.54E-04	6.97E-04	Protein kinase C, brain isozyme; similar with KPC1_DROME
Px007619.2	1.7921	0.00E+00	0.00E+00	Trypsin, alkaline A; similar with TRYA_MANSE
Px012048.1	1.7891	2.09E-29	2.38E-28	Chymotrypsin BI; similar with CTRB1_LITVA
Px006345.1	1.7781	1.52E-87	3.77E-86	Pancreatic lipase-related protein 2; similar with LIPR2_MYOCO
Px001831.1	1.7548	1.93E-08	8.52E-08	Headcase protein homolog; similar with HDC_HUMAN
Px004131.5	1.7548	8.64E-41	1.23E-39	Calcium-independent phospholipase A2-gamma
Px011886.1	1.7454	1.59E-62	3.15E-61	Trypsin CFT-1; similar with TRYP_CHOFU
Px000991.6	1.7324	1.37E-46	2.20E-45	Carboxypeptidase O; similar with CBPO_BOVIN
Px007674.2	1.7282	5.92E-240	3.08E-238	Collagenase; similar with COGS_HYPLI
Px005238.1	1.7135	3.84E-20	3.26E-19	Trypsin, alkaline B; similar with TRYB_MANSE
Px012568.1	1.7128	3.61E-103	1.02E-101	Trypsin CFT-1; similar with TRYP_CHOFU
Px004753.1	1.7057	1.64E-52	2.88E-51	Pancreatic lipase-related protein 2; similar with LIPR2_MYOCO
Px003649.1	1.6988	0.00E+00	0.00E+00	Esterase FE4; similar with ESTF_MYZPE
Px013162.1	1.6859	3.98E-103	1.12E-101	Serine protease snake; similar with SNAK_DROME
Px000995.2	1.6774	3.14E-184	1.31E-182	Carboxypeptidase A4; similar with CBPA4_HUMAN

Px016055.1	1.6104	9.12E-07	3.45E-06	Trypsin, alkaline C; similar with TRYC_MANSE
Px009638.1	1.5712	0.00E+00	0.00E+00	Trypsin, alkaline B; similar with TRYB_MANSE
Px011049.1	1.5512	8.68E-06	2.92E-05	Acetylcholinesterase; similar with ACES_FELCA
Px002866.1	1.5483	8.45E-05	2.49E-04	Trypsin, alkaline A; similar with TRYA_MANSE
Px001833.1	1.5360	1.24E-301	7.61E-300	Serine protease easter; similar with EAST_DROME
Px002441.1	1.5324	9.72E-35	1.25E-33	Trypsin, alkaline B; similar with TRYB_MANSE
Px000107.1	1.5230	1.38E-223	6.76E-222	Trypsin, alkaline C; similar with TRYC_MANSE
Px006572.1	1.5222	0.00E+00	0.00E+00	Trypsin; similar with TRYP_PHACE
Px005239.1	1.5039	3.47E-65	7.08E-64	Trypsin, alkaline A; similar with TRYA_MANSE
Px002861.1	1.5031	0.00E+00	0.00E+00	Trypsin, alkaline C; similar with TRYC_MANSE
Px011889.1	1.4989	1.23E-05	4.08E-05	Achelase-1; similar with ACH1_LONAC
Px000993.1	1.4934	0.00E+00	0.00E+00	Carboxypeptidase A2; similar with CBPA2_RAT
Px009789.1	1.4710	1.21E-05	3.99E-05	Adenylate cyclase type 2; similar with ADCY2_DROME
Px009940.2	1.4584	2.00E-08	8.84E-08	Acetylcholinesterase; similar with ACES_CULPI
Px003712.1	1.4525	1.46E-09	7.13E-09	Acetylcholinesterase; similar with ACES_CULPI
Px011499.2	1.4297	2.51E-12	1.48E-11	Serine protease persephone; similar with PSH_DROME
Px016056.1	1.4149	0.00E+00	0.00E+00	Trypsin, alkaline A; similar with TRYA_MANSE
Px004752.1	1.3734	0.00E+00	0.00E+00	Pancreatic lipase-related protein 2; similar with LIPR2_BOVIN
Px000515.1	1.3647	1.85E-27	1.99E-26	Esterase FE4; similar with ESTF_MYZPE
Px007900.1	1.3608	0.00E+00	0.00E+00	Chymotrypsin BI; similar with CTRB1_LITVA
Px002296.1	1.3429	6.45E-75	1.45E-73	Pancreatic triacylglycerol lipase; similar with LIPP_HUMAN
Px002864.1	1.3149	1.41E-13	8.90E-13	Trypsin, alkaline B; similar with TRYB_MANSE
Px011443.1	1.2632	1.85E-11	1.03E-10	Esterase FE4; similar with ESTF_MYZPE
Px005193.2	1.2375	6.57E-27	6.96E-26	Pancreatic lipase-related protein 2; similar with LIPR2_MYOCO
Px009566.1	1.2291	1.92E-62	3.79E-61	Trypsin, alkaline C; similar with TRYC_MANSE
Px012939.1	1.2048	1.82E-08	8.07E-08	Protein turtle; similar with TUTL_DROME
Px015791.1	1.1914	1.59E-08	7.06E-08	Sodium/potassium-transporting ATPase subunit beta-1
Px005342.1	1.1769	1.93E-52	3.38E-51	Collagenase; similar with COGS_HYPLI
Px004750.1	1.1637	0.00E+00	0.00E+00	Pancreatic lipase-related protein 2; similar with LIPR2_RAT
Px009639.1	1.1592	0.00E+00	0.00E+00	Trypsin CFT-1; similar with TRYP_CHOFU
Px017996.1	1.1300	3.04E-23	2.87E-22	Acetylcholinesterase; similar with ACES_HUMAN
Px003670.2	1.1149	0.00E+00	0.00E+00	Serine protease easter; similar with EAST_DROME
Px006570.1	1.1073	0.00E+00	0.00E+00	Trypsin, alkaline B; similar with TRYB_MANSE
Px007601.1	1.1017	1.31E-16	9.72E-16	Chymotrypsin-1; similar with CTR1_ANOGA
Px003970.1	1.0952	2.72E-182	1.13E-180	Plasma membrane calcium-transporting ATPase 3
Px016307.3	1.0876	1.22E-104	3.47E-103	Sodium/potassium-transporting ATPase subunit alpha
Px000220.2	1.0629	2.79E-05	8.80E-05	Bumetanide-sensitive sodium-(potassium)-chloride cotransporter
Px002295.1	1.0591	2.25E-92	5.79E-91	Pancreatic lipase-related protein 2; similar with LIPR2_BOVIN
Px010855.1	1.0588	6.22E-09	2.89E-08	Trypsin, alkaline A; similar with TRYA_MANSE
Px017515.1	1.0202	4.47E-23	4.19E-22	Trypsin, alkaline C; similar with TRYC_MANSE
Px000994.1	1.0157	0.00E+00	0.00E+00	Zinc carboxypeptidase A 1; similar with CBPA1_DROPS
Px000996.2	1.0153	2.16E-05	6.93E-05	Carboxypeptidase A2; similar with CBPA2_MOUSE
Px005936.2	1.0100	2.58E-14	1.70E-13	Trypsin, alkaline C; similar with TRYC_MANSE
Px011887.1	1.0083	4.45E-16	3.21E-15	Trypsin, alkaline C; similar with TRYC_MANSE
Px000395.1	1.0057	3.37E-180	1.39E-178	Alpha-amylase 4N; similar with AM4N_DROAN
Px000191.1	-8.8435	3.73E-04	9.91E-04	Trypsin-4; similar with TRY4_ANOGA
Px003251.1	-5.6346	2.17E-287	1.29E-285	Putative cuticle protein; similar with C0H6E8_BOMMO
Px008989.1	-4.8302	9.70E-08	4.03E-07	Trypsin CFT-1; similar with TRYP_CHOFU
Px010907.1	-3.3130	1.22E-46	1.96E-45	Putative uncharacterized protein; similar with D6WJ01_TRICA
Px001990.2	-2.4252	2.16E-214	9.99E-213	Carboxypeptidase B; similar with CBPB_ASTFL
Px014636.1	-2.1712	8.11E-09	3.72E-08	Trypsin-1; similar with TRY1_ANOGA
Px016923.1	-1.8978	1.61E-208	7.33E-207	Carbonic anhydrase 2; similar with CAH2_HUMAN
Px000102.1	-1.8916	1.29E-32	1.57E-31	Pancreatic lipase-related protein 3; similar with LIPR3_HUMAN
Px005373.1	-1.8302	1.62E-09	7.87E-09	Carboxypeptidase B; similar with CBPB_ASTFL

Px002588.1	-1.7813	6.19E-16	4.43E-15	Trypsin, alkaline C; similar with TRYC_MANSE
Px015279.1	-1.5306	1.85E-04	5.19E-04	Trypsin, alkaline A; similar with TRYA_MANSE
Px012677.1	-1.2183	2.47E-04	6.79E-04	Lipase member H-A; similar with LIPHA_XENLA
Px004846.1	-1.1784	2.93E-85	7.09E-84	Putative carbonic anhydrase 5; similar with CAH5_CAEEL
Px002440.1	-1.1249	5.69E-06	1.96E-05	Trypsin, alkaline B; similar with TRYB_MANSE

Involved in leishmaniasis

Px004914.1	10.5170	2.04E-04	5.68E-04	Short neuropeptide F; similar with SNPF_ANOGA
Px005645.3	3.3986	8.90E-10	4.43E-09	Integrin alpha-PS3; similar with ITA3_DROME
Px002004.1	2.4537	5.97E-61	1.17E-59	Mitogen-activated protein kinase kinase kinase 7
Px006837.1	2.4520	3.28E-29	3.72E-28	Serine/threonine-protein kinase pelle; similar with KPEL_DROME
Px014553.1	2.1507	7.24E-12	4.15E-11	Mitogen-activated protein kinase ERK-A
Px003725.1	2.1160	5.10E-66	1.05E-64	Interleukin-1 receptor-associated kinase 4
Px010499.1	1.9100	1.29E-36	1.70E-35	Serine/threonine-protein kinase pelle
Px000138.1	1.8328	1.68E-04	4.76E-04	Mitogen-activated protein kinase ERK-A
Px006878.1	1.7943	2.54E-04	6.97E-04	Protein kinase C, brain isozyme; similar with KPC1_DROME
Px012604.1	1.6352	2.94E-07	1.16E-06	Integrin beta; similar with E9GI17_DAPPU
Px004977.1	1.5523	5.30E-143	1.87E-141	Transcription factor AP-1; similar with JRA_DROME
Px015514.1	1.4407	7.97E-06	2.69E-05	Serine/threonine-protein kinase pelle; similar with KPEL_DROME
Px015291.1	1.1344	1.11E-12	6.70E-12	Mitogen-activated protein kinase 14B
Px011555.1	1.1315	1.20E-63	2.40E-62	Integrin beta-PS; similar with ITBX_DROME
Px002012.1	1.1305	5.59E-10	2.83E-09	Integrin beta-nu; similar with ITBN_DROME
Px003409.1	1.1173	2.96E-04	8.01E-04	Protein toll; similar with TOLL_DROME
Px013907.1	1.0469	8.58E-06	2.89E-05	Tyrosine-protein kinase hopscotch; similar with JAK_DROME
Px001789.1	-1.4859	7.13E-28	7.75E-27	Protein toll; similar with TOLL_DROME

Involved in Influenza A

Px004914.1	10.5170	2.04E-04	5.68E-04	Short neuropeptide F; similar with SNPF_ANOGA
Px008374.1	10.1228	1.11E-04	3.21E-04	Phosphatidylinositol-4,5-bisphosphate 3-kinase catalytic subunit delta isoform
Px001483.1	5.1123	2.71E-10	1.40E-09	Leucine-rich repeat and WD repeat-containing protein KIAA1239
Px005242.1	3.0767	6.37E-17	4.80E-16	Trypsin CFT-1; similar with TRYP_CHOFU
Px007621.1	3.0027	5.16E-07	2.00E-06	Trypsin CFT-1; similar with TRYP_CHOFU
Px007357.2	2.9638	0.00E+00	0.00E+00	Serine proteinase stubble; similar with STUB_DROME
Px004099.2	2.9544	2.23E-23	2.11E-22	Chaoptin; similar with CHAO_DROME
Px017492.1	2.9247	3.48E-05	1.08E-04	Trypsin CFT-1; similar with TRYP_CHOFU
Px005241.1	2.8923	3.63E-27	3.87E-26	Trypsin CFT-1; similar with TRYP_CHOFU
Px007617.1	2.8498	1.67E-246	8.96E-245	Trypsin CFT-1; similar with TRYP_CHOFU
Px014882.1	2.6696	8.90E-26	9.20E-25	Trypsin, alkaline C; similar with TRYC_MANSE
Px008830.1	2.5145	3.44E-20	2.93E-19	Trypsin, alkaline C; similar with TRYC_MANSE
Px001804.1	2.4772	1.61E-09	7.81E-09	Trypsin, alkaline B; similar with TRYB_MANSE
Px006837.1	2.4520	3.28E-29	3.72E-28	Serine/threonine-protein kinase pelle; similar with KPEL_DROME
Px005243.1	2.4179	0.00E+00	0.00E+00	Trypsin, alkaline A; similar with TRYA_MANSE
Px008372.1	2.4177	3.42E-08	1.48E-07	Phosphatidylinositol-4,5-bisphosphate 3-kinase catalytic subunit beta isoform
Px009569.1	2.3697	7.48E-240	3.88E-238	Trypsin CFT-1; similar with TRYP_CHOFU
Px007616.1	2.3535	1.02E-74	2.30E-73	Trypsin CFT-1; similar with TRYP_CHOFU
Px007618.1	2.2331	1.62E-143	5.75E-142	Trypsin CFT-1; similar with TRYP_CHOFU
Px010505.1	2.2215	1.05E-44	1.61E-43	Programmed cell death protein 4; similar with PDCD4_CHICK
Px013101.1	2.2142	7.92E-05	2.35E-04	Nose resistant to fluoxetine protein 6; similar with NRF6_CAEEL
Px000402.2	2.2104	2.09E-62	4.14E-61	Serine protease persephone; similar with PSH_DROME
Px011248.1	2.1953	2.38E-05	7.59E-05	Radial spoke head protein 4 homolog A
Px014592.1	2.1944	1.03E-24	1.02E-23	Trypsin CFT-1; similar with TRYP_CHOFU
Px006571.1	2.1935	0.00E+00	0.00E+00	Trypsin, alkaline B; similar with TRYB_MANSE
Px014553.1	2.1507	7.24E-12	4.15E-11	Mitogen-activated protein kinase ERK-A
Px002457.1	2.1483	7.92E-64	1.59E-62	Protein dpy-19 homolog 1; similar with D19L1_HUMAN

Px016057.1	2.1333	4.30E-39	5.93E-38	Trypsin, alkaline B; similar with TRYB_MANSE
Px003725.1	2.1160	5.10E-66	1.05E-64	Interleukin-1 receptor-associated kinase 4
Px009870.1	2.1030	0.00E+00	0.00E+00	Inter-alpha-trypsin inhibitor heavy chain H5-like protein
Px013633.1	2.0616	3.46E-39	4.80E-38	tRNA 2-thiocytidine biosynthesis protein TtcA
Px003186.1	2.0295	2.84E-71	6.21E-70	RAC serine/threonine-protein kinase; similar with AKT1_DROME
Px006234.1	2.0012	1.49E-24	1.47E-23	Chaoptin; similar with CHAO_DROME
Px001239.1	2.0005	4.17E-33	5.19E-32	Serine protease P162; similar with D2A5Y8_TRICA
Px010386.1	1.9598	3.91E-233	1.99E-231	Trypsin CFT-1; similar with TRYP_CHOFU
Px015275.1	1.9447	0.00E+00	0.00E+00	Trypsin CFT-1; similar with TRYP_CHOFU
Px005244.1	1.9247	6.69E-08	2.82E-07	Trypsin CFT-1; similar with TRYP_CHOFU
Px010499.1	1.9100	1.29E-36	1.70E-35	Serine/threonine-protein kinase pelle; similar with KPEL_DROME
Px015277.1	1.8808	1.20E-25	1.23E-24	Trypsin CFT-1; similar with TRYP_CHOFU
Px013377.1	1.8477	2.46E-41	3.55E-40	Trypsin; similar with TRYP_PHACE
Px000138.1	1.8328	1.68E-04	4.76E-04	Mitogen-activated protein kinase ERK-A
Px006878.1	1.7943	2.54E-04	6.97E-04	Protein kinase C, brain isozyme; similar with KPC1_DROME
Px007619.2	1.7921	0.00E+00	0.00E+00	Trypsin, alkaline A; similar with TRYA_MANSE
Px011886.1	1.7454	1.59E-62	3.15E-61	Trypsin CFT-1; similar with TRYP_CHOFU
Px005238.1	1.7135	3.84E-20	3.26E-19	Trypsin, alkaline B; similar with TRYB_MANSE
Px012568.1	1.7128	3.61E-103	1.02E-101	Trypsin CFT-1; similar with TRYP_CHOFU
Px013162.1	1.6859	3.98E-103	1.12E-101	Serine protease snake; similar with SNAK_DROME
Px016055.1	1.6104	9.12E-07	3.45E-06	Trypsin, alkaline C; similar with TRYC_MANSE
Px009638.1	1.5712	0.00E+00	0.00E+00	Trypsin, alkaline B; similar with TRYB_MANSE
Px004977.1	1.5523	5.30E-143	1.87E-141	Transcription factor AP-1; similar with JRA_DROME
Px002866.1	1.5483	8.45E-05	2.49E-04	Trypsin, alkaline A; similar with TRYA_MANSE
Px001833.1	1.5360	1.24E-301	7.61E-300	Serine protease easter; similar with EAST_DROME
Px009928.1	1.5324	1.81E-04	5.08E-04	Farnesyl pyrophosphate synthase 2; similar with FPPS2_ARATH
Px002441.1	1.5324	9.72E-35	1.25E-33	Trypsin, alkaline B; similar with TRYB_MANSE
Px008604.1	1.5243	7.49E-30	8.64E-29	AGAP011476-PA; similar with Q7Q343_ANOGA
Px000107.1	1.5230	1.38E-223	6.76E-222	Trypsin, alkaline C; similar with TRYC_MANSE
Px006572.1	1.5222	0.00E+00	0.00E+00	Trypsin; similar with TRYP_PHACE
Px005239.1	1.5039	3.47E-65	7.08E-64	Trypsin, alkaline A; similar with TRYA_MANSE
Px002861.1	1.5031	0.00E+00	0.00E+00	Trypsin, alkaline C; similar with TRYC_MANSE
Px011889.1	1.4989	1.23E-05	4.08E-05	Achelase-1; similar with ACH1_LONAC
Px015514.1	1.4407	7.97E-06	2.69E-05	Serine/threonine-protein kinase pelle; similar with KPEL_DROME
Px011499.2	1.4297	2.51E-12	1.48E-11	Serine protease persephone; similar with PSH_DROME
Px016056.1	1.4149	0.00E+00	0.00E+00	Trypsin, alkaline A; similar with TRYA_MANSE
Px009586.1	1.4046	1.13E-06	4.22E-06	Phosphatidylinositol 3-kinase regulatory subunit gamma
Px017224.1	1.3397	6.85E-10	3.44E-09	Chaoptin; similar with CHAO_DROME
Px013698.3	1.3397	1.45E-04	4.14E-04	Dedicator of cytokinesis protein 6; similar with DOCK6_HUMAN
Px007552.1	1.3210	8.40E-38	1.13E-36	Paramyosin, putative; similar with E0VGP7_PEDHC
Px002864.1	1.3149	1.41E-13	8.90E-13	Trypsin, alkaline B; similar with TRYB_MANSE
Px010747.1	1.2582	3.63E-14	2.36E-13	Protein phosphatase 1 regulatory subunit 7
Px009566.1	1.2291	1.92E-62	3.79E-61	Trypsin, alkaline C; similar with TRYC_MANSE
Px005490.1	1.2205	5.89E-39	8.08E-38	Inhibitor of nuclear factor kappa-B kinase subunit beta
Px013205.1	1.2182	3.01E-14	1.97E-13	Putative uncharacterized protein; similar with D6WHY1_TRICA
Px005342.1	1.1769	1.93E-52	3.38E-51	Collagenase; similar with COGS_HYPLI
Px009639.1	1.1592	0.00E+00	0.00E+00	Trypsin CFT-1; similar with TRYP_CHOFU
Px015291.1	1.1344	1.11E-12	6.70E-12	Mitogen-activated protein kinase 14B
Px003409.1	1.1173	2.96E-04	8.01E-04	Protein toll; similar with TOLL_DROME
Px003670.2	1.1149	0.00E+00	0.00E+00	Serine protease easter; similar with EAST_DROME
Px006570.1	1.1073	0.00E+00	0.00E+00	Trypsin, alkaline B; similar with TRYB_MANSE
Px007601.1	1.1017	1.31E-16	9.72E-16	Chymotrypsin-1; similar with CTR1_ANOGA
Px007918.1	1.1016	4.70E-06	1.64E-05	Three prime repair exonuclease 2; similar with TREX2_MOUSE
Px011869.1	1.0924	5.18E-16	3.73E-15	Arf-GAP domain and FG repeats-containing protein 1

Px014947.1	1.0681	1.22E-23	1.17E-22	Putative uncharacterized protein; similar with E2AAT5_9HYME
Px010855.1	1.0588	6.22E-09	2.89E-08	Trypsin, alkaline A; similar with TRYA_MANSE
Px007700.1	1.0471	1.22E-07	5.02E-07	Nuclear RNA export factor 1; similar with NXF1_DROME
Px013907.1	1.0469	8.58E-06	2.89E-05	Tyrosine-protein kinase hopscotch; similar with JAK_DROME
Px005422.1	1.0342	2.89E-73	6.46E-72	Exportin-1; similar with XPO1_MOUSE
Px006146.1	1.0275	2.16E-16	1.58E-15	Eukaryotic translation initiation factor 2-alpha kinase 3
Px011958.1	1.0257	7.39E-12	4.23E-11	Transmembrane protein 184B; similar with T184B_HUMAN
Px007415.2	1.0237	1.29E-06	4.81E-06	Furin-like protease 1, isoforms 1/1-X/2
Px017515.1	1.0202	4.47E-23	4.19E-22	Trypsin, alkaline C; similar with TRYC_MANSE
Px005936.2	1.0100	2.58E-14	1.70E-13	Trypsin, alkaline C; similar with TRYC_MANSE
Px011887.1	1.0083	4.45E-16	3.21E-15	Trypsin, alkaline C; similar with TRYC_MANSE
Px016832.3	1.0068	6.61E-06	2.26E-05	Stress-activated protein kinase JNK; similar with JNK_DROME
Px000191.1	-8.8435	3.73E-04	9.91E-04	Trypsin-4; similar with TRY4_ANOGA
Px008989.1	-4.8302	9.70E-08	4.03E-07	Trypsin CFT-1; similar with TRYP_CHOFU
Px014636.1	-2.1712	8.11E-09	3.72E-08	Trypsin-1; similar with TRY1_ANOGA
Px002588.1	-1.7813	6.19E-16	4.43E-15	Trypsin, alkaline C; similar with TRYC_MANSE
Px009322.1	-1.7596	1.89E-98	5.11E-97	Unknown functio
Px015279.1	-1.5306	1.85E-04	5.19E-04	Trypsin, alkaline A; similar with TRYA_MANSE
Px006532.1	-1.3578	1.19E-126	3.83E-125	Eukaryotic translation initiation factor 2 subunit 1
Px001559.1	-1.3250	2.05E-07	8.24E-07	Unknown functio
Px002440.1	-1.1249	5.69E-06	1.96E-05	Trypsin, alkaline B; similar with TRYB_MANSE
Px012579.1	-1.1226	3.09E-34	3.92E-33	mRNA export factor; similar with RAEL1_PIG
**Involved in protein digestion and absorption**				
Px005242.1	3.0767	6.37E-17	4.80E-16	Trypsin CFT-1; similar with TRYP_CHOFU
Px007621.1	3.0027	5.16E-07	2.00E-06	Trypsin CFT-1; similar with TRYP_CHOFU
Px007357.2	2.9638	0.00E+00	0.00E+00	Serine proteinase stubble; similar with STUB_DROME
Px017492.1	2.9247	3.48E-05	1.08E-04	Trypsin CFT-1; similar with TRYP_CHOFU
Px005241.1	2.8923	3.63E-27	3.87E-26	Trypsin CFT-1; similar with TRYP_CHOFU
Px007617.1	2.8498	1.67E-246	8.96E-245	Trypsin CFT-1; similar with TRYP_CHOFU
Px000992.1	2.7356	0.00E+00	0.00E+00	Carboxypeptidase A1; similar with CBPA1_BOVIN
Px014882.1	2.6696	8.90E-26	9.20E-25	Trypsin, alkaline C; similar with TRYC_MANSE
Px008830.1	2.5145	3.44E-20	2.93E-19	Trypsin, alkaline C; similar with TRYC_MANSE
Px001804.1	2.4772	1.61E-09	7.81E-09	Trypsin, alkaline B; similar with TRYB_MANSE
Px005243.1	2.4179	0.00E+00	0.00E+00	Trypsin, alkaline A; similar with TRYA_MANSE
Px009569.1	2.3697	7.48E-240	3.88E-238	Trypsin CFT-1; similar with TRYP_CHOFU
Px007616.1	2.3535	1.02E-74	2.30E-73	Trypsin CFT-1; similar with TRYP_CHOFU
Px007618.1	2.2331	1.62E-143	5.75E-142	Trypsin CFT-1; similar with TRYP_CHOFU
Px000402.2	2.2104	2.09E-62	4.14E-61	Serine protease persephone; similar with PSH_DROME
Px014592.1	2.1944	1.03E-24	1.02E-23	Trypsin CFT-1; similar with TRYP_CHOFU
Px002512.1	2.1941	1.00E-35	1.30E-34	Brachyurin; similar with COGS_UCAPU
Px006571.1	2.1935	0.00E+00	0.00E+00	Trypsin, alkaline B; similar with TRYB_MANSE
Px010627.1	2.1698	0.00E+00	0.00E+00	Wing disc-specific protein; similar with Q8WPH2_BOMMO
Px005328.1	2.1424	1.21E-73	2.72E-72	Tubulin-specific chaperone cofactor E-like protein
Px016057.1	2.1333	4.30E-39	5.93E-38	Trypsin, alkaline B; similar with TRYB_MANSE
Px001239.1	2.0005	4.17E-33	5.19E-32	Serine protease P162; similar with D2A5Y8_TRICA
Px010386.1	1.9598	3.91E-233	1.99E-231	Trypsin CFT-1; similar with TRYP_CHOFU
Px015275.1	1.9447	0.00E+00	0.00E+00	Trypsin CFT-1; similar with TRYP_CHOFU
Px005244.1	1.9247	6.69E-08	2.82E-07	Trypsin CFT-1; similar with TRYP_CHOFU
Px015277.1	1.8808	1.20E-25	1.23E-24	Trypsin CFT-1; similar with TRYP_CHOFU
Px013377.1	1.8477	2.46E-41	3.55E-40	Trypsin; similar with TRYP_PHACE
Px007619.2	1.7921	0.00E+00	0.00E+00	Trypsin, alkaline A; similar with TRYA_MANSE
Px012048.1	1.7891	2.09E-29	2.38E-28	Chymotrypsin BI; similar with CTRB1_LITVA
Px011886.1	1.7454	1.59E-62	3.15E-61	Trypsin CFT-1; similar with TRYP_CHOFU

Px000991.6	1.7324	1.37E-46	2.20E-45	Carboxypeptidase O; similar with CBPO_BOVIN
Px007674.2	1.7282	5.92E-240	3.08E-238	Collagenase; similar with COGS_HYPLI
Px005238.1	1.7135	3.84E-20	3.26E-19	Trypsin, alkaline B; similar with TRYB_MANSE
Px012568.1	1.7128	3.61E-103	1.02E-101	Trypsin CFT-1; similar with TRYP_CHOFU
Px013162.1	1.6859	3.98E-103	1.12E-101	Serine protease snake; similar with SNAK_DROME
Px000995.2	1.6774	3.14E-184	1.31E-182	Carboxypeptidase A4; similar with CBPA4_HUMAN
Px016055.1	1.6104	9.12E-07	3.45E-06	Trypsin, alkaline C; similar with TRYC_MANSE
Px009638.1	1.5712	0.00E+00	0.00E+00	Trypsin, alkaline B; similar with TRYB_MANSE
Px002866.1	1.5483	8.45E-05	2.49E-04	Trypsin, alkaline A; similar with TRYA_MANSE
Px001833.1	1.5360	1.24E-301	7.61E-300	Serine protease easter; similar with EAST_DROME
Px002441.1	1.5324	9.72E-35	1.25E-33	Trypsin, alkaline B; similar with TRYB_MANSE
Px000107.1	1.5230	1.38E-223	6.76E-222	Trypsin, alkaline C; similar with TRYC_MANSE
Px006572.1	1.5222	0.00E+00	0.00E+00	Trypsin; similar with TRYP_PHACE
Px004947.1	1.5156	2.31E-09	1.11E-08	Proton-coupled amino acid transporter 4
Px005239.1	1.5039	3.47E-65	7.08E-64	Trypsin, alkaline A; similar with TRYA_MANSE
Px002861.1	1.5031	0.00E+00	0.00E+00	Trypsin, alkaline C; similar with TRYC_MANSE
Px011889.1	1.4989	1.23E-05	4.08E-05	Achelase-1; similar with ACH1_LONAC
Px000993.1	1.4934	0.00E+00	0.00E+00	Carboxypeptidase A2; similar with CBPA2_RAT
Px011499.2	1.4297	2.51E-12	1.48E-11	Serine protease persephone; similar with PSH_DROME
Px016056.1	1.4149	0.00E+00	0.00E+00	Trypsin, alkaline A; similar with TRYA_MANSE
Px007900.1	1.3608	0.00E+00	0.00E+00	Chymotrypsin BI; similar with CTRB1_LITVA
Px002864.1	1.3149	1.41E-13	8.90E-13	Trypsin, alkaline B; similar with TRYB_MANSE
Px005604.1	1.3142	3.78E-19	3.08E-18	Monocarboxylate transporter 10; similar with MOT10_MOUSE
Px011737.1	1.3022	0.00E+00	0.00E+00	Neurofilament heavy polypeptide; similar with NFH_MOUSE
Px002792.1	1.2920	7.53E-26	7.80E-25	Unknown functio
Px002540.1	1.2876	1.80E-19	1.49E-18	Proton-coupled amino acid transporter 4
Px008960.1	1.2616	6.61E-37	8.79E-36	Peptide transporter family 1; similar with PEPT1_DROME
Px013646.1	1.2335	7.74E-16	5.53E-15	Proton-coupled amino acid transporter 4
Px009566.1	1.2291	1.92E-62	3.79E-61	Trypsin, alkaline C; similar with TRYC_MANSE
Px008882.1	1.2281	6.01E-123	1.88E-121	Unknown functio
Px012067.1	1.2111	7.18E-07	2.75E-06	Proton-coupled amino acid transporter 4
Px015791.1	1.1914	1.59E-08	7.06E-08	Sodium/potassium-transporting ATPase subunit beta-1
Px004330.1	1.1861	4.87E-44	7.38E-43	Proton-coupled amino acid transporter 1
Px005342.1	1.1769	1.93E-52	3.38E-51	Collagenase; similar with COGS_HYPLI
Px009639.1	1.1592	0.00E+00	0.00E+00	Trypsin CFT-1; similar with TRYP_CHOFU
Px005739.1	1.1553	1.10E-15	7.78E-15	Putative uncharacterized protein; similar with B0W6E4_CULQU
Px003670.2	1.1149	0.00E+00	0.00E+00	Serine protease easter; similar with EAST_DROME
Px008393.1	1.1138	3.51E-06	1.24E-05	Nck-associated protein 5; similar with NCKP5_HUMAN
Px006570.1	1.1073	0.00E+00	0.00E+00	Trypsin, alkaline B; similar with TRYB_MANSE
Px007601.1	1.1017	1.31E-16	9.72E-16	Chymotrypsin-1; similar with CTR1_ANOGA
Px016307.3	1.0876	1.22E-104	3.47E-103	Sodium/potassium-transporting ATPase subunit alpha
Px002572.2	1.0850	2.97E-25	3.01E-24	Large proline-rich protein bag6-A; similar with BAG6A_XENLA
Px010855.1	1.0588	6.22E-09	2.89E-08	Trypsin, alkaline A; similar with TRYA_MANSE
Px017515.1	1.0202	4.47E-23	4.19E-22	Trypsin, alkaline C; similar with TRYC_MANSE
Px000994.1	1.0157	0.00E+00	0.00E+00	Zinc carboxypeptidase A 1; similar with CBPA1_DROPS
Px000996.2	1.0153	2.16E-05	6.93E-05	Carboxypeptidase A2; similar with CBPA2_MOUSE
Px005936.2	1.0100	2.58E-14	1.70E-13	Trypsin, alkaline C; similar with TRYC_MANSE
Px011887.1	1.0083	4.45E-16	3.21E-15	Trypsin, alkaline C; similar with TRYC_MANSE
Px000191.1	-8.8435	3.73E-04	9.91E-04	Trypsin-4; similar with TRY4_ANOGA
Px008989.1	-4.8302	9.70E-08	4.03E-07	Trypsin CFT-1; similar with TRYP_CHOFU
Px001674.1	-3.5040	1.12E-30	1.32E-29	Cuticular protein CPG7; similar with B2DBG1_9NEOP
Px000267.1	-3.2452	8.15E-08	3.41E-07	Zinc metalloproteinase nas-15; similar with NAS15_CAEEL
Px001990.2	-2.4252	2.16E-214	9.99E-213	Carboxypeptidase B; similar with CBPB_ASTFL
Px014636.1	-2.1712	8.11E-09	3.72E-08	Trypsin-1; similar with TRY1_ANOGA

Px005373.1	-1.8302	1.62E-09	7.87E-09	Carboxypeptidase B; similar with CBPB_ASTFL
Px002588.1	-1.7813	6.19E-16	4.43E-15	Trypsin, alkaline C; similar with TRYC_MANSE
Px015279.1	-1.5306	1.85E-04	5.19E-04	Trypsin, alkaline A; similar with TRYA_MANSE
Px012323.1	-1.3989	2.47E-15	1.73E-14	Lysine histidine transporter 1; similar with LHT1_ARATH
Px012129.1	-1.2754	4.68E-19	3.82E-18	Lysosomal Pro-X carboxypeptidase; similar with PCP_PONAB
Px008408.1	-1.2190	8.68E-14	5.53E-13	Ras-related protein RabJ; similar with E2C0I8_9HYME
Px003112.1	-1.1686	3.03E-04	8.21E-04	Collagen alpha-1(XV) chain; similar with COFA1_MOUSE
Px001226.2	-1.1578	4.15E-18	3.25E-17	Collagen alpha-2(I) chain; similar with CO1A2_RAT
Px002440.1	-1.1249	5.69E-06	1.96E-05	Trypsin, alkaline B; similar with TRYB_MANSE
Px007681.2	-1.0735	2.17E-05	6.95E-05	Monocarboxylate transporter 2; similar with MOT2_RAT
Px004618.2	-1.0610	8.78E-52	1.51E-50	Vesicular inhibitory amino acid transporter

Involved in apoptosis

Px008374.1	10.1228	1.11E-04	3.21E-04	Phosphatidylinositol-4,5-bisphosphate 3-kinase catalytic subunit delta isoform
Px012662.1	2.9772	2.07E-05	6.66E-05	cAMP-dependent protein kinase type II regulatory subunit
Px002283.1	2.4843	2.38E-57	4.44E-56	Apoptosis-inducing factor 3; similar with AIFM3_HUMAN
Px006837.1	2.4520	3.28E-29	3.72E-28	Serine/threonine-protein kinase pelle; similar with KPEL_DROME
Px008372.1	2.4177	3.42E-08	1.48E-07	Phosphatidylinositol-4,5-bisphosphate 3-kinase catalytic subunit beta isoform
Px009987.1	2.1996	6.07E-12	3.49E-11	Caspase-1; similar with CASP1_DROME
Px003725.1	2.1160	5.10E-66	1.05E-64	Interleukin-1 receptor-associated kinase 4
Px003186.1	2.0295	2.84E-71	6.21E-70	RAC serine/threonine-protein kinase; similar with AKT1_DROME
Px010499.1	1.9100	1.29E-36	1.70E-35	Serine/threonine-protein kinase pelle; similar with KPEL_DROME
Px009688.1	1.7268	2.61E-05	8.28E-05	Putative apoptosis-inducing factor 1, mitochondrial
Px012893.1	1.5996	1.14E-20	9.85E-20	Putative apoptosis-inducing factor 1, mitochondrial
Px015514.1	1.4407	7.97E-06	2.69E-05	Serine/threonine-protein kinase pelle; similar with KPEL_DROME
Px009351.1	1.4222	1.66E-04	4.71E-04	DNA fragmentation factor subunit beta; similar with DFFB_RAT
Px009586.1	1.4046	1.13E-06	4.22E-06	Phosphatidylinositol 3-kinase regulatory subunit gamma
Px016244.2	1.3330	9.62E-36	1.26E-34	Calcium and integrin-binding family member 2
Px013159.1	1.2799	2.29E-07	9.21E-07	Calcium-binding protein p22; similar with CHP1_RAT
Px002608.1	1.2583	1.73E-12	1.03E-11	Protein kinase DC2; similar with KDC2_DROME
Px005490.1	1.2205	5.89E-39	8.08E-38	Inhibitor of nuclear factor kappa-B kinase subunit beta
Px008798.1	1.1684	3.42E-59	6.55E-58	Serine/threonine-protein phosphatase 2B catalytic subunit 3
Px008031.1	1.1629	2.57E-04	7.03E-04	cAMP-dependent protein kinase catalytic subunit alpha
Px002728.1	1.0196	6.50E-19	5.27E-18	Calcium and integrin-binding family member 2
Px004548.2	-1.0947	1.89E-20	1.63E-19	Putative apoptosis-inducing factor 1, mitochondrial

Involved in glycerophospholipid metabolism

Px009476.1	4.3986	3.12E-06	1.11E-05	Group 3 secretory phospholipase A2; similar with PA2G3_BOVIN
Px014969.1	4.0027	9.70E-05	2.83E-04	Glycerol-3-phosphate acyltransferase 3
Px012694.3	3.5448	5.62E-16	4.03E-15	Lipase 1; similar with LIP1_DROME
Px016285.1	3.3986	2.09E-05	6.73E-05	Tafazzin homolog; similar with TAZ_DROME
Px011234.1	3.0096	2.31E-17	1.77E-16	Nose resistant to fluoxetine protein 6; similar with NRF6_CAEEL
Px003942.1	2.6934	1.55E-07	6.31E-07	Choline/ethanolaminephosphotransferase 1
Px002585.2	2.6652	1.09E-28	1.22E-27	Nose resistant to fluoxetine protein 6; similar with NRF6_CAEEL
Px012696.1	2.6414	1.16E-90	2.93E-89	Lipase 3; similar with LIP3_DROME
Px009569.1	2.3697	7.48E-240	3.88E-238	Trypsin CFT-1; similar with TRYP_CHOFU
Px008913.1	2.3454	0.00E+00	0.00E+00	Acetylcholinesterase; similar with ACES_CULPI
Px011477.2	2.3341	5.77E-119	1.80E-117	Lipase 1; similar with LIP1_DROME
Px013101.1	2.2142	7.92E-05	2.35E-04	Nose resistant to fluoxetine protein 6; similar with NRF6_CAEEL
Px011788.2	2.1319	2.40E-67	5.04E-66	Pancreatic lipase-related protein 2; similar with LIPR2_BOVIN
Px005805.1	2.1005	1.53E-07	6.25E-07	Lipase 1; similar with LIP1_DROME
Px006720.1	1.9247	2.21E-05	7.08E-05	Glycerol-3-phosphate acyltransferase 4
Px006345.1	1.7781	1.52E-87	3.77E-86	Pancreatic lipase-related protein 2; similar with LIPR2_MYOCO
Px004131.5	1.7548	8.64E-41	1.23E-39	Calcium-independent phospholipase A2-gamma

Px004753.1	1.7057	1.64E-52	2.88E-51	Pancreatic lipase-related protein 2; similar with LIPR2_MYOCO
Px004662.1	1.6826	3.08E-13	1.91E-12	Lipase 1; similar with LIP1_DROME
Px001937.2	1.6560	5.20E-133	1.73E-131	Phosphatidate phosphatase LPIN2; similar with LPIN2_MOUSE
Px013003.3	1.5799	1.02E-12	6.16E-12	Neuropathy target esterase sws; similar with SWS_DROSE
Px017196.1	1.5696	1.86E-21	1.66E-20	Choline/ethanolamine kinase; similar with CHKB_HUMAN
Px011049.1	1.5512	8.68E-06	2.92E-05	Acetylcholinesterase; similar with ACES_FELCA
Px009940.2	1.4584	2.00E-08	8.84E-08	Acetylcholinesterase; similar with ACES_CULPI
Px003712.1	1.4525	1.46E-09	7.13E-09	Acetylcholinesterase; similar with ACES_CULPI
Px006262.1	1.4414	4.21E-28	4.61E-27	Lipase 3; similar with LIP3_DROME
Px004752.1	1.3734	0.00E+00	0.00E+00	Pancreatic lipase-related protein 2; similar with LIPR2_BOVIN
Px012695.1	1.3457	1.30E-14	8.69E-14	Lipase 1; similar with LIP1_DROME
Px002296.1	1.3429	6.45E-75	1.45E-73	Pancreatic triacylglycerol lipase; similar with LIPP_HUMAN
Px000371.1	1.2993	0.00E+00	0.00E+00	Lipase 1; similar with LIP1_DROME
Px009674.1	1.2991	5.95E-05	1.80E-04	N-alpha-acetyltransferase 40, NatD catalytic subunit
Px016094.1	1.2718	1.65E-05	5.39E-05	Group XV phospholipase A2; similar with PAG15_MOUSE
Px010099.1	1.2634	5.64E-17	4.26E-16	Phospholipase D1; similar with PLD1_RAT
Px005193.2	1.2375	6.57E-27	6.96E-26	Pancreatic lipase-related protein 2; similar with LIPR2_MYOCO
Px005646.1	1.2204	7.68E-07	2.93E-06	Nose resistant to fluoxetine protein 6; similar with NRF6_CAEEL
Px005804.1	1.1870	2.65E-10	1.37E-09	Lipase 1; similar with LIP1_DROME
Px011790.1	1.1858	3.36E-08	1.46E-07	Phosphatidylserine decarboxylase proenzyme
Px001894.8	1.1698	4.31E-20	3.65E-19	Ethanolamine-phosphate cytidylyltransferase
Px004750.1	1.1637	0.00E+00	0.00E+00	Pancreatic lipase-related protein 2; similar with LIPR2_RAT
Px017144.1	1.1569	1.61E-04	4.58E-04	1-acyl-sn-glycerol-3-phosphate acyltransferase beta
Px003579.1	1.1214	2.54E-20	2.17E-19	Glycerol-3-phosphate acyltransferase 1, mitochondrial
Px008093.1	1.0740	2.58E-22	2.36E-21	Nose resistant to fluoxetine protein 6; similar with NRF6_CAEEL
Px011476.1	1.0679	1.93E-04	5.39E-04	Lipase 3; similar with LIP3_DROME
Px002295.1	1.0591	2.25E-92	5.79E-91	Pancreatic lipase-related protein 2; similar with LIPR2_BOVIN
Px004200.1	1.0183	4.72E-21	4.15E-20	Putative phosphatidate phosphatase; similar with WUN_DROME
Px016246.1	-9.0336	3.73E-04	9.91E-04	Unknown functio
Px000102.1	-1.8916	1.29E-32	1.57E-31	Pancreatic lipase-related protein 3; similar with LIPR3_HUMAN
Px009691.1	-1.7400	2.15E-07	8.65E-07	Lipase 1; similar with LIP1_DROME
Px011624.1	-1.5253	7.85E-07	2.99E-06	Nose resistant to fluoxetine protein 6; similar with NRF6_CAEEL
Px006923.1	-1.4676	1.27E-24	1.25E-23	Glycerophosphoryl diester phosphodiesterase
Px004201.1	-1.2925	3.87E-106	1.11E-104	Putative phosphatidate phosphatase; similar with WUN_DROME

Involved in VEGF signaling pathway

Px008374.1	10.1228	1.11E-04	3.21E-04	Phosphatidylinositol-4,5-bisphosphate 3-kinase catalytic subunit delta isoform
Px009476.1	4.3986	3.12E-06	1.11E-05	Group 3 secretory phospholipase A2; similar with PA2G3_BOVIN
Px014687.1	2.9086	1.32E-27	1.42E-26	Putative uncharacterized protein GLEAN_08148
Px005596.1	2.7721	1.45E-36	1.92E-35	FERM and PDZ domain-containing protein 4
Px006086.2	2.6617	3.04E-05	9.56E-05	Uncharacterized protein K02A2.6; similar with YRD6_CAEEL
Px008372.1	2.4177	3.42E-08	1.48E-07	Phosphatidylinositol-4,5-bisphosphate 3-kinase catalytic subunit beta isoform
Px014553.1	2.1507	7.24E-12	4.15E-11	Mitogen-activated protein kinase ERK-A
Px003186.1	2.0295	2.84E-71	6.21E-70	RAC serine/threonine-protein kinase; similar with AKT1_DROME
Px013699.1	1.9424	2.80E-20	2.40E-19	GG17798; similar with B3NWL4_DROER
Px010399.1	1.8633	2.33E-07	9.35E-07	Sphingosine kinase 2; similar with SPHK2_MOUSE
Px000138.1	1.8328	1.68E-04	4.76E-04	Mitogen-activated protein kinase ERK-A
Px006878.1	1.7943	2.54E-04	6.97E-04	Protein kinase C, brain isozyme; similar with KPC1_DROME
Px004131.5	1.7548	8.64E-41	1.23E-39	Calcium-independent phospholipase A2-gamma
Px015426.1	1.5443	1.62E-09	7.88E-09	MAP kinase-activated protein kinase 2
Px003232.1	1.4984	1.17E-10	6.25E-10	1-phosphatidylinositol-4,5-bisphosphate phosphodiesterase gamma-1
Px009586.1	1.4046	1.13E-06	4.22E-06	Phosphatidylinositol 3-kinase regulatory subunit gamma
Px016244.2	1.3330	9.62E-36	1.26E-34	Calcium and integrin-binding family member 2
Px013159.1	1.2799	2.29E-07	9.21E-07	Calcium-binding protein p22; similar with CHP1_RAT

Px005140.1	1.2596	1.46E-06	5.39E-06	Focal adhesion kinase 1; similar with FAK1_RAT
Px008581.1	1.2495	4.68E-15	3.23E-14	PDZ and LIM domain protein Zasp; similar with ZASP_DROME
Px008798.1	1.1684	3.42E-59	6.55E-58	Serine/threonine-protein phosphatase 2B catalytic subunit 3
Px015291.1	1.1344	1.11E-12	6.70E-12	Mitogen-activated protein kinase 14B
Px015010.1	1.0950	1.19E-43	1.79E-42	Heat shock protein beta-1; similar with HSPB1_CHICK
Px008584.1	1.0409	6.82E-98	1.83E-96	PDZ and LIM domain protein Zasp; similar with ZASP_DROME
Px002728.1	1.0196	6.50E-19	5.27E-18	Calcium and integrin-binding family member 2

Involved in glycerolipid metabolism

Px016415.1	9.5474	6.00E-05	1.81E-04	Alcohol dehydrogenase [NADP+] A; similar with A1A1A_DANRE
Px014969.1	4.0027	9.70E-05	2.83E-04	Glycerol-3-phosphate acyltransferase 3
Px002516.1	3.9642	2.70E-08	1.18E-07	Glycerol kinase; similar with GLPK_RAT
Px008089.1	3.9186	0.00E+00	0.00E+00	Pancreatic lipase-related protein 2; similar with LIPR2_CAVPO
Px012694.3	3.5448	5.62E-16	4.03E-15	Lipase 1; similar with LIP1_DROME
Px006441.1	2.6454	0.00E+00	0.00E+00	Lipase member H; similar with LIPH_RAT
Px012696.1	2.6414	1.16E-90	2.93E-89	Lipase 3; similar with LIP3_DROME
Px016623.1	2.6198	4.98E-08	2.12E-07	Putative glycerol kinase 3; similar with GLPK3_HUMAN
Px011683.2	2.4698	9.12E-191	3.91E-189	Venom carboxylesterase-6; similar with EST6_APIME
Px001456.1	2.3739	1.19E-96	3.15E-95	Gastric triacylglycerol lipase; similar with LIPG_RAT
Px000104.1	2.3695	6.34E-13	3.87E-12	Pancreatic lipase-related protein 2; similar with LIPR2_CAVPO
Px011477.2	2.3341	5.77E-119	1.80E-117	Lipase 1; similar with LIP1_DROME
Px008088.2	2.3204	0.00E+00	0.00E+00	Lipoprotein lipase; similar with LIPL_CAVPO
Px011788.2	2.1319	2.40E-67	5.04E-66	Pancreatic lipase-related protein 2; similar with LIPR2_BOVIN
Px005876.1	2.1186	1.17E-33	1.47E-32	Patatin-like phospholipase domain-containing protein 2
Px005805.1	2.1005	1.53E-07	6.25E-07	Lipase 1; similar with LIP1_DROME
Px006720.1	1.9247	2.21E-05	7.08E-05	Glycerol-3-phosphate acyltransferase 4
Px006440.1	1.8690	1.16E-34	1.49E-33	Phospholipase A1 member A; similar with PLA1A_HUMAN
Px006345.1	1.7781	1.52E-87	3.77E-86	Pancreatic lipase-related protein 2; similar with LIPR2_MYOCO
Px004753.1	1.7057	1.64E-52	2.88E-51	Pancreatic lipase-related protein 2; similar with LIPR2_MYOCO
Px003649.1	1.6988	0.00E+00	0.00E+00	Esterase FE4; similar with ESTF_MYZPE
Px004662.1	1.6826	3.08E-13	1.91E-12	Lipase 1; similar with LIP1_DROME
Px001937.2	1.6560	5.20E-133	1.73E-131	Phosphatidate phosphatase LPIN2; similar with LPIN2_MOUSE
Px000132.1	1.5548	2.39E-41	3.44E-40	FGGY carbohydrate kinase domain-containing protein
Px011049.1	1.5512	8.68E-06	2.92E-05	Acetylcholinesterase; similar with ACES_FELCA
Px009940.2	1.4584	2.00E-08	8.84E-08	Acetylcholinesterase; similar with ACES_CULPI
Px003712.1	1.4525	1.46E-09	7.13E-09	Acetylcholinesterase; similar with ACES_CULPI
Px006262.1	1.4414	4.21E-28	4.61E-27	Lipase 3; similar with LIP3_DROME
Px013239.1	1.4393	8.23E-07	3.13E-06	Putative glycerol kinase 3; similar with GLPK3_HUMAN
Px000496.1	1.3885	8.80E-48	1.44E-46	Putative aldehyde dehydrogenase family 7 member A1 homolog
Px004752.1	1.3734	0.00E+00	0.00E+00	Pancreatic lipase-related protein 2; similar with LIPR2_BOVIN
Px000515.1	1.3647	1.85E-27	1.99E-26	Esterase FE4; similar with ESTF_MYZPE
Px012695.1	1.3457	1.30E-14	8.69E-14	Lipase 1; similar with LIP1_DROME
Px002296.1	1.3429	6.45E-75	1.45E-73	Pancreatic triacylglycerol lipase; similar with LIPP_HUMAN
Px000371.1	1.2993	0.00E+00	0.00E+00	Lipase 1; similar with LIP1_DROME
Px006008.1	1.2918	8.39E-47	1.35E-45	Alcohol dehydrogenase [NADP+]; similar with AK1A1_HUMAN
Px011443.1	1.2632	1.85E-11	1.03E-10	Esterase FE4; similar with ESTF_MYZPE
Px005193.2	1.2375	6.57E-27	6.96E-26	Pancreatic lipase-related protein 2; similar with LIPR2_MYOCO
Px012939.1	1.2048	1.82E-08	8.07E-08	Protein turtle; similar with TUTL_DROME
Px004571.1	1.2039	8.31E-100	2.29E-98	Aldose reductase; similar with ALDR_BOVIN
Px005804.1	1.1870	2.65E-10	1.37E-09	Lipase 1; similar with LIP1_DROME
Px004750.1	1.1637	0.00E+00	0.00E+00	Pancreatic lipase-related protein 2; similar with LIPR2_RAT
Px017144.1	1.1569	1.61E-04	4.58E-04	1-acyl-sn-glycerol-3-phosphate acyltransferase beta
Px011727.1	1.1383	4.42E-23	4.15E-22	Protein GCY; similar with GCY_YEAST
Px017996.1	1.1300	3.04E-23	2.87E-22	Acetylcholinesterase; similar with ACES_HUMAN

Px003579.1	1.1214	2.54E-20	2.17E-19	Glycerol-3-phosphate acyltransferase 1, mitochondrial
Px011476.1	1.0679	1.93E-04	5.39E-04	Lipase 3; similar with LIP3_DROME
Px002295.1	1.0591	2.25E-92	5.79E-91	Pancreatic lipase-related protein 2; similar with LIPR2_BOVIN
Px002733.2	1.0202	2.01E-161	7.66E-160	Aldehyde dehydrogenase, dimeric NADP-preferring
Px004200.1	1.0183	4.72E-21	4.15E-20	Putative phosphatidate phosphatase; similar with WUN_DROME
Px013493.2	-9.3785	3.73E-04	9.91E-04	Pancreatic lipase-related protein 2; similar with LIPR2_MYOCO
Px016246.1	-9.0336	3.73E-04	9.91E-04	Unknown functio
Px000102.1	-1.8916	1.29E-32	1.57E-31	Pancreatic lipase-related protein 3; similar with LIPR3_HUMAN
Px009691.1	-1.7400	2.15E-07	8.65E-07	Lipase 1; similar with LIP1_DROME
Px004201.1	-1.2925	3.87E-106	1.11E-104	Putative phosphatidate phosphatase; similar with WUN_DROME
Px012677.1	-1.2183	2.47E-04	6.79E-04	Lipase member H-A; similar with LIPHA_XENLA
Px004655.2	-1.0214	4.12E-49	6.87E-48	Aldo-keto reductase family 1 member C18

Involved in fat digestion and absorption

Px009476.1	4.3986	3.12E-06	1.11E-05	Group 3 secretory phospholipase A2; similar with PA2G3_BOVIN
Px008089.1	3.9186	0.00E+00	0.00E+00	Pancreatic lipase-related protein 2; similar with LIPR2_CAVPO
Px012694.3	3.5448	5.62E-16	4.03E-15	Lipase 1; similar with LIP1_DROME
Px006441.1	2.6454	0.00E+00	0.00E+00	Lipase member H; similar with LIPH_RAT
Px012696.1	2.6414	1.16E-90	2.93E-89	Lipase 3; similar with LIP3_DROME
Px013824.1	2.5061	4.55E-14	2.94E-13	Sensory neuron membrane protein 1; similar with SNMP1_PEDHC
Px011683.2	2.4698	9.12E-191	3.91E-189	Venom carboxylesterase-6; similar with EST6_APIME
Px001456.1	2.3739	1.19E-96	3.15E-95	Gastric triacylglycerol lipase; similar with LIPG_RAT
Px000104.1	2.3695	6.34E-13	3.87E-12	Pancreatic lipase-related protein 2; similar with LIPR2_CAVPO
Px011788.2	2.1319	2.40E-67	5.04E-66	Pancreatic lipase-related protein 2; similar with LIPR2_BOVIN
Px005805.1	2.1005	1.53E-07	6.25E-07	Lipase 1; similar with LIP1_DROME
Px001510.4	1.8746	7.52E-40	1.05E-38	Scavenger receptor class B member 1; similar with SCRB1_PIG
Px006345.1	1.7781	1.52E-87	3.77E-86	Pancreatic lipase-related protein 2; similar with LIPR2_MYOCO
Px004131.5	1.7548	8.64E-41	1.23E-39	Calcium-independent phospholipase A2-gamma
Px004753.1	1.7057	1.64E-52	2.88E-51	Pancreatic lipase-related protein 2; similar with LIPR2_MYOCO
Px003649.1	1.6988	0.00E+00	0.00E+00	Esterase FE4; similar with ESTF_MYZPE
Px017838.1	1.6880	1.08E-28	1.20E-27	ATP-binding cassette sub-family A member 12
Px005197.1	1.6696	5.48E-15	3.76E-14	Long-chain fatty acid transport protein 4
Px011049.1	1.5512	8.68E-06	2.92E-05	Acetylcholinesterase; similar with ACES_FELCA
Px001337.1	1.4834	0.00E+00	0.00E+00	Putative uncharacterized protein; similar with A9NYL2_PICSI
Px009940.2	1.4584	2.00E-08	8.84E-08	Acetylcholinesterase; similar with ACES_CULPI
Px003712.1	1.4525	1.46E-09	7.13E-09	Acetylcholinesterase; similar with ACES_CULPI
Px006262.1	1.4414	4.21E-28	4.61E-27	Lipase 3; similar with LIP3_DROME
Px011325.1	1.3737	4.72E-28	5.16E-27	Lysosome membrane protein 2; similar with SCRB2_MOUSE
Px004752.1	1.3734	0.00E+00	0.00E+00	Pancreatic lipase-related protein 2; similar with LIPR2_BOVIN
Px000515.1	1.3647	1.85E-27	1.99E-26	Esterase FE4; similar with ESTF_MYZPE
Px002296.1	1.3429	6.45E-75	1.45E-73	Pancreatic triacylglycerol lipase; similar with LIPP_HUMAN
Px011443.1	1.2632	1.85E-11	1.03E-10	Esterase FE4; similar with ESTF_MYZPE
Px005193.2	1.2375	6.57E-27	6.96E-26	Pancreatic lipase-related protein 2; similar with LIPR2_MYOCO
Px008150.1	1.2054	4.02E-05	1.24E-04	Scavenger receptor class B member 1; similar with SCRB1_PIG
Px006313.1	1.2054	4.59E-07	1.79E-06	Unknown functio
Px012939.1	1.2048	1.82E-08	8.07E-08	Protein turtle; similar with TUTL_DROME
Px005199.1	1.1968	3.62E-34	4.59E-33	Long-chain fatty acid transport protein 4
Px011529.1	1.1775	1.57E-37	2.10E-36	Apolipophorins; similar with APLP_LOCMI
Px004510.1	1.1686	1.57E-18	1.25E-17	ABC transporter G family member 20; similar with ABCGK_DICDI
Px004750.1	1.1637	0.00E+00	0.00E+00	Pancreatic lipase-related protein 2; similar with LIPR2_RAT
Px017144.1	1.1569	1.61E-04	4.58E-04	1-acyl-sn-glycerol-3-phosphate acyltransferase beta
Px017996.1	1.1300	3.04E-23	2.87E-22	Acetylcholinesterase; similar with ACES_HUMAN
Px002295.1	1.0591	2.25E-92	5.79E-91	Pancreatic lipase-related protein 2; similar with LIPR2_BOVIN
Px013659.1	1.0189	6.26E-11	3.38E-10	ATP-binding cassette sub-family A member 1

Px004200.1	1.0183	4.72E-21	4.15E-20	Putative phosphatidate phosphatase; similar with WUN_DROME
Px000102.1	-1.8916	1.29E-32	1.57E-31	Pancreatic lipase-related protein 3; similar with LIPR3_HUMAN
Px009691.1	-1.7400	2.15E-07	8.65E-07	Lipase 1; similar with LIP1_DROME
Px008080.1	-1.3571	7.20E-06	2.45E-05	Lysosome membrane protein 2; similar with SCRB2_HUMAN
Px004201.1	-1.2925	3.87E-106	1.11E-104	Putative phosphatidate phosphatase; similar with WUN_DROME
Px012677.1	-1.2183	2.47E-04	6.79E-04	Lipase member H-A; similar with LIPHA_XENLA
Px001589.1	-1.2061	1.74E-07	7.04E-07	Scavenger receptor class B member 1; similar with SCRB1_CRIGR

Table E. A list of DEGs enriched in the *P. xylostella* pathways at 36 hpt_I versus 24 hpt_I.

Gene ID	log_2 R	P-value	FDR	Annotation
**Involved in alpha-Linolenic acid metabolism**				
Px009691.1	3.4211	1.35E-22	1.59E-21	Lipase 1; similar with LIP1_DROME
Px012560.1	3.3198	5.27E-05	1.91E-04	Lipase member H-B; similar with LIPHB_XENLA
Px002439.1	2.8901	8.15E-09	4.70E-08	85 kDa calcium-independent phospholipase A2
Px008299.1	1.5466	1.31E-43	2.60E-42	Pancreatic triacylglycerol lipase; similar with LIPP_RABIT
Px012696.1	1.4788	9.94E-81	3.24E-79	Lipase 3; similar with LIP3_DROME
Px004131.5	1.4770	1.90E-34	3.14E-33	Calcium-independent phospholipase A2-gamma
Px011476.1	1.4379	1.22E-13	9.89E-13	Lipase 3; similar with LIP3_DROME
Px006345.1	1.3604	1.89E-137	9.14E-136	Pancreatic lipase-related protein 2; similar with LIPR2_MYOCO
Px001913.1	1.3119	1.10E-58	2.72E-57	Gastric triacylglycerol lipase; similar with LIPG_RAT
Px003273.1	1.2793	6.98E-08	3.65E-07	Pancreatic lipase-related protein 2; similar with LIPR2_CAVPO
Px005804.1	1.2619	8.87E-38	1.58E-36	Lipase 1; similar with LIP1_DROME
Px007883.1	1.1586	1.71E-20	1.90E-19	Probable medium-chain specific acyl-CoA dehydrogenase, mitochondrial
Px011475.1	1.1550	1.37E-130	6.32E-129	Lipase 3; similar with LIP3_DROME
Px012695.1	1.1284	9.95E-39	1.81E-37	Lipase 1; similar with LIP1_DROME
Px004753.1	1.1219	1.62E-67	4.55E-66	Pancreatic lipase-related protein 2; similar with LIPR2_MYOCO
Px004662.1	1.0372	2.78E-05	1.05E-04	Lipase 1; similar with LIP1_DROME
Px017173.1	-1.0855	9.56E-10	5.99E-09	Lipase 1; similar with LIP1_DROME
Px016338.1	-1.0692	1.84E-51	4.13E-50	Lipase 1; similar with LIP1_DROME
**Involved in jak-STAT signaling pathway**				
Px008866.1	4.0169	3.36E-23	4.06E-22	Chorion peroxidase; similar with PERC_DROME
Px013761.3	3.4453	1.68E-05	6.54E-05	Peroxidase; similar with PERO_DROME
Px016174.1	2.5233	6.89E-09	4.01E-08	Suppressor of cytokine signaling 5
Px017055.1	2.3629	1.93E-05	7.43E-05	Signal transducer and activator of transcription short form
Px006617.2	2.2296	3.60E-19	3.80E-18	Chorion peroxidase; similar with PERC_DROME
Px001416.2	1.9193	4.72E-06	1.98E-05	Son of sevenless homolog 1; similar with SOS1_MOUSE
Px000279.1	1.7250	2.21E-21	2.54E-20	Suppressor of cytokine signaling 2; similar with SOCS2_RAT
Px007394.2	1.6531	2.52E-69	7.27E-68	Peroxidasin homolog; similar with PXDN_MOUSE
Px014219.1	1.3472	8.72E-13	6.72E-12	Signal transducer and transcription activator
Px009359.1	1.3141	8.23E-12	5.97E-11	Cytokine receptor; similar with DOME_DROME
Px009586.1	1.2474	1.51E-06	6.72E-06	Phosphatidylinositol 3-kinase regulatory subunit gamma
Px002890.1	1.1629	7.37E-35	1.23E-33	Sorting nexin-33; similar with SNX33_MOUSE
Px012070.1	1.1354	5.24E-12	3.84E-11	TBC1 domain family member 23; similar with TBC23_PONAB
Px017291.1	1.1127	9.26E-05	3.23E-04	TOM1-like protein 2; similar with TM1L2_HUMAN
Px001125.2	1.0085	2.57E-07	1.26E-06	Unknown functio
Px006512.1	-1.2636	2.82E-11	1.98E-10	RNA-binding protein 41; similar with RBM41_HUMAN
**Involved in complement and coagulation cascades**				
Px003000.1	12.5364	5.20E-38	9.24E-37	Transmembrane protease serine 6; similar with TMPS6_MOUSE
Px004167.1	5.4149	1.64E-12	1.25E-11	Plasma kallikrein; similar with KLKB1_MOUSE
Px015905.1	4.3586	3.39E-50	7.48E-49	Transmembrane protease serine 6; similar with TMPS6_MOUSE
Px006453.1	3.7183	9.28E-07	4.24E-06	Transmembrane protease serine 6; similar with TMPS6_MOUSE
Px007757.1	2.6932	9.34E-26	1.23E-24	Putative trypsin Inhibitor like cysteine rich domain protein
Px004168.1	2.3782	3.79E-09	2.25E-08	Plasma kallikrein; similar with KLKB1_HUMAN
Px005947.1	1.7534	6.64E-08	3.49E-07	Serine proteinase stubble; similar with STUB_DROME
Px013945.1	1.6753	7.42E-09	4.31E-08	Pregnancy zone protein; similar with PZP_HUMAN
Px016053.1	1.4991	6.82E-18	6.86E-17	Trypsin; similar with TRYP_PHACE
Px006790.1	1.4338	6.89E-25	8.80E-24	Hemocytin; similar with HMCT_BOMMO
Px013431.3	1.2846	9.04E-21	1.01E-19	Pregnancy zone protein; similar with PZP_HUMAN
Px006789.3	1.2682	3.55E-247	2.82E-245	Hemocytin; similar with HMCT_BOMMO

Px007556.1	-1.0904	6.20E-16	5.70E-15	Plasma kallikrein; similar with KLKB1_HUMAN

Involved in fat digestion and absorption

Px009691.1	3.4211	1.35E-22	1.59E-21	Lipase 1; similar with LIP1_DROME
Px012560.1	3.3198	5.27E-05	1.91E-04	Lipase member H-B; similar with LIPHB_XENLA
Px002439.1	2.8901	8.15E-09	4.70E-08	85 kDa calcium-independent phospholipase A2
Px004112.1	2.0072	1.43E-07	7.21E-07	Phosphatidate phosphatase PPAPDC1B
Px004046.1	1.8369	2.68E-12	2.01E-11	Esterase FE4; similar with ESTF_MYZPE
Px008089.1	1.6672	0.00E+00	0.00E+00	Pancreatic lipase-related protein 2; similar with LIPR2_CAVPO
Px008299.1	1.5466	1.31E-43	2.60E-42	Pancreatic triacylglycerol lipase; similar with LIPP_RABIT
Px012696.1	1.4788	9.94E-81	3.24E-79	Lipase 3; similar with LIP3_DROME
Px004131.5	1.4770	1.90E-34	3.14E-33	Calcium-independent phospholipase A2-gamma
Px005198.1	1.4333	2.45E-31	3.80E-30	Long-chain fatty acid transport protein 4
Px003649.1	1.3690	0.00E+00	0.00E+00	Esterase FE4; similar with ESTF_MYZPE
Px006345.1	1.3604	1.89E-137	9.14E-136	Pancreatic lipase-related protein 2; similar with LIPR2_MYOCO
Px000644.1	1.3260	7.39E-08	3.85E-07	Pancreatic triacylglycerol lipase; similar with LIPP_MOUSE
Px003273.1	1.2793	6.98E-08	3.65E-07	Pancreatic lipase-related protein 2; similar with LIPR2_CAVPO
Px004200.1	1.2352	1.36E-36	2.37E-35	Putative phosphatidate phosphatase
Px017838.1	1.1994	1.28E-19	1.37E-18	ATP-binding cassette sub-family A member 12
Px001456.1	1.1395	3.96E-60	1.01E-58	Gastric triacylglycerol lipase; similar with LIPG_RAT
Px011443.1	1.1348	1.60E-12	1.22E-11	Esterase FE4; similar with ESTF_MYZPE
Px004753.1	1.1219	1.62E-67	4.55E-66	Pancreatic lipase-related protein 2; similar with LIPR2_MYOCO
Px008150.1	1.0828	3.26E-08	1.77E-07	Scavenger receptor class B member 1; similar with SCRB1_PIG
Px011529.1	1.0687	3.97E-67	1.11E-65	Apolipophorins; similar with APLP_LOCMI
Px011335.1	1.0468	1.92E-06	8.43E-06	Scavenger receptor class B member 1
Px008997.1	1.0372	2.78E-05	1.05E-04	Juvenile hormone esterase; similar with ESTJ_HELVI
Px017144.1	1.0267	4.23E-05	1.56E-04	1-acyl-sn-glycerol-3-phosphate acyltransferase beta
Px004160.1	-1.4027	2.83E-06	1.22E-05	Pancreatic lipase-related protein 2; similar with LIPR2_RAT

Involved in amino sugar and nucleotide sugar metabolism

Px009803.1	10.8775	1.02E-07	5.23E-07	Probable chitinase 2; similar with CHIT2_DROME
Px008062.1	5.8146	2.04E-156	1.10E-154	Endochitinase; similar with CHIT_MANSE
Px001433.1	3.9896	2.70E-08	1.48E-07	Probable chitinase 2; similar with CHIT2_DROME
Px011561.1	2.1976	1.57E-75	4.80E-74	Probable chitinase 3; similar with CHIT3_DROME
Px016709.1	2.0403	4.32E-31	6.66E-30	Glucosamine-6-phosphate isomerase
Px005496.1	1.9438	1.51E-18	1.56E-17	GDP-mannose 4,6 dehydratase; similar with GMDS_DROME
Px009279.1	1.8168	3.74E-64	9.98E-63	Chitotriosidase-1; similar with CHIT1_HUMAN
Px012913.1	1.7704	7.66E-144	3.85E-142	Chitin synthase 6; similar with CHS6_USTMA
Px017503.2	1.6594	3.98E-08	2.14E-07	Probable chitinase 2; similar with CHIT2_DROME
Px012404.1	1.5652	3.69E-36	6.38E-35	Probable chitinase 3; similar with CHIT3_DROME
Px006848.1	1.4775	2.65E-16	2.48E-15	UDP-glucose 6-dehydrogenase; similar with UGDH_DROME
Px016080.1	1.3964	8.21E-05	2.89E-04	Glucosamine-6-phosphate isomerase
Px005479.2	1.3390	4.90E-11	3.39E-10	Chitooligosaccharidolytic beta-N-acetylglucosaminidase
Px002919.1	1.3073	1.52E-15	1.37E-14	Putative N-acetylglucosamine-6-phosphate deacetylase
Px003540.1	1.2878	3.64E-22	4.26E-21	Hexokinase type 2; similar with HXK2_DROME
Px016079.1	1.2055	7.03E-06	2.89E-05	Glucosamine-6-phosphate isomerase; similar with GNPI_DROPS
Px014908.1	1.2014	5.74E-05	2.07E-04	Mannose-1-phosphate guanyltransferase beta
Px000053.3	1.1695	1.85E-19	1.97E-18	N-acylneuraminate-9-phosphatase; similar with NANP_RAT
Px003769.1	1.1263	2.76E-31	4.27E-30	Hexokinase type 2; similar with HXK2_DROME
Px013778.1	1.0955	3.00E-132	1.40E-130	Chitin synthase 3; similar with CHS3_USTMA
Px014904.1	1.0156	1.44E-08	8.12E-08	Mannose-1-phosphate guanyltransferase beta
Px008741.1	-1.2612	1.25E-37	2.22E-36	Chondroitin proteoglycan 2; similar with CPG2_CAEBR

Involved in glycerophospholipid metabolism

Px015388.1	3.7672	5.33E-10	3.43E-09	Nose resistant to fluoxetine protein 6; similar with NRF6_CAEEL
Px009691.1	3.4211	1.35E-22	1.59E-21	Lipase 1; similar with LIP1_DROME
Px012560.1	3.3198	5.27E-05	1.91E-04	Lipase member H-B; similar with LIPHB_XENLA
Px005418.1	3.0303	4.90E-07	2.32E-06	Nose resistant to fluoxetine protein 6; similar with NRF6_CAEEL
Px002439.1	2.8901	8.15E-09	4.70E-08	85 kDa calcium-independent phospholipase A2
Px013101.1	2.6932	1.90E-09	1.16E-08	Nose resistant to fluoxetine protein 6; similar with NRF6_CAEEL
Px004112.1	2.0072	1.43E-07	7.21E-07	Phosphatidate phosphatase PPAPDC1B
Px000040.1	1.9896	9.64E-05	3.35E-04	N-alpha-acetyltransferase 40, NatD catalytic subunit
Px008299.1	1.5466	1.31E-43	2.60E-42	Pancreatic triacylglycerol lipase; similar with LIPP_RABIT
Px014079.1	1.5138	1.12E-11	8.07E-11	Nose resistant to fluoxetine protein 6; similar with NRF6_CAEEL
Px012696.1	1.4788	9.94E-81	3.24E-79	Lipase 3; similar with LIP3_DROME
Px004131.5	1.4770	1.90E-34	3.14E-33	Calcium-independent phospholipase A2-gamma
Px011476.1	1.4379	1.22E-13	9.89E-13	Lipase 3; similar with LIP3_DROME
Px006345.1	1.3604	1.89E-137	9.14E-136	Pancreatic lipase-related protein 2; similar with LIPR2_MYOCO
Px001913.1	1.3119	1.10E-58	2.72E-57	Gastric triacylglycerol lipase; similar with LIPG_RAT
Px003273.1	1.2793	6.98E-08	3.65E-07	Pancreatic lipase-related protein 2; similar with LIPR2_CAVPO
Px005804.1	1.2619	8.87E-38	1.58E-36	Lipase 1; similar with LIP1_DROME
Px004200.1	1.2352	1.36E-36	2.37E-35	Putative phosphatidate phosphatase; similar with WUN_DROME
Px008142.1	1.1823	5.57E-07	2.61E-06	Meteorin-like protein; similar with METRL_RAT
Px011475.1	1.1550	1.37E-130	6.32E-129	Lipase 3; similar with LIP3_DROME
Px005172.2	1.1389	3.80E-07	1.82E-06	Group XV phospholipase A2; similar with PAG15_HUMAN
Px012695.1	1.1284	9.95E-39	1.81E-37	Lipase 1; similar with LIP1_DROME
Px004753.1	1.1219	1.62E-67	4.55E-66	Pancreatic lipase-related protein 2; similar with LIPR2_MYOCO
Px003579.1	1.1187	2.25E-27	3.11E-26	Glycerol-3-phosphate acyltransferase 1, mitochondrial
Px014043.4	1.1083	3.62E-06	1.54E-05	Nose resistant to fluoxetine protein 6; similar with NRF6_CAEEL
Px003528.3	1.0998	7.23E-05	2.58E-04	Nose resistant to fluoxetine protein 6; similar with NRF6_CAEEL
Px006720.1	1.0776	4.40E-05	1.62E-04	Glycerol-3-phosphate acyltransferase 4
Px013003.3	1.0713	2.35E-06	1.02E-05	Neuropathy target esterase sws; similar with SWS_DROSE
Px004662.1	1.0372	2.78E-05	1.05E-04	Lipase 1; similar with LIP1_DROME
Px017144.1	1.0267	4.23E-05	1.56E-04	1-acyl-sn-glycerol-3-phosphate acyltransferase beta
Px017173.1	-1.0855	9.56E-10	5.99E-09	Lipase 1; similar with LIP1_DROME
Px016338.1	-1.0692	1.84E-51	4.13E-50	Lipase 1; similar with LIP1_DROME

Involved in tyrosine metabolism

Px008866.1	4.0169	3.36E-23	4.06E-22	Chorion peroxidase; similar with PERC_DROME
Px015388.1	3.7672	5.33E-10	3.43E-09	Nose resistant to fluoxetine protein 6; similar with NRF6_CAEEL
Px013761.3	3.4453	1.68E-05	6.54E-05	#N/A
Px005418.1	3.0303	4.90E-07	2.32E-06	Nose resistant to fluoxetine protein 6; similar with NRF6_CAEEL
Px013101.1	2.6932	1.90E-09	1.16E-08	Nose resistant to fluoxetine protein 6; similar with NRF6_CAEEL
Px006617.2	2.2296	3.60E-19	3.80E-18	Chorion peroxidase; similar with PERC_DROME
Px000040.1	1.9896	9.64E-05	3.35E-04	N-alpha-acetyltransferase 40, NatD catalytic subunit
Px011987.1	1.7765	2.20E-11	1.56E-10	Amine oxidase [flavin-containing] B; similar with AOFB_MOUSE
Px007394.2	1.6531	2.52E-69	7.27E-68	Peroxidasin homolog; similar with PXDN_MOUSE
Px014079.1	1.5138	1.12E-11	8.07E-11	Nose resistant to fluoxetine protein 6; similar with NRF6_CAEEL
Px007028.1	1.4107	0.00E+00	0.00E+00	Arylphorin subunit alpha; similar with ARYA_MANSE
Px000359.2	1.3395	1.41E-06	6.28E-06	Alpha-methyldopa hypersensitive protein
Px007030.1	1.1691	0.00E+00	0.00E+00	Basic juvenile hormone-suppressible protein 2
Px006820.1	1.1363	0.00E+00	0.00E+00	Basic juvenile hormone-suppressible protein 1
Px012208.1	1.1156	1.08E-07	5.54E-07	Probable flavin-containing monoamine oxidase A
Px014043.4	1.1083	3.62E-06	1.54E-05	Nose resistant to fluoxetine protein 6; similar with NRF6_CAEEL
Px003528.3	1.0998	7.23E-05	2.58E-04	Nose resistant to fluoxetine protein 6; similar with NRF6_CAEEL
Px000224.1	-1.9102	1.61E-07	8.07E-07	4-hydroxyphenylpyruvate dioxygenase

Involved in glycerolipid metabolism

Px009691.1	3.4211	1.35E-22	1.59E-21	Lipase 1; similar with LIP1_DROME
Px012560.1	3.3198	5.27E-05	1.91E-04	Lipase member H-B; similar with LIPHB_XENLA
Px014975.1	3.0828	8.77E-06	3.57E-05	Alcohol dehydrogenase [NADP+] A
Px004112.1	2.0072	1.43E-07	7.21E-07	Phosphatidate phosphatase PPAPDC1B
Px004046.1	1.8369	2.68E-12	2.01E-11	Esterase FE4; similar with ESTF_MYZPE
Px008089.1	1.6672	0.00E+00	0.00E+00	Pancreatic lipase-related protein 2; similar with LIPR2_CAVPO
Px008299.1	1.5466	1.31E-43	2.60E-42	Pancreatic triacylglycerol lipase; similar with LIPP_RABIT
Px012696.1	1.4788	9.94E-81	3.24E-79	Lipase 3; similar with LIP3_DROME
Px011476.1	1.4379	1.22E-13	9.89E-13	Lipase 3; similar with LIP3_DROME
Px003649.1	1.3690	0.00E+00	0.00E+00	Esterase FE4; similar with ESTF_MYZPE
Px006345.1	1.3604	1.89E-137	9.14E-136	Pancreatic lipase-related protein 2; similar with LIPR2_MYOCO
Px005876.1	1.3414	6.47E-31	9.92E-30	Patatin-like phospholipase domain-containing protein 2
Px000644.1	1.3260	7.39E-08	3.85E-07	Pancreatic triacylglycerol lipase; similar with LIPP_MOUSE
Px001913.1	1.3119	1.10E-58	2.72E-57	Gastric triacylglycerol lipase; similar with LIPG_RAT
Px003273.1	1.2793	6.98E-08	3.65E-07	Pancreatic lipase-related protein 2; similar with LIPR2_CAVPO
Px005804.1	1.2619	8.87E-38	1.58E-36	Lipase 1; similar with LIP1_DROME
Px004200.1	1.2352	1.36E-36	2.37E-35	Putative phosphatidate phosphatase
Px008142.1	1.1823	5.57E-07	2.61E-06	Meteorin-like protein; similar with METRL_RAT
Px000132.1	1.1786	8.56E-47	1.79E-45	FGGY carbohydrate kinase domain-containing protein
Px011475.1	1.1550	1.37E-130	6.32E-129	Lipase 3; similar with LIP3_DROME
Px001456.1	1.1395	3.96E-60	1.01E-58	Gastric triacylglycerol lipase; similar with LIPG_RAT
Px011443.1	1.1348	1.60E-12	1.22E-11	Esterase FE4; similar with ESTF_MYZPE
Px012695.1	1.1284	9.95E-39	1.81E-37	Lipase 1; similar with LIP1_DROME
Px004753.1	1.1219	1.62E-67	4.55E-66	Pancreatic lipase-related protein 2; similar with LIPR2_MYOCO
Px003579.1	1.1187	2.25E-27	3.11E-26	Glycerol-3-phosphate acyltransferase 1, mitochondrial
Px006720.1	1.0776	4.40E-05	1.62E-04	Glycerol-3-phosphate acyltransferase 4
Px008997.1	1.0372	2.78E-05	1.05E-04	Juvenile hormone esterase; similar with ESTJ_HELVI
Px004662.1	1.0372	2.78E-05	1.05E-04	Lipase 1; similar with LIP1_DROME
Px017144.1	1.0267	4.23E-05	1.56E-04	1-acyl-sn-glycerol-3-phosphate acyltransferase beta
Px004160.1	-1.4027	2.83E-06	1.22E-05	Pancreatic lipase-related protein 2; similar with LIPR2_RAT
Px004110.1	-1.3990	3.91E-12	2.89E-11	Aldose reductase; similar with ALDR_RABIT
Px012956.1	-1.2100	1.62E-10	1.08E-09	Aldose reductase; similar with ALDR_RABIT
Px017173.1	-1.0855	9.56E-10	5.99E-09	Lipase 1; similar with LIP1_DROME
Px016338.1	-1.0692	1.84E-51	4.13E-50	Lipase 1; similar with LIP1_DROME

Table F. A list of DEGs enriched in the *P. xylostella* pathways at 48 hpt_{I} versus 36 hpt_{I}.

Gene ID	$\log_2$ R	P-value	FDR	Annotation
**Involved in protein digestion and absorption**				
Px006007.1	1.8646	6.62E-06	3.41E-05	Unknown function
Px013814.3	1.3796	2.93E-07	1.75E-06	Sodium/potassium-transporting ATPase subunit beta-2
Px008393.1	1.2783	4.96E-08	3.17E-07	Nck-associated protein 5; similar with NCKP5_HUMAN
Px014256.1	-4.8476	3.36E-08	2.18E-07	Unknown function
Px005554.1	-4.5132	1.70E-06	9.36E-06	Chymotrypsin-1; similar with CTR1_ANOGA
Px013747.1	-3.3917	2.28E-15	2.37E-14	Proton-coupled amino acid transporter 1
Px005373.1	-3.1779	1.15E-43	2.34E-42	Carboxypeptidase B; similar with CBPB_ASTFL
Px010313.2	-3.0785	4.07E-85	1.31E-83	Serine proteinase stubble; similar with STUB_DROME
Px015280.1	-3.0485	6.83E-10	5.07E-09	Trypsin CFT-1; similar with TRYP_CHOFU
Px011754.1	-2.9282	1.53E-16	1.66E-15	Proton-coupled amino acid transporter 1
Px011887.1	-2.8795	0.00E+00	0.00E+00	Trypsin, alkaline C; similar with TRYC_MANSE
Px001649.1	-2.8762	9.90E-53	2.29E-51	MAM domain-containing glycosylphosphatidylinositol anchor protein 1
Px007670.1	-2.8376	8.00E-36	1.42E-34	Proclotting enzyme; similar with PCE_TACTR
Px012813.1	-2.5746	1.13E-04	4.76E-04	Serine proteinase stubble; similar with STUB_DROME
Px004167.1	-2.5442	5.53E-08	3.53E-07	Plasma kallikrein; similar with KLKB1_MOUSE
Px015279.1	-2.4814	8.78E-14	8.44E-13	Trypsin, alkaline A; similar with TRYA_MANSE
Px009395.1	-2.3608	7.33E-17	8.06E-16	Proclotting enzyme; similar with PCE_TACTR
Px016053.1	-2.2883	3.96E-32	6.55E-31	Trypsin; similar with TRYP_PHACE
Px007600.1	-2.1704	8.57E-179	4.82E-177	Chymotrypsin-1; similar with CTR1_ANOGA
Px015278.1	-2.1543	1.50E-17	1.69E-16	Trypsin, alkaline A; similar with TRYA_MANSE
Px015276.1	-2.1364	2.14E-33	3.68E-32	Trypsin CFT-1; similar with TRYP_CHOFU
Px009568.1	-2.0485	8.77E-07	4.95E-06	Trypsin, alkaline C; similar with TRYC_MANSE
Px012570.1	-1.9507	0.00E+00	0.00E+00	Trypsin CFT-1; similar with TRYP_CHOFU
Px002586.1	-1.9496	8.66E-27	1.26E-25	Trypsin CFT-1; similar with TRYP_CHOFU
Px008406.1	-1.9282	2.58E-08	1.70E-07	Serine proteinase stubble; similar with STUB_DROME
Px002587.1	-1.9104	0.00E+00	0.00E+00	Trypsin CFT-1; similar with TRYP_CHOFU
Px005947.1	-1.8827	5.51E-09	3.84E-08	Serine proteinase stubble; similar with STUB_DROME
Px007902.1	-1.8728	1.80E-17	2.03E-16	Collagenase; similar with COGS_HYPLI
Px002440.1	-1.8376	2.21E-18	2.58E-17	Trypsin, alkaline B; similar with TRYB_MANSE
Px000996.2	-1.8376	2.80E-40	5.43E-39	Carboxypeptidase A2; similar with CBPA2_MOUSE
Px009637.1	-1.8225	4.86E-12	4.22E-11	Trypsin CFT-1; similar with TRYP_CHOFU
Px002993.1	-1.8035	0.00E+00	0.00E+00	Zinc metalloproteinase nas-4; similar with NAS4_CAEEL
Px014882.1	-1.7867	4.24E-58	1.03E-56	Trypsin, alkaline C; similar with TRYC_MANSE
Px007903.1	-1.7472	0.00E+00	0.00E+00	Collagenase; similar with COGS_HYPLI
Px016054.1	-1.7315	2.91E-18	3.38E-17	Trypsin; similar with TRYP_PHACE
Px002589.1	-1.6905	8.93E-61	2.24E-59	Trypsin, alkaline A; similar with TRYA_MANSE
Px009567.1	-1.6684	1.19E-67	3.20E-66	Trypsin, alkaline B; similar with TRYB_MANSE
Px000991.6	-1.5978	1.12E-135	5.05E-134	Carboxypeptidase O; similar with CBPO_BOVIN
Px007599.1	-1.5923	0.00E+00	0.00E+00	Chymotrypsin-1; similar with CTR1_ANOGA
Px011097.1	-1.5746	9.30E-08	5.81E-07	Serine proteinase stubble; similar with STUB_DROME
Px002442.1	-1.5451	1.48E-08	9.93E-08	Trypsin, alkaline C; similar with TRYC_MANSE
Px016055.1	-1.5361	1.17E-17	1.33E-16	Trypsin, alkaline C; similar with TRYC_MANSE
Px004168.1	-1.4433	1.50E-05	7.32E-05	Plasma kallikrein; similar with KLKB1_HUMAN
Px000994.1	-1.3902	0.00E+00	0.00E+00	Zinc carboxypeptidase A 1; similar with CBPA1_DROPS
Px007357.2	-1.3810	0.00E+00	0.00E+00	Serine proteinase stubble; similar with STUB_DROME
Px001804.1	-1.3798	3.28E-12	2.87E-11	Trypsin, alkaline B; similar with TRYB_MANSE
Px007621.1	-1.3710	1.67E-07	1.02E-06	Trypsin CFT-1; similar with TRYP_CHOFU

Px001990.2	-1.3597	3.72E-51	8.50E-50	Carboxypeptidase B; similar with CBPB_ASTFL
Px008830.1	-1.3365	7.12E-24	9.79E-23	Trypsin, alkaline C; similar with TRYC_MANSE
Px015277.1	-1.3334	1.86E-42	3.75E-41	Trypsin CFT-1; similar with TRYP_CHOFU
Px011428.1	-1.3275	9.95E-07	5.59E-06	Peptide transporter family 1; similar with PEPT1_DROME
Px005240.1	-1.2961	1.15E-27	1.71E-26	Trypsin, alkaline A; similar with TRYA_MANSE
Px001262.4	-1.2953	1.18E-112	4.58E-111	Collagen alpha-1(IV) chain; similar with CO4A1_DROME
Px007901.1	-1.2804	1.02E-94	3.49E-93	Chymotrypsin BI; similar with CTRB1_LITVA
Px015049.1	-1.2783	3.98E-12	3.47E-11	Venom dipeptidyl peptidase 4; similar with VDDP4_VESVU
Px007675.1	-1.2333	2.71E-176	1.50E-174	Collagenase; similar with COGS_HYPLI
Px000992.1	-1.1391	3.27E-295	2.88E-293	Carboxypeptidase A1; similar with CBPA1_BOVIN
Px013212.4	-1.1366	5.72E-50	1.28E-48	Inactive dipeptidyl peptidase 10; similar with DPP10_HUMAN
Px013919.1	-1.1300	0.00E+00	0.00E+00	Collagen alpha-4(VI) chain; similar with CO6A4_MOUSE
Px005244.1	-1.1143	2.04E-08	1.36E-07	Trypsin CFT-1; similar with TRYP_CHOFU
Px002861.1	-1.0790	0.00E+00	0.00E+00	Trypsin, alkaline C; similar with TRYC_MANSE
Px005239.1	-1.0638	4.56E-87	1.48E-85	Trypsin, alkaline A; similar with TRYA_MANSE
Px014592.1	-1.0450	1.60E-19	1.93E-18	Trypsin CFT-1; similar with TRYP_CHOFU
Px013377.1	-1.0384	2.02E-39	3.87E-38	Trypsin; similar with TRYP_PHACE
Px013665.3	-1.0337	7.86E-16	8.29E-15	Collagenase; similar with COGS_HYPLI
Px002865.1	-1.0134	0.00E+00	0.00E+00	Trypsin 5G1; similar with TRY5_AEDAE
Px007900.1	-1.0083	0.00E+00	0.00E+00	Chymotrypsin BI; similar with CTRB1_LITVA
Px016056.1	-1.0042	0.00E+00	0.00E+00	Trypsin, alkaline A; similar with TRYA_MANSE
Px011889.1	-1.0003	1.94E-06	1.06E-05	Achelase-1; similar with ACH1_LONAC
Px005341.1	-1.0002	6.21E-27	9.07E-26	Collagenase; similar with COGS_HYPLI

Involved in drug metabolism - other enzymes

Px013955.1	1.9862	1.32E-07	8.12E-07	Neuroligin-1; similar with NLGN1_HUMAN
Px009818.1	1.6813	1.48E-158	7.58E-157	Putative aldehyde oxidase-like protein; similar with ALDOL_ORYSJ
Px015080.1	1.5382	2.00E-23	2.70E-22	Ecdysteroid UDP-glucosyltransferase; similar with UDPE_NPVMB
Px013145.1	1.0351	2.01E-231	1.45E-229	Cytochrome P450 6B7; similar with CP6B7_HELAM
Px015079.1	-3.1595	1.17E-07	7.28E-07	Ecdysteroid UDP-glucosyltransferase; similar with UDPE_NPVSL
Px004854.1	-2.7504	3.14E-20	3.86E-19	Ecdysteroid UDP-glucosyltransferase; similar with UDPE_NPVSL
Px014217.1	-1.9896	7.35E-20	8.93E-19	Cytochrome P450 6k1; similar with CP6K1_BLAGE
Px000872.2	-1.9896	8.91E-10	6.55E-09	Ecdysteroid UDP-glucosyltransferase; similar with UDPE_NPVSL
Px005900.1	-1.9164	8.05E-36	1.43E-34	Cytochrome P450 6B2; similar with CP6B2_HELAM
Px011049.1	-1.8032	1.05E-22	1.38E-21	Acetylcholinesterase; similar with ACES_FELCA
Px015078.1	-1.6900	8.07E-07	4.58E-06	Ecdysteroid UDP-glucosyltransferase; similar with UDPE_NPVSL
Px014216.1	-1.5409	2.47E-29	3.84E-28	Cytochrome P450 6B6; similar with CP6B6_HELAM
Px013958.1	-1.5363	1.75E-141	8.12E-140	Cytochrome P450 6k1; similar with CP6K1_BLAGE
Px002736.1	-1.3736	3.57E-192	2.18E-190	Venom carboxylesterase-6; similar with EST6_APIME
Px006292.1	-1.3115	1.53E-13	1.46E-12	Ecdysteroid UDP-glucosyltransferase; similar with UDPE_NPVSL
Px002735.1	-1.2993	2.03E-97	7.10E-96	Esterase FE4; similar with ESTF_MYZPE
Px017454.1	-1.2946	1.98E-32	3.32E-31	Liver carboxylesterase; similar with EST1_MESAU
Px005902.4	-1.2680	1.53E-55	3.64E-54	Cytochrome P450 6B6; similar with CP6B6_HELAM
Px000089.1	-1.2537	7.16E-31	1.16E-29	Acetylcholinesterase; similar with ACES_FELCA
Px001164.1	-1.2470	1.19E-129	5.15E-128	UDP-glucuronosyltransferase; similar with UGT3_PLEPL
Px010678.1	-1.1851	7.84E-05	3.39E-04	Ecdysteroid UDP-glucosyltransferase; similar with UDPE_NPVSL
Px017253.1	-1.1579	3.91E-33	6.66E-32	Cytochrome P450 6B2; similar with CP6B2_HELAM
Px001162.1	-1.1273	1.39E-70	3.89E-69	UDP-glucuronosyltransferase 2B1; similar with UD2B1_RAT
Px013202.1	-1.0671	1.48E-27	2.20E-26	UDP-glucuronosyltransferase 2C1; similar with UD2C1_RABIT
Px017996.1	-1.0647	8.55E-47	1.83E-45	Acetylcholinesterase; similar with ACES_HUMAN
Px002723.1	-1.0254	5.27E-169	2.82E-167	Cytochrome P450 6B7; similar with CP6B7_HELAM

Px003352.1	-1.0082	2.58E-07	1.55E-06	Venom carboxylesterase-6; similar with EST6_APIME

Involed in drug metabolism - cytochrome P450

Px015080.1	1.5382	2.00E-23	2.70E-22	Ecdysteroid UDP-glucosyltransferase; similar with UDPE_NPVMB
Px013145.1	1.0351	2.01E-231	1.45E-229	Cytochrome P450 6B7; similar with CP6B7_HELAM
Px015079.1	-3.1595	1.17E-07	7.28E-07	Ecdysteroid UDP-glucosyltransferase; similar with UDPE_NPVSL
Px004854.1	-2.7504	3.14E-20	3.86E-19	Ecdysteroid UDP-glucosyltransferase; similar with UDPE_NPVSL
Px011987.1	-2.3364	2.05E-16	2.21E-15	Amine oxidase [flavin-containing] B; similar with AOFB_MOUSE
Px014217.1	-1.9896	7.35E-20	8.93E-19	Cytochrome P450 6k1; similar with CP6K1_BLAGE
Px000872.2	-1.9896	8.91E-10	6.55E-09	Ecdysteroid UDP-glucosyltransferase; similar with UDPE_NPVSL
Px005900.1	-1.9164	8.05E-36	1.43E-34	Cytochrome P450 6B2; similar with CP6B2_HELAM
Px015078.1	-1.6900	8.07E-07	4.58E-06	Ecdysteroid UDP-glucosyltransferase; similar with UDPE_NPVSL
Px014216.1	-1.5409	2.47E-29	3.84E-28	Cytochrome P450 6B6; similar with CP6B6_HELAM
Px013958.1	-1.5363	1.75E-141	8.12E-140	Cytochrome P450 6k1; similar with CP6K1_BLAGE
Px000790.1	-1.5164	2.95E-29	4.57E-28	Glutathione S-transferase 1; similar with GSTT1_MANSE
Px006292.1	-1.3115	1.53E-13	1.46E-12	Ecdysteroid UDP-glucosyltransferase; similar with UDPE_NPVSL
Px005902.4	-1.2680	1.53E-55	3.64E-54	Cytochrome P450 6B6; similar with CP6B6_HELAM
Px006106.1	-1.2511	1.12E-164	5.90E-163	Glutathione S-transferase 1; similar with GSTT1_MANSE
Px001164.1	-1.2470	1.19E-129	5.15E-128	UDP-glucuronosyltransferase; similar with UGT3_PLEPL
Px010678.1	-1.1851	7.84E-05	3.39E-04	Ecdysteroid UDP-glucosyltransferase; similar with UDPE_NPVSL
Px017253.1	-1.1579	3.91E-33	6.66E-32	Cytochrome P450 6B2; similar with CP6B2_HELAM
Px001162.1	-1.1273	1.39E-70	3.89E-69	UDP-glucuronosyltransferase 2B1; similar with UD2B1_RAT
Px013202.1	-1.0671	1.48E-27	2.20E-26	UDP-glucuronosyltransferase 2C1; similar with UD2C1_RABIT
Px002723.1	-1.0254	5.27E-169	2.82E-167	Cytochrome P450 6B7; similar with CP6B7_HELAM

Involved incomplement and coagulation cascades

Px005668.1	2.7474	1.53E-07	9.40E-07	NGFI-A-binding protein homolog; similar with NAB_DROME
Px006739.1	1.8778	0.00E+00	0.00E+00	Putative trypsin Inhibitor like cysteine rich domain protein
Px013792.1	1.1458	6.31E-33	1.07E-31	Thrombospondin type-1 domain-containing protein 7A
Px012678.4	1.1408	3.74E-05	1.71E-04	Locomotion-related protein Hikaru genki; similar with HIG_DROME
Px003000.1	#######	1.59E-40	3.09E-39	Transmembrane protease serine 6; similar with TMPS6_MOUSE
Px015905.1	-4.2248	3.15E-52	7.24E-51	Transmembrane protease serine 6; similar with TMPS6_MOUSE
Px004167.1	-2.5442	5.53E-08	3.53E-07	Plasma kallikrein; similar with KLKB1_MOUSE
Px016549.2	-2.4814	1.62E-07	9.92E-07	Glia-derived nexin; similar with GDN_MOUSE
Px016053.1	-2.2883	3.96E-32	6.55E-31	Trypsin; similar with TRYP_PHACE
Px005947.1	-1.8827	5.51E-09	3.84E-08	Serine proteinase stubble; similar with STUB_DROME
Px004168.1	-1.4433	1.50E-05	7.32E-05	Plasma kallikrein; similar with KLKB1_HUMAN
Px007357.2	-1.3810	0.00E+00	0.00E+00	Serine proteinase stubble; similar with STUB_DROME
Px007757.1	-1.3200	2.18E-11	1.81E-10	Putative trypsin Inhibitor like cysteine rich domain protein
Px006864.1	-1.0865	1.30E-14	1.30E-13	Serine protease snake; similar with SNAK_DROME

Involved in lysosome

Px007889.1	1.9173	7.13E-06	3.66E-05	V-type proton ATPase 116 kDa subunit a isoform 1
Px007446.1	1.3698	1.05E-25	1.50E-24	Alpha-N-acetylgalactosaminidase; similar with NAGAB_CHICK
Px003790.1	1.1669	0.00E+00	0.00E+00	Testicular acid phosphatase homolog; similar with PPAT_XENLA
Px007749.1	1.1513	1.29E-09	9.37E-09	Unknown function
Px006262.1	1.0468	2.05E-19	2.46E-18	Lipase 3; similar with LIP3_DROME
Px006315.2	1.0426	1.25E-21	1.59E-20	Lysosomal alpha-glucosidase; similar with LYAG_MOUSE
Px010775.1	-3.0540	6.34E-76	1.89E-74	Putative inorganic phosphate cotransporter
Px009691.1	-2.4128	3.95E-17	4.40E-16	Lipase 1; similar with LIP1_DROME

Px001913.1	-1.9424	2.00E-107	7.50E-106	Gastric triacylglycerol lipase; similar with LIPG_RAT
Px013680.1	-1.8855	4.80E-30	7.62E-29	Sialin; similar with S17A5_HUMAN
Px012695.1	-1.8366	2.01E-82	6.33E-81	Lipase 1; similar with LIP1_DROME
Px005804.1	-1.8351	1.46E-67	3.90E-66	Lipase 1; similar with LIP1_DROME
Px004426.1	-1.8092	9.99E-104	3.65E-102	Niemann-Pick C1 protein; similar with NPC1_MOUSE
Px013556.1	-1.7544	0.00E+00	0.00E+00	Flexible cuticle protein 12; similar with CU12_HYACE
Px003742.1	-1.6686	1.74E-27	2.57E-26	Sphingomyelin phosphodiesterase; similar with ASM_HUMAN
Px005805.1	-1.6287	1.50E-16	1.63E-15	Lipase 1; similar with LIP1_DROME
Px008080.1	-1.5900	1.25E-08	8.47E-08	Lysosome membrane protein 2; similar with SCRB2_HUMAN
Px013679.1	-1.5509	2.16E-10	1.69E-09	Putative inorganic phosphate cotransporter
Px012829.1	-1.4307	7.16E-96	2.47E-94	Tetraspanin-17; similar with TSN17_RAT
Px004605.1	-1.2838	2.12E-41	4.20E-40	Sphingomyelin phosphodiesterase; similar with ASM_HUMAN
Px016066.1	-1.2734	1.11E-10	8.80E-10	Lipase member M; similar with LIPM_MOUSE
Px013678.1	-1.2380	4.66E-21	5.85E-20	Putative inorganic phosphate cotransporter
Px004125.2	-1.1955	2.64E-120	1.09E-118	Sulfatase-modifying factor 1; similar with SUMF1_MOUSE
Px002047.1	-1.1417	3.74E-76	1.12E-74	Uncharacterized family 31 glucosidase KIAA1161
Px011477.2	-1.1127	3.00E-99	1.07E-97	Lipase 1; similar with LIP1_DROME
Px001079.1	-1.1085	0.00E+00	0.00E+00	Unknown function
Px011475.1	-1.0965	2.90E-126	1.23E-124	Lipase 3; similar with LIP3_DROME
Px011111.1	-1.0868	1.63E-31	2.67E-30	Ecdysteroid-regulated protein; similar with B6A8L2_HELAM

Involved in metabolic pathways

Px009455.1	4.0104	1.36E-04	5.61E-04	Uridylate kinase; similar with UMPK_ARATH
Px006540.1	2.3796	2.18E-05	1.04E-04	ACYPI009600 protein; similar with C4WT25_ACYPI
Px003101.1	2.0104	6.13E-07	3.53E-06	Tyramine beta-hydroxylase; similar with TBH1_DROME
Px013955.1	1.9862	1.32E-07	8.12E-07	Neuroligin-1; similar with NLGN1_HUMAN
Px006993.1	1.9490	4.43E-06	2.32E-05	Polypeptide N-acetylgalactosaminyltransferase 1
Px007889.1	1.9173	7.13E-06	3.66E-05	V-type proton ATPase 116 kDa subunit a isoform 1
Px007720.1	1.7166	5.06E-56	1.21E-54	Catalase; similar with CATA_GLARU
Px015393.1	1.7108	7.79E-06	3.96E-05	Gag-like protein; similar with Q9BPP9_BOMMO
Px015148.1	1.6885	2.11E-05	1.01E-04	ATP synthase subunit beta, mitochondrial; similar with ATPB_RAT
Px009818.1	1.6813	1.48E-158	7.58E-157	Putative aldehyde oxidase-like protein; similar with ALDOL_ORYSJ
Px016786.1	1.6161	5.93E-07	3.42E-06	Peroxidase; similar with PERO_DROME
Px004229.1	1.5931	1.10E-50	2.49E-49	Retrovirus-related Pol polyprotein LINE-1
Px007722.1	1.5425	3.70E-39	6.99E-38	Catalase; similar with CATA_PONAB
Px015080.1	1.5382	2.00E-23	2.70E-22	Ecdysteroid UDP-glucosyltransferase; similar with UDPE_NPVMB
Px012983.1	1.5250	9.55E-06	4.79E-05	Folylpolyglutamate synthase, mitochondrial
Px007028.1	1.5228	0.00E+00	0.00E+00	Arylphorin subunit alpha; similar with ARYA_MANSE
Px012332.1	1.4063	6.88E-21	8.59E-20	ATP synthase subunit beta, mitochondrial
Px009277.1	1.3870	8.25E-50	1.84E-48	Probable aconitate hydratase, mitochondrial
Px001209.1	1.3865	0.00E+00	0.00E+00	Catalase; similar with CATA_PIG
Px013482.1	1.3839	6.02E-05	2.65E-04	Exostosin-1; similar with EXT1_DROME
Px002634.1	1.3665	2.50E-05	1.18E-04	Beta-1,4-N-acetylgalactosaminyltransferase bre-4
Px014748.1	1.3175	1.52E-91	5.11E-90	Unknown function
Px011844.1	1.3123	8.80E-10	6.47E-09	Adenylate kinase 7; similar with KAD7_MOUSE
Px002394.3	1.3100	2.66E-90	8.90E-89	D-beta-hydroxybutyrate dehydrogenase, mitochondrial
Px010889.1	1.2841	3.14E-12	2.76E-11	LINE-1 reverse transcriptase homolog; similar with LIN1_NYCCO
Px012442.1	1.2592	3.07E-66	8.07E-65	Peroxidase; similar with PERO_DROME
Px018009.1	1.2456	1.71E-07	1.04E-06	Cytosol aminopeptidase; similar with AMPL_HUMAN
Px007568.1	1.2449	1.13E-05	5.63E-05	Sesquipedalian-1; similar with SESQ1_MOUSE

Px005927.1	1.1989	2.34E-06	1.27E-05	Beta-1,3-galactosyltransferase 5; similar with B3GT5_MOUSE
Px010267.1	1.1863	2.29E-04	9.08E-04	Inositol polyphosphate 1-phosphatase; similar with INPP_MOUSE
Px013191.1	1.1200	6.80E-09	4.69E-08	Aromatic-L-amino-acid decarboxylase; similar with DDC_RAT
Px000615.1	1.0950	2.04E-34	3.56E-33	Retinol dehydrogenase 11; similar with RDH11_HUMAN
Px011830.5	1.0868	1.41E-05	6.90E-05	DNA polymerase epsilon catalytic subunit A
Px012229.1	1.0709	2.01E-04	8.06E-04	Probable UDP-glucose 4-epimerase; similar with GALE_DROME
Px009343.1	1.0610	3.15E-06	1.68E-05	NADH-ubiquinone oxidoreductase 75 kDa subunit, mitochondrial
Px006262.1	1.0468	2.05E-19	2.46E-18	Lipase 3; similar with LIP3_DROME
Px006315.2	1.0426	1.25E-21	1.59E-20	Lysosomal alpha-glucosidase; similar with LYAG_MOUSE
Px013145.1	1.0351	2.01E-231	1.45E-229	Cytochrome P450 6B7; similar with CP6B7_HELAM
Px013390.1	1.0237	1.15E-05	5.72E-05	NADH dehydrogenase [ubiquinone] iron-sulfur protein 3, mitochondrial
Px002247.1	1.0224	1.35E-34	2.37E-33	ADP-dependent glucokinase; similar with ADPGK_MOUSE
Px010954.1	1.0211	1.09E-06	6.08E-06	Probable DNA-directed RNA polymerases I and III subunit RPAC2
Px014253.1	#######	3.43E-14	3.37E-13	Unknown function
Px000337.1	-5.6413	6.62E-147	3.17E-145	Osiris 19; similar with B6DXA8_BOMMO
Px000336.1	-5.1595	2.16E-92	7.30E-91	Osiris 20; similar with B6DXA7_BOMMO
Px014975.1	-4.7969	6.48E-08	4.10E-07	Alcohol dehydrogenase [NADP+] A; similar with A1A1A_DANRE
Px005494.1	-4.6043	1.32E-59	3.26E-58	Tubulin polyglutamylase TTLL6; similar with TTLL6_MOUSE
Px010126.1	-4.2842	5.01E-37	9.15E-36	DNA-directed RNA polymerase II subunit RPB1
Px000024.1	-4.2375	2.27E-05	1.07E-04	Cuticular protein CPG4; similar with B2DBL4_9NEOP
Px010831.1	-3.6714	6.10E-17	6.72E-16	Luciferin 4-monooxygenase; similar with LUCI_PHOPE
Px016492.1	-3.3845	4.82E-55	1.14E-53	Luciferin 4-monooxygenase; similar with LUCI_LUCCR
Px003251.1	-3.2604	5.86E-42	1.17E-40	Putative cuticle protein; similar with C0H6E8_BOMMO
Px015079.1	-3.1595	1.17E-07	7.28E-07	Ecdysteroid UDP-glucosyltransferase; similar with UDPE_NPVSL
Px016235.1	-3.0771	3.82E-10	2.90E-09	3-oxoacyl-[acyl-carrier-protein] reductase FabG
Px007180.1	-2.9490	1.26E-24	1.76E-23	Juvenile hormone esterase; similar with ESTJ_HELVI
Px014796.1	-2.7969	1.80E-13	1.70E-12	Apyrase; similar with APY_AEDAE
Px004854.1	-2.7504	3.14E-20	3.86E-19	Ecdysteroid UDP-glucosyltransferase; similar with UDPE_NPVSL
Px008866.1	-2.7311	8.72E-18	9.93E-17	Chorion peroxidase; similar with PERC_DROME
Px013527.1	-2.6900	3.73E-05	1.71E-04	Carbonyl reductase [NADPH] 1; similar with CBR1_RABIT
Px018069.2	-2.6785	1.96E-29	3.05E-28	3-ketoacyl-CoA thiolase, mitochondrial; similar with THIM_HUMAN
Px011757.1	-2.5132	1.72E-33	2.95E-32	Esterase FE4; similar with ESTF_MYZPE
Px002199.1	-2.4921	5.49E-06	2.86E-05	4-coumarate--CoA ligase-like 5; similar with 4CLL5_ARATH
Px009691.1	-2.4128	3.95E-17	4.40E-16	Lipase 1; similar with LIP1_DROME
Px006617.2	-2.3588	1.63E-21	2.07E-20	Chorion peroxidase; similar with PERC_DROME
Px011987.1	-2.3364	2.05E-16	2.21E-15	Amine oxidase [flavin-containing] B; similar with AOFB_MOUSE
Px004490.1	-2.2872	1.08E-212	7.20E-211	Adenosine deaminase CECR1; similar with CECR1_XENLA
Px002274.1	-2.2838	8.00E-307	7.21E-305	Phenoloxidase subunit 2; similar with PRP2_BOMMO
Px003155.2	-2.2612	3.08E-22	4.00E-21	Retrovirus-related Pol polyprotein from transposon 17.6
Px009159.1	-2.2120	2.35E-06	1.27E-05	Alpha-aminoadipic semialdehyde synthase, mitochondrial
Px008598.1	-2.2120	4.51E-08	2.90E-07	Phosphatidylinositol glycan anchor biosynthesis class U protein
Px011550.1	-2.1807	1.46E-17	1.65E-16	Glycerate kinase; similar with GLCTK_RAT
Px003540.1	-2.1707	6.03E-47	1.29E-45	Hexokinase type 2; similar with HXK2_DROME
Px016518.1	-2.0402	1.22E-07	7.55E-07	Ribulose-phosphate 3-epimerase; similar with RPE_MOUSE
Px015642.1	-2.0171	5.29E-07	3.08E-06	Estradiol 17-beta-dehydrogenase 8; similar with DHB8_CANFA
Px014217.1	-1.9896	7.35E-20	8.93E-19	Cytochrome P450 6k1; similar with CP6K1_BLAGE
Px000872.2	-1.9896	8.91E-10	6.55E-09	Ecdysteroid UDP-glucosyltransferase; similar with UDPE_NPVSL
Px012011.2	-1.9715	3.54E-204	2.27E-202	Pancreatic triacylglycerol lipase; similar with LIPP_MYOCO
Px011686.1	-1.9569	3.07E-28	4.64E-27	5-formyltetrahydrofolate cyclo-ligase
Px008276.1	-1.9483	1.70E-213	1.14E-211	Membrane alanyl aminopeptidase; similar with AMPM_HELVI
Px001913.1	-1.9424	2.00E-107	7.50E-106	Gastric triacylglycerol lipase; similar with LIPG_RAT

Px003486.1	-1.9201	1.08E-287	9.35E-286	Probable maltase H; similar with MAL2_DROME
Px005900.1	-1.9164	8.05E-36	1.43E-34	Cytochrome P450 6B2; similar with CP6B2_HELAM
Px000359.2	-1.8693	2.82E-10	2.17E-09	Alpha-methyldopa hypersensitive protein
Px001371.1	-1.8641	1.23E-06	6.87E-06	Aminoacylase-1A; similar with ACY1A_RAT
Px008278.1	-1.8417	1.19E-27	1.77E-26	Membrane alanyl aminopeptidase; similar with AMPM_MANSE
Px012695.1	-1.8366	2.01E-82	6.33E-81	Lipase 1; similar with LIP1_DROME
Px005804.1	-1.8351	1.46E-67	3.90E-66	Lipase 1; similar with LIP1_DROME
Px011049.1	-1.8032	1.05E-22	1.38E-21	Acetylcholinesterase; similar with ACES_FELCA
Px004610.1	-1.7646	2.93E-50	6.58E-49	Sphingosine-1-phosphate lyase; similar with SGPL_DROME
Px016080.1	-1.7601	1.66E-06	9.14E-06	Glucosamine-6-phosphate isomerase; similar with GNPI_AEDAE
Px009077.7	-1.7541	1.06E-244	8.14E-243	ATP-citrate synthase; similar with ACLY_BOVIN
Px011755.1	-1.7175	1.14E-37	2.10E-36	Antennal esterase CXE4; similar with D3GDL5_SPOLI
Px002187.1	-1.7145	1.31E-11	1.11E-10	Alpha-aminoadipic semialdehyde synthase, mitochondrial
Px002926.1	-1.6997	7.49E-30	1.18E-28	Choline dehydrogenase, mitochondrial; similar with CHDH_HUMAN
Px015078.1	-1.6900	8.07E-07	4.58E-06	Ecdysteroid UDP-glucosyltransferase; similar with UDPE_NPVSL
Px017452.1	-1.6900	1.43E-04	5.92E-04	Putative uncharacterized protein; similar with D6WQR2_TRICA
Px000123.1	-1.6773	1.05E-300	9.40E-299	Putative aldehyde dehydrogenase family 7 member A1 homolog
Px009007.1	-1.6741	4.83E-05	2.16E-04	4-coumarate--CoA ligase-like 5; similar with 4CLL5_ARATH
Px003742.1	-1.6686	1.74E-27	2.57E-26	Sphingomyelin phosphodiesterase; similar with ASM_HUMAN
Px005805.1	-1.6287	1.50E-16	1.63E-15	Lipase 1; similar with LIP1_DROME
Px013509.3	-1.6243	4.97E-06	2.60E-05	Phosphatidylinositol-4-phosphate 5-kinase type-1 gamma
Px014078.1	-1.6118	5.34E-26	7.67E-25	Fructose-bisphosphate aldolase; similar with ALF_DROME
Px000763.1	-1.5860	3.76E-11	3.08E-10	Acetyl-CoA carboxylase; similar with ACAC_CHICK
Px016709.1	-1.5845	1.40E-23	1.90E-22	Glucosamine-6-phosphate isomerase; similar with GNPI_AEDAE
Px016655.1	-1.5797	5.94E-23	7.89E-22	Luciferin 4-monooxygenase; similar with LUCI_LUCCR
Px013169.1	-1.5746	8.44E-13	7.70E-12	Lactase-phlorizin hydrolase; similar with LPH_HUMAN
Px016493.1	-1.5635	1.34E-30	2.15E-29	Luciferin 4-monooxygenase; similar with LUCI_LUCMI
Px002140.1	-1.5608	5.78E-156	2.90E-154	Alpha-aminoadipic semialdehyde synthase, mitochondrial
Px000084.1	-1.5608	5.40E-17	5.96E-16	Cysteine sulfinic acid decarboxylase; similar with CSAD_HUMAN
Px015569.1	-1.5545	1.85E-38	3.45E-37	3-oxoacyl-[acyl-carrier-protein] reductase FabG
Px014216.1	-1.5409	2.47E-29	3.84E-28	Cytochrome P450 6B6; similar with CP6B6_HELAM
Px013958.1	-1.5363	1.75E-141	8.12E-140	Cytochrome P450 6k1; similar with CP6K1_BLAGE
Px011916.1	-1.4712	3.87E-17	4.31E-16	3-hydroxyisobutyryl-CoA hydrolase, mitochondrial
Px003205.1	-1.4688	1.09E-07	6.74E-07	Envelope fusion protein; similar with ENV_NPVLD
Px005799.4	-1.4526	2.86E-08	1.87E-07	Probable 4-coumarate--CoA ligase 3; similar with 4CL3_ORYSJ
Px008322.1	-1.4354	1.34E-06	7.40E-06	Aminoacylase-1A; similar with ACY1A_RAT
Px004354.1	-1.4211	4.63E-28	6.96E-27	UDP-N-acetylhexosamine pyrophosphorylase-like protein 1
Px009815.1	-1.4145	5.08E-245	3.93E-243	Pyrroline-5-carboxylate reductase; similar with P5CR_SOYBN
Px015448.1	-1.4118	1.12E-05	5.59E-05	Acetyl-CoA carboxylase; similar with ACAC_CHICK
Px002027.1	-1.4107	1.31E-11	1.10E-10	L-sorbose 1-dehydrogenase; similar with SDH_GLUOX
Px012806.2	-1.4027	4.47E-17	4.96E-16	Luciferin 4-monooxygenase; similar with LUCI_LUCCR
Px003013.3	-1.3887	5.16E-201	3.25E-199	Lipoyltransferase 1, mitochondrial; similar with LIPT_MOUSE
Px014887.1	-1.3881	2.57E-59	6.32E-58	Dihydropteridine reductase; similar with DHPR_RAT
Px013730.1	-1.3842	3.22E-26	4.64E-25	Myrosinase 1; similar with MYRO1_BREBR
Px005277.3	-1.3778	0.00E+00	0.00E+00	Lactase-phlorizin hydrolase; similar with LPH_HUMAN
Px002736.1	-1.3736	3.57E-192	2.18E-190	Venom carboxylesterase-6; similar with EST6_APIME
Px007493.1	-1.3725	1.02E-157	5.15E-156	Delta-1-pyrroline-5-carboxylate synthase; similar with P5CS_PONAB
Px015449.1	-1.3608	1.13E-08	7.71E-08	Acetyl-CoA carboxylase 1; similar with ACACA_SHEEP
Px002115.1	-1.3555	1.38E-38	2.58E-37	5-formyltetrahydrofolate cyclo-ligase; similar with MTHFS_HUMAN
Px012400.1	-1.3371	1.19E-21	1.52E-20	Argininosuccinate lyase; similar with ARLY2_ANAPL
Px004065.1	-1.3335	3.00E-07	1.79E-06	Mannosyl-oligosaccharide alpha-1,2-mannosidase isoform A

Px011713.1	-1.3121	1.24E-67	3.32E-66	Fatty acid synthase; similar with FAS_HUMAN
Px006292.1	-1.3115	1.53E-13	1.46E-12	Ecdysteroid UDP-glucosyltransferase; similar with UDPE_NPVSL
Px011707.1	-1.3115	3.98E-38	7.39E-37	Amidophosphoribosyltransferase; similar with PUR1_DROME
Px000825.3	-1.3000	8.64E-37	1.57E-35	Ferrochelatase, mitochondrial; similar with HEMH_DROME
Px011984.1	-1.2997	6.98E-94	2.38E-92	Ribose-phosphate pyrophosphokinase 1; similar with PRPS1_RAT
Px002735.1	-1.2993	2.03E-97	7.10E-96	Esterase FE4; similar with ESTF_MYZPE
Px002859.2	-1.2971	3.12E-99	1.11E-97	Bifunctional purine biosynthesis protein PURH
Px004605.1	-1.2838	2.12E-41	4.20E-40	Sphingomyelin phosphodiesterase; similar with ASM_HUMAN
Px014232.1	-1.2808	1.50E-06	8.27E-06	Pantothenate kinase 2, mitochondrial; similar with PANK2_HUMAN
Px012794.1	-1.2769	2.19E-09	1.57E-08	Methylcrotonoyl-CoA carboxylase subunit alpha, mitochondrial
Px016066.1	-1.2734	1.11E-10	8.80E-10	Lipase member M; similar with LIPM_MOUSE
Px011885.3	-1.2703	2.13E-76	6.43E-75	Bifunctional purine biosynthesis protein PURH
Px005902.4	-1.2680	1.53E-55	3.64E-54	Cytochrome P450 6B6; similar with CP6B6_HELAM
Px012677.1	-1.2571	7.62E-05	3.30E-04	Lipase member H-A; similar with LIPHA_XENLA
Px000765.5	-1.2565	1.62E-21	2.05E-20	Acetyl-CoA carboxylase; similar with ACAC_CHICK
Px000089.1	-1.2537	7.16E-31	1.16E-29	Acetylcholinesterase; similar with ACES_FELCA
Px001164.1	-1.2470	1.19E-129	5.15E-128	UDP-glucuronosyltransferase; similar with UGT3_PLEPL
Px013937.1	-1.2420	1.18E-05	5.88E-05	3-ketoacyl-CoA thiolase, mitochondrial
Px011476.1	-1.2320	1.40E-11	1.18E-10	Lipase 3; similar with LIP3_DROME
Px012800.1	-1.2320	1.03E-26	1.49E-25	Pyruvate carboxylase 1; similar with PYC1_CAEEL
Px004749.1	-1.2238	0.00E+00	0.00E+00	Lipoprotein lipase; similar with LIPL_CAVPO
Px006190.1	-1.2201	0.00E+00	0.00E+00	Lipase member H-A; similar with LIPHA_XENLA
Px016488.1	-1.2191	7.78E-05	3.36E-04	Glucose dehydrogenase [acceptor]; similar with DHGL_DROME
Px004753.1	-1.2160	2.50E-80	7.79E-79	Pancreatic lipase-related protein 2; similar with LIPR2_MYOCO
Px004980.1	-1.2120	1.54E-04	6.33E-04	UMP-CMP kinase; similar with KCY_XENTR
Px010260.3	-1.2101	1.05E-14	1.06E-13	Alcohol dehydrogenase [NADP+] A; similar with A1A1A_DANRE
Px009776.1	-1.2078	3.55E-07	2.10E-06	Mevalonate kinase; similar with KIME_DICDI
Px000764.1	-1.2030	2.00E-49	4.42E-48	Acetyl-CoA carboxylase; similar with ACAC_CHICK
Px007878.1	-1.1969	2.78E-39	5.29E-38	3-hydroxyacyl-CoA dehydrogenase type-2
Px010678.1	-1.1851	7.84E-05	3.39E-04	Ecdysteroid UDP-glucosyltransferase; similar with UDPE_NPVSL
Px011035.1	-1.1760	5.18E-201	3.25E-199	Ribose-phosphate pyrophosphokinase 2
Px005884.1	-1.1728	4.40E-98	1.54E-96	Transaldolase; similar with TALDO_CRIGR
Px003616.1	-1.1710	4.18E-134	1.87E-132	Transaldolase; similar with TALDO_RAT
Px007863.4	-1.1641	3.18E-109	1.21E-107	6-phosphogluconate dehydrogenase, decarboxylating
Px017253.1	-1.1579	3.91E-33	6.66E-32	Cytochrome P450 6B2; similar with CP6B2_HELAM
Px006345.1	-1.1527	6.76E-112	2.60E-110	Pancreatic lipase-related protein 2; similar with LIPR2_MYOCO
Px016079.1	-1.1519	8.64E-06	4.36E-05	Glucosamine-6-phosphate isomerase; similar with GNPI_DROPS
Px016756.1	-1.1480	3.98E-13	3.68E-12	Cytoplasmic aconitate hydratase; similar with ACOC_HUMAN
Px010556.1	-1.1463	0.00E+00	0.00E+00	Probable phosphoserine aminotransferase
Px009940.2	-1.1454	2.86E-13	2.67E-12	Acetylcholinesterase; similar with ACES_CULPI
Px002047.1	-1.1417	3.74E-76	1.12E-74	Uncharacterized family 31 glucosidase KIAA1161
Px016733.1	-1.1416	6.72E-08	4.25E-07	Acyl-CoA synthetase family member 3, mitochondrial
Px009630.1	-1.1394	9.09E-62	2.30E-60	Fatty acid synthase; similar with FAS_CHICK
Px016278.1	-1.1364	4.19E-07	2.46E-06	Cytoplasmic aconitate hydratase; similar with ACOC_MOUSE
Px011480.1	-1.1316	3.44E-123	1.44E-121	Purine nucleoside phosphorylase; similar with PNPH_BOVIN
Px010509.1	-1.1311	3.30E-11	2.72E-10	Glycerate kinase; similar with GLCTK_RAT
Px001162.1	-1.1273	1.39E-70	3.89E-69	UDP-glucuronosyltransferase 2B1; similar with UD2B1_RAT
Px002046.1	-1.1174	7.12E-244	5.40E-242	Uncharacterized family 31 glucosidase KIAA1161
Px012455.1	-1.1171	7.44E-116	2.97E-114	NADP-dependent malic enzyme; similar with MAOX_ANAPL
Px005203.1	-1.1159	3.84E-31	6.22E-30	Glucose-6-phosphate 1-dehydrogenase; similar with G6PD_CERCA
Px011477.2	-1.1127	3.00E-99	1.07E-97	Lipase 1; similar with LIP1_DROME

Px011791.1	-1.1123	1.16E-291	1.01E-289	Lipase member H; similar with LIPH_XENTR
Px013315.1	-1.1099	5.18E-256	4.15E-254	Short/branched chain specific acyl-CoA dehydrogenase, mitochondrial
Px010660.1	-1.1027	7.41E-19	8.78E-18	Probable general secretion pathway protein D
Px006668.1	-1.0988	1.89E-26	2.75E-25	Phosphoglucomutase-2; similar with PGM2_HUMAN
Px011475.1	-1.0965	2.90E-126	1.23E-124	Lipase 3; similar with LIP3_DROME
Px011636.4	-1.0964	1.91E-96	6.62E-95	Phosphoribosylformylglycinamidine synthase
Px006816.1	-1.0920	0.00E+00	0.00E+00	2-oxoisovalerate dehydrogenase subunit alpha, mitochondrial
Px001933.1	-1.0839	3.76E-08	2.44E-07	Myrosinase 1; similar with MYRO1_BREBR
Px000532.1	-1.0750	2.79E-31	4.54E-30	Hydroxyacid oxidase 1; similar with HAOX1_HUMAN
Px013202.1	-1.0671	1.48E-27	2.20E-26	UDP-glucuronosyltransferase 2C1; similar with UD2C1_RABIT
Px000644.1	-1.0662	3.37E-06	1.79E-05	Pancreatic triacylglycerol lipase; similar with LIPP_MOUSE
Px000372.1	-1.0658	1.11E-08	7.57E-08	Putative gamma-glutamyltransferase ywrD
Px017996.1	-1.0647	8.55E-47	1.83E-45	Acetylcholinesterase; similar with ACES_HUMAN
Px006468.1	-1.0636	1.21E-04	5.05E-04	Adenine phosphoribosyltransferase; similar with APT_DROME
Px016731.1	-1.0617	5.94E-15	6.04E-14	Acyl-CoA synthetase family member 3, mitochondrial
Px000395.1	-1.0609	0.00E+00	0.00E+00	Alpha-amylase 4N; similar with AM4N_DROAN
Px006270.1	-1.0573	7.72E-06	3.93E-05	Luciferin 4-monooxygenase; similar with LUCI_PHOPE
Px011788.2	-1.0547	1.82E-55	4.32E-54	Pancreatic lipase-related protein 2; similar with LIPR2_BOVIN
Px009411.3	-1.0508	1.83E-82	5.77E-81	Trifunctional purine biosynthetic protein adenosine-3
Px014429.1	-1.0489	1.94E-24	2.71E-23	Acetyl-CoA carboxylase; similar with ACAC_CHICK
Px008848.1	-1.0387	1.30E-48	2.85E-47	Myrosinase 1; similar with MYRO1_BREBR
Px006643.3	-1.0372	3.20E-28	4.84E-27	Luciferin 4-monooxygenase; similar with LUCI_LUCMI
Px006720.1	-1.0369	4.84E-05	2.17E-04	Glycerol-3-phosphate acyltransferase 4
Px012275.1	-1.0310	2.02E-41	4.00E-40	Glucosamine--fructose-6-phosphate aminotransferase [isomerizing] 1
Px002723.1	-1.0254	5.27E-169	2.82E-167	Cytochrome P450 6B7; similar with CP6B7_HELAM
Px011708.1	-1.0187	1.78E-24	2.49E-23	Amidophosphoribosyltransferase; similar with PUR1_DROME
Px002029.1	-1.0155	2.06E-48	4.48E-47	Oxidoreductase ucpA; similar with UCPA_SALTY
Px012993.1	-1.0097	3.06E-69	8.45E-68	Multifunctional protein ADE2; similar with PUR6_DROME
Px003352.1	-1.0082	2.58E-07	1.55E-06	Venom carboxylesterase-6; similar with EST6_APIME
Px011202.1	-1.0066	3.64E-27	5.35E-26	Aromatic-L-amino-acid decarboxylase; similar with DDC_MANSE

Involved in glycerolipid metabolism

Px007446.1	1.3698	1.05E-25	1.50E-24	Alpha-N-acetylgalactosaminidase; similar with NAGAB_CHICK
Px006262.1	1.0468	2.05E-19	2.46E-18	Lipase 3; similar with LIP3_DROME
Px014975.1	-4.7969	6.48E-08	4.10E-07	Alcohol dehydrogenase [NADP+] A; similar with A1A1A_DANRE
Px007180.1	-2.9490	1.26E-24	1.76E-23	Juvenile hormone esterase; similar with ESTJ_HELVI
Px011757.1	-2.5132	1.72E-33	2.95E-32	Esterase FE4; similar with ESTF_MYZPE
Px009691.1	-2.4128	3.95E-17	4.40E-16	Lipase 1; similar with LIP1_DROME
Px011550.1	-2.1807	1.46E-17	1.65E-16	Glycerate kinase; similar with GLCTK_RAT
Px012011.2	-1.9715	3.54E-204	2.27E-202	Pancreatic triacylglycerol lipase; similar with LIPP_MYOCO
Px001913.1	-1.9424	2.00E-107	7.50E-106	Gastric triacylglycerol lipase; similar with LIPG_RAT
Px012695.1	-1.8366	2.01E-82	6.33E-81	Lipase 1; similar with LIP1_DROME
Px005804.1	-1.8351	1.46E-67	3.90E-66	Lipase 1; similar with LIP1_DROME
Px011049.1	-1.8032	1.05E-22	1.38E-21	Acetylcholinesterase; similar with ACES_FELCA
Px011755.1	-1.7175	1.14E-37	2.10E-36	Antennal esterase CXE4; similar with D3GDL5_SPOLI
Px000123.1	-1.6773	1.05E-300	9.40E-299	Putative aldehyde dehydrogenase family 7 member A1 homolog
Px005805.1	-1.6287	1.50E-16	1.63E-15	Lipase 1; similar with LIP1_DROME
Px002736.1	-1.3736	3.57E-192	2.18E-190	Venom carboxylesterase-6; similar with EST6_APIME
Px002735.1	-1.2993	2.03E-97	7.10E-96	Esterase FE4; similar with ESTF_MYZPE
Px016066.1	-1.2734	1.11E-10	8.80E-10	Lipase member M; similar with LIPM_MOUSE
Px012677.1	-1.2571	7.62E-05	3.30E-04	Lipase member H-A; similar with LIPHA_XENLA

Px000089.1	-1.2537	7.16E-31	1.16E-29	Acetylcholinesterase; similar with ACES_FELCA
Px011476.1	-1.2320	1.40E-11	1.18E-10	Lipase 3; similar with LIP3_DROME
Px004749.1	-1.2238	0.00E+00	0.00E+00	Lipoprotein lipase; similar with LIPL_CAVPO
Px006190.1	-1.2201	0.00E+00	0.00E+00	Lipase member H-A; similar with LIPHA_XENLA
Px004753.1	-1.2160	2.50E-80	7.79E-79	Pancreatic lipase-related protein 2; similar with LIPR2_MYOCO
Px010260.3	-1.2101	1.05E-14	1.06E-13	Alcohol dehydrogenase [NADP+] A; similar with A1A1A_DANRE
Px006345.1	-1.1527	6.76E-112	2.60E-110	Pancreatic lipase-related protein 2; similar with LIPR2_MYOCO
Px009940.2	-1.1454	2.86E-13	2.67E-12	Acetylcholinesterase; similar with ACES_CULPI
Px010509.1	-1.1311	3.30E-11	2.72E-10	Glycerate kinase; similar with GLCTK_RAT
Px011477.2	-1.1127	3.00E-99	1.07E-97	Lipase 1; similar with LIP1_DROME
Px011791.1	-1.1123	1.16E-291	1.01E-289	Lipase member H; similar with LIPH_XENTR
Px011475.1	-1.0965	2.90E-126	1.23E-124	Lipase 3; similar with LIP3_DROME
Px000644.1	-1.0662	3.37E-06	1.79E-05	Pancreatic triacylglycerol lipase; similar with LIPP_MOUSE
Px017996.1	-1.0647	8.55E-47	1.83E-45	Acetylcholinesterase; similar with ACES_HUMAN
Px011788.2	-1.0547	1.82E-55	4.32E-54	Pancreatic lipase-related protein 2; similar with LIPR2_BOVIN
Px006720.1	-1.0369	4.84E-05	2.17E-04	Glycerol-3-phosphate acyltransferase 4; similar with GPAT4_PONAB
Px003352.1	-1.0082	2.58E-07	1.55E-06	Venom carboxylesterase-6; similar with EST6_APIME

Involved in starch and sucrose metabolism

Px015080.1	1.5382	2.00E-23	2.70E-22	Ecdysteroid UDP-glucosyltransferase; similar with UDPE_NPVMB
Px006315.2	1.0426	1.25E-21	1.59E-20	Lysosomal alpha-glucosidase; similar with LYAG_MOUSE
Px015079.1	-3.1595	1.17E-07	7.28E-07	Ecdysteroid UDP-glucosyltransferase; similar with UDPE_NPVSL
Px004854.1	-2.7504	3.14E-20	3.86E-19	Ecdysteroid UDP-glucosyltransferase; similar with UDPE_NPVSL
Px003540.1	-2.1707	6.03E-47	1.29E-45	Hexokinase type 2; similar with HXK2_DROME
Px000872.2	-1.9896	8.91E-10	6.55E-09	Ecdysteroid UDP-glucosyltransferase; similar with UDPE_NPVSL
Px003486.1	-1.9201	1.08E-287	9.35E-286	Probable maltase H; similar with MAL2_DROME
Px015078.1	-1.6900	8.07E-07	4.58E-06	Ecdysteroid UDP-glucosyltransferase; similar with UDPE_NPVSL
Px003205.1	-1.4688	1.09E-07	6.74E-07	Envelope fusion protein; similar with ENV_NPVLD
Px004726.1	-1.3861	1.31E-17	1.48E-16	Alpha,alpha-trehalose-phosphate synthase [UDP-forming] 56 kDa subunit
Px006292.1	-1.3115	1.53E-13	1.46E-12	Ecdysteroid UDP-glucosyltransferase; similar with UDPE_NPVSL
Px001164.1	-1.2470	1.19E-129	5.15E-128	UDP-glucuronosyltransferase; similar with UGT3_PLEPL
Px002118.1	-1.2076	4.48E-160	2.30E-158	Alpha,alpha-trehalose-phosphate synthase [UDP-forming] A
Px010678.1	-1.1851	7.84E-05	3.39E-04	Ecdysteroid UDP-glucosyltransferase; similar with UDPE_NPVSL
Px002047.1	-1.1417	3.74E-76	1.12E-74	Uncharacterized family 31 glucosidase KIAA1161
Px001162.1	-1.1273	1.39E-70	3.89E-69	UDP-glucuronosyltransferase 2B1; similar with UD2B1_RAT
Px002046.1	-1.1174	7.12E-244	5.40E-242	Uncharacterized family 31 glucosidase KIAA1161
Px006668.1	-1.0988	1.89E-26	2.75E-25	Phosphoglucomutase-2; similar with PGM2_HUMAN
Px013202.1	-1.0671	1.48E-27	2.20E-26	UDP-glucuronosyltransferase 2C1; similar with UD2C1_RABIT
Px000395.1	-1.0609	0.00E+00	0.00E+00	Alpha-amylase 4N; similar with AM4N_DROAN

Involved infFat digestion and absorption

Px006262.1	1.0468	2.05E-19	2.46E-18	Lipase 3; similar with LIP3_DROME
Px007180.1	-2.9490	1.26E-24	1.76E-23	Juvenile hormone esterase; similar with ESTJ_HELVI
Px011757.1	-2.5132	1.72E-33	2.95E-32	Esterase FE4; similar with ESTF_MYZPE
Px009691.1	-2.4128	3.95E-17	4.40E-16	Lipase 1; similar with LIP1_DROME
Px012011.2	-1.9715	3.54E-204	2.27E-202	Pancreatic triacylglycerol lipase; similar with LIPP_MYOCO
Px012164.1	-1.8535	4.44E-10	3.35E-09	Protein croquemort; similar with CRQ_DROME
Px011049.1	-1.8032	1.05E-22	1.38E-21	Acetylcholinesterase; similar with ACES_FELCA
Px011755.1	-1.7175	1.14E-37	2.10E-36	Antennal esterase CXE4; similar with D3GDL5_SPOLI
Px005805.1	-1.6287	1.50E-16	1.63E-15	Lipase 1; similar with LIP1_DROME
Px008080.1	-1.5900	1.25E-08	8.47E-08	Lysosome membrane protein 2; similar with SCRB2_HUMAN

Px002736.1	-1.3736	3.57E-192	2.18E-190	Venom carboxylesterase-6; similar with EST6_APIME
Px002735.1	-1.2993	2.03E-97	7.10E-96	Esterase FE4; similar with ESTF_MYZPE
Px012677.1	-1.2571	7.62E-05	3.30E-04	Lipase member H-A; similar with LIPHA_XENLA
Px000089.1	-1.2537	7.16E-31	1.16E-29	Acetylcholinesterase; similar with ACES_FELCA
Px004270.2	-1.2482	1.17E-18	1.37E-17	Microsomal triglyceride transfer protein large subunit
Px006190.1	-1.2201	0.00E+00	0.00E+00	Lipase member H-A; similar with LIPHA_XENLA
Px004753.1	-1.2160	2.50E-80	7.79E-79	Pancreatic lipase-related protein 2; similar with LIPR2_MYOCO
Px006345.1	-1.1527	6.76E-112	2.60E-110	Pancreatic lipase-related protein 2; similar with LIPR2_MYOCO
Px009940.2	-1.1454	2.86E-13	2.67E-12	Acetylcholinesterase; similar with ACES_CULPI
Px016733.1	-1.1416	6.72E-08	4.25E-07	Acyl-CoA synthetase family member 3, mitochondrial
Px011791.1	-1.1123	1.16E-291	1.01E-289	Lipase member H; similar with LIPH_XENTR
Px000644.1	-1.0662	3.37E-06	1.79E-05	Pancreatic triacylglycerol lipase; similar with LIPP_MOUSE
Px017996.1	-1.0647	8.55E-47	1.83E-45	Acetylcholinesterase; similar with ACES_HUMAN
Px011788.2	-1.0547	1.82E-55	4.32E-54	Pancreatic lipase-related protein 2; similar with LIPR2_BOVIN
Px001510.4	-1.0333	6.73E-37	1.23E-35	Scavenger receptor class B member 1; similar with SCRB1_PIG
Px011529.1	-1.0272	4.23E-66	1.11E-64	Apolipophorins; similar with APLP_LOCMI
Px008150.1	-1.0244	6.04E-08	3.84E-07	Scavenger receptor class B member 1; similar with SCRB1_PIG
Px012163.2	-1.0220	6.81E-12	5.85E-11	Protein croquemort; similar with CRQ_DROME
Px003352.1	-1.0082	2.58E-07	1.55E-06	Venom carboxylesterase-6; similar with EST6_APIME

Involved in metabolism of xenobiotics by cytochrome P450

Px015080.1	1.5382	2.00E-23	2.70E-22	Ecdysteroid UDP-glucosyltransferase; similar with UDPE_NPVMB
Px013145.1	1.0351	2.01E-231	1.45E-229	Cytochrome P450 6B7; similar with CP6B7_HELAM
Px015079.1	-3.1595	1.17E-07	7.28E-07	Ecdysteroid UDP-glucosyltransferase; similar with UDPE_NPVSL
Px004854.1	-2.7504	3.14E-20	3.86E-19	Ecdysteroid UDP-glucosyltransferase; similar with UDPE_NPVSL
Px013527.1	-2.6900	3.73E-05	1.71E-04	Carbonyl reductase [NADPH] 1; similar with CBR1_RABIT
Px014217.1	-1.9896	7.35E-20	8.93E-19	Cytochrome P450 6k1; similar with CP6K1_BLAGE
Px000872.2	-1.9896	8.91E-10	6.55E-09	Ecdysteroid UDP-glucosyltransferase; similar with UDPE_NPVSL
Px005900.1	-1.9164	8.05E-36	1.43E-34	Cytochrome P450 6B2; similar with CP6B2_HELAM
Px015078.1	-1.6900	8.07E-07	4.58E-06	Ecdysteroid UDP-glucosyltransferase; similar with UDPE_NPVSL
Px014216.1	-1.5409	2.47E-29	3.84E-28	Cytochrome P450 6B6; similar with CP6B6_HELAM
Px013958.1	-1.5363	1.75E-141	8.12E-140	Cytochrome P450 6k1; similar with CP6K1_BLAGE
Px000790.1	-1.5164	2.95E-29	4.57E-28	Glutathione S-transferase 1; similar with GSTT1_MANSE
Px006292.1	-1.3115	1.53E-13	1.46E-12	Ecdysteroid UDP-glucosyltransferase; similar with UDPE_NPVSL
Px005902.4	-1.2680	1.53E-55	3.64E-54	Cytochrome P450 6B6; similar with CP6B6_HELAM
Px006106.1	-1.2511	1.12E-164	5.90E-163	Glutathione S-transferase 1; similar with GSTT1_MANSE
Px001164.1	-1.2470	1.19E-129	5.15E-128	UDP-glucuronosyltransferase; similar with UGT3_PLEPL
Px010678.1	-1.1851	7.84E-05	3.39E-04	Ecdysteroid UDP-glucosyltransferase; similar with UDPE_NPVSL
Px017253.1	-1.1579	3.91E-33	6.66E-32	Cytochrome P450 6B2; similar with CP6B2_HELAM
Px001162.1	-1.1273	1.39E-70	3.89E-69	UDP-glucuronosyltransferase 2B1; similar with UD2B1_RAT
Px013202.1	-1.0671	1.48E-27	2.20E-26	UDP-glucuronosyltransferase 2C1; similar with UD2C1_RABIT
Px002723.1	-1.0254	5.27E-169	2.82E-167	Cytochrome P450 6B7; similar with CP6B7_HELAM

Involved in linoleic acid metabolism

Px000615.1	1.0950	2.04E-34	3.56E-33	Retinol dehydrogenase 11; similar with RDH11_HUMAN
Px013145.1	1.0351	2.01E-231	1.45E-229	Cytochrome P450 6B7; similar with CP6B7_HELAM
Px014217.1	-1.9896	7.35E-20	8.93E-19	Cytochrome P450 6k1; similar with CP6K1_BLAGE
Px005900.1	-1.9164	8.05E-36	1.43E-34	Cytochrome P450 6B2; similar with CP6B2_HELAM
Px014216.1	-1.5409	2.47E-29	3.84E-28	Cytochrome P450 6B6; similar with CP6B6_HELAM
Px013958.1	-1.5363	1.75E-141	8.12E-140	Cytochrome P450 6k1; similar with CP6K1_BLAGE
Px005902.4	-1.2680	1.53E-55	3.64E-54	Cytochrome P450 6B6; similar with CP6B6_HELAM

Px017253.1	-1.1579	3.91E-33	6.66E-32	Cytochrome P450 6B2; similar with CP6B2_HELAM
Px002723.1	-1.0254	5.27E-169	2.82E-167	Cytochrome P450 6B7; similar with CP6B7_HELAM

Involved in PPAR signaling pathway

Px015380.1	-3.1169	1.43E-65	3.73E-64	Acyl-CoA Delta(11) desaturase; similar with ACO11_TRINI
Px000524.1	-2.3593	6.86E-128	2.93E-126	Putative acyl-CoA-binding protein; similar with ACBP_HYPDU
Px017039.1	-1.7904	0.00E+00	0.00E+00	Putative acyl-CoA-binding protein; similar with ACBP_HYPDU
Px015467.1	-1.6138	4.30E-71	1.21E-69	Acyl-CoA Delta(11) desaturase; similar with ACO11_TRINI
Px012916.1	-1.6002	1.24E-101	4.50E-100	Fatty acid-binding protein 1; similar with FABP1_MANSE
Px008080.1	-1.5900	1.25E-08	8.47E-08	Lysosome membrane protein 2; similar with SCRB2_HUMAN
Px001605.1	-1.5492	7.75E-228	5.50E-226	Putative acyl-CoA-binding protein; similar with ACBP_HYPDU
Px002136.1	-1.3265	3.32E-66	8.72E-65	Acyl-CoA Delta(11) desaturase; similar with ACO11_TRINI
Px013837.1	-1.2989	1.47E-04	6.04E-04	Carnitine O-palmitoyltransferase 1, muscle isoform
Px012677.1	-1.2571	7.62E-05	3.30E-04	Lipase member H-A; similar with LIPHA_XENLA
Px004749.1	-1.2238	0.00E+00	0.00E+00	Lipoprotein lipase; similar with LIPL_CAVPO
Px004753.1	-1.2160	2.50E-80	7.79E-79	Pancreatic lipase-related protein 2; similar with LIPR2_MYOCO
Px016733.1	-1.1416	6.72E-08	4.25E-07	Acyl-CoA synthetase family member 3, mitochondrial
Px012455.1	-1.1171	7.44E-116	2.97E-114	NADP-dependent malic enzyme; similar with MAOX_ANAPL

Involved in phenylalanine metabolism

Px016786.1	1.6161	5.93E-07	3.42E-06	Peroxidase; similar with PERO_DROME
Px013191.1	1.1200	6.80E-09	4.69E-08	Aromatic-L-amino-acid decarboxylase; similar with DDC_RAT
Px010831.1	-3.6714	6.10E-17	6.72E-16	Luciferin 4-monooxygenase; similar with LUCI_PHOPE
Px016492.1	-3.3845	4.82E-55	1.14E-53	Luciferin 4-monooxygenase; similar with LUCI_LUCCR
Px002199.1	-2.4921	5.49E-06	2.86E-05	4-coumarate--CoA ligase-like 5; similar with 4CLL5_ARATH
Px011987.1	-2.3364	2.05E-16	2.21E-15	Amine oxidase [flavin-containing] B; similar with AOFB_MOUSE
Px005763.1	-2.1051	3.50E-10	2.66E-09	Fatty-acid amide hydrolase 2; similar with FAAH2_HUMAN
Px000359.2	-1.8693	2.82E-10	2.17E-09	Alpha-methyldopa hypersensitive protein
Px009007.1	-1.6741	4.83E-05	2.16E-04	4-coumarate--CoA ligase-like 5; similar with 4CLL5_ARATH
Px016655.1	-1.5797	5.94E-23	7.89E-22	Luciferin 4-monooxygenase; similar with LUCI_LUCCR
Px016493.1	-1.5635	1.34E-30	2.15E-29	Luciferin 4-monooxygenase; similar with LUCI_LUCMI
Px005799.4	-1.4526	2.86E-08	1.87E-07	Probable 4-coumarate--CoA ligase 3; similar with 4CL3_ORYSJ
Px012806.2	-1.4027	4.47E-17	4.96E-16	Luciferin 4-monooxygenase; similar with LUCI_LUCCR
Px006643.3	-1.0372	3.20E-28	4.84E-27	Luciferin 4-monooxygenase; similar with LUCI_LUCMI
Px011202.1	-1.0066	3.64E-27	5.35E-26	Aromatic-L-amino-acid decarboxylase; similar with DDC_MANSE

Involved in retinol metabolism

Px015080.1	1.5382	2.00E-23	2.70E-22	Ecdysteroid UDP-glucosyltransferase; similar with UDPE_NPVMB
Px000615.1	1.0950	2.04E-34	3.56E-33	Retinol dehydrogenase 11; similar with RDH11_HUMAN
Px013145.1	1.0351	2.01E-231	1.45E-229	Cytochrome P450 6B7; similar with CP6B7_HELAM
Px015079.1	-3.1595	1.17E-07	7.28E-07	Ecdysteroid UDP-glucosyltransferase; similar with UDPE_NPVSL
Px004854.1	-2.7504	3.14E-20	3.86E-19	Ecdysteroid UDP-glucosyltransferase; similar with UDPE_NPVSL
Px014217.1	-1.9896	7.35E-20	8.93E-19	Cytochrome P450 6k1; similar with CP6K1_BLAGE
Px000872.2	-1.9896	8.91E-10	6.55E-09	Ecdysteroid UDP-glucosyltransferase; similar with UDPE_NPVSL
Px005900.1	-1.9164	8.05E-36	1.43E-34	Cytochrome P450 6B2; similar with CP6B2_HELAM
Px015078.1	-1.6900	8.07E-07	4.58E-06	Ecdysteroid UDP-glucosyltransferase; similar with UDPE_NPVSL
Px015569.1	-1.5545	1.85E-38	3.45E-37	3-oxoacyl-[acyl-carrier-protein] reductase FabG
Px014216.1	-1.5409	2.47E-29	3.84E-28	Cytochrome P450 6B6; similar with CP6B6_HELAM
Px013958.1	-1.5363	1.75E-141	8.12E-140	Cytochrome P450 6k1; similar with CP6K1_BLAGE
Px006292.1	-1.3115	1.53E-13	1.46E-12	Ecdysteroid UDP-glucosyltransferase; similar with UDPE_NPVSL
Px005902.4	-1.2680	1.53E-55	3.64E-54	Cytochrome P450 6B6; similar with CP6B6_HELAM

Px001164.1	-1.2470	1.19E-129	5.15E-128	UDP-glucuronosyltransferase; similar with UGT3_PLEPL
Px010678.1	-1.1851	7.84E-05	3.39E-04	Ecdysteroid UDP-glucosyltransferase; similar with UDPE_NPVSL
Px017253.1	-1.1579	3.91E-33	6.66E-32	Cytochrome P450 6B2; similar with CP6B2_HELAM
Px001162.1	-1.1273	1.39E-70	3.89E-69	UDP-glucuronosyltransferase 2B1; similar with UD2B1_RAT
Px013202.1	-1.0671	1.48E-27	2.20E-26	UDP-glucuronosyltransferase 2C1; similar with UD2C1_RABIT
Px002723.1	-1.0254	5.27E-169	2.82E-167	Cytochrome P450 6B7; similar with CP6B7_HELAM
Px002029.1	-1.0155	2.06E-48	4.48E-47	Oxidoreductase ucpA; similar with UCPA_SALTY

Involved in tyrosine metabolism

Px003101.1	2.0104	6.13E-07	3.53E-06	Tyramine beta-hydroxylase; similar with TBH1_DROME
Px007028.1	1.5228	0.00E+00	0.00E+00	Arylphorin subunit alpha; similar with ARYA_MANSE
Px012442.1	1.2592	3.07E-66	8.07E-65	Peroxidase; similar with PERO_DROME
Px013191.1	1.1200	6.80E-09	4.69E-08	Aromatic-L-amino-acid decarboxylase; similar with DDC_RAT
Px011809.2	-3.7266	1.89E-09	1.36E-08	Nose resistant to fluoxetine protein 6; similar with NRF6_CAEEL
Px008866.1	-2.7311	8.72E-18	9.93E-17	Chorion peroxidase; similar with PERC_DROME
Px015388.1	-2.4814	1.62E-07	9.92E-07	Nose resistant to fluoxetine protein 6; similar with NRF6_CAEEL
Px006617.2	-2.3588	1.63E-21	2.07E-20	Chorion peroxidase; similar with PERC_DROME
Px011987.1	-2.3364	2.05E-16	2.21E-15	Amine oxidase [flavin-containing] B; similar with AOFB_MOUSE
Px002274.1	-2.2838	8.00E-307	7.21E-305	Phenoloxidase subunit 2; similar with PRP2_BOMMO
Px005418.1	-2.1595	1.73E-05	8.37E-05	Nose resistant to fluoxetine protein 6; similar with NRF6_CAEEL
Px005417.1	-2.1099	1.03E-12	9.36E-12	Nose resistant to fluoxetine protein 6; similar with NRF6_CAEEL
Px016277.1	-1.9071	1.18E-04	4.95E-04	Nose resistant to fluoxetine protein 6; similar with NRF6_CAEEL
Px000359.2	-1.8693	2.82E-10	2.17E-09	Alpha-methyldopa hypersensitive protein
Px003528.3	-1.6441	2.99E-08	1.95E-07	Nose resistant to fluoxetine protein 6; similar with NRF6_CAEEL
Px011624.1	-1.3721	7.16E-06	3.67E-05	Nose resistant to fluoxetine protein 6; similar with NRF6_CAEEL
Px011202.1	-1.0066	3.64E-27	5.35E-26	Aromatic-L-amino-acid decarboxylase; similar with DDC_MANSE

Involved in amino sugar and nucleotide sugar metabolism

Px017244.1	1.8055	1.16E-71	3.29E-70	N-acetylneuraminate lyase; similar with NPL_BOVIN
Px010131.1	1.1276	2.57E-34	4.46E-33	Peritrophin 1; similar with Q86BV0_9NEOP
Px012229.1	1.0709	2.01E-04	8.06E-04	Probable UDP-glucose 4-epimerase; similar with GALE_DROME
Px008062.1	-4.4584	1.02E-145	4.81E-144	Endochitinase; similar with CHIT_MANSE
Px006322.1	-3.4490	2.12E-05	1.01E-04	Phosphoacetylglucosamine mutase; similar with AGM1_HUMAN
Px017503.2	-2.4325	4.27E-13	3.95E-12	Probable chitinase 2; similar with CHIT2_DROME
Px003540.1	-2.1707	6.03E-47	1.29E-45	Hexokinase type 2; similar with HXK2_DROME
Px009279.1	-1.8850	5.69E-71	1.60E-69	Chitotriosidase-1; similar with CHIT1_HUMAN
Px016080.1	-1.7601	1.66E-06	9.14E-06	Glucosamine-6-phosphate isomerase; similar with GNPI_AEDAE
Px016709.1	-1.5845	1.40E-23	1.90E-22	Glucosamine-6-phosphate isomerase; similar with GNPI_AEDAE
Px004354.1	-1.4211	4.63E-28	6.96E-27	UDP-N-acetylhexosamine pyrophosphorylase-like protein 1
Px005972.1	-1.3849	6.30E-214	4.27E-212	N-acetylneuraminate lyase; similar with NPL_RAT
Px015646.1	-1.3432	2.76E-08	1.81E-07	Chondroitin proteoglycan-2; similar with CPG2_CAEEL
Px012913.1	-1.3137	2.57E-98	9.06E-97	Chitin synthase 6; similar with CHS6_USTMA
Px014300.1	-1.2104	1.75E-16	1.89E-15	Chitin deacetylase 4; similar with A8W489_TRICA
Px016079.1	-1.1519	8.64E-06	4.36E-05	Glucosamine-6-phosphate isomerase; similar with GNPI_DROPS
Px006668.1	-1.0988	1.89E-26	2.75E-25	Phosphoglucomutase-2; similar with PGM2_HUMAN
Px012275.1	-1.0310	2.02E-41	4.00E-40	Glucosamine--fructose-6-phosphate aminotransferase [isomerizing] 1

Involved in ascorbate and aldarate metabolism

Px015080.1	1.5382	2.00E-23	2.70E-22	Ecdysteroid UDP-glucosyltransferase; similar with UDPE_NPVMB
Px015079.1	-3.1595	1.17E-07	7.28E-07	Ecdysteroid UDP-glucosyltransferase; similar with UDPE_NPVSL
Px004854.1	-2.7504	3.14E-20	3.86E-19	Ecdysteroid UDP-glucosyltransferase; similar with UDPE_NPVSL

Px000872.2	-1.9896	8.91E-10	6.55E-09	Ecdysteroid UDP-glucosyltransferase; similar with UDPE_NPVSL
Px015078.1	-1.6900	8.07E-07	4.58E-06	Ecdysteroid UDP-glucosyltransferase; similar with UDPE_NPVSL
Px000123.1	-1.6773	1.05E-300	9.40E-299	Putative aldehyde dehydrogenase family 7 member A1 homolog
Px006292.1	-1.3115	1.53E-13	1.46E-12	Ecdysteroid UDP-glucosyltransferase; similar with UDPE_NPVSL
Px001164.1	-1.2470	1.19E-129	5.15E-128	UDP-glucuronosyltransferase; similar with UGT3_PLEPL
Px010678.1	-1.1851	7.84E-05	3.39E-04	Ecdysteroid UDP-glucosyltransferase; similar with UDPE_NPVSL
Px010576.1	-1.1539	2.05E-22	2.68E-21	Inositol oxygenase; similar with MIOX_DANRE
Px001162.1	-1.1273	1.39E-70	3.89E-69	UDP-glucuronosyltransferase 2B1; similar with UD2B1_RAT
Px013202.1	-1.0671	1.48E-27	2.20E-26	UDP-glucuronosyltransferase 2C1; similar with UD2C1_RABIT

Involved in porphyrin and chlorophyll metabolism

Px015080.1	1.5382	2.00E-23	2.70E-22	Ecdysteroid UDP-glucosyltransferase; similar with UDPE_NPVMB
Px015079.1	-3.1595	1.17E-07	7.28E-07	Ecdysteroid UDP-glucosyltransferase; similar with UDPE_NPVSL
Px004854.1	-2.7504	3.14E-20	3.86E-19	Ecdysteroid UDP-glucosyltransferase; similar with UDPE_NPVSL
Px000872.2	-1.9896	8.91E-10	6.55E-09	Ecdysteroid UDP-glucosyltransferase; similar with UDPE_NPVSL
Px015078.1	-1.6900	8.07E-07	4.58E-06	Ecdysteroid UDP-glucosyltransferase; similar with UDPE_NPVSL
Px006292.1	-1.3115	1.53E-13	1.46E-12	Ecdysteroid UDP-glucosyltransferase; similar with UDPE_NPVSL
Px000825.3	-1.3000	8.64E-37	1.57E-35	Ferrochelatase, mitochondrial; similar with HEMH_DROME
Px001164.1	-1.2470	1.19E-129	5.15E-128	UDP-glucuronosyltransferase; similar with UGT3_PLEPL
Px010678.1	-1.1851	7.84E-05	3.39E-04	Ecdysteroid UDP-glucosyltransferase; similar with UDPE_NPVSL
Px012540.1	-1.1556	5.07E-36	9.05E-35	Heme oxygenase 1; similar with HMOX1_HUMAN
Px001162.1	-1.1273	1.39E-70	3.89E-69	UDP-glucuronosyltransferase 2B1; similar with UD2B1_RAT
Px013202.1	-1.0671	1.48E-27	2.20E-26	UDP-glucuronosyltransferase 2C1; similar with UD2C1_RABIT

Involved in glycerophospholipid metabolism

Px006262.1	1.0468	2.05E-19	2.46E-18	Lipase 3; similar with LIP3_DROME
Px011809.2	-3.7266	1.89E-09	1.36E-08	Nose resistant to fluoxetine protein 6; similar with NRF6_CAEEL
Px015388.1	-2.4814	1.62E-07	9.92E-07	Nose resistant to fluoxetine protein 6; similar with NRF6_CAEEL
Px009691.1	-2.4128	3.95E-17	4.40E-16	Lipase 1; similar with LIP1_DROME
Px005418.1	-2.1595	1.73E-05	8.37E-05	Nose resistant to fluoxetine protein 6; similar with NRF6_CAEEL
Px005417.1	-2.1099	1.03E-12	9.36E-12	Nose resistant to fluoxetine protein 6; similar with NRF6_CAEEL
Px001913.1	-1.9424	2.00E-107	7.50E-106	Gastric triacylglycerol lipase; similar with LIPG_RAT
Px016277.1	-1.9071	1.18E-04	4.95E-04	Nose resistant to fluoxetine protein 6; similar with NRF6_CAEEL
Px012695.1	-1.8366	2.01E-82	6.33E-81	Lipase 1; similar with LIP1_DROME
Px005804.1	-1.8351	1.46E-67	3.90E-66	Lipase 1; similar with LIP1_DROME
Px011049.1	-1.8032	1.05E-22	1.38E-21	Acetylcholinesterase; similar with ACES_FELCA
Px003528.3	-1.6441	2.99E-08	1.95E-07	Nose resistant to fluoxetine protein 6; similar with NRF6_CAEEL
Px005805.1	-1.6287	1.50E-16	1.63E-15	Lipase 1; similar with LIP1_DROME
Px011624.1	-1.3721	7.16E-06	3.67E-05	Nose resistant to fluoxetine protein 6; similar with NRF6_CAEEL
Px017454.1	-1.2946	1.98E-32	3.32E-31	Liver carboxylesterase; similar with EST1_MESAU
Px016066.1	-1.2734	1.11E-10	8.80E-10	Lipase member M; similar with LIPM_MOUSE
Px011476.1	-1.2320	1.40E-11	1.18E-10	Lipase 3; similar with LIP3_DROME
Px004749.1	-1.2238	0.00E+00	0.00E+00	Lipoprotein lipase; similar with LIPL_CAVPO
Px004753.1	-1.2160	2.50E-80	7.79E-79	Pancreatic lipase-related protein 2; similar with LIPR2_MYOCO
Px006345.1	-1.1527	6.76E-112	2.60E-110	Pancreatic lipase-related protein 2; similar with LIPR2_MYOCO
Px009940.2	-1.1454	2.86E-13	2.67E-12	Acetylcholinesterase; similar with ACES_CULPI
Px011477.2	-1.1127	3.00E-99	1.07E-97	Lipase 1; similar with LIP1_DROME
Px011475.1	-1.0965	2.90E-126	1.23E-124	Lipase 3; similar with LIP3_DROME
Px011788.2	-1.0547	1.82E-55	4.32E-54	Pancreatic lipase-related protein 2; similar with LIPR2_BOVIN
Px006720.1	-1.0369	4.84E-05	2.17E-04	Glycerol-3-phosphate acyltransferase 4; similar with GPAT4_PONAB

Involved in pyruvate metabolism

Px008696.1	-5.3497	5.87E-297	5.20E-295	Osiris 9; similar with B6DXB0_BOMMO
Px014975.1	-4.7969	6.48E-08	4.10E-07	Alcohol dehydrogenase [NADP+] A; similar with A1A1A_DANRE
Px008692.2	-4.4151	0.00E+00	0.00E+00	Osiris 9; similar with B6DXB0_BOMMO
Px008693.1	-3.2626	2.45E-91	8.23E-90	Osiris 9; similar with B6DXB0_BOMMO
Px008691.1	-2.9941	0.00E+00	0.00E+00	Osiris 9; similar with B6DXB0_BOMMO
Px000123.1	-1.6773	1.05E-300	9.40E-299	Putative aldehyde dehydrogenase family 7 member A1 homolog
Px000763.1	-1.5860	3.76E-11	3.08E-10	Acetyl-CoA carboxylase; similar with ACAC_CHICK
Px015448.1	-1.4118	1.12E-05	5.59E-05	Acetyl-CoA carboxylase; similar with ACAC_CHICK
Px015449.1	-1.3608	1.13E-08	7.71E-08	Acetyl-CoA carboxylase 1; similar with ACACA_SHEEP
Px000765.5	-1.2565	1.62E-21	2.05E-20	Acetyl-CoA carboxylase; similar with ACAC_CHICK
Px012800.1	-1.2320	1.03E-26	1.49E-25	Pyruvate carboxylase 1; similar with PYC1_CAEEL
Px010260.3	-1.2101	1.05E-14	1.06E-13	Alcohol dehydrogenase [NADP+] A; similar with A1A1A_DANRE
Px000764.1	-1.2030	2.00E-49	4.42E-48	Acetyl-CoA carboxylase; similar with ACAC_CHICK
Px012455.1	-1.1171	7.44E-116	2.97E-114	NADP-dependent malic enzyme; similar with MAOX_ANAPL
Px016731.1	-1.0617	5.94E-15	6.04E-14	Acyl-CoA synthetase family member 3, mitochondrial
Px014429.1	-1.0489	1.94E-24	2.71E-23	Acetyl-CoA carboxylase; similar with ACAC_CHICK

Involved in galactose metabolism

Px007446.1	1.3698	1.05E-25	1.50E-24	Alpha-N-acetylgalactosaminidase; similar with NAGAB_CHICK
Px002634.1	1.3665	2.50E-05	1.18E-04	Beta-1,4-N-acetylgalactosaminyltransferase bre-4
Px012229.1	1.0709	2.01E-04	8.06E-04	Probable UDP-glucose 4-epimerase; similar with GALE_DROME
Px006315.2	1.0426	1.25E-21	1.59E-20	Lysosomal alpha-glucosidase; similar with LYAG_MOUSE
Px014975.1	-4.7969	6.48E-08	4.10E-07	Alcohol dehydrogenase [NADP+] A; similar with A1A1A_DANRE
Px003540.1	-2.1707	6.03E-47	1.29E-45	Hexokinase type 2; similar with HXK2_DROME
Px003486.1	-1.9201	1.08E-287	9.35E-286	Probable maltase H; similar with MAL2_DROME
Px013169.1	-1.5746	8.44E-13	7.70E-12	Lactase-phlorizin hydrolase; similar with LPH_HUMAN
Px003205.1	-1.4688	1.09E-07	6.74E-07	Envelope fusion protein; similar with ENV_NPVLD
Px013730.1	-1.3842	3.22E-26	4.64E-25	Myrosinase 1; similar with MYRO1_BREBR
Px005277.3	-1.3778	0.00E+00	0.00E+00	Lactase-phlorizin hydrolase; similar with LPH_HUMAN
Px010260.3	-1.2101	1.05E-14	1.06E-13	Alcohol dehydrogenase [NADP+] A; similar with A1A1A_DANRE
Px002047.1	-1.1417	3.74E-76	1.12E-74	Uncharacterized family 31 glucosidase KIAA1161
Px002046.1	-1.1174	7.12E-244	5.40E-242	Uncharacterized family 31 glucosidase KIAA1161
Px006668.1	-1.0988	1.89E-26	2.75E-25	Phosphoglucomutase-2; similar with PGM2_HUMAN
Px001933.1	-1.0839	3.76E-08	2.44E-07	Myrosinase 1; similar with MYRO1_BREBR
Px008848.1	-1.0387	1.30E-48	2.85E-47	Myrosinase 1; similar with MYRO1_BREBR

Involved in alpha-Linolenic acid metabolism

Px006262.1	1.0468	2.05E-19	2.46E-18	Lipase 3; similar with LIP3_DROME
Px009691.1	-2.4128	3.95E-17	4.40E-16	Lipase 1; similar with LIP1_DROME
Px001913.1	-1.9424	2.00E-107	7.50E-106	Gastric triacylglycerol lipase; similar with LIPG_RAT
Px012695.1	-1.8366	2.01E-82	6.33E-81	Lipase 1; similar with LIP1_DROME
Px005804.1	-1.8351	1.46E-67	3.90E-66	Lipase 1; similar with LIP1_DROME
Px005805.1	-1.6287	1.50E-16	1.63E-15	Lipase 1; similar with LIP1_DROME
Px016066.1	-1.2734	1.11E-10	8.80E-10	Lipase member M; similar with LIPM_MOUSE
Px011476.1	-1.2320	1.40E-11	1.18E-10	Lipase 3; similar with LIP3_DROME
Px004749.1	-1.2238	0.00E+00	0.00E+00	Lipoprotein lipase; similar with LIPL_CAVPO
Px004753.1	-1.2160	2.50E-80	7.79E-79	Pancreatic lipase-related protein 2; similar with LIPR2_MYOCO
Px006345.1	-1.1527	6.76E-112	2.60E-110	Pancreatic lipase-related protein 2; similar with LIPR2_MYOCO
Px011477.2	-1.1127	3.00E-99	1.07E-97	Lipase 1; similar with LIP1_DROME
Px011475.1	-1.0965	2.90E-126	1.23E-124	Lipase 3; similar with LIP3_DROME

Px011788.2	-1.0547	1.82E-55	4.32E-54	Pancreatic lipase-related protein 2; similar with LIPR2_BOVIN

Table G. Statistically enriched KEGG pathway for specific expressed genes.

Pathway ID	Description	Total genes	Specific genes	p-value
**Specific expressed in infected *P. xylostella***				
ko04745	Phototransduction - fly	66	9	1.81E-03
ko04080	Neuroactive ligand-receptor interaction	316	25	2.10E-03
ko04725	Cholinergic synapse	79	9	6.21E-03
ko04723	Retrograde endocannabinoid signaling	80	9	6.74E-03
ko04730	Long-term depression	69	8	8.70E-03
ko04540	Gap junction	71	8	1.03E-02
ko04972	Pancreatic secretion	341	24	1.08E-02
ko04916	Melanogenesis	119	11	1.24E-02
ko04130	SNARE interactions in vesicular transport	25	4	2.02E-02
ko04610	Complement and coagulation cascades	57	6	3.35E-02
ko04970	Salivary secretion	176	13	3.76E-02
ko04724	Glutamatergic synapse	107	9	3.85E-02
ko04962	Vasopressin-regulated water reabsorption	124	10	3.86E-02
ko04914	Progesterone-mediated oocyte maturation	126	10	4.24E-02
**Specific expressed in uninfected *P. xylostella***				
ko04640	Hematopoietic cell lineage	54	4	1.14E-02
ko00563	Glycosylphosphatidylinositol(GPI)-anchor biosynthesis	30	3	1.25E-02
ko00020	Citrate cycle (TCA cycle)	62	4	1.83E-02
ko04144	Endocytosis	237	8	4.01E-02
ko03015	mRNA surveillance pathway	329	10	4.27E-02
ko04916	Melanogenesis	119	5	4.49E-02
ko04726	Serotonergic synapse	84	4	4.81E-02
ko04666	Fc gamma R-mediated phagocytosis	162	6	4.92E-02

Table H. A list of DEGs involved in the detoxification of *P. xylostella* 24, 36 and 48 hpt_I versus hpt_C.

Gene ID	log_2 R 24 hpt_I/24 hpt_C	log_2 R 36 hpt_I/36 hpt_C	Log_2 R 48 hpt_I/48 hpt_C	Annotation
Px005901.1		1.0179		cytochrome P450
Px008537.1		1.3212		cytochrome P450
Px013454.1	-1.0045	2.0425		cytochrome P450
Px004867.1				cytochrome P450
Px013145.1				cytochrome P450
Px007339.1				cytochrome P450
Px009113.1				Glutathione S-transferase (GST)
Px010078.1		1.1245		GST
Px006481.1				GST
Px006106.1		1.3067		GST
Px000991.1	1.3435	3.2679	1.7324	Carboxylesterase (COE)
Px006430.1		2.4038		COE
Px009940.1		3.4221	1.4584	COE
Px002736.1		2.4868		COE
Px002735.1		2.6010		COE
Px011049.1		2.5090	1.5512	COE
Px011683.1		2.9306	2.4698	COE
Px011756.1		2.5480		COE
Px000089.1		2.6887		COE
Px005687.1		2.2408	1.1173	COE
Px000515.1		1.9774	1.3647	COE
Px011757.1	-1.1422	1.0401		(Antennal) COE
Px011755.1		1.7284		(Antennal) COE
Px001164.1		1.1390		UDP-glucosyltransferase (UGT)
Px000872.1		1.8756		UGT
Px015078.1	-1.2320			UGT
Px013203.1	1.3285	1.4517		UGT
Px004854.1	-1.2931	2.1011		UGT
Px014884.1				Aldehyde oxidase (AOX)
Px001209.1				Catalase (CAT)
Px005628.1			-2.5380	CAT
Px012442.1				Peroxidase (POD)
Px008505.1	-1.1688	1.7699	1.0866	Glucose oxidase (GOX)
Px004125.1		2.4130		Sulfatase-modifying factor (SUMF)
Px018104.1		3.5281	1.2702	Glucosinolate sulphatase (GSS)
Px018105.1		3.5788	2.0303	GSS
Px007687.1			1.7310	GSS
Px006776.1	-1.3711	2.0492		Sodium-independent sulfate anion transporter
Px004317.1		2.0159		Sodium-dependent phosphate transporter
Px004211.1		1.0195		Sodium-dependent phosphate transporter
Px013910.1				Organic anion transporter
Px008906.1		1.1025		Organic cation transporter

Table I. A list of DEGs enriched in the GO terms of *P. xylostella* at 24 hpt_I verus 24 hpt_C

Gene ID	log_2 R	P-value	FDR	Annotation
**GO:0005218**				
Px010453.1	-1.0959	3.64E-05	1.29E-04	Ryanodine receptor 44F; similar with RY44_DROME;
Px013237.1	-1.4617	5.34E-169	2.91E-167	Ryanodine receptor 44F; similar with RY44_DROME;
Px007169.1	-1.6039	1.11E-142	5.36E-141	Ryanodine receptor 44F; similar with RY44_DROME;
Px006829.9	-2.1487	1.74E-16	1.63E-15	Ryanodine receptor 44F; similar with RY44_DROME;
Px015884.1	-2.2297	3.22E-28	4.73E-27	Ryanodine receptor 44F; similar with RY44_DROME;
Px004335.2	-2.8349	1.43E-27	2.05E-26	Ryanodine receptor 44F; similar with RY44_DROME;
**GO:0005219**				
Px010453.1	-1.0959	3.64E-05	1.29E-04	Ryanodine receptor 44F; similar with RY44_DROME;
Px013237.1	-1.4617	5.34E-169	2.91E-167	Ryanodine receptor 44F; similar with RY44_DROME;
Px007169.1	-1.6039	1.11E-142	5.36E-141	Ryanodine receptor 44F; similar with RY44_DROME;
Px006829.9	-2.1487	1.74E-16	1.63E-15	Ryanodine receptor 44F; similar with RY44_DROME;
Px015884.1	-2.2297	3.22E-28	4.73E-27	Ryanodine receptor 44F; similar with RY44_DROME;
Px004335.2	-2.8349	1.43E-27	2.05E-26	Ryanodine receptor 44F; similar with RY44_DROME;
**GO:0015085**				
Px010453.1	-1.0959	3.64E-05	1.29E-04	Ryanodine receptor 44F;
Px014591.1	-1.0980	5.54E-10	3.30E-09	Inositol 1,4,5-trisphosphate receptor;
Px016332.1	-1.2199	0.00E+00	0.00E+00	Calcium-transporting ATPase sarcoplasmic/endoplasmic reticulum type;
Px004866.1	-1.2725	1.52E-09	8.72E-09	Inositol 1,4,5-trisphosphate receptor;
Px003569.1	-1.3529	1.56E-28	2.30E-27	Voltage-dependent calcium channel type D subunit alpha-1;
Px016654.1	-1.4183	1.81E-80	5.97E-79	Ryanodine receptor 44F; similar with RY44_DROME;
Px003970.1	-1.4540	0.00E+00	0.00E+00	Plasma membrane calcium-transporting ATPase 3;
Px013237.1	-1.4617	5.34E-169	2.91E-167	Ryanodine receptor 44F; similar with RY44_DROME;
Px008442.1	-1.4977	2.29E-09	1.29E-08	Sodium leak channel non-selective protein;
Px007169.1	-1.6039	1.11E-142	5.36E-141	Ryanodine receptor 44F;
Px008343.1	-1.6794	3.24E-35	5.68E-34	Voltage-dependent L-type calcium channel subunit beta-2;
Px003969.1	-1.8898	4.45E-12	3.12E-11	Plasma membrane calcium-transporting ATPase 3;
Px012737.1	-2.0831	3.17E-19	3.44E-18	Voltage-dependent L-type calcium channel subunit beta-2;
Px006829.9	-2.1487	1.74E-16	1.63E-15	Ryanodine receptor 44F; similar with RY44_DROME;
Px015884.1	-2.2297	3.22E-28	4.73E-27	Ryanodine receptor 44F; similar with RY44_DROME;
Px015604.1	-2.3122	1.59E-04	5.06E-04	Calcium channel flower; similar with FLOWR_DROWI;
Px001039.2	-2.3186	5.11E-12	3.57E-11	Voltage-dependent calcium channel type D subunit alpha-1;
Px004335.2	-2.8349	1.43E-27	2.05E-26	Ryanodine receptor 44F; similar with RY44_DROME;
**GO:0005262**				
Px010453.1	-1.0959	3.64E-05	1.29E-04	Ryanodine receptor 44F; similar with RY44_DROME;
Px014591.1	-1.0980	5.54E-10	3.30E-09	Inositol 1,4,5-trisphosphate receptor;
Px004866.1	-1.2725	1.52E-09	8.72E-09	Inositol 1,4,5-trisphosphate receptor;
Px003569.1	-1.3529	1.56E-28	2.30E-27	Voltage-dependent calcium channel type D subunit alpha-1;
Px016654.1	-1.4183	1.81E-80	5.97E-79	Ryanodine receptor 44F; similar with RY44_DROME;
Px013237.1	-1.4617	5.34E-169	2.91E-167	Ryanodine receptor 44F; similar with RY44_DROME;
Px008442.1	-1.4977	2.29E-09	1.29E-08	Sodium leak channel non-selective protein;
Px007169.1	-1.6039	1.11E-142	5.36E-141	Ryanodine receptor 44F; similar with RY44_DROME;
Px008343.1	-1.6794	3.24E-35	5.68E-34	Voltage-dependent L-type calcium channel subunit beta-2;
Px012737.1	-2.0831	3.17E-19	3.44E-18	Voltage-dependent L-type calcium channel subunit beta-2;
Px006829.9	-2.1487	1.74E-16	1.63E-15	Ryanodine receptor 44F; similar with RY44_DROME;
Px015884.1	-2.2297	3.22E-28	4.73E-27	Ryanodine receptor 44F; similar with RY44_DROME;
Px015604.1	-2.3122	1.59E-04	5.06E-04	Calcium channel flower; similar with FLOWR_DROWI;

Px001039.2	-2.3186	5.11E-12	3.57E-11	Voltage-dependent calcium channel type D subunit alpha-1;
Px004335.2	-2.8349	1.43E-27	2.05E-26	Ryanodine receptor 44F; similar with RY44_DROME;

Table J. A list of DEGs enriched in the GO terms of *P. xylostella* at 36 hpt_I verus 36 hpt_C

Gene ID	log_2 R	P-value	FDR	Annotation
**GO:0008233**				
Px015905.1	13.4712	1.77E-71	2.63E-70	Transmembrane protease serine 6;
Px003000.1	12.5364	2.28E-42	2.43E-41	Transmembrane protease serine 6;
Px007620.1	10.3950	8.15E-10	3.63E-09	Trypsin CFT-1; similar with TRYP_CHOFU;
Px006453.1	10.3391	8.15E-10	3.63E-09	Transmembrane protease serine 6;
Px006069.1	10.0136	2.26E-06	7.79E-06	Trypsin CFT-1; similar with TRYP_CHOFU;
Px017505.1	9.7974	1.71E-04	4.81E-04	Plasma kallikrein; similar with KLKB1_HUMAN;
Px007670.1	6.2472	5.75E-63	7.92E-62	Proclotting enzyme; similar with PCE_TACTR;
Px016055.1	4.5497	4.52E-56	5.70E-55	Trypsin, alkaline C; similar with TRYC_MANSE;
Px000995.2	4.3915	0.00E+00	0.00E+00	Carboxypeptidase A4; similar with CBPA4_HUMAN;
Px005241.1	4.3794	1.43E-53	1.74E-52	Trypsin CFT-1; similar with TRYP_CHOFU;
Px015276.1	4.0315	9.55E-65	1.33E-63	Trypsin CFT-1; similar with TRYP_CHOFU;
Px005243.1	3.9876	0.00E+00	0.00E+00	Trypsin, alkaline A; similar with TRYA_MANSE;
Px015277.1	3.9698	7.98E-158	2.09E-156	Trypsin CFT-1; similar with TRYP_CHOFU;
Px010313.2	3.7896	2.43E-106	4.78E-105	Serine proteinase stubble; similar with STUB_DROME;
Px006623.1	3.7777	1.10E-26	9.00E-26	Transmembrane protease serine 9;
Px013732.1	3.7553	4.20E-15	2.42E-14	Proteasome subunit alpha type-7-1;
Px012813.1	3.6622	2.98E-06	1.02E-05	Serine proteinase stubble; similar with STUB_DROME;
Px000992.1	3.6006	0.00E+00	0.00E+00	Carboxypeptidase A1; similar with CBPA1_BOVIN;
Px007617.1	3.5958	0.00E+00	0.00E+00	Trypsin CFT-1; similar with TRYP_CHOFU;
Px005765.1	3.4325	1.33E-62	1.82E-61	Vitellin-degrading protease; similar with VDP_BOMMO;
Px016056.1	3.4297	0.00E+00	0.00E+00	Trypsin, alkaline A; similar with TRYA_MANSE;
Px008538.1	3.4202	3.15E-138	7.39E-137	Trypsin, alkaline B; similar with TRYB_MANSE;
Px005657.1	3.4122	1.95E-23	1.46E-22	Proclotting enzyme; similar with PCE_TACTR;
Px012048.1	3.3901	4.29E-64	5.96E-63	Chymotrypsin BI; similar with CTRB1_LITVA;
Px007621.1	3.3847	6.61E-21	4.65E-20	Trypsin CFT-1; similar with TRYP_CHOFU;
Px008277.1	3.3443	1.09E-141	2.62E-140	Membrane alanyl aminopeptidase;
Px010386.1	3.1714	0.00E+00	0.00E+00	Trypsin CFT-1; similar with TRYP_CHOFU;
Px015928.1	3.1647	2.57E-04	7.05E-04	Carboxypeptidase B; similar with CBPB_ASTFL;
Px007619.2	3.0951	0.00E+00	0.00E+00	Trypsin, alkaline A; similar with TRYA_MANSE;
Px007676.1	3.0603	2.33E-107	4.62E-106	Chymotrypsin-C; similar with CTRC_HUMAN;
Px002861.1	3.0431	0.00E+00	0.00E+00	Trypsin, alkaline C; similar with TRYC_MANSE;
Px007903.1	2.9954	0.00E+00	0.00E+00	Collagenase; similar with COGS_HYPLI;
Px015049.1	2.9693	5.52E-35	5.22E-34	Venom dipeptidyl peptidase 4; similar with VDDP4_VESVU;
Px003479.1	2.9314	1.50E-17	9.44E-17	Protein rhomboid; similar with RHOM_DROME;
Px015280.1	2.9138	7.05E-10	3.15E-09	Trypsin CFT-1; similar with TRYP_CHOFU;
Px000994.1	2.8631	0.00E+00	0.00E+00	Zinc carboxypeptidase A 1; similar with CBPA1_DROPS;
Px002993.1	2.7717	0.00E+00	0.00E+00	Zinc metalloproteinase nas-4; similar with NAS4_CAEEL;
Px005373.1	2.7565	1.52E-39	1.56E-38	Carboxypeptidase B; similar with CBPB_ASTFL;
Px007618.1	2.7222	1.13E-272	4.41E-271	Trypsin CFT-1; similar with TRYP_CHOFU;
Px011887.1	2.7196	0.00E+00	0.00E+00	Trypsin, alkaline C; similar with TRYC_MANSE;
Px009286.1	2.7141	0.00E+00	0.00E+00	Trypsin, alkaline C; similar with TRYC_MANSE;
Px009784.1	2.6561	0.00E+00	0.00E+00	Ribosome-binding protein 1; similar with RRBP1_HUMAN;
Px016567.1	2.6458	1.14E-175	3.25E-174	Matrix metalloproteinase-14; similar with MMP14_RAT;
Px018074.1	2.6056	3.91E-24	2.97E-23	Chymotrypsin-like elastase family member 2A;
Px009757.1	2.5486	2.19E-10	1.01E-09	Proteasome subunit beta type-2; similar with PSB2_MOUSE;
Px007674.2	2.5239	0.00E+00	0.00E+00	Collagenase; similar with COGS_HYPLI;
Px007675.1	2.5233	0.00E+00	0.00E+00	Collagenase; similar with COGS_HYPLI;
Px007677.1	2.4787	0.00E+00	0.00E+00	Trypsin-7; similar with TRY7_ANOGA;
Px002588.1	2.4151	4.81E-14	2.66E-13	Trypsin, alkaline C; similar with TRYC_MANSE;
Px010489.1	2.4085	6.89E-66	9.73E-65	Trypsin, alkaline C; similar with TRYC_MANSE;

Px003467.1	2.3876	2.10E-18	1.36E-17	Matrix metalloproteinase-14; similar with MMP14_RABIT;
Px009395.1	2.3871	1.03E-17	6.47E-17	Proclotting enzyme; similar with PCE_TACTR;
Px007616.1	2.3746	1.09E-158	2.87E-157	Trypsin CFT-1; similar with TRYP_CHOFU;
Px001708.1	2.3708	0.00E+00	0.00E+00	Aminopeptidase N; similar with AMPN_PLUXY;
Px005242.1	2.3668	2.59E-19	1.73E-18	Trypsin CFT-1; similar with TRYP_CHOFU;
Px015275.1	2.3286	0.00E+00	0.00E+00	Trypsin CFT-1; similar with TRYP_CHOFU;
Px001705.1	2.3213	0.00E+00	0.00E+00	Membrane alanyl aminopeptidase;
Px006622.3	2.2728	9.45E-20	6.46E-19	Ovochymase-1; similar with OVCH1_HUMAN;
Px001990.2	2.2289	2.92E-104	5.69E-103	Carboxypeptidase B; similar with CBPB_ASTFL;
Px001633.1	2.2212	3.10E-80	5.00E-79	Angiotensin-converting enzyme;
Px011796.5	2.0740	1.05E-51	1.26E-50	Putative aminopeptidase W07G4.4;
Px001833.1	2.0603	0.00E+00	0.00E+00	Serine protease easter; similar with EAST_DROME;
Px002632.1	2.0480	2.47E-165	6.70E-164	Xaa-Pro dipeptidase; similar with PEPD_HUMAN;
Px016844.1	2.0366	3.06E-05	9.40E-05	Proteasome subunit beta type-1; similar with PSB1_RAT;
Px011097.1	1.9460	2.65E-10	1.22E-09	Serine proteinase stubble; similar with STUB_DROME;
Px003754.1	1.9228	1.67E-147	4.13E-146	Membrane alanyl aminopeptidase;
Px006956.1	1.8788	1.02E-112	2.08E-111	Xaa-Pro dipeptidase; similar with PEPD_HUMAN;
Px008406.1	1.8639	2.77E-08	1.10E-07	Serine proteinase stubble; similar with STUB_DROME;
Px012329.1	1.7693	5.39E-84	8.95E-83	Serine protease persephone; similar with PSH_DROME;
Px015778.1	1.7676	5.49E-08	2.14E-07	Sentrin-specific protease 8; similar with SENP8_HUMAN;
Px012643.1	1.7650	5.67E-95	1.02E-93	Angiotensin-converting enzyme; similar with ACE_MOUSE;
Px003753.1	1.7587	0.00E+00	0.00E+00	Membrane alanyl aminopeptidase;
Px000402.2	1.6821	5.77E-83	9.50E-82	Serine protease persephone; similar with PSH_DROME;
Px006502.1	1.6424	1.34E-55	1.68E-54	Calpain-B; similar with CANB_DROME;
Px009087.2	1.6281	1.83E-240	6.53E-239	Coagulation factor XII; similar with FA12_CAVPO;
Px008070.1	1.6216	3.59E-04	9.63E-04	Peroxisomal leader peptide-processing protease;
Px009715.1	1.5988	8.85E-16	5.24E-15	Mitochondrial inner membrane protease subunit 1;
Px011489.1	1.4705	1.48E-69	2.16E-68	Gamma-glutamyl hydrolase A; similar with GGHA_DICDI;
Px014831.2	1.4467	0.00E+00	0.00E+00	Serine proteinase stubble; similar with STUB_DROME;
Px005726.1	1.4124	1.92E-12	9.85E-12	Xaa-Pro aminopeptidase 1; similar with XPP1_HUMAN;
Px005747.1	1.4025	6.57E-16	3.92E-15	Proteasome subunit alpha type-5;
Px009568.1	1.3992	1.23E-04	3.53E-04	Trypsin, alkaline C; similar with TRYC_MANSE;
Px016171.2	1.3471	1.79E-54	2.21E-53	Prolyl endopeptidase; similar with PPCE_MOUSE;
Px003361.1	1.3252	8.90E-19	5.86E-18	Cysteine protease ATG4D; similar with ATG4D_MOUSE;
Px008337.1	1.2761	1.16E-111	2.35E-110	Leishmanolysin-like peptidase; similar with LMLN_DROME;
Px002715.1	1.2673	4.39E-154	1.12E-152	Cathepsin L; similar with CATL_DROME;
Px010436.1	1.1181	3.98E-92	7.03E-91	Venom serine carboxypeptidase; similar with VCP_APIME;
Px011311.1	1.1044	8.81E-09	3.65E-08	Proteasome subunit beta type-1;
Px008596.1	1.0870	1.05E-14	5.94E-14	Transmembrane protease serine 9;
Px016983.3	1.0379	6.64E-09	2.78E-08	Serine proteinase stubble; similar with STUB_DROME;
Px004984.1	1.0033	2.67E-04	7.30E-04	Peroxisomal leader peptide-processing protease;
Px001502.1	-1.0334	1.38E-18	9.02E-18	Ubiquitin carboxyl-terminal hydrolase 2;
Px013179.1	-1.1092	3.73E-04	9.98E-04	Insulin-degrading enzyme; similar with IDE_HUMAN;
Px004104.1	-1.1191	3.46E-06	1.17E-05	GH18371; similar with B4JEW4_DROGR;
Px000827.1	-1.1679	2.17E-07	8.16E-07	Ubiquitin carboxyl-terminal hydrolase 2;
Px009314.2	-1.3802	1.36E-12	7.02E-12	A disintegrin and metalloproteinase with thrombospondin motifs 16;
Px004087.1	-1.3985	1.15E-50	1.37E-49	Signal peptidase complex subunit 2;
Px000572.1	-1.4791	1.12E-04	3.23E-04	Phosphatidylinositol-binding clathrin assembly protein LAP;
Px002987.1	-1.5181	0.00E+00	0.00E+00	Methionine aminopeptidase 2; similar with AMPM2_BOVIN;
Px016787.1	-1.6669	1.01E-06	3.59E-06	Insulin-degrading enzyme; similar with IDE_HUMAN;
Px010814.1	-3.0382	1.61E-05	5.11E-05	ADAMTS-like protein 3; similar with ATL3_HUMAN;

GO:0016787

Px015905.1	13.4712	1.77E-71	2.63E-70	Transmembrane protease serine 6;

Px003000.1	12.5364	2.28E-42	2.43E-41	Transmembrane protease serine 6;
Px015074.1	11.6135	2.99E-08	1.19E-07	Dynein light chain Tctex-type 1;
Px009803.1	10.8775	1.46E-08	5.93E-08	Probable chitinase 2; similar with CHIT2_DROME;
Px012560.1	10.5968	1.26E-07	4.83E-07	Lipase member H-B; similar with LIPHB_XENLA;
Px007620.1	10.3950	8.15E-10	3.63E-09	Trypsin CFT-1; similar with TRYP_CHOFU;
Px001433.1	10.3916	1.08E-11	5.35E-11	Probable chitinase 2; similar with CHIT2_DROME;
Px006453.1	10.3391	8.15E-10	3.63E-09	Transmembrane protease serine 6;
Px006069.1	10.0136	2.26E-06	7.79E-06	Trypsin CFT-1; similar with TRYP_CHOFU;
Px017505.1	9.7974	1.71E-04	4.81E-04	Plasma kallikrein; similar with KLKB1_HUMAN;
Px014880.1	8.9447	4.64E-06	1.55E-05	ATP synthase subunit beta, mitochondrial;
Px013802.1	8.5527	1.46E-08	5.93E-08	Sodium/potassium-transporting ATPase subunit alpha-2;
Px016580.1	8.3897	3.51E-04	9.44E-04	T-complex protein 1 subunit epsilon;
Px015148.1	8.0644	1.96E-05	6.16E-05	ATP synthase subunit beta, mitochondrial;
Px015265.1	6.8160	1.67E-09	7.31E-09	Dynein beta chain, ciliary; similar with DYHC_TRIGR;
Px008062.1	6.7684	1.28E-182	3.76E-181	Endochitinase; similar with CHIT_MANSE;
Px001804.1	6.5119	2.83E-51	3.39E-50	Trypsin, alkaline B; similar with TRYB_MANSE;
Px007670.1	6.2472	5.75E-63	7.92E-62	Proclotting enzyme; similar with PCE_TACTR;
Px015893.1	4.9841	6.70E-09	2.81E-08	Putative inner dynein arm light chain, axonemal;
Px014882.1	4.8506	4.25E-162	1.13E-160	Trypsin, alkaline C; similar with TRYC_MANSE;
Px008276.1	4.6077	0.00E+00	0.00E+00	Membrane alanyl aminopeptidase;
Px016055.1	4.5497	4.52E-56	5.70E-55	Trypsin, alkaline C; similar with TRYC_MANSE;
Px002864.1	4.5298	1.55E-103	3.01E-102	Trypsin, alkaline B; similar with TRYB_MANSE;
Px000995.2	4.3915	0.00E+00	0.00E+00	Carboxypeptidase A4; similar with CBPA4_HUMAN;
Px005241.1	4.3794	1.43E-53	1.74E-52	Trypsin CFT-1; similar with TRYP_CHOFU;
Px000084.1	4.3183	5.99E-51	7.13E-50	Cysteine sulfinic acid decarboxylase;;
Px000291.1	4.0996	2.97E-16	1.79E-15	Myrosinase 1; similar with MYRO1_BREBR;
Px002586.1	4.0373	5.30E-58	6.84E-57	Trypsin CFT-1; similar with TRYP_CHOFU;
Px015276.1	4.0315	9.55E-65	1.33E-63	Trypsin CFT-1; similar with TRYP_CHOFU;
Px005243.1	3.9876	0.00E+00	0.00E+00	Trypsin, alkaline A; similar with TRYA_MANSE;
Px015277.1	3.9698	7.98E-158	2.09E-156	Trypsin CFT-1; similar with TRYP_CHOFU;
Px004490.1	3.9557	0.00E+00	0.00E+00	Adenosine deaminase CECR1;
Px006518.1	3.8846	1.12E-13	6.06E-13	Probable protein phosphatase 2C T23F11.1;
Px009202.1	3.8846	3.51E-04	9.44E-04	Myosin heavy chain, non-muscle;
Px016657.1	3.8498	2.94E-10	1.35E-09	Katanin p60 ATPase-containing subunit;
Px010313.2	3.7896	2.43E-106	4.78E-105	Serine proteinase stubble; similar with STUB_DROME;
Px006623.1	3.7777	1.10E-26	9.00E-26	Transmembrane protease serine 9;
Px013732.1	3.7553	4.20E-15	2.42E-14	Proteasome subunit alpha type-7-1;
Px012813.1	3.6622	2.98E-06	1.02E-05	Serine proteinase stubble; similar with STUB_DROME;
Px014201.1	3.6008	7.33E-11	3.48E-10	Sodium/potassium-transporting ATPase subunit alpha-2;
Px000992.1	3.6006	0.00E+00	0.00E+00	Carboxypeptidase A1; similar with CBPA1_BOVIN;
Px007617.1	3.5958	0.00E+00	0.00E+00	Trypsin CFT-1; similar with TRYP_CHOFU;
Px002116.1	3.5798	2.74E-08	1.10E-07	ATP-binding cassette sub-family G member 1;
Px008495.2	3.5537	1.86E-106	3.67E-105	Peptidoglycan-recognition protein LB;
Px005765.1	3.4325	1.33E-62	1.82E-61	Vitellin-degrading protease; similar with VDP_BOMMO;
Px016056.1	3.4297	0.00E+00	0.00E+00	Trypsin, alkaline A; similar with TRYA_MANSE;
Px008538.1	3.4202	3.15E-138	7.39E-137	Trypsin, alkaline B; similar with TRYB_MANSE;
Px005657.1	3.4122	1.95E-23	1.46E-22	Proclotting enzyme; similar with PCE_TACTR;
Px001762.1	3.3992	3.26E-11	1.58E-10	Sucrose-6-phosphate hydrolase;
Px012048.1	3.3901	4.29E-64	5.96E-63	Chymotrypsin BI; similar with CTRB1_LITVA;
Px007621.1	3.3847	6.61E-21	4.65E-20	Trypsin CFT-1; similar with TRYP_CHOFU;
Px017503.2	3.3503	2.24E-18	1.45E-17	Probable chitinase 2; similar with CHIT2_DROME;
Px008277.1	3.3443	1.09E-141	2.62E-140	Membrane alanyl aminopeptidase;
Px007120.2	3.3099	1.50E-19	1.02E-18	ATP synthase subunit beta, mitochondrial;
Px000993.1	3.2755	0.00E+00	0.00E+00	Carboxypeptidase A2; similar with CBPA2_RAT;

Px000991.6	3.2679	0.00E+00	0.00E+00	Carboxypeptidase O; similar with CBPO_BOVIN;
Px015279.1	3.2472	1.79E-18	1.17E-17	Trypsin, alkaline A; similar with TRYA_MANSE;
Px012568.1	3.2398	0.00E+00	0.00E+00	Trypsin CFT-1; similar with TRYP_CHOFU;
Px010386.1	3.1714	0.00E+00	0.00E+00	Trypsin CFT-1; similar with TRYP_CHOFU;
Px013274.1	3.1667	4.10E-69	5.97E-68	Ubiquitin carboxyl-terminal hydrolase 7;
Px015741.1	3.1647	2.57E-04	7.05E-04	Endoribonuclease Dicer; similar with DICER_BOVIN;
Px015928.1	3.1647	2.57E-04	7.05E-04	Carboxypeptidase B; similar with CBPB_ASTFL;
Px006580.1	3.1217	2.62E-07	9.75E-07	Mitochondrial cardiolipin hydrolase;
Px007619.2	3.0951	0.00E+00	0.00E+00	Trypsin, alkaline A; similar with TRYA_MANSE;
Px005671.1	3.0773	4.83E-07	1.76E-06	Pyrazinamidase/nicotinamidase;
Px007676.1	3.0603	2.33E-107	4.62E-106	Chymotrypsin-C; similar with CTRC_HUMAN;
Px002861.1	3.0431	0.00E+00	0.00E+00	Trypsin, alkaline C; similar with TRYC_MANSE;
Px009639.1	3.0376	0.00E+00	0.00E+00	Trypsin CFT-1; similar with TRYP_CHOFU;
Px007903.1	2.9954	0.00E+00	0.00E+00	Collagenase; similar with COGS_HYPLI;
Px005244.1	2.9796	7.91E-29	6.74E-28	Trypsin CFT-1; similar with TRYP_CHOFU;
Px015049.1	2.9693	5.52E-35	5.22E-34	Venom dipeptidyl peptidase 4;
Px005724.3	2.9382	3.59E-86	6.08E-85	Xaa-Pro aminopeptidase 1; similar with XPP1_MOUSE;
Px008281.1	2.9352	2.46E-11	1.20E-10	Kinesin-like protein KIF12; similar with KIF12_MOUSE;
Px003479.1	2.9314	1.50E-17	9.44E-17	Protein rhomboid; similar with RHOM_DROME;
Px011683.2	2.9306	0.00E+00	0.00E+00	Venom carboxylesterase-6; similar with EST6_APIME;
Px002784.1	2.9253	1.81E-07	6.86E-07	Multidrug resistance-associated protein 7;
Px015280.1	2.9138	7.05E-10	3.15E-09	Trypsin CFT-1; similar with TRYP_CHOFU;
Px009987.1	2.8978	1.47E-38	1.48E-37	Caspase-1; similar with CASP1_DROME;
Px000616.1	2.8942	0.00E+00	0.00E+00	Tubulin alpha chain; similar with TBA_TORMA;
Px000994.1	2.8631	0.00E+00	0.00E+00	Zinc carboxypeptidase A 1; similar with CBPA1_DROPS;
Px009638.1	2.8523	0.00E+00	0.00E+00	Trypsin, alkaline B; similar with TRYB_MANSE;
Px002141.1	2.8381	6.72E-42	7.09E-41	Probable phospholipid-transporting ATPase IF;
Px012332.1	2.8227	3.03E-20	2.09E-19	ATP synthase subunit beta, mitochondrial;
Px011608.1	2.8142	1.67E-04	4.72E-04	Fanconi-associated nuclease 1;
Px002587.1	2.7903	0.00E+00	0.00E+00	Trypsin CFT-1; similar with TRYP_CHOFU;
Px002993.1	2.7717	0.00E+00	0.00E+00	Zinc metalloproteinase nas-4; similar with NAS4_CAEEL;
Px005373.1	2.7565	1.52E-39	1.56E-38	Carboxypeptidase B; similar with CBPB_ASTFL;
Px002047.1	2.7527	1.74E-261	6.60E-260	Uncharacterized family 31 glucosidase KIAA1161;
Px016263.3	2.7402	1.48E-13	7.96E-13	ATP-binding cassette sub-family G member 4;
Px000107.1	2.7335	0.00E+00	0.00E+00	Trypsin, alkaline C; similar with TRYC_MANSE;
Px007618.1	2.7222	1.13E-272	4.41E-271	Trypsin CFT-1; similar with TRYP_CHOFU;
Px011887.1	2.7196	0.00E+00	0.00E+00	Trypsin, alkaline C; similar with TRYC_MANSE;
Px009286.1	2.7141	0.00E+00	0.00E+00	Trypsin, alkaline C; similar with TRYC_MANSE;
Px013288.1	2.7099	5.83E-272	2.27E-270	Tubulin beta chain; similar with TBB_PARLI;
Px013968.1	2.6816	1.66E-71	2.47E-70	Dual specificity protein phosphatase 19;
Px009784.1	2.6561	0.00E+00	0.00E+00	Ribosome-binding protein 1;
Px000119.2	2.6517	0.00E+00	0.00E+00	Inositol hexakisphosphate and diphosphoinositol-pentakisphosphate kinase;
Px016567.1	2.6458	1.14E-175	3.25E-174	Matrix metalloproteinase-14; similar with MMP14_RAT;
Px002441.1	2.6127	4.36E-75	6.75E-74	Trypsin, alkaline B; similar with TRYB_MANSE;
Px018074.1	2.6056	3.91E-24	2.97E-23	Chymotrypsin-like elastase family member 2A;
Px000395.1	2.6001	0.00E+00	0.00E+00	Alpha-amylase 4N; similar with AM4N_DROAN;
Px006728.1	2.5849	0.00E+00	0.00E+00	Sucrose-6-phosphate hydrolase;
Px006600.1	2.5735	7.58E-284	3.05E-282	Acetylcholinesterase; similar with ACES_BOVIN;
Px011359.1	2.5541	3.85E-26	3.07E-25	Dynein light chain roadblock-type 1;
Px009757.1	2.5486	2.19E-10	1.01E-09	Proteasome subunit beta type-2;
Px005866.1	2.5435	0.00E+00	0.00E+00	Zinc carboxypeptidase A 1;
Px001977.1	2.5367	1.82E-04	5.08E-04	Tyrosine-protein phosphatase 99A;
Px007674.2	2.5239	0.00E+00	0.00E+00	Collagenase; similar with COGS_HYPLI;
Px007675.1	2.5233	0.00E+00	0.00E+00	Collagenase; similar with COGS_HYPLI;

Px004489.1	2.5013	7.47E-48	8.61E-47	Adenosine deaminase CECR1;
Px009766.1	2.4923	6.69E-06	2.20E-05	Lysosomal Pro-X carboxypeptidase;
Px002046.1	2.4907	0.00E+00	0.00E+00	Uncharacterized family 31 glucosidase KIAA1161;
Px007677.1	2.4787	0.00E+00	0.00E+00	Trypsin-7; similar with TRY7_ANOGA;
Px012582.1	2.4724	2.13E-127	4.68E-126	Collagenase; similar with COGS_HYPLI;
Px004163.1	2.4696	2.57E-07	9.61E-07	Ubiquitin carboxyl-terminal hydrolase 8;
Px010022.2	2.4600	2.32E-49	2.71E-48	Cytosolic beta-glucosidase; similar with GBA3_CAVPO;
Px008848.1	2.4433	2.05E-168	5.68E-167	Myrosinase 1; similar with MYRO1_BREBR;
Px016058.1	2.4185	0.00E+00	0.00E+00	Trypsin, alkaline C; similar with TRYC_MANSE;
Px007814.1	2.4181	0.00E+00	0.00E+00	Multiple inositol polyphosphate phosphatase 1;
Px002588.1	2.4151	4.81E-14	2.66E-13	Trypsin, alkaline C; similar with TRYC_MANSE;
Px010489.1	2.4085	6.89E-66	9.73E-65	Trypsin, alkaline C; similar with TRYC_MANSE;
Px002416.1	2.4045	6.85E-76	1.07E-74	Probable multidrug resistance-associated protein letha;
Px009287.1	2.4027	1.27E-279	5.09E-278	Trypsin CFT-1; similar with TRYP_CHOFU;
Px006226.1	2.3992	1.06E-04	3.06E-04	Calpain-C; similar with CANC_DROME;
Px003467.1	2.3876	2.10E-18	1.36E-17	Matrix metalloproteinase-14; similar with MMP14_RABIT;
Px009395.1	2.3871	1.03E-17	6.47E-17	Proclotting enzyme; similar with PCE_TACTR;
Px003761.3	2.3858	4.84E-205	1.52E-203	Endoribonuclease Dicer; similar with DICER_BOVIN;
Px007616.1	2.3746	1.09E-158	2.87E-157	Trypsin CFT-1; similar with TRYP_CHOFU;
Px002209.3	2.3727	4.22E-16	2.54E-15	Dynein heavy chain 1, axonemal;
Px001708.1	2.3708	0.00E+00	0.00E+00	Aminopeptidase N; similar with AMPN_PLUXY;
Px005242.1	2.3668	2.59E-19	1.73E-18	Trypsin CFT-1; similar with TRYP_CHOFU;
Px002589.1	2.3435	3.45E-96	6.29E-95	Trypsin, alkaline A; similar with TRYA_MANSE;
Px004218.1	2.3409	0.00E+00	0.00E+00	Sucrose-6-phosphate hydrolase;
Px015275.1	2.3286	0.00E+00	0.00E+00	Trypsin CFT-1; similar with TRYP_CHOFU;
Px001761.1	2.3229	1.01E-228	3.51E-227	Sucrose-6-phosphate hydrolase;
Px001705.1	2.3213	0.00E+00	0.00E+00	Membrane alanyl aminopeptidase;
Px005876.1	2.3107	8.18E-70	1.20E-68	Patatin-like phospholipase domain-containing protein 2;
Px006622.3	2.2728	9.45E-20	6.46E-19	Ovochymase-1; similar with OVCH1_HUMAN;
Px001275.1	2.2595	6.17E-09	2.59E-08	Probable multidrug resistance-associated protein lethal;
Px009567.1	2.2552	3.00E-104	5.83E-103	Trypsin, alkaline B; similar with TRYB_MANSE;
Px002576.1	2.2413	7.31E-79	1.17E-77	Leucine-rich repeats and immunoglobulin-like domains protein 2;
Px001990.2	2.2289	2.92E-104	5.69E-103	Carboxypeptidase B; similar with CBPB_ASTFL;
Px009566.1	2.2251	1.29E-259	4.84E-258	Trypsin, alkaline C; similar with TRYC_MANSE;
Px006265.1	2.2241	3.46E-05	1.06E-04	Alpha-tocopherol transfer protein-like;
Px001633.1	2.2212	3.10E-80	5.00E-79	Angiotensin-converting enzyme; similar with ACE_PANTR;
Px007927.1	2.2154	4.79E-60	6.34E-59	V-type proton ATPase subunit G;
Px008913.1	2.1926	0.00E+00	0.00E+00	Acetylcholinesterase; similar with ACES_CULPI;
Px001878.1	2.1781	2.23E-21	1.59E-20	Lysosomal Pro-X carboxypeptidase;
Px004946.1	2.1753	3.57E-18	2.30E-17	ATP synthase subunit gamma, mitochondrial;
Px011886.1	2.1750	1.89E-125	4.14E-124	Trypsin CFT-1; similar with TRYP_CHOFU;
Px009347.1	2.1107	3.40E-32	3.07E-31	Protein shuttle craft; similar with STC_DROME;
Px011501.1	2.0881	7.26E-17	4.47E-16	ATP synthase subunit beta, mitochondrial;
Px011796.5	2.0740	1.05E-51	1.26E-50	Putative aminopeptidase W07G4.4;
Px009703.1	2.0631	0.00E+00	0.00E+00	Beta-1,3-glucan-binding protein;
Px016550.6	2.0608	2.43E-11	1.19E-10	Probable L-asparaginase GA20639;
Px001833.1	2.0603	0.00E+00	0.00E+00	Serine protease easter; similar with EAST_DROME;
Px002382.1	2.0597	1.62E-130	3.62E-129	Myosin-VIIa; similar with MYO7A_AEDAE;
Px002632.1	2.0480	2.47E-165	6.70E-164	Xaa-Pro dipeptidase; similar with PEPD_HUMAN;
Px004167.1	2.0469	1.15E-06	4.06E-06	Plasma kallikrein; similar with KLKB1_MOUSE;
Px016844.1	2.0366	3.06E-05	9.40E-05	Proteasome subunit beta type-1; similar with PSB1_RAT;
Px012569.1	2.0285	0.00E+00	0.00E+00	Trypsin, alkaline C; similar with TRYC_MANSE;
Px004726.1	2.0010	1.03E-29	8.97E-29	Alpha,alpha-trehalose-phosphate synthase [UDP-forming] 56 kDa subunit;
Px005079.1	1.9962	0.00E+00	0.00E+00	Lactase-phlorizin hydrolase; similar with LPH_HUMAN;

Px011097.1	1.9460	2.65E-10	1.22E-09	Serine proteinase stubble;
Px001111.1	1.9440	1.54E-14	8.66E-14	Probable multidrug resistance-associated protein lethal;
Px000116.1	1.9398	7.72E-10	3.45E-09	Protein phosphatase 1E; similar with PPM1E_RAT;
Px002889.1	1.9297	3.44E-08	1.36E-07	Dynein beta chain, ciliary; similar with DYHC_TRIGR;
Px003754.1	1.9228	1.67E-147	4.13E-146	Membrane alanyl aminopeptidase;
Px017171.1	1.9108	8.59E-16	5.10E-15	Putative inositol monophosphatase 3;
Px012937.2	1.8846	1.37E-05	4.37E-05	Phospholipase D1; similar with PLD1_HUMAN;
Px006956.1	1.8788	1.02E-112	2.08E-111	Xaa-Pro dipeptidase; similar with PEPD_HUMAN;
Px000394.1	1.8666	1.72E-210	5.53E-209	Alpha-amylase 4N; similar with AM4N_DROAN;
Px002866.1	1.8658	5.28E-09	2.23E-08	Trypsin, alkaline A; similar with TRYA_MANSE;
Px008406.1	1.8639	2.77E-08	1.10E-07	Serine proteinase stubble; similar with STUB_DROME;
Px004577.1	1.8549	4.19E-18	2.69E-17	Phosphotriesterase-related protein;
Px007992.2	1.8540	1.63E-24	1.25E-23	Multidrug resistance protein homolog 49;
Px013665.3	1.8475	2.46E-37	2.43E-36	Collagenase; similar with COGS_HYPLI;
Px004611.1	1.8428	1.21E-04	3.46E-04	Katanin p60 ATPase-containing subunit A-like 1;
Px016270.1	1.7997	7.53E-11	3.57E-10	Histone deacetylase 3; similar with HDAC3_TETNG;
Px015779.1	1.7984	0.00E+00	0.00E+00	Fatty-acid amide hydrolase 2;
Px013655.1	1.7878	3.45E-08	1.37E-07	Chitotriosidase-1; similar with CHIT1_HUMAN;
Px011697.1	1.7841	4.24E-28	3.56E-27	Bifunctional methylenetetrahydrofolate dehydrogenase/cyclohydrolase;
Px012329.1	1.7693	5.39E-84	8.95E-83	Serine protease persephone; similar with PSH_DROME;
Px015778.1	1.7676	5.49E-08	2.14E-07	Sentrin-specific protease 8; similar with SENP8_HUMAN;
Px012643.1	1.7650	5.67E-95	1.02E-93	Angiotensin-converting enzyme; similar with ACE_MOUSE;
Px003753.1	1.7587	0.00E+00	0.00E+00	Membrane alanyl aminopeptidase;
Px004725.1	1.7553	9.24E-06	3.01E-05	ATP-binding cassette sub-family G member 1;
Px008938.1	1.7508	1.35E-30	1.19E-29	Carboxypeptidase D; similar with CBPD_DROME;
Px014955.1	1.7471	5.65E-27	4.64E-26	ABC transporter G family member 23;
Px011231.1	1.7453	1.76E-27	1.46E-26	Protein unc-50 homolog; similar with UNC50_DROME;
Px010017.1	1.7418	0.00E+00	0.00E+00	Carboxypeptidase B; similar with CBPB_ASTFL;
Px004446.3	1.6970	2.01E-45	2.23E-44	Copper-transporting ATPase 2;
Px005403.1	1.6902	6.87E-06	2.26E-05	Probable multidrug resistance-associated protein lethal;
Px000402.2	1.6821	5.77E-83	9.50E-82	Serine protease persephone; similar with PSH_DROME;
Px007305.1	1.6562	9.37E-42	9.88E-41	C-1-tetrahydrofolate synthase, cytoplasmic;
Px003712.1	1.6490	1.05E-19	7.17E-19	Acetylcholinesterase; similar with ACES_CULPI;
Px006502.1	1.6424	1.34E-55	1.68E-54	Calpain-B; similar with CANB_DROME;
Px005342.1	1.6408	3.80E-156	9.82E-155	Collagenase; similar with COGS_HYPLI;
Px009087.2	1.6281	1.83E-240	6.53E-239	Coagulation factor XII; similar with FA12_CAVPO;
Px008070.1	1.6216	3.59E-04	9.63E-04	Peroxisomal leader peptide-processing protease;
Px004065.1	1.6192	1.53E-09	6.71E-09	Mannosyl-oligosaccharide alpha-1,2-mannosidase isoform A;
Px007904.1	1.6124	0.00E+00	0.00E+00	Collagenase; similar with COGS_HYPLI;
Px002015.1	1.6107	7.78E-06	2.55E-05	AGAP008123-PA; similar with Q7Q401_ANOGA;
Px007111.3	1.6050	2.44E-116	5.04E-115	ER degradation-enhancing alpha-mannosidase-like 2;
Px002919.1	1.6022	2.42E-22	1.76E-21	Putative N-acetylglucosamine-6-phosphate deacetylase;
Px009715.1	1.5988	8.85E-16	5.24E-15	Mitochondrial inner membrane protease subunit 1;
Px013162.1	1.5740	7.41E-151	1.88E-149	Serine protease snake; similar with SNAK_DROME;
Px017275.1	1.5732	4.40E-10	1.99E-09	Inositol monophosphatase 3;
Px006124.1	1.5716	2.01E-24	1.53E-23	Tubulin alpha-2/alpha-4 chain; similar with TBA2_PATVU;
Px002826.2	1.5627	1.01E-05	3.28E-05	CTD small phosphatase-like protein 2;
Px014172.1	1.5367	3.01E-07	1.11E-06	Heparanase; similar with HPSE_BOVIN;
Px001106.1	1.5290	1.97E-08	7.95E-08	Protein retinal degeneration B;
Px016262.1	1.5084	3.45E-19	2.30E-18	Cysteine sulfinic acid decarboxylase;
Px003229.1	1.5011	1.66E-09	7.28E-09	Dynein heavy chain 5, axonemal;
Px005265.1	1.4972	1.16E-08	4.78E-08	Ubiquitin carboxyl-terminal hydrolase 6;
Px016677.1	1.4862	1.49E-136	3.44E-135	ATP-binding cassette sub-family G member 1;
Px013984.1	1.4786	1.70E-11	8.37E-11	Dynein heavy chain 8, axonemal;

Px002706.1	1.4721	0.00E+00	0.00E+00	Lysozyme; similar with LYS_TRINI;
Px017858.1	1.4719	1.07E-34	1.01E-33	ATP-binding cassette sub-family G member 1;
Px011489.1	1.4705	1.48E-69	2.16E-68	Gamma-glutamyl hydrolase A;
Px011338.1	1.4635	5.55E-12	2.79E-11	GTP cyclohydrolase 1; similar with GCH1_DROME;
Px015886.1	1.4619	1.21E-04	3.45E-04	ATP-dependent DNA helicase Q5;
Px000855.1	1.4469	1.40E-135	3.22E-134	Juvenile hormone epoxide hydrolase;
Px014831.2	1.4467	0.00E+00	0.00E+00	Serine proteinase stubble; similar with STUB_DROME;
Px014586.1	1.4462	1.33E-30	1.17E-29	Tubulointerstitial nephritis antigen;
Px007510.1	1.4398	3.94E-10	1.80E-09	Spectrin beta chain; similar with SPTCB_DROME;
Px015740.3	1.4224	8.84E-08	3.41E-07	Endoribonuclease Dcr-1; similar with DCR1_DROME;
Px008607.1	1.4201	1.74E-29	1.51E-28	Ras-related protein Rap-2b; similar with RAP2B_RAT;
Px017644.1	1.4197	3.55E-06	1.20E-05	Dynein heavy chain 10, axonemal;
Px013053.1	1.4168	5.05E-81	8.20E-80	Endoribonuclease Dicer; similar with DICER_MOUSE;
Px008806.2	1.4150	2.28E-44	2.49E-43	Hydroxyacylglutathione hydrolase, mitochondrial;
Px007577.1	1.4133	8.83E-12	4.40E-11	Protein N-terminal asparagine amidohydrolase;
Px005726.1	1.4124	1.92E-12	9.85E-12	Xaa-Pro aminopeptidase 1; similar with XPP1_HUMAN;
Px016080.1	1.4117	3.08E-05	9.48E-05	Glucosamine-6-phosphate isomerase;
Px008591.1	1.4098	1.56E-10	7.26E-10	Hexosaminidase D; similar with HEXDC_HUMAN;
Px012191.1	1.4028	2.38E-208	7.61E-207	Sodium/potassium-transporting ATPase subunit alpha-1;
Px005747.1	1.4025	6.57E-16	3.92E-15	Proteasome subunit alpha type-5;
Px003398.1	1.3992	2.33E-08	9.33E-08	Probable phospholipid-transporting ATPase IA;
Px009568.1	1.3992	1.23E-04	3.53E-04	Trypsin, alkaline C; similar with TRYC_MANSE;
Px001847.1	1.3803	1.09E-180	3.16E-179	Aminoacylase-1; similar with ACY1_MOUSE;
Px005341.1	1.3783	1.92E-45	2.13E-44	Collagenase; similar with COGS_HYPLI;
Px001370.1	1.3780	6.09E-19	4.04E-18	Aminoacylase-1; similar with ACY1_MOUSE;
Px013252.1	1.3766	0.00E+00	0.00E+00	Calexcitin-2; similar with CEX2_CAEEL;
Px004590.1	1.3727	8.33E-05	2.43E-04	Dynein heavy chain 7, axonemal; similar with DYH7_RAT;
Px000087.2	1.3668	5.17E-07	1.88E-06	ATP-binding cassette sub-family G member 4;
Px005329.1	1.3592	4.00E-66	5.66E-65	Protein ETHE1, mitochondrial;
Px009798.1	1.3537	1.57E-40	1.63E-39	Aminoacylase-1A; similar with ACY1A_RAT;
Px006663.1	1.3501	2.50E-38	2.52E-37	Ribonuclease Oy; similar with RNOY_CRAGI;
Px010161.1	1.3476	3.12E-26	2.50E-25	GTP cyclohydrolase 1; similar with GCH1_DROME;
Px016171.2	1.3471	1.79E-54	2.21E-53	Prolyl endopeptidase; similar with PPCE_MOUSE;
Px007369.1	1.3403	4.19E-08	1.65E-07	DNA excision repair protein haywire;
Px009117.1	1.3403	1.32E-09	5.80E-09	Phosphotriesterase-related protein;
Px015923.1	1.3371	5.16E-07	1.88E-06	OTU domain-containing protein 1;
Px012027.3	1.3347	9.75E-93	1.73E-91	Protein 5NUC; similar with 5NTD_LUTLO;
Px012523.1	1.3305	6.77E-16	4.04E-15	Ras-related protein Rab-35; similar with RAB35_RAT;
Px003361.1	1.3252	8.90E-19	5.86E-18	Cysteine protease ATG4D; similar with ATG4D_MOUSE;
Px007906.2	1.3236	0.00E+00	0.00E+00	Collagenase; similar with COGS_HYPLI;
Px009982.1	1.3211	1.20E-43	1.30E-42	5-oxoprolinase; similar with OPLA_HUMAN;
Px003782.1	1.3205	7.56E-110	1.51E-108	Carboxypeptidase E; similar with CBPE_LOPAM;
Px009887.1	1.3111	4.97E-09	2.10E-08	Probable protein phosphatase 2C T23F11.1;
Px016664.1	1.3099	2.71E-18	1.75E-17	DNA replication complex GINS protein PSF1;
Px008217.3	1.3035	8.78E-09	3.64E-08	Phosphatidylinositol 4,5-bisphosphate 5-phosphatase A;
Px000513.1	1.2987	1.37E-31	1.23E-30	Teneurin-2; similar with TEN2_MOUSE;
Px016679.1	1.2867	1.13E-05	3.64E-05	ATP-binding cassette transporter subfamily G;
Px008337.1	1.2761	1.16E-111	2.35E-110	Leishmanolysin-like peptidase;
Px002715.1	1.2673	4.39E-154	1.12E-152	Cathepsin L; similar with CATL_DROME;
Px013298.1	1.2577	3.36E-51	4.02E-50	Serine proteinase-like protein;
Px001059.1	1.2567	2.89E-79	4.64E-78	Beta-1,3-glucan-binding protein;
Px002767.1	1.2554	0.00E+00	0.00E+00	Spectrin alpha chain; similar with SPTCA_DROME;
Px006054.1	1.2358	6.60E-273	2.59E-271	Myrosinase 1; similar with MYRO1_BREBR;
Px001277.2	1.2325	1.00E-06	3.55E-06	Probable phospholipid-transporting ATPase IIB;

Px006295.1	1.2186	2.27E-05	7.07E-05	ATP-dependent RNA helicase DDX18;
Px012198.1	1.2125	2.46E-68	3.54E-67	Fatty acid synthase; similar with FAS_RAT;
Px004810.1	1.2046	7.06E-09	2.95E-08	ADP-ribosylation factor-like protein 2;
Px011015.1	1.1970	2.82E-07	1.05E-06	NAD-dependent deacetylase sirtuin-5;
Px001933.1	1.1912	1.41E-09	6.20E-09	Myrosinase 1; similar with MYRO1_BREBR;
Px005839.1	1.1906	1.80E-18	1.17E-17	Mannosyl-oligosaccharide alpha-1,2-mannosidase isoform A;
Px008946.1	1.1905	9.69E-78	1.54E-76	Dynein heavy chain 7, axonemal;
Px005376.1	1.1799	4.53E-08	1.78E-07	Chitotriosidase-1; similar with CHIT1_HUMAN;
Px000499.1	1.1782	3.06E-54	3.76E-53	Inositol hexakisphosphate and diphosphoinositol-pentakisphosphate kinase;
Px008144.2	1.1768	3.02E-08	1.20E-07	Serine/threonine-protein kinase/endoribonuclease ire-1;
Px015447.4	1.1577	9.03E-08	3.48E-07	Probable multidrug resistance-associated protein lethal;
Px009700.1	1.1567	6.53E-07	2.36E-06	Phosphotriesterase-related protein;
Px000966.1	1.1548	2.65E-18	1.71E-17	Omega-amidase NIT2; similar with NIT2_BOVIN;
Px009523.1	1.1532	3.53E-05	1.08E-04	N(4)-(Beta-N-acetylglucosaminyl)-L-asparaginase;
Px017788.1	1.1517	2.14E-54	2.63E-53	Serine protease snake; similar with SNAK_DROME;
Px010099.1	1.1453	2.76E-19	1.85E-18	Phospholipase D1; similar with PLD1_RAT;
Px016675.1	1.1203	5.63E-11	2.69E-10	ATP-binding cassette sub-family G member 1;
Px010436.1	1.1181	3.98E-92	7.03E-91	Venom serine carboxypeptidase; similar with VCP_APIME;
Px011311.1	1.1044	8.81E-09	3.65E-08	Proteasome subunit beta type-1;
Px003316.1	1.0981	0.00E+00	0.00E+00	C-1-tetrahydrofolate synthase, cytoplasmic;
Px016926.1	1.0912	1.86E-15	1.08E-14	Ubiquitin carboxyl-terminal hydrolase 20;
Px008596.1	1.0870	1.05E-14	5.94E-14	Transmembrane protease serine 9;
Px005110.3	1.0773	2.59E-04	7.09E-04	ABC transporter G family member 23;
Px009393.2	1.0773	2.59E-04	7.09E-04	Guanine deaminase; similar with GUAD_PONAB;
Px007185.1	1.0652	1.08E-11	5.33E-11	ATP-binding cassette sub-family G member 4;
Px013543.1	1.0629	1.37E-18	8.94E-18	Matrix metalloproteinase-25
Px007850.1	1.0586	5.47E-08	2.14E-07	Dynein heavy chain 2, axonemal;
Px015503.1	1.0510	1.25E-10	5.86E-10	Histone deacetylase 4; similar with HDAC4_MOUSE;
Px002232.1	1.0403	8.42E-08	3.25E-07	Spectrin alpha chain; similar with SPTCA_DROME;
Px016983.3	1.0379	6.64E-09	2.78E-08	Serine proteinase stubble; similar with STUB_DROME;
Px013730.1	1.0378	8.17E-18	5.18E-17	Myrosinase 1; similar with MYRO1_BREBR;
Px008659.1	1.0347	2.76E-08	1.10E-07	Tyrosine-protein phosphatase non-receptor type 23;
Px006391.1	1.0276	4.24E-21	3.00E-20	ATP-binding cassette sub-family B member 6, mitochondrial;
Px010631.1	1.0225	2.51E-26	2.02E-25	Sphingomyelin phosphodiesterase;
Px004326.1	1.0176	3.65E-04	9.79E-04	PI-PLC X domain-containing protein 3;
Px004984.1	1.0033	2.67E-04	7.30E-04	Peroxisomal leader peptide-processing protease;
Px012297.1	1.0024	1.28E-14	7.21E-14	ABC transporter G family member 20;
Px002093.2	1.0006	1.18E-05	3.78E-05	Ras-like protein 1; similar with RAS1_DROYA;
Px002896.2	-1.0039	5.09E-96	9.24E-95	Recombination repair protein 1; similar with RRP1_DROME;
Px014740.1	-1.0140	7.74E-60	1.02E-58	Probable ATP-dependent RNA helicase DDX27;
Px009226.1	-1.0167	9.86E-10	4.37E-09	DNA replication licensing factor MCM4;
Px002786.1	-1.0167	2.80E-85	4.70E-84	Elongation factor Tu, mitochondrial;
Px008828.1	-1.0322	1.20E-16	7.32E-16	Uncharacterized protein C2orf79 homolog;
Px001502.1	-1.0334	1.38E-18	9.02E-18	Ubiquitin carboxyl-terminal hydrolase 2;
Px014608.2	-1.0993	1.84E-38	1.86E-37	AGAP011281-PA; similar with Q7QHP0_ANOGA;
Px013179.1	-1.1092	3.73E-04	9.98E-04	Insulin-degrading enzyme; similar with IDE_HUMAN;
Px004104.1	-1.1191	3.46E-06	1.17E-05	GH18371; similar with B4JEW4_DROGR;
Px015490.1	-1.1584	5.68E-45	6.25E-44	Probable ATP-dependent RNA helicase DDX52;
Px000827.1	-1.1679	2.17E-07	8.16E-07	Ubiquitin carboxyl-terminal hydrolase 2;
Px017914.1	-1.1872	0.00E+00	0.00E+00	Paramyosin, long form; similar with MYSP1_DROME;
Px002758.1	-1.2096	1.96E-07	7.39E-07	RNA polymerase II subunit B1 CTD phosphatase Rpap2;
Px017913.1	-1.2473	0.00E+00	0.00E+00	Paramyosin, short form; similar with MYSP2_DROME;
Px007815.1	-1.2476	0.00E+00	0.00E+00	Myosin heavy chain, muscle;
Px012158.1	-1.2615	1.63E-10	7.54E-10	Alpha-N-acetylgalactosaminidase;

Px016541.1	-1.2656	6.55E-07	2.37E-06	Elongation factor Tu, mitochondrial;
Px002640.1	-1.2858	0.00E+00	0.00E+00	Alpha-actinin, sarcomeric; similar with ACTN_DROME;
Px012129.1	-1.2949	1.78E-33	1.64E-32	Lysosomal Pro-X carboxypeptidase;
Px015632.1	-1.3238	2.97E-07	1.10E-06	Flap endonuclease 1; similar with FEN1_DROAN;
Px009149.1	-1.3366	4.40E-70	6.46E-69	116 kDa U5 small nuclear ribonucleoprotein component;
Px008876.1	-1.3446	1.07E-09	4.74E-09	Tubulin beta-4 chain; similar with TBB4_CAEEL;
Px010423.1	-1.3483	1.94E-09	8.45E-09	G/T mismatch-specific thymine DNA glycosylase;
Px017176.1	-1.3522	4.34E-23	3.21E-22	Probable ATP-dependent RNA helicase kurz;
Px010023.2	-1.3680	1.39E-22	1.02E-21	ATP-dependent RNA helicase DDX24;
Px009314.2	-1.3802	1.36E-12	7.02E-12	A disintegrin and metalloproteinase with thrombospondin motifs 16;
Px008352.1	-1.3967	7.24E-17	4.46E-16	SOX domain-containing protein dichaete;
Px004087.1	-1.3985	1.15E-50	1.37E-49	Signal peptidase complex subunit 2;
Px009337.1	-1.4082	8.00E-05	2.35E-04	M-phase inducer phosphatase;
Px006766.1	-1.4330	1.04E-119	2.20E-118	ATP-binding cassette sub-family F member 1;
Px011795.1	-1.4417	5.27E-29	4.51E-28	RuvB-like helicase 2; similar with RUVB2_DROPS;
Px000037.1	-1.4737	2.19E-29	1.89E-28	DNA replication licensing factor Mcm5;
Px000572.1	-1.4791	1.12E-04	3.23E-04	Phosphatidylinositol-binding clathrin assembly protein LAP;
Px016332.1	-1.5166	0.00E+00	0.00E+00	Calcium-transporting ATPase sarcoplasmic/endoplasmic reticulum type;
Px002987.1	-1.5181	0.00E+00	0.00E+00	Methionine aminopeptidase 2;
Px010951.2	-1.5366	3.53E-143	8.64E-142	Lysosomal alpha-mannosidase; similar with MA2B1_CAVPO;
Px013405.1	-1.5636	1.01E-18	6.66E-18	CAP isoform A; similar with C9DTM4_BOMMO;
Px016787.1	-1.6669	1.01E-06	3.59E-06	Insulin-degrading enzyme; similar with IDE_HUMAN;
Px003941.1	-1.7142	4.69E-05	1.42E-04	Probable phospholipid-transporting ATPase IA;
Px014610.1	-1.7464	1.84E-20	1.28E-19	Tubulin beta-2 chain; similar with TBB2_HOMAM;
Px011896.1	-2.2353	0.00E+00	0.00E+00	PAB-dependent poly(A)-specific ribonuclease subunit 2;
Px012510.1	-2.2884	1.67E-09	7.30E-09	N-glycosylase/DNA lyase; similar with OGG1_DROME;
Px009429.3	-2.5772	1.39E-110	2.81E-109	ATP-dependent RNA helicase DDX24;
Px017404.1	-2.6008	9.07E-06	2.95E-05	Fidgetin-like protein 1; similar with FIGL1_XENLA;
Px012212.1	-2.8769	4.13E-06	1.39E-05	Multidrug resistance protein homolog 49;
Px010814.1	-3.0382	1.61E-05	5.11E-05	ADAMTS-like protein 3; similar with ATL3_HUMAN;
Px012074.1	#######	3.46E-04	9.33E-04	Acylphosphatase-2; similar with ACYP2_ANAPL;

GO:0003824

Px015905.1	13.4712	1.77E-71	2.63E-70	Transmembrane protease serine 6;
Px003000.1	12.5364	2.28E-42	2.43E-41	Transmembrane protease serine 6;
Px015074.1	11.6135	2.99E-08	1.19E-07	Dynein light chain Tctex-type 1;
Px003417.1	11.0707	4.48E-16	2.69E-15	Beta-1,3-galactosyltransferase 5;
Px012802.1	10.9217	9.55E-06	3.10E-05	GH16830; similar with B4IWX8_DROGR;
Px009803.1	10.8775	1.46E-08	5.93E-08	Probable chitinase 2; similar with CHIT2_DROME;
Px008374.1	10.7904	2.99E-08	1.19E-07	Phosphatidylinositol-4,5-bisphosphate 3-kinase catalytic subunit delta isoform;
Px012560.1	10.5968	1.26E-07	4.83E-07	Lipase member H-B; similar with LIPHB_XENLA;
Px009423.1	10.3964	3.44E-09	1.47E-08	Putative uncharacterized protein;
Px007620.1	10.3950	8.15E-10	3.63E-09	Trypsin CFT-1; similar with TRYP_CHOFU;
Px001433.1	10.3916	1.08E-11	5.35E-11	Probable chitinase 2; similar with CHIT2_DROME;
Px006453.1	10.3391	8.15E-10	3.63E-09	Transmembrane protease serine 6;
Px006069.1	10.0136	2.26E-06	7.79E-06	Trypsin CFT-1; similar with TRYP_CHOFU;
Px009935.1	9.9490	3.51E-04	9.43E-04	Cytochrome P450 6d3; similar with CP6D3_MUSDO;
Px011676.1	9.9119	4.04E-05	1.22E-04	Alpha-aminoadipic semialdehyde synthase, mitochondrial;
Px017505.1	9.7974	1.71E-04	4.81E-04	Plasma kallikrein; similar with KLKB1_HUMAN;
Px002969.1	9.6359	1.96E-05	6.16E-05	Adenylate kinase isoenzyme 1; similar with KAD1_RAT;
Px008029.1	9.1359	8.30E-05	2.43E-04	cAMP-dependent protein kinase catalytic subunit alpha;
Px014880.1	8.9447	4.64E-06	1.55E-05	ATP synthase subunit beta, mitochondrial;
Px013802.1	8.5527	1.46E-08	5.93E-08	Sodium/potassium-transporting ATPase subunit alpha-2;
Px016580.1	8.3897	3.51E-04	9.44E-04	T-complex protein 1 subunit epsilon;

Px015148.1	8.0644	1.96E-05	6.16E-05	ATP synthase subunit beta, mitochondrial;
Px015265.1	6.8160	1.67E-09	7.31E-09	Dynein beta chain, ciliary; similar with DYHC_TRIGR;
Px008062.1	6.7684	1.28E-182	3.76E-181	Endochitinase; similar with CHIT_MANSE;
Px008830.1	6.6433	1.43E-111	2.90E-110	Trypsin, alkaline C; similar with TRYC_MANSE;
Px001804.1	6.5119	2.83E-51	3.39E-50	Trypsin, alkaline B; similar with TRYB_MANSE;
Px007670.1	6.2472	5.75E-63	7.92E-62	Proclotting enzyme; similar with PCE_TACTR;
Px010971.1	5.2472	1.05E-10	4.92E-10	Trypsin CFT-1; similar with TRYP_CHOFU;
Px015893.1	4.9841	6.70E-09	2.81E-08	Putative inner dynein arm light chain, axonemal;
Px014882.1	4.8506	4.25E-162	1.13E-160	Trypsin, alkaline C; similar with TRYC_MANSE;
Px015921.1	4.7402	1.00E-39	1.03E-38	Putative uncharacterized protein;
Px008276.1	4.6077	0.00E+00	0.00E+00	Membrane alanyl aminopeptidase;
Px016055.1	4.5497	4.52E-56	5.70E-55	Trypsin, alkaline C; similar with TRYC_MANSE;
Px002864.1	4.5298	1.55E-103	3.01E-102	Trypsin, alkaline B; similar with TRYB_MANSE;
Px000995.2	4.3915	0.00E+00	0.00E+00	Carboxypeptidase A4; similar with CBPA4_HUMAN;
Px005241.1	4.3794	1.43E-53	1.74E-52	Trypsin CFT-1; similar with TRYP_CHOFU;
Px005268.1	4.3252	1.24E-05	3.97E-05	Glucose dehydrogenase [acceptor];
Px000084.1	4.3183	5.99E-51	7.13E-50	Cysteine sulfinic acid decarboxylase;
Px016053.1	4.2124	5.01E-59	6.53E-58	Trypsin; similar with TRYP_PHACE;
Px000702.1	4.1858	2.08E-17	1.30E-16	NADH dehydrogenase [ubiquinone] iron-sulfur protein 2, mitochondrial;
Px009637.1	4.1028	1.35E-27	1.13E-26	Trypsin CFT-1; similar with TRYP_CHOFU;
Px000291.1	4.0996	2.97E-16	1.79E-15	Myrosinase 1; similar with MYRO1_BREBR;
Px002586.1	4.0373	5.30E-58	6.84E-57	Trypsin CFT-1; similar with TRYP_CHOFU;
Px013377.1	4.0330	2.06E-219	6.86E-218	Trypsin; similar with TRYP_PHACE;
Px015276.1	4.0315	9.55E-65	1.33E-63	Trypsin CFT-1; similar with TRYP_CHOFU;
Px000359.2	4.0159	3.40E-22	2.47E-21	Alpha-methyldopa hypersensitive protein;
Px005243.1	3.9876	0.00E+00	0.00E+00	Trypsin, alkaline A; similar with TRYA_MANSE;
Px015277.1	3.9698	7.98E-158	2.09E-156	Trypsin CFT-1; similar with TRYP_CHOFU;
Px004490.1	3.9557	0.00E+00	0.00E+00	Adenosine deaminase CECR1;
Px014859.2	3.9352	1.13E-07	4.34E-07	Uridine phosphorylase 1; similar with UPP1_MOUSE;
Px012913.1	3.8855	0.00E+00	0.00E+00	Chitin synthase 6; similar with CHS6_USTMA;
Px006518.1	3.8846	1.12E-13	6.06E-13	Probable protein phosphatase 2C T23F11.1;
Px009202.1	3.8846	3.51E-04	9.44E-04	Myosin heavy chain, non-muscle;
Px016657.1	3.8498	2.94E-10	1.35E-09	Katanin p60 ATPase-containing subunit;
Px009019.1	3.7997	1.15E-15	6.76E-15	Uridine phosphorylase 1; similar with UPP1_MOUSE;
Px010313.2	3.7896	2.43E-106	4.78E-105	Serine proteinase stubble; similar with STUB_DROME;
Px006571.1	3.7885	0.00E+00	0.00E+00	Trypsin, alkaline B; similar with TRYB_MANSE;
Px006623.1	3.7777	1.10E-26	9.00E-26	Transmembrane protease serine 9;
Px009254.3	3.7703	2.83E-43	3.04E-42	Cyclin-dependent kinase-like 2;
Px013732.1	3.7553	4.20E-15	2.42E-14	Proteasome subunit alpha type-7-1;
Px009370.2	3.7497	2.91E-12	1.48E-11	Prostaglandin reductase 1; similar with PTGR1_BOVIN;
Px002634.1	3.7211	1.56E-06	5.44E-06	Beta-1,4-N-acetylgalactosaminyltransferase bre-4;
Px008031.1	3.6920	1.06E-11	5.27E-11	cAMP-dependent protein kinase catalytic subunit alpha;
Px012045.3	3.6672	2.30E-186	6.84E-185	Glucose dehydrogenase [acceptor];
Px012813.1	3.6622	2.98E-06	1.02E-05	Serine proteinase stubble; similar with STUB_DROME;
Px016942.1	3.6242	1.71E-40	1.77E-39	NADH dehydrogenase [ubiquinone] 1 beta subcomplex subunit 9;
Px014201.1	3.6008	7.33E-11	3.48E-10	Sodium/potassium-transporting ATPase subunit alpha-2;
Px000992.1	3.6006	0.00E+00	0.00E+00	Carboxypeptidase A1; similar with CBPA1_BOVIN;
Px000114.1	3.5971	6.55E-42	6.92E-41	Sulfotransferase 1C4; similar with ST1C4_HUMAN;
Px007617.1	3.5958	0.00E+00	0.00E+00	Trypsin CFT-1; similar with TRYP_CHOFU;
Px011202.1	3.5882	8.25E-143	2.01E-141	Aromatic-L-amino-acid decarboxylase;
Px002116.1	3.5798	2.74E-08	1.10E-07	ATP-binding cassette sub-family G member 1;
Px008495.2	3.5537	1.86E-106	3.67E-105	Peptidoglycan-recognition protein LB;
Px008783.1	3.5367	1.08E-05	3.49E-05	Long-chain fatty acid transport protein 1;
Px006572.1	3.4867	0.00E+00	0.00E+00	Trypsin; similar with TRYP_PHACE;

Px003371.1	3.4542	6.30E-39	6.39E-38	NADH dehydrogenase [ubiquinone] 1 alpha subcomplex subunit 8;
Px005765.1	3.4325	1.33E-62	1.82E-61	Vitellin-degrading protease;
Px016056.1	3.4297	0.00E+00	0.00E+00	Trypsin, alkaline A; similar with TRYA_MANSE;
Px008538.1	3.4202	3.15E-138	7.39E-137	Trypsin, alkaline B; similar with TRYB_MANSE;
Px005657.1	3.4122	1.95E-23	1.46E-22	Proclotting enzyme; similar with PCE_TACTR;
Px013214.1	3.3992	3.25E-13	1.73E-12	Peroxisomal N(1)-acetyl-spermine/spermidine oxidase;
Px001762.1	3.3992	3.26E-11	1.58E-10	Sucrose-6-phosphate hydrolase;
Px012048.1	3.3901	4.29E-64	5.96E-63	Chymotrypsin BI; similar with CTRB1_LITVA;
Px007621.1	3.3847	6.61E-21	4.65E-20	Trypsin CFT-1; similar with TRYP_CHOFU;
Px006617.2	3.3534	1.05E-31	9.40E-31	Chorion peroxidase; similar with PERC_DROME;
Px017503.2	3.3503	2.24E-18	1.45E-17	Probable chitinase 2; similar with CHIT2_DROME;
Px008277.1	3.3443	1.09E-141	2.62E-140	Membrane alanyl aminopeptidase;
Px007120.2	3.3099	1.50E-19	1.02E-18	ATP synthase subunit beta, mitochondrial;
Px000993.1	3.2755	0.00E+00	0.00E+00	Carboxypeptidase A2; similar with CBPA2_RAT;
Px000991.6	3.2679	0.00E+00	0.00E+00	Carboxypeptidase O; similar with CBPO_BOVIN;
Px006485.1	3.2472	1.38E-04	3.92E-04	Activin receptor type-1; similar with ACVR1_MOUSE;
Px013989.1	3.2472	4.09E-08	1.62E-07	NADH dehydrogenase [ubiquinone] flavoprotein 2, mitochondrial;
Px015279.1	3.2472	1.79E-18	1.17E-17	Trypsin, alkaline A; similar with TRYA_MANSE;
Px004168.1	3.2472	2.59E-13	1.38E-12	Plasma kallikrein; similar with KLKB1_HUMAN;
Px012568.1	3.2398	0.00E+00	0.00E+00	Trypsin CFT-1; similar with TRYP_CHOFU;
Px007471.1	3.2141	3.12E-82	5.11E-81	Pyruvate kinase; similar with KPYK_DROME;
Px013918.1	3.2126	0.00E+00	0.00E+00	Putative uncharacterized protein;
Px005513.1	3.1816	1.98E-25	1.56E-24	Polypeptide N-acetylgalactosaminyltransferase 1;
Px010386.1	3.1714	0.00E+00	0.00E+00	Trypsin CFT-1; similar with TRYP_CHOFU;
Px013274.1	3.1667	4.10E-69	5.97E-68	Ubiquitin carboxyl-terminal hydrolase 7;
Px015741.1	3.1647	2.57E-04	7.05E-04	Endoribonuclease Dicer; similar with DICER_BOVIN;
Px015928.1	3.1647	2.57E-04	7.05E-04	Carboxypeptidase B; similar with CBPB_ASTFL;
Px009262.1	3.1647	2.57E-04	7.05E-04	Slowpoke-binding protein; similar with SLOB_DROME;
Px013999.1	3.1647	1.41E-07	5.38E-07	GL23714; similar with B4G658_DROPE;
Px001081.1	3.1539	0.00E+00	0.00E+00	Unknown function
Px016054.1	3.1536	3.24E-37	3.20E-36	Trypsin; similar with TRYP_PHACE;
Px006580.1	3.1217	2.62E-07	9.75E-07	Mitochondrial cardiolipin hydrolase;
Px007619.2	3.0951	0.00E+00	0.00E+00	Trypsin, alkaline A; similar with TRYA_MANSE;
Px005368.1	3.0773	1.61E-47	1.85E-46	Malate dehydrogenase 2, mitochondrial;
Px005671.1	3.0773	4.83E-07	1.76E-06	Pyrazinamidase/nicotinamidase; similar with PNCA_ECOLI;
Px007676.1	3.0603	2.33E-107	4.62E-106	Chymotrypsin-C; similar with CTRC_HUMAN;
Px002861.1	3.0431	0.00E+00	0.00E+00	Trypsin, alkaline C; similar with TRYC_MANSE;
Px009639.1	3.0376	0.00E+00	0.00E+00	Trypsin CFT-1; similar with TRYP_CHOFU;
Px007903.1	2.9954	0.00E+00	0.00E+00	Collagenase; similar with COGS_HYPLI;
Px005244.1	2.9796	7.91E-29	6.74E-28	Trypsin CFT-1; similar with TRYP_CHOFU;
Px015049.1	2.9693	5.52E-35	5.22E-34	Venom dipeptidyl peptidase 4;
Px004698.1	2.9517	1.27E-16	7.77E-16	NADH dehydrogenase [ubiquinone] iron-sulfur protein 8, mitochondrial;
Px005724.3	2.9382	3.59E-86	6.08E-85	Xaa-Pro aminopeptidase 1; similar with XPP1_MOUSE;
Px003660.1	2.9352	2.46E-11	1.20E-10	NADH dehydrogenase [ubiquinone] iron-sulfur protein 7, mitochondrial;
Px008281.1	2.9352	2.46E-11	1.20E-10	Kinesin-like protein KIF12; similar with KIF12_MOUSE;
Px003479.1	2.9314	1.50E-17	9.44E-17	Protein rhomboid; similar with RHOM_DROME;
Px011683.2	2.9306	0.00E+00	0.00E+00	Venom carboxylesterase-6; similar with EST6_APIME;
Px002784.1	2.9253	1.81E-07	6.86E-07	Multidrug resistance-associated protein 7;
Px015280.1	2.9138	7.05E-10	3.15E-09	Trypsin CFT-1; similar with TRYP_CHOFU;
Px009987.1	2.8978	1.47E-38	1.48E-37	Caspase-1; similar with CASP1_DROME;
Px006570.1	2.8972	0.00E+00	0.00E+00	Trypsin, alkaline B; similar with TRYB_MANSE;
Px000616.1	2.8942	0.00E+00	0.00E+00	Tubulin alpha chain; similar with TBA_TORMA;
Px000994.1	2.8631	0.00E+00	0.00E+00	Zinc carboxypeptidase A 1; similar with CBPA1_DROPS;
Px009638.1	2.8523	0.00E+00	0.00E+00	Trypsin, alkaline B; similar with TRYB_MANSE;

Px002141.1	2.8381	6.72E-42	7.09E-41	Probable phospholipid-transporting ATPase IF;
Px008030.1	2.8370	3.71E-26	2.97E-25	cAMP-dependent protein kinase catalytic subunit;
Px012332.1	2.8227	3.03E-20	2.09E-19	ATP synthase subunit beta, mitochondrial;
Px011608.1	2.8142	1.67E-04	4.72E-04	Fanconi-associated nuclease 1; similar with FAN1_DANRE;
Px016752.1	2.8120	1.98E-36	1.92E-35	Glutamate dehydrogenase 2, mitochondrial;
Px013778.1	2.8072	0.00E+00	0.00E+00	Chitin synthase 3; similar with CHS3_USTMA;
Px002587.1	2.7903	0.00E+00	0.00E+00	Trypsin CFT-1; similar with TRYP_CHOFU;
Px011553.1	2.7787	9.89E-243	3.55E-241	cGMP-dependent protein kinase, isozyme 1;
Px002993.1	2.7717	0.00E+00	0.00E+00	Zinc metalloproteinase nas-4; similar with NAS4_CAEEL;
Px015388.1	2.7618	1.35E-08	5.50E-08	Nose resistant to fluoxetine protein 6;
Px005373.1	2.7565	1.52E-39	1.56E-38	Carboxypeptidase B; similar with CBPB_ASTFL;
Px002047.1	2.7527	1.74E-261	6.60E-260	Uncharacterized family 31 glucosidase KIAA1161;
Px016235.1	2.7497	1.52E-09	6.70E-09	3-oxoacyl-[acyl-carrier-protein] reductase FabG;
Px002926.1	2.7459	2.44E-55	3.05E-54	Choline dehydrogenase, mitochondrial;
Px016263.3	2.7402	1.48E-13	7.96E-13	ATP-binding cassette sub-family G member 4;
Px000107.1	2.7335	0.00E+00	0.00E+00	Trypsin, alkaline C; similar with TRYC_MANSE;
Px001079.1	2.7320	0.00E+00	0.00E+00	Unknown function
Px007618.1	2.7222	1.13E-272	4.41E-271	Trypsin CFT-1; similar with TRYP_CHOFU;
Px011887.1	2.7196	0.00E+00	0.00E+00	Trypsin, alkaline C; similar with TRYC_MANSE;
Px002980.1	2.7147	3.10E-10	1.42E-09	Putative deoxyribose-phosphate aldolase;
Px009286.1	2.7141	0.00E+00	0.00E+00	Trypsin, alkaline C; similar with TRYC_MANSE;
Px002274.1	2.7121	0.00E+00	0.00E+00	Phenoloxidase subunit 2; similar with PRP2_BOMMO;
Px013288.1	2.7099	5.83E-272	2.27E-270	Tubulin beta chain; similar with TBB_PARLI;
Px005936.2	2.7085	3.12E-86	5.29E-85	Trypsin, alkaline C; similar with TRYC_MANSE;
Px013968.1	2.6816	1.66E-71	2.47E-70	Dual specificity protein phosphatase 19;
Px006228.1	2.6731	3.87E-22	2.80E-21	Beta-1,3-galactosyltransferase 5;
Px002865.1	2.6726	0.00E+00	0.00E+00	Trypsin 5G1; similar with TRY5_AEDAE;
Px009784.1	2.6561	0.00E+00	0.00E+00	Ribosome-binding protein 1;
Px000119.2	2.6517	0.00E+00	0.00E+00	Inositol hexakisphosphate and diphosphoinositol-pentakisphosphate kinase;
Px016567.1	2.6458	1.14E-175	3.25E-174	Matrix metalloproteinase-14;
Px004610.1	2.6440	7.63E-86	1.29E-84	Sphingosine-1-phosphate lyase;
Px006940.2	2.6353	0.00E+00	0.00E+00	Cytosolic beta-glucosidase; similar with GBA3_CAVPO;
Px002441.1	2.6127	4.36E-75	6.75E-74	Trypsin, alkaline B; similar with TRYB_MANSE;
Px018074.1	2.6056	3.91E-24	2.97E-23	Chymotrypsin-like elastase family member 2A;
Px000395.1	2.6001	0.00E+00	0.00E+00	Alpha-amylase 4N; similar with AM4N_DROAN;
Px006728.1	2.5849	0.00E+00	0.00E+00	Sucrose-6-phosphate hydrolase;
Px006600.1	2.5735	7.58E-284	3.05E-282	Acetylcholinesterase; similar with ACES_BOVIN;
Px011889.1	2.5691	1.52E-21	1.09E-20	Achelase-1; similar with ACH1_LONAC;
Px017228.1	2.5584	2.52E-11	1.23E-10	Facilitated trehalose transporter Tret1;
Px011359.1	2.5541	3.85E-26	3.07E-25	Dynein light chain roadblock-type 1;
Px009757.1	2.5486	2.19E-10	1.01E-09	Proteasome subunit beta type-2; similar with PSB2_MOUSE;
Px006305.1	2.5477	4.59E-69	6.67E-68	E3 ubiquitin-protein ligase SINA-like 2;
Px011283.1	2.5473	9.94E-62	1.34E-60	Alpha N-terminal protein methyltransferase 1A;
Px005866.1	2.5435	0.00E+00	0.00E+00	Zinc carboxypeptidase A 1;
Px005277.3	2.5372	0.00E+00	0.00E+00	Lactase-phlorizin hydrolase; similar with LPH_HUMAN;
Px001977.1	2.5367	1.82E-04	5.08E-04	Tyrosine-protein phosphatase 99A;
Px003486.1	2.5359	0.00E+00	0.00E+00	Probable maltase H; similar with MAL2_DROME;
Px005199.1	2.5327	1.44E-159	3.81E-158	Long-chain fatty acid transport protein 4;
Px014883.1	2.5298	7.38E-32	6.64E-31	Ecdysteroid UDP-glucosyltransferase;
Px005820.1	2.5294	1.10E-144	2.70E-143	Glutaminyl-peptide cyclotransferase;
Px014887.1	2.5258	3.34E-136	7.70E-135	Dihydropteridine reductase; similar with DHPR_RAT;
Px007674.2	2.5239	0.00E+00	0.00E+00	Collagenase; similar with COGS_HYPLI;
Px007675.1	2.5233	0.00E+00	0.00E+00	Collagenase; similar with COGS_HYPLI;
Px004489.1	2.5013	7.47E-48	8.61E-47	Adenosine deaminase CECR1;

Px009766.1	2.4923	6.69E-06	2.20E-05	Lysosomal Pro-X carboxypeptidase;
Px002046.1	2.4907	0.00E+00	0.00E+00	Uncharacterized family 31 glucosidase KIAA1161;
Px007677.1	2.4787	0.00E+00	0.00E+00	Trypsin-7; similar with TRY7_ANOGA;
Px013884.1	2.4758	5.37E-18	3.43E-17	Probable cytochrome P450 9f2;
Px012582.1	2.4724	2.13E-127	4.68E-126	Collagenase; similar with COGS_HYPLI;
Px007691.1	2.4696	3.21E-04	8.67E-04	Polypeptide N-acetylgalactosaminyltransferase 1;
Px004163.1	2.4696	2.57E-07	9.61E-07	Ubiquitin carboxyl-terminal hydrolase 8;
Px015266.1	2.4663	4.55E-17	2.83E-16	Glutathione S-transferase omega-1;
Px001785.1	2.4629	2.00E-24	1.53E-23	Gamma-glutamyltranspeptidase 1; similar with GGT1_PIG;
Px010022.2	2.4600	2.32E-49	2.71E-48	Cytosolic beta-glucosidase; similar with GBA3_CAVPO;
Px014181.2	2.4517	2.43E-111	4.92E-110	Aldose 1-epimerase; similar with GALM_PIG;
Px008848.1	2.4433	2.05E-168	5.68E-167	Myrosinase 1; similar with MYRO1_BREBR;
Px014981.1	2.4259	0.00E+00	0.00E+00	Cytochrome P450 9e2; similar with CP9E2_BLAGE;
Px011894.1	2.4252	3.52E-09	1.50E-08	Fatty-acid amide hydrolase 2; similar with FAAH2_HUMAN;
Px016058.1	2.4185	0.00E+00	0.00E+00	Trypsin, alkaline C; similar with TRYC_MANSE;
Px007814.1	2.4181	0.00E+00	0.00E+00	Multiple inositol polyphosphate phosphatase 1;
Px002588.1	2.4151	4.81E-14	2.66E-13	Trypsin, alkaline C; similar with TRYC_MANSE;
Px002118.1	2.4143	0.00E+00	0.00E+00	Alpha,alpha-trehalose-phosphate synthase [UDP-forming] A;
Px010489.1	2.4085	6.89E-66	9.73E-65	Trypsin, alkaline C; similar with TRYC_MANSE;
Px002416.1	2.4045	6.85E-76	1.07E-74	Probable multidrug resistance-associated protein lethal;
Px009287.1	2.4027	1.27E-279	5.09E-278	Trypsin CFT-1; similar with TRYP_CHOFU;
Px000111.1	2.3992	3.43E-15	1.98E-14	Unknown function
Px001189.1	2.3992	3.96E-06	1.33E-05	Phospholipase D3; similar with PLD3_XENLA;
Px006226.1	2.3992	1.06E-04	3.06E-04	Calpain-C; similar with CANC_DROME;
Px003467.1	2.3876	2.10E-18	1.36E-17	Matrix metalloproteinase-14; similar with MMP14_RABIT;
Px009395.1	2.3871	1.03E-17	6.47E-17	Proclotting enzyme; similar with PCE_TACTR;
Px003761.3	2.3858	4.84E-205	1.52E-203	Endoribonuclease Dicer; similar with DICER_BOVIN;
Px007764.1	2.3822	1.77E-173	4.99E-172	Cytochrome c oxidase subunit 6A1, mitochondrial;
Px007616.1	2.3746	1.09E-158	2.87E-157	Trypsin CFT-1; similar with TRYP_CHOFU;
Px013390.1	2.3727	1.05E-08	4.33E-08	NADH dehydrogenase [ubiquinone] iron-sulfur protein 3, mitochondrial;
Px002209.3	2.3727	4.22E-16	2.54E-15	Dynein heavy chain 1, axonemal; similar with DYH1_RAT;
Px001708.1	2.3708	0.00E+00	0.00E+00	Aminopeptidase N; similar with AMPN_PLUXY;
Px005242.1	2.3668	2.59E-19	1.73E-18	Trypsin CFT-1; similar with TRYP_CHOFU;
Px004327.1	2.3658	1.41E-24	1.08E-23	Glyceraldehyde-3-phosphate dehydrogenase 2;
Px004752.1	2.3527	0.00E+00	0.00E+00	Pancreatic lipase-related protein 2;
Px003335.1	2.3461	5.65E-140	1.34E-138	Testis-specific serine/threonine-protein kinase 1;
Px002589.1	2.3435	3.45E-96	6.29E-95	Trypsin, alkaline A; similar with TRYA_MANSE;
Px004218.1	2.3409	0.00E+00	0.00E+00	Sucrose-6-phosphate hydrolase;
Px015275.1	2.3286	0.00E+00	0.00E+00	Trypsin CFT-1; similar with TRYP_CHOFU;
Px001761.1	2.3229	1.01E-228	3.51E-227	Sucrose-6-phosphate hydrolase;
Px015898.1	2.3215	0.00E+00	0.00E+00	Putative farnesoic acid O-methyl transferase;
Px001705.1	2.3213	0.00E+00	0.00E+00	Membrane alanyl aminopeptidase;
Px005876.1	2.3107	8.18E-70	1.20E-68	Patatin-like phospholipase domain-containing protein 2;
Px007688.3	2.3038	8.95E-17	5.49E-16	Molybdenum cofactor sulfurase;
Px015631.1	2.2963	1.88E-38	1.89E-37	Glutathione S-transferase 1; similar with GSTT1_MUSDO;
Px009642.2	2.2874	7.41E-37	7.24E-36	Unknown function
Px011160.1	2.2730	0.00E+00	0.00E+00	Lactase-phlorizin hydrolase; similar with LPH_HUMAN;
Px006622.3	2.2728	9.45E-20	6.46E-19	Ovochymase-1; similar with OVCH1_HUMAN;
Px007331.1	2.2718	1.93E-58	2.49E-57	Ovarian-specific serine/threonine-protein kinase Lok;
Px001275.1	2.2595	6.17E-09	2.59E-08	Probable multidrug resistance-associated protein lethal;
Px002795.1	2.2574	1.02E-55	1.28E-54	Laccase-4; similar with LAC4_TRAVE;
Px009567.1	2.2552	3.00E-104	5.83E-103	Trypsin, alkaline B; similar with TRYB_MANSE;
Px005211.1	2.2472	4.52E-07	1.65E-06	Tyrocidine synthase 3; similar with TYCC_BREPA;
Px008810.1	2.2431	1.22E-23	9.18E-23	Probable tubulin polyglutamylase TTLL1;

Px002576.1	2.2413	7.31E-79	1.17E-77	Leucine-rich repeats and immunoglobulin-like domains protein 2;
Px007360.3	2.2294	2.18E-223	7.44E-222	3-phosphoinositide-dependent protein kinase 1;
Px001990.2	2.2289	2.92E-104	5.69E-103	Carboxypeptidase B; similar with CBPB_ASTFL;
Px009566.1	2.2251	1.29E-259	4.84E-258	Trypsin, alkaline C; similar with TRYC_MANSE;
Px006265.1	2.2241	3.46E-05	1.06E-04	Alpha-tocopherol transfer protein-like;
Px001633.1	2.2212	3.10E-80	5.00E-79	Angiotensin-converting enzyme;
Px007927.1	2.2154	4.79E-60	6.34E-59	V-type proton ATPase subunit G;
Px010612.1	2.1991	5.97E-76	9.32E-75	Serine/arginine repetitive matrix protein 5;
Px008913.1	2.1926	0.00E+00	0.00E+00	Acetylcholinesterase; similar with ACES_CULPI;
Px000771.1	2.1857	1.04E-117	2.18E-116	Trypsin CFT-1; similar with TRYP_CHOFU;
Px001878.1	2.1781	2.23E-21	1.59E-20	Lysosomal Pro-X carboxypeptidase;
Px004946.1	2.1753	3.57E-18	2.30E-17	ATP synthase subunit gamma, mitochondrial;
Px011886.1	2.1750	1.89E-125	4.14E-124	Trypsin CFT-1; similar with TRYP_CHOFU;
Px006319.1	2.1734	2.37E-82	3.89E-81	Retinol dehydrogenase 11; similar with RDH11_HUMAN;
Px018023.1	2.1708	1.08E-51	1.30E-50	Adenylate kinase isoenzyme 1;
Px016488.1	2.1647	1.18E-09	5.22E-09	Glucose dehydrogenase [acceptor];
Px010816.1	2.1582	5.02E-08	1.97E-07	E3 ubiquitin-protein ligase MARCH2; ;
Px008152.2	2.1559	5.44E-31	4.83E-30	Alpha-1,6-mannosyl-glycoprotein 2-beta-N-acetylglucosaminyltransferase;
Px002652.1	2.1520	2.76E-25	2.16E-24	Aryl hydrocarbon receptor; similar with AHR_MUSSP;
Px010079.1	2.1426	1.30E-95	2.36E-94	Glutamine synthetase 1, mitochondrial;
Px005902.4	2.1318	2.80E-119	5.89E-118	Cytochrome P450 6B6; similar with CP6B6_HELAM;
Px003273.1	2.1129	6.40E-16	3.82E-15	Pancreatic lipase-related protein 2;
Px009347.1	2.1107	3.40E-32	3.07E-31	Protein shuttle craft; similar with STC_DROME;
Px008811.1	2.1078	3.17E-18	2.04E-17	Probable tubulin polyglutamylase TTLL1;
Px004854.1	2.1011	1.06E-15	6.27E-15	Ecdysteroid UDP-glucosyltransferase;
Px011567.1	2.0952	7.54E-11	3.57E-10	Glucose dehydrogenase [acceptor];
Px011501.1	2.0881	7.26E-17	4.47E-16	ATP synthase subunit beta, mitochondrial;
Px010067.1	2.0773	6.13E-06	2.03E-05	Glutamine-rich protein 2; similar with E2A5X5_9HYME;
Px002188.1	2.0773	1.67E-04	4.71E-04	Optomotor-blind protein; similar with OMB_DROME;
Px011796.5	2.0740	1.05E-51	1.26E-50	Putative aminopeptidase W07G4.4;
Px009703.1	2.0631	0.00E+00	0.00E+00	Beta-1,3-glucan-binding protein;
Px004200.1	2.0610	1.11E-81	1.81E-80	Putative phosphatidate phosphatase;
Px016550.6	2.0608	2.43E-11	1.19E-10	Probable L-asparaginase GA20639;
Px001833.1	2.0603	0.00E+00	0.00E+00	Serine protease easter; similar with EAST_DROME;
Px002382.1	2.0597	1.62E-130	3.62E-129	Myosin-VIIa; similar with MYO7A_AEDAE;
Px006500.1	2.0492	9.92E-42	1.05E-40	Peroxisomal acyl-coenzyme A oxidase 3;
Px007402.1	2.0481	1.63E-12	8.38E-12	Dephospho-CoA kinase domain-containing protein;
Px002632.1	2.0480	2.47E-165	6.70E-164	Xaa-Pro dipeptidase; similar with PEPD_HUMAN;
Px011307.1	2.0469	2.97E-33	2.74E-32	Poly(A) polymerase gamma;
Px004167.1	2.0469	1.15E-06	4.06E-06	Plasma kallikrein; similar with KLKB1_MOUSE;
Px006763.1	2.0407	1.02E-05	3.30E-05	Peroxisomal acyl-coenzyme A oxidase 3;
Px016844.1	2.0366	3.06E-05	9.40E-05	Proteasome subunit beta type-1; similar with PSB1_RAT;
Px002985.1	2.0315	9.20E-05	2.68E-04	Uncharacterized protein YJR142W; similar with YJ9J_YEAST;
Px011034.1	2.0305	3.34E-62	4.54E-61	Lysosomal alpha-glucosidase; similar with LYAG_PONAB;
Px011280.1	2.0291	2.23E-60	2.97E-59	4-coumarate--CoA ligase 1; similar with 4CL1_TOBAC;
Px012569.1	2.0285	0.00E+00	0.00E+00	Trypsin, alkaline C; similar with TRYC_MANSE;
Px010481.1	2.0283	7.38E-08	2.86E-07	Tctex1 domain-containing protein 3;
Px015936.1	2.0166	0.00E+00	0.00E+00	Putative uncharacterized protein;
Px009283.1	2.0101	5.66E-06	1.88E-05	Decaprenyl-diphosphate synthase subunit 1;
Px004726.1	2.0010	1.03E-29	8.97E-29	Alpha,alpha-trehalose-phosphate synthase [UDP-forming] 56 kDa subunit;
Px005079.1	1.9962	0.00E+00	0.00E+00	Lactase-phlorizin hydrolase; similar with LPH_HUMAN;
Px001161.1	1.9925	6.56E-100	1.24E-98	Superoxide dismutase [Cu-Zn];
Px013789.1	1.9877	3.65E-89	6.34E-88	L-threonine 3-dehydrogenase;
Px012400.1	1.9673	6.21E-38	6.19E-37	Argininosuccinate lyase; similar with ARLY2_ANAPL;

Px012570.1	1.9654	0.00E+00	0.00E+00	Trypsin CFT-1; similar with TRYP_CHOFU;
Px006403.1	1.9489	1.14E-19	7.74E-19	Prostaglandin reductase 1; similar with PTGR1_BOVIN;
Px003725.1	1.9489	6.60E-74	1.01E-72	Interleukin-1 receptor-associated kinase 4;
Px011097.1	1.9460	2.65E-10	1.22E-09	Serine proteinase stubble; similar with STUB_DROME;
Px001111.1	1.9440	1.54E-14	8.66E-14	Probable multidrug resistance-associated protein lethal;
Px000116.1	1.9398	7.72E-10	3.45E-09	Protein phosphatase 1E; similar with PPM1E_RAT;
Px007071.1	1.9352	2.52E-04	6.90E-04	Histone-lysine N-methyltransferase Suv4-20;
Px006941.1	1.9351	4.59E-121	9.83E-120	Myrosinase 1; similar with MYRO1_BREBR;
Px008153.1	1.9344	1.29E-24	9.93E-24	Alpha-1,6-mannosyl-glycoprotein 2-beta-N-acetylglucosaminyltransferase;
Px002889.1	1.9297	3.44E-08	1.36E-07	Dynein beta chain, ciliary; similar with DYHC_TRIGR;
Px004751.1	1.9293	0.00E+00	0.00E+00	Pancreatic triacylglycerol lipase;
Px002827.2	1.9253	4.57E-05	1.38E-04	Leucine-rich repeat serine/threonine-protein kinase 1;
Px015380.1	1.9245	1.43E-39	1.47E-38	Acyl-CoA Delta(11) desaturase; similar with ACO11_TRINI;
Px003754.1	1.9228	1.67E-147	4.13E-146	Membrane alanyl aminopeptidase;
Px017171.1	1.9108	8.59E-16	5.10E-15	Putative inositol monophosphatase 3;
Px014216.1	1.9094	1.32E-40	1.38E-39	Cytochrome P450 6B6; similar with CP6B6_HELAM;
Px012937.2	1.8846	1.37E-05	4.37E-05	Phospholipase D1; similar with PLD1_HUMAN;
Px006956.1	1.8788	1.02E-112	2.08E-111	Xaa-Pro dipeptidase; similar with PEPD_HUMAN;
Px000872.2	1.8756	1.82E-09	7.93E-09	Ecdysteroid UDP-glucosyltransferase;
Px000394.1	1.8666	1.72E-210	5.53E-209	Alpha-amylase 4N; similar with AM4N_DROAN;
Px002866.1	1.8658	5.28E-09	2.23E-08	Trypsin, alkaline A; similar with TRYA_MANSE;
Px008406.1	1.8639	2.77E-08	1.10E-07	Serine proteinase stubble; similar with STUB_DROME;
Px004577.1	1.8549	4.19E-18	2.69E-17	Phosphotriesterase-related protein;
Px007992.2	1.8540	1.63E-24	1.25E-23	Multidrug resistance protein homolog 49;
Px013665.3	1.8475	2.46E-37	2.43E-36	Collagenase; similar with COGS_HYPLI;
Px016835.1	1.8438	7.68E-29	6.55E-28	Inositol polyphosphate multikinase;
Px004611.1	1.8428	1.21E-04	3.46E-04	Katanin p60 ATPase-containing subunit A-like 1;
Px009920.1	1.8428	8.21E-15	4.67E-14	Branched-chain-amino-acid aminotransferase, mitochondrial;
Px005763.1	1.8391	4.70E-09	1.99E-08	Fatty-acid amide hydrolase 2;
Px005124.5	1.8306	2.07E-24	1.58E-23	Transposon Ty3-I Gag-Pol polyprotein;
Px000647.3	1.8232	6.21E-280	2.49E-278	UDP-glucuronosyltransferase 2B7;
Px010831.1	1.8205	7.51E-09	3.13E-08	Luciferin 4-monooxygenase; similar with LUCI_PHOPE;
Px017172.1	1.8142	5.10E-12	2.56E-11	Serine palmitoyltransferase 1;
Px002968.1	1.8131	5.34E-41	5.58E-40	Adenylate kinase isoenzyme 1;
Px007409.1	1.8110	8.48E-30	7.38E-29	Succinate dehydrogenase [ubiquinone] iron-sulfur subunit, mitochondrial;
Px009194.1	1.8005	8.55E-179	2.46E-177	Dihydropteridine reductase;
Px016270.1	1.7997	7.53E-11	3.57E-10	Histone deacetylase 3; similar with HDAC3_TETNG;
Px015779.1	1.7984	0.00E+00	0.00E+00	Fatty-acid amide hydrolase 2;
Px008457.1	1.7972	1.09E-06	3.86E-06	Carbohydrate sulfotransferase 13;
Px003540.1	1.7898	3.78E-38	3.78E-37	Hexokinase type 2; similar with HXK2_DROME;
Px013655.1	1.7878	3.45E-08	1.37E-07	Chitotriosidase-1; similar with CHIT1_HUMAN;
Px004684.1	1.7858	0.00E+00	0.00E+00	Tyrosine 3-monooxygenase; similar with TY3H_DROME;
Px014984.1	1.7851	2.81E-15	1.63E-14	Cytochrome P450; similar with B5TVL6_PLUXY;
Px011697.1	1.7841	4.24E-28	3.56E-27	Bifunctional methylenetetrahydrofolate dehydrogenase/cyclohydrolase;
Px008505.1	1.7699	2.97E-27	2.45E-26	Glucose dehydrogenase [acceptor];
Px012329.1	1.7693	5.39E-84	8.95E-83	Serine protease persephone; similar with PSH_DROME;
Px002295.1	1.7684	0.00E+00	0.00E+00	Pancreatic lipase-related protein 2;
Px015778.1	1.7676	5.49E-08	2.14E-07	Sentrin-specific protease 8; similar with SENP8_HUMAN;
Px012643.1	1.7650	5.67E-95	1.02E-93	Angiotensin-converting enzyme;
Px015449.1	1.7626	1.93E-12	9.88E-12	Acetyl-CoA carboxylase 1; similar with ACACA_SHEEP;
Px000123.1	1.7598	0.00E+00	0.00E+00	Putative aldehyde dehydrogenase family 7 member A1 homolog;
Px003753.1	1.7587	0.00E+00	0.00E+00	Membrane alanyl aminopeptidase;
Px004725.1	1.7553	9.24E-06	3.01E-05	ATP-binding cassette sub-family G member 1;
Px017680.3	1.7524	1.24E-28	1.06E-27	CaiB/baiF CoA-transferase family protein C7orf10 homolog;

Px008938.1	1.7508	1.35E-30	1.19E-29	Carboxypeptidase D; similar with CBPD_DROME;
Px014955.1	1.7471	5.65E-27	4.64E-26	ABC transporter G family member 23;
Px011231.1	1.7453	1.76E-27	1.46E-26	Protein unc-50 homolog; similar with UNC50_DROME;
Px014078.1	1.7425	5.92E-30	5.19E-29	Fructose-bisphosphate aldolase; similar with ALF_DROME;
Px010017.1	1.7418	0.00E+00	0.00E+00	Carboxypeptidase B; similar with CBPB_ASTFL;
Px009015.1	1.7327	5.76E-172	1.61E-170	Testis-specific serine/threonine-protein kinase 4;
Px000957.1	1.7245	2.09E-58	2.70E-57	Putative lipoyltransferase 2, mitochondrial;
Px009343.1	1.7147	2.39E-06	8.20E-06	NADH-ubiquinone oxidoreductase 75 kDa subunit, mitochondrial;
Px000844.1	1.7136	3.72E-14	2.07E-13	Ribosomal protein S6 kinase delta-1;
Px011320.1	1.7053	2.69E-04	7.34E-04	Cytochrome b-c1 complex subunit Rieske, mitochondrial;
Px015730.1	1.7021	0.00E+00	0.00E+00	Apolipophorins; similar with APLP_MANSE;
Px004446.3	1.6970	2.01E-45	2.23E-44	Copper-transporting ATPase 2; similar with ATP7B_MOUSE;
Px005689.1	1.6962	5.99E-09	2.52E-08	Peptidylglycine alpha-hydroxylating monooxygenase;
Px005403.1	1.6902	6.87E-06	2.26E-05	Probable multidrug resistance-associated protein lethal;
Px001113.1	1.6873	1.59E-11	7.83E-11	Ecdysteroid UDP-glucosyltransferase;
Px000402.2	1.6821	5.77E-83	9.50E-82	Serine protease persephone; similar with PSH_DROME;
Px008154.1	1.6773	3.06E-26	2.46E-25	Alpha-1,6-mannosyl-glycoprotein 2-beta-N-acetylglucosaminyltransferase;
Px007305.1	1.6562	9.37E-42	9.88E-41	C-1-tetrahydrofolate synthase, cytoplasmic;
Px003771.1	1.6556	6.82E-20	4.67E-19	ATP synthase subunit s, mitochondrial;
Px004798.2	1.6515	1.57E-68	2.27E-67	Malate dehydrogenase, mitochondrial;
Px003712.1	1.6490	1.05E-19	7.17E-19	Acetylcholinesterase; similar with ACES_CULPI;
Px006502.1	1.6424	1.34E-55	1.68E-54	Calpain-B; similar with CANB_DROME;
Px005342.1	1.6408	3.80E-156	9.82E-155	Collagenase; similar with COGS_HYPLI;
Px009087.2	1.6281	1.83E-240	6.53E-239	Coagulation factor XII; similar with FA12_CAVPO;
Px008070.1	1.6216	3.59E-04	9.63E-04	Peroxisomal leader peptide-processing protease;
Px004065.1	1.6192	1.53E-09	6.71E-09	Mannosyl-oligosaccharide alpha-1,2-mannosidase isoform A;
Px007904.1	1.6124	0.00E+00	0.00E+00	Collagenase; similar with COGS_HYPLI;
Px002015.1	1.6107	7.78E-06	2.55E-05	AGAP008123-PA; similar with Q7Q401_ANOGA;
Px016278.1	1.6052	1.76E-11	8.64E-11	Cytoplasmic aconitate hydratase;
Px007111.3	1.6050	2.44E-116	5.04E-115	ER degradation-enhancing alpha-mannosidase-like 2;
Px002919.1	1.6022	2.42E-22	1.76E-21	Putative N-acetylglucosamine-6-phosphate deacetylase;
Px009715.1	1.5988	8.85E-16	5.24E-15	Mitochondrial inner membrane protease subunit 1;
Px013783.3	1.5951	1.97E-06	6.85E-06	Methyltransferase-like protein 14 homolog;
Px004643.1	1.5882	6.58E-06	2.17E-05	Cytochrome P450 6B6; similar with CP6B6_HELAM;
Px000132.1	1.5866	3.04E-79	4.87E-78	FGGY carbohydrate kinase domain-containing protein;
Px011340.1	1.5860	0.00E+00	0.00E+00	Non-specific lipid-transfer protein;
Px005490.1	1.5819	5.31E-62	7.19E-61	Inhibitor of nuclear factor kappa-B kinase subunit beta;
Px016731.1	1.5768	2.81E-27	2.33E-26	Acyl-CoA synthetase family member 3, mitochondrial;
Px015306.1	1.5761	1.08E-11	5.34E-11	1,2-dihydroxy-3-keto-5-methylthiopentene dioxygenase;
Px013162.1	1.5740	7.41E-151	1.88E-149	Serine protease snake; similar with SNAK_DROME;
Px017275.1	1.5732	4.40E-10	1.99E-09	Inositol monophosphatase 3;
Px006124.1	1.5716	2.01E-24	1.53E-23	Tubulin alpha-2/alpha-4 chain;
Px001531.1	1.5708	7.84E-60	1.04E-58	Probable peroxisomal acyl-coenzyme A oxidase 1;
Px016564.1	1.5653	3.09E-27	2.55E-26	Hydroxyacyl-coenzyme A dehydrogenase, mitochondrial;
Px002826.2	1.5627	1.01E-05	3.28E-05	CTD small phosphatase-like protein 2;
Px008540.1	1.5543	2.19E-09	9.49E-09	Unknown function
Px005340.1	1.5499	1.24E-139	2.94E-138	Collagenase; similar with COGS_HYPLI;
Px012743.1	1.5433	6.47E-14	3.55E-13	Probable dolichol-phosphate mannosyltransferase;
Px014172.1	1.5367	3.01E-07	1.11E-06	Heparanase; similar with HPSE_BOVIN;
Px010498.1	1.5315	2.77E-59	3.62E-58	Fumarylacetoacetate hydrolase domain-containing protein 2
Px001106.1	1.5290	1.97E-08	7.95E-08	Protein retinal degeneration B;
Px010148.3	1.5270	0.00E+00	0.00E+00	Putative uncharacterized protein;
Px003277.1	1.5220	5.21E-05	1.56E-04	Dual specificity tyrosine-phosphorylation-regulated kinase 2;
Px013958.1	1.5211	6.83E-145	1.68E-143	Cytochrome P450 6k1; similar with CP6K1_BLAGE;

Px007047.2	1.5134	9.55E-05	2.77E-04	Probable peroxisomal acyl-coenzyme A oxidase 1;
Px016262.1	1.5084	3.45E-19	2.30E-18	Cysteine sulfinic acid decarboxylase;
Px008849.1	1.5018	7.18E-50	8.45E-49	Myrosinase 1; similar with MYRO1_BREBR;
Px003229.1	1.5011	1.66E-09	7.28E-09	Dynein heavy chain 5, axonemal;
Px005265.1	1.4972	1.16E-08	4.78E-08	Ubiquitin carboxyl-terminal hydrolase 6;
Px006995.1	1.4923	7.94E-05	2.33E-04	Polypeptide N-acetylgalactosaminyltransferase 1;
Px016677.1	1.4862	1.49E-136	3.44E-135	ATP-binding cassette sub-family G member 1;
Px004945.1	1.4848	1.39E-157	3.62E-156	Protein henna; similar with PH4H_DROME;
Px011686.1	1.4813	2.69E-20	1.86E-19	5-formyltetrahydrofolate cyclo-ligase;
Px013984.1	1.4786	1.70E-11	8.37E-11	Dynein heavy chain 8, axonemal; s
Px003016.1	1.4735	1.08E-46	1.22E-45	Tyrosine-protein kinase HTK16;
Px013714.1	1.4732	6.59E-05	1.95E-04	Putative serine/threonine-protein kinase C05D10.2;
Px002706.1	1.4721	0.00E+00	0.00E+00	Lysozyme; similar with LYS_TRINI;
Px017858.1	1.4719	1.07E-34	1.01E-33	ATP-binding cassette sub-family G member 1;
Px011489.1	1.4705	1.48E-69	2.16E-68	Gamma-glutamyl hydrolase A;
Px005954.1	1.4684	8.08E-13	4.22E-12	UDP-glucose:glycoprotein glucosyltransferase;
Px011338.1	1.4635	5.55E-12	2.79E-11	GTP cyclohydrolase 1; similar with GCH1_DROME;
Px014982.1	1.4635	0.00E+00	0.00E+00	Probable cytochrome P450 9f2;
Px000965.1	1.4634	3.13E-36	3.03E-35	D-amino-acid oxidase 2;
Px015886.1	1.4619	1.21E-04	3.45E-04	ATP-dependent DNA helicase Q5;
Px013203.1	1.4517	2.86E-06	9.77E-06	UDP-glucuronosyltransferase 2B2;
Px000855.1	1.4469	1.40E-135	3.22E-134	Juvenile hormone epoxide hydrolase;
Px014831.2	1.4467	0.00E+00	0.00E+00	Serine proteinase stubble; similar with STUB_DROME;
Px014586.1	1.4462	1.33E-30	1.17E-29	Tubulointerstitial nephritis antigen;
Px007510.1	1.4398	3.94E-10	1.80E-09	Spectrin beta chain; similar with SPTCB_DROME;
Px001349.1	1.4343	2.79E-14	1.56E-13	Myosin-IIIa; similar with MYO3A_HUMAN;
Px015740.3	1.4224	8.84E-08	3.41E-07	Endoribonuclease Dcr-1; similar with DCR1_DROME;
Px008607.1	1.4201	1.74E-29	1.51E-28	Ras-related protein Rap-2b; similar with RAP2B_RAT;
Px017644.1	1.4197	3.55E-06	1.20E-05	Dynein heavy chain 10, axonemal;
Px013053.1	1.4168	5.05E-81	8.20E-80	Endoribonuclease Dicer; similar with DICER_MOUSE;
Px010627.1	1.4167	1.53E-153	3.89E-152	Wing disc-specific protein;
Px008806.2	1.4150	2.28E-44	2.49E-43	Hydroxyacylglutathione hydrolase, mitochondrial;
Px007577.1	1.4133	8.83E-12	4.40E-11	Protein N-terminal asparagine amidohydrolase;
Px009011.1	1.4129	6.79E-05	2.01E-04	Tyrosine-protein kinase hopscotch;
Px005726.1	1.4124	1.92E-12	9.85E-12	Xaa-Pro aminopeptidase 1; similar with XPP1_HUMAN;
Px016080.1	1.4117	3.08E-05	9.48E-05	Glucosamine-6-phosphate isomerase;
Px011844.1	1.4117	3.08E-05	9.48E-05	Adenylate kinase 7; similar with KAD7_MOUSE;
Px011611.1	1.4113	0.00E+00	0.00E+00	Probable enoyl-CoA hydratase, mitochondrial;
Px016914.1	1.4109	6.83E-63	9.40E-62	Sarcoplasmic calcium-binding protein;
Px008591.1	1.4098	1.56E-10	7.26E-10	Hexosaminidase D; similar with HEXDC_HUMAN;
Px006720.1	1.4070	1.32E-07	5.02E-07	Glycerol-3-phosphate acyltransferase 4;
Px013538.1	1.4047	8.56E-19	5.64E-18	2-oxoglutarate dehydrogenase, mitochondrial;
Px012191.1	1.4028	2.38E-208	7.61E-207	Sodium/potassium-transporting ATPase subunit alpha-1;
Px005747.1	1.4025	6.57E-16	3.92E-15	Proteasome subunit alpha type-5;
Px003398.1	1.3992	2.33E-08	9.33E-08	Probable phospholipid-transporting ATPase IA;
Px009568.1	1.3992	1.23E-04	3.53E-04	Trypsin, alkaline C; similar with TRYC_MANSE;
Px017669.1	1.3964	2.24E-18	1.45E-17	2-hydroxyacyl-CoA lyase 1;
Px001847.1	1.3803	1.09E-180	3.16E-179	Aminoacylase-1; similar with ACY1_MOUSE;
Px005341.1	1.3783	1.92E-45	2.13E-44	Collagenase; similar with COGS_HYPLI;
Px001370.1	1.3780	6.09E-19	4.04E-18	Aminoacylase-1; similar with ACY1_MOUSE;
Px013252.1	1.3766	0.00E+00	0.00E+00	Calexcitin-2; similar with CEX2_CAEEL;
Px004590.1	1.3727	8.33E-05	2.43E-04	Dynein heavy chain 7, axonemal;
Px000087.2	1.3668	5.17E-07	1.88E-06	ATP-binding cassette sub-family G member 4;
Px009062.1	1.3641	3.11E-05	9.55E-05	Probable cytochrome P450 304a1;

Px005329.1	1.3592	4.00E-66	5.66E-65	Protein ETHE1, mitochondrial;
Px012976.1	1.3549	1.30E-32	1.19E-31	Sulfiredoxin-1; similar with SRXN1_MOUSE;
Px009798.1	1.3537	1.57E-40	1.63E-39	Aminoacylase-1A; similar with ACY1A_RAT;
Px000146.1	1.3503	5.61E-05	1.68E-04	Adenylate cyclase type 9; similar with ADCY9_HUMAN;
Px006663.1	1.3501	2.50E-38	2.52E-37	Ribonuclease Oy; similar with RNOY_CRAGI;
Px010161.1	1.3476	3.12E-26	2.50E-25	GTP cyclohydrolase 1; similar with GCH1_DROME;
Px016171.2	1.3471	1.79E-54	2.21E-53	Prolyl endopeptidase; similar with PPCE_MOUSE;
Px012630.1	1.3430	4.20E-41	4.40E-40	Tribbles homolog 2; similar with TRIB2_MOUSE;
Px007369.1	1.3403	4.19E-08	1.65E-07	DNA excision repair protein haywire;
Px009117.1	1.3403	1.32E-09	5.80E-09	Phosphotriesterase-related protein;
Px000763.1	1.3380	2.84E-09	1.22E-08	Acetyl-CoA carboxylase;
Px015923.1	1.3371	5.16E-07	1.88E-06	OTU domain-containing protein 1;
Px012027.3	1.3347	9.75E-93	1.73E-91	Protein 5NUC; similar with 5NTD_LUTLO;
Px008602.1	1.3331	2.87E-11	1.39E-10	RING-box protein 2; similar with RBX2_HUMAN;
Px012523.1	1.3305	6.77E-16	4.04E-15	Ras-related protein Rab-35; similar with RAB35_RAT;
Px001200.1	1.3269	1.44E-72	2.17E-71	Electron transfer flavoprotein-ubiquinone oxidoreductase, mitochondrial;
Px003361.1	1.3252	8.90E-19	5.86E-18	Cysteine protease ATG4D;
Px009776.1	1.3252	2.34E-08	9.36E-08	Mevalonate kinase; similar with KIME_DICDI;
Px007906.2	1.3236	0.00E+00	0.00E+00	Collagenase; similar with COGS_HYPLI;
Px009982.1	1.3211	1.20E-43	1.30E-42	5-oxoprolinase; similar with OPLA_HUMAN;
Px005295.1	1.3205	1.92E-08	7.76E-08	Tyrosine-protein kinase Src64B;
Px003782.1	1.3205	7.56E-110	1.51E-108	Carboxypeptidase E; similar with CBPE_LOPAM;
Px009887.1	1.3111	4.97E-09	2.10E-08	Probable protein phosphatase 2C T23F11.1;
Px016664.1	1.3099	2.71E-18	1.75E-17	DNA replication complex GINS protein PSF1;
Px004571.1	1.3081	2.26E-185	6.68E-184	Aldose reductase; similar with ALDR_BOVIN;
Px003168.1	1.3070	0.00E+00	0.00E+00	Soma ferritin; similar with FRIS_LYMST;
Px006106.1	1.3067	6.05E-183	1.77E-181	Glutathione S-transferase 1;
Px016456.1	1.3048	0.00E+00	0.00E+00	Lambda-crystallin homolog;
Px008217.3	1.3035	8.78E-09	3.64E-08	Phosphatidylinositol 4,5-bisphosphate 5-phosphatase A;
Px000513.1	1.2987	1.37E-31	1.23E-30	Teneurin-2; similar with TEN2_MOUSE;
Px003149.1	1.2980	7.04E-19	4.66E-18	SET domain-containing protein 4; similar with SETD4_MOUSE;
Px016666.1	1.2974	6.21E-14	3.41E-13	Sphingosine-1-phosphate phosphatase 1;
Px011025.3	1.2894	1.82E-122	3.93E-121	Protein yellow; similar with YELL_DROYA;
Px004607.1	1.2880	1.91E-13	1.02E-12	NEDD8-activating enzyme E1 catalytic subunit;
Px016679.1	1.2867	1.13E-05	3.64E-05	ATP-binding cassette transporter subfamily G;
Px015448.1	1.2854	3.01E-05	9.28E-05	Acetyl-CoA carboxylase; similar with ACAC_CHICK;
Px016834.1	1.2833	2.58E-98	4.76E-97	Probable 3-hydroxyacyl-CoA dehydrogenase B0272.3;
Px008337.1	1.2761	1.16E-111	2.35E-110	Leishmanolysin-like peptidase;
Px016733.1	1.2758	1.82E-09	7.92E-09	Acyl-CoA synthetase family member 3, mitochondrial;
Px006848.1	1.2745	1.88E-14	1.05E-13	UDP-glucose 6-dehydrogenase;
Px006311.1	1.2734	4.37E-18	2.80E-17	Chorion peroxidase; similar with PERC_ANOGA;
Px001795.1	1.2730	9.10E-17	5.57E-16	Ribosomal protein S6 kinase alpha-3;
Px002115.1	1.2726	1.76E-36	1.71E-35	5-formyltetrahydrofolate cyclo-ligase;
Px003167.1	1.2675	3.51E-157	9.10E-156	Ferritin subunit; similar with FRI_AEDAE;
Px002715.1	1.2673	4.39E-154	1.12E-152	Cathepsin L; similar with CATL_DROME;
Px013837.1	1.2663	1.45E-04	4.10E-04	Carnitine O-palmitoyltransferase 1, muscle isoform;
Px015426.1	1.2649	4.69E-13	2.48E-12	MAP kinase-activated protein kinase 2;
Px003365.1	1.2639	2.20E-57	2.82E-56	Luciferin 4-monooxygenase; similar with LUCI_PHOPE;
Px000372.1	1.2617	2.59E-11	1.26E-10	Putative gamma-glutamyltransferase ywrD;
Px008334.1	1.2586	3.79E-49	4.42E-48	Putative uncharacterized protein GLEAN_14945;
Px013298.1	1.2577	3.36E-51	4.02E-50	Serine proteinase-like protein;
Px001059.1	1.2567	2.89E-79	4.64E-78	Beta-1,3-glucan-binding protein;
Px002767.1	1.2554	0.00E+00	0.00E+00	Spectrin alpha chain; similar with SPTCA_DROME;
Px010934.1	1.2536	9.95E-36	9.54E-35	Vascular endothelial growth factor receptor 1;

Px013936.1	1.2531	1.39E-28	1.18E-27	3-ketoacyl-CoA thiolase, mitochondrial;
Px003788.1	1.2526	4.81E-57	6.14E-56	D-2-hydroxyglutarate dehydrogenase, mitochondrial;
Px007466.2	1.2506	2.64E-07	9.84E-07	Glucose dehydrogenase [acceptor];
Px010271.1	1.2456	5.38E-14	2.96E-13	2-acylglycerol O-acyltransferase 1;
Px012993.1	1.2368	4.80E-99	8.92E-98	Multifunctional protein ADE2;
Px006054.1	1.2358	6.60E-273	2.59E-271	Myrosinase 1; similar with MYRO1_BREBR;
Px001277.2	1.2325	1.00E-06	3.55E-06	Probable phospholipid-transporting ATPase IIB;
Px012877.1	1.2309	5.17E-17	3.21E-16	Inositol oxygenase; similar with MIOX_DANRE;
Px006295.1	1.2186	2.27E-05	7.07E-05	ATP-dependent RNA helicase DDX18;
Px017253.1	1.2169	4.53E-37	4.47E-36	Cytochrome P450 6B2; similar with CP6B2_HELAM;
Px011707.1	1.2143	2.86E-35	2.71E-34	Amidophosphoribosyltransferase;
Px007055.2	1.2138	3.00E-67	4.29E-66	Branched-chain-amino-acid aminotransferase, cytosolic;
Px011620.2	1.2137	5.39E-29	4.60E-28	Flavin-containing monooxygenase FMO GS-OX3;
Px012198.1	1.2125	2.46E-68	3.54E-67	Fatty acid synthase; similar with FAS_RAT;
Px011286.1	1.2119	8.71E-219	2.89E-217	Glutaryl-CoA dehydrogenase, mitochondrial;
Px008312.1	1.2097	2.46E-04	6.74E-04	Serine/threonine-protein kinase PAK 3;
Px002733.2	1.2093	0.00E+00	0.00E+00	Aldehyde dehydrogenase, dimeric NADP-preferring;
Px002532.1	1.2056	1.34E-124	2.93E-123	Peroxidasin; similar with PXDN_DROME;
Px004810.1	1.2046	7.06E-09	2.95E-08	ADP-ribosylation factor-like protein 2;
Px011015.1	1.1970	2.82E-07	1.05E-06	NAD-dependent deacetylase sirtuin-5;
Px000039.1	1.1965	1.40E-231	4.93E-230	UDP-glucuronosyltransferase 1-8;
Px011499.2	1.1964	1.89E-18	1.23E-17	Serine protease persephone; similar with PSH_DROME;
Px003013.3	1.1926	3.26E-165	8.82E-164	Lipoyltransferase 1, mitochondrial;
Px001933.1	1.1912	1.41E-09	6.20E-09	Myrosinase 1; similar with MYRO1_BREBR;
Px005839.1	1.1906	1.80E-18	1.17E-17	Mannosyl-oligosaccharide alpha-1,2-mannosidase isoform A;
Px008946.1	1.1905	9.69E-78	1.54E-76	Dynein heavy chain 7, axonemal;
Px010887.1	1.1902	3.16E-21	2.24E-20	Phosphoenolpyruvate carboxykinase [GTP];
Px001894.8	1.1880	5.51E-25	4.27E-24	Ethanolamine-phosphate cytidylyltransferase;
Px009877.1	1.1833	6.80E-08	2.64E-07	Ferrochelatase, mitochondrial; similar with HEMH_DROME;
Px000954.1	1.1823	1.17E-74	1.80E-73	Succinyl-CoA ligase [GDP-forming] subunit beta, mitochondrial;
Px005376.1	1.1799	4.53E-08	1.78E-07	Chitotriosidase-1; similar with CHIT1_HUMAN;
Px000499.1	1.1782	3.06E-54	3.76E-53	Inositol hexakisphosphate and diphosphoinositol-pentakisphosphate kinase;
Px008144.2	1.1768	3.02E-08	1.20E-07	Serine/threonine-protein kinase/endoribonuclease ire-1;
Px007318.5	1.1744	5.94E-10	2.67E-09	Serine/threonine-protein kinase 3;
Px001224.1	1.1741	3.07E-05	9.43E-05	Maleylacetoacetate isomerase;
Px001380.1	1.1712	9.90E-20	6.75E-19	Serine/threonine-protein kinase PAK 1;
Px009624.5	1.1652	5.53E-155	1.42E-153	Peroxisomal multifunctional enzyme type 2;
Px012423.1	1.1613	1.21E-06	4.25E-06	TNF receptor-associated factor 6;
Px006643.3	1.1605	8.63E-35	8.15E-34	Luciferin 4-monooxygenase; similar with LUCI_LUCMI;
Px012467.1	1.1592	1.77E-09	7.71E-09	GTP:AMP phosphotransferase, mitochondrial;
Px013246.1	1.1587	3.16E-98	5.83E-97	Dehydrogenase/reductase SDR family member 4;
Px015447.4	1.1577	9.03E-08	3.48E-07	Probable multidrug resistance-associated protein lethal;
Px000720.1	1.1574	2.24E-12	1.15E-11	Galactokinase; similar with GALK1_HUMAN;
Px009700.1	1.1567	6.53E-07	2.36E-06	Phosphotriesterase-related protein;
Px000966.1	1.1548	2.65E-18	1.71E-17	Omega-amidase NIT2; similar with NIT2_BOVIN;
Px009523.1	1.1532	3.53E-05	1.08E-04	N(4)-(Beta-N-acetylglucosaminyl)-L-asparaginase;
Px012044.1	1.1527	1.58E-22	1.16E-21	Acyl-CoA synthetase family member 2, mitochondrial;
Px017788.1	1.1517	2.14E-54	2.63E-53	Serine protease snake; similar with SNAK_DROME;
Px008985.1	1.1502	4.44E-26	3.54E-25	Ubiquitin-conjugating enzyme E2 J1;
Px015896.1	1.1494	3.63E-206	1.14E-204	Glutathione S-transferase 1-1;
Px015683.1	1.1458	8.18E-148	2.04E-146	Protein yellow; similar with YELL_DROYA;
Px010099.1	1.1453	2.76E-19	1.85E-18	Phospholipase D1; similar with PLD1_RAT;
Px016486.3	1.1425	1.12E-86	1.92E-85	Glucose dehydrogenase [acceptor];
Px012019.1	1.1399	6.88E-14	3.77E-13	Protein ariadne-1 homolog; similar with ARI1_HUMAN;

Px001164.1	1.1390	6.83E-117	1.42E-115	UDP-glucuronosyltransferase;
Px007988.1	1.1338	2.66E-16	1.61E-15	WD repeat-containing protein 48 homolog;
Px009675.1	1.1334	1.86E-96	3.40E-95	UDP-glucuronosyltransferase 1-2;
Px015496.1	1.1317	1.05E-11	5.23E-11	Protein-S-isoprenylcysteine O-methyltransferase;
Px013533.1	1.1291	1.77E-09	7.71E-09	Tetratricopeptide repeat protein 30A;
Px010078.3	1.1245	3.16E-85	5.30E-84	Glutathione S-transferase 1; similar with GSTT1_MANSE;
Px016675.1	1.1203	5.63E-11	2.69E-10	ATP-binding cassette sub-family G member 1;
Px015376.1	1.1200	1.05E-146	2.61E-145	Phosphoenolpyruvate carboxykinase [GTP];
Px002044.1	1.1191	1.32E-04	3.76E-04	Phosphatidylinositol 3-kinase catalytic subunit type 3;
Px010436.1	1.1181	3.98E-92	7.03E-91	Venom serine carboxypeptidase;
Px002601.1	1.1167	9.50E-97	1.74E-95	Probable pterin-4-alpha-carbinolamine dehydratase;
Px007883.1	1.1153	4.95E-21	3.49E-20	Probable medium-chain specific acyl-CoA dehydrogenase, mitochondrial;
Px005413.1	1.1117	2.64E-198	8.16E-197	Protein lethal(2)essential for life;
Px000042.3	1.1085	4.45E-27	3.66E-26	Alcohol dehydrogenase [NADP+];
Px016883.1	1.1047	3.11E-06	1.06E-05	Mitogen-activated protein kinase kinase kinase kinase 3;
Px011311.1	1.1044	8.81E-09	3.65E-08	Proteasome subunit beta type-1;
Px003316.1	1.0981	0.00E+00	0.00E+00	C-1-tetrahydrofolate synthase, cytoplasmic;
Px016926.1	1.0912	1.86E-15	1.08E-14	Ubiquitin carboxyl-terminal hydrolase 20;
Px004968.1	1.0900	1.23E-11	6.07E-11	RNA 3'-terminal phosphate cyclase;
Px008244.2	1.0882	5.98E-89	1.04E-87	Sialic acid synthase; similar with SIAS_HUMAN;
Px008596.1	1.0870	1.05E-14	5.94E-14	Transmembrane protease serine 9;
Px013663.1	1.0859	8.76E-97	1.60E-95	Aldehyde dehydrogenase family 1 member L1;
Px009265.1	1.0812	1.04E-17	6.55E-17	Sepiapterin reductase; similar with SPRE_XENTR;
Px005900.1	1.0773	1.30E-16	7.94E-16	Cytochrome P450 6B2; similar with CP6B2_HELAM;
Px016558.1	1.0773	3.20E-04	8.66E-04	Putative glycerol kinase 5; similar with GLPK5_DANRE;
Px005110.3	1.0773	2.59E-04	7.09E-04	ABC transporter G family member 23;
Px008898.1	1.0773	3.98E-06	1.34E-05	Serine/threonine-protein kinase GA29083;
Px009393.2	1.0773	2.59E-04	7.09E-04	Guanine deaminase; similar with GUAD_PONAB;
Px001888.1	1.0743	2.09E-65	2.94E-64	N-acetyltransferase 15; similar with NAT15_MOUSE;
Px000764.1	1.0743	4.55E-43	4.88E-42	Acetyl-CoA carboxylase; similar with ACAC_CHICK;
Px003029.1	1.0715	3.95E-23	2.93E-22	Insulin receptor; similar with INSR_XENLA;
Px000678.1	1.0709	3.30E-234	1.17E-232	Thioredoxin, mitochondrial; similar with THIOM_RAT;
Px007394.2	1.0691	1.25E-39	1.29E-38	Peroxidasin homolog; similar with PXDN_MOUSE;
Px007185.1	1.0652	1.08E-11	5.33E-11	ATP-binding cassette sub-family G member 4;
Px006816.1	1.0638	0.00E+00	0.00E+00	2-oxoisovalerate dehydrogenase subunit alpha, mitochondrial;
Px013543.1	1.0629	1.37E-18	8.94E-18	Matrix metalloproteinase-25;
Px004811.1	1.0625	9.30E-10	4.13E-09	Calcium-independent protein kinase C;
Px011285.1	1.0624	2.26E-250	8.22E-249	UDP-glucuronosyltransferase 2B4;
Px000825.3	1.0612	4.33E-28	3.63E-27	Ferrochelatase, mitochondrial;
Px007850.1	1.0586	5.47E-08	2.14E-07	Dynein heavy chain 2, axonemal;
Px010576.1	1.0580	2.09E-20	1.44E-19	Inositol oxygenase; similar with MIOX_DANRE;
Px013239.1	1.0562	3.46E-07	1.28E-06	Putative glycerol kinase 3;
Px003609.1	1.0559	4.25E-07	1.56E-06	Guanylate cyclase 32E; similar with GCY3E_DROME;
Px016756.1	1.0544	4.92E-12	2.48E-11	Cytoplasmic aconitate hydratase;
Px005951.1	1.0534	7.33E-57	9.34E-56	Phospholipid hydroperoxide glutathione peroxidase, mitochondrial;
Px015503.1	1.0510	1.25E-10	5.86E-10	Histone deacetylase 4; similar with HDAC4_MOUSE;
Px012100.1	1.0492	1.15E-05	3.71E-05	Stromal cell-derived factor 2; similar with SDF2_HUMAN;
Px000351.3	1.0490	3.50E-79	5.60E-78	Chitin synthase 8; similar with CHS8_USTMA;
Px005198.1	1.0445	2.21E-21	1.58E-20	Long-chain fatty acid transport protein 4;
Px012834.1	1.0441	9.14E-32	8.21E-31	Phosphopantothenate--cysteine ligase;
Px002232.1	1.0403	8.42E-08	3.25E-07	Spectrin alpha chain; similar with SPTCA_DROME;
Px010797.1	1.0393	1.27E-07	4.84E-07	Epidermal retinol dehydrogenase 2;
Px011708.1	1.0393	3.52E-26	2.82E-25	Amidophosphoribosyltransferase;
Px016062.1	1.0391	1.04E-10	4.90E-10	Prostamide/prostaglandin F synthase; similar with PGFS_RAT;

Px007493.1	1.0389	5.06E-106	9.92E-105	Delta-1-pyrroline-5-carboxylate synthase;
Px016651.3	1.0381	6.93E-06	2.28E-05	Polypeptide N-acetylgalactosaminyltransferase 5;
Px016983.3	1.0379	6.64E-09	2.78E-08	Serine proteinase stubble; similar with STUB_DROME;
Px013730.1	1.0378	8.17E-18	5.18E-17	Myrosinase 1; similar with MYRO1_BREBR;
Px004354.1	1.0356	2.73E-18	1.76E-17	UDP-N-acetylhexosamine pyrophosphorylase-like protein 1;
Px008096.1	1.0350	1.59E-05	5.03E-05	TNF receptor-associated factor 6-B;
Px008659.1	1.0347	2.76E-08	1.10E-07	Tyrosine-protein phosphatase non-receptor type 23;
Px016791.1	1.0335	1.04E-22	7.66E-22	Inositol oxygenase; similar with MIOX_DANRE;
Px006391.1	1.0276	4.24E-21	3.00E-20	ATP-binding cassette sub-family B member 6, mitochondrial;
Px003748.1	1.0252	1.80E-24	1.37E-23	Sphingosine-1-phosphate lyase; similar with SGPL_DROME;
Px010631.1	1.0225	2.51E-26	2.02E-25	Sphingomyelin phosphodiesterase;
Px008837.1	1.0217	1.55E-94	2.77E-93	Long-chain-fatty-acid--CoA ligase ACSBG2;
Px010263.1	1.0192	2.96E-04	8.05E-04	Probable methylmalonate-semialdehyde dehydrogenase, mitochondrial;
Px008871.1	1.0184	8.47E-17	5.20E-16	Beta-1,3-galactosyltransferase brn;
Px015217.1	1.0179	5.90E-29	5.04E-28	4-aminobutyrate aminotransferase, mitochondrial;
Px005901.1	1.0179	5.85E-11	2.79E-10	Cytochrome P450 6B4; similar with CP6B4_PAPGL;
Px004326.1	1.0176	3.65E-04	9.79E-04	PI-PLC X domain-containing protein 3;
Px012551.1	1.0171	6.11E-09	2.57E-08	Uncharacterized protein R102.4; similar with YF64_CAEEL;
Px014822.1	1.0107	6.18E-81	1.00E-79	3-ketoacyl-CoA thiolase, mitochondrial;
Px013693.1	1.0094	5.72E-08	2.23E-07	Aldehyde oxidase; similar with ADO_HUMAN;
Px003215.1	1.0078	3.27E-10	1.49E-09	N-acetyllactosaminide beta-1,3-N-acetylglucosaminyltransferase;
Px004243.3	1.0071	7.75E-78	1.23E-76	Ferritin subunit; similar with FRI_AEDAE;
Px015882.1	1.0071	2.32E-110	4.67E-109	Alcohol dehydrogenase [NADP+] A;
Px010660.1	1.0065	4.71E-17	2.92E-16	Probable general secretion pathway protein D;
Px009169.1	1.0057	5.15E-06	1.71E-05	Myrosinase 1; similar with MYRO1_BREBR;
Px004984.1	1.0033	2.67E-04	7.30E-04	Peroxisomal leader peptide-processing protease;
Px012297.1	1.0024	1.28E-14	7.21E-14	ABC transporter G family member 20;
Px013169.1	1.0023	2.92E-07	1.09E-06	Lactase-phlorizin hydrolase; similar with LPH_HUMAN;
Px002093.2	1.0006	1.18E-05	3.78E-05	Ras-like protein 1; similar with RAS1_DROYA;
Px014199.1	-1.0020	3.63E-06	1.23E-05	Serine/threonine-protein kinase atr;
Px002896.2	-1.0039	5.09E-96	9.24E-95	Recombination repair protein 1;
Px012716.1	-1.0128	4.03E-70	5.92E-69	Proline dehydrogenase 1, mitochondrial;
Px014740.1	-1.0140	7.74E-60	1.02E-58	Probable ATP-dependent RNA helicase DDX27;
Px006855.1	-1.0156	1.75E-131	3.94E-130	STE20-like serine/threonine-protein kinase;
Px009226.1	-1.0167	9.86E-10	4.37E-09	DNA replication licensing factor MCM4;
Px002786.1	-1.0167	2.80E-85	4.70E-84	Elongation factor Tu, mitochondrial;
Px013606.1	-1.0228	0.00E+00	0.00E+00	Unknown function
Px014827.2	-1.0240	2.30E-10	1.06E-09	Interleukin enhancer-binding factor 2 homolog;
Px016098.1	-1.0241	8.54E-10	3.80E-09	Phytanoyl-CoA dioxygenase, peroxisomal;
Px003169.3	-1.0251	2.29E-69	3.34E-68	Probable lysine-specific demethylase 4B;
Px004110.1	-1.0281	8.36E-07	2.99E-06	Aldose reductase; similar with ALDR_RABIT;
Px008828.1	-1.0322	1.20E-16	7.32E-16	Uncharacterized protein C2orf79 homolog;
Px011056.1	-1.0324	1.65E-139	3.92E-138	rRNA 2'-O-methyltransferase fibrillarin;
Px001502.1	-1.0334	1.38E-18	9.02E-18	Ubiquitin carboxyl-terminal hydrolase 2;
Px018067.1	-1.0346	8.35E-101	1.58E-99	Twitchin; similar with UNC22_CAEEL;
Px013971.1	-1.0481	0.00E+00	0.00E+00	Twitchin; similar with UNC22_CAEEL;
Px009682.1	-1.0521	0.00E+00	0.00E+00	Cytochrome c oxidase subunit 6A1, mitochondrial;
Px012460.1	-1.0566	1.78E-04	5.00E-04	E3 ubiquitin-protein ligase hyd; similar with HYD_DROME;
Px010334.1	-1.0697	2.71E-144	6.64E-143	46 kDa FK506-binding nuclear protein;
Px013049.2	-1.0857	1.18E-05	3.79E-05	Uncharacterized aarF domain-containing protein kinase 1;
Px000929.1	-1.0927	2.38E-04	6.54E-04	Octopamine receptor beta-2R; similar with OCTB2_DROME;
Px016931.1	-1.0939	3.25E-26	2.60E-25	Methyltransferase-like protein 6;
Px014608.2	-1.0993	1.84E-38	1.86E-37	AGAP011281-PA; similar with Q7QHP0_ANOGA;
Px013792.1	-1.1047	6.26E-31	5.54E-30	Thrombospondin type-1 domain-containing protein 7A;

Px013179.1	-1.1092	3.73E-04	9.98E-04	Insulin-degrading enzyme; similar with IDE_HUMAN;
Px012461.1	-1.1191	1.86E-13	9.95E-13	Lethal(2)neighbour of tid protein;
Px004104.1	-1.1191	3.46E-06	1.17E-05	GH18371; similar with B4JEW4_DROGR;
Px010245.2	-1.1234	1.68E-45	1.88E-44	Probable small nuclear ribonucleoprotein G;
Px010417.1	-1.1283	8.94E-13	4.66E-12	Lysine-specific demethylase 5B;
Px006411.1	-1.1419	6.89E-15	3.93E-14	UDP-N-acetylglucosamine--peptide N-acetylglucosaminyltransferase 110 kDa subunit;
Px002001.1	-1.1422	3.10E-161	8.27E-160	Peptidyl-prolyl cis-trans isomerase-like 4;
Px010954.1	-1.1513	1.34E-08	5.50E-08	Probable DNA-directed RNA polymerases I and III subunit RPAC2;
Px015490.1	-1.1584	5.68E-45	6.25E-44	Probable ATP-dependent RNA helicase DDX52;
Px002394.3	-1.1661	5.87E-70	8.60E-69	D-beta-hydroxybutyrate dehydrogenase, mitochondrial;
Px000827.1	-1.1679	2.17E-07	8.16E-07	Ubiquitin carboxyl-terminal hydrolase 2;
Px017914.1	-1.1872	0.00E+00	0.00E+00	Paramyosin, long form; similar with MYSP1_DROME;
Px000295.3	-1.1899	1.60E-274	6.33E-273	Halomucin; similar with HMU_HALWD;
Px008486.1	-1.1907	3.74E-24	2.84E-23	Pre-mRNA-processing factor 19;
Px005700.2	-1.1982	9.41E-21	6.58E-20	Serine/threonine-protein kinase SBK1;
Px003971.1	-1.2004	2.05E-216	6.76E-215	Unknown function
Px012350.1	-1.2045	5.28E-49	6.14E-48	Ribonucleoside-diphosphate reductase large subunit;
Px012661.1	-1.2046	8.66E-13	4.51E-12	cAMP-dependent protein kinase type II regulatory subunit;
Px014783.1	-1.2077	6.81E-102	1.30E-100	Histone-lysine N-methyltransferase SETD1B;
Px002758.1	-1.2096	1.96E-07	7.39E-07	RNA polymerase II subunit B1 CTD phosphatase Rpap2;
Px007614.2	-1.2127	9.33E-98	1.72E-96	Muscle M-line assembly protein unc-89;
Px013191.1	-1.2294	5.69E-11	2.72E-10	Aromatic-L-amino-acid decarboxylase;
Px003102.1	-1.2302	1.72E-11	8.46E-11	tRNA (uracil-5-)-methyltransferase homolog A;
Px017913.1	-1.2473	0.00E+00	0.00E+00	Paramyosin, short form; similar with MYSP2_DROME;
Px007815.1	-1.2476	0.00E+00	0.00E+00	Myosin heavy chain, muscle;
Px009182.1	-1.2505	0.00E+00	0.00E+00	Seryl-tRNA synthetase, cytoplasmic;
Px012158.1	-1.2615	1.63E-10	7.54E-10	Alpha-N-acetylgalactosaminidase;
Px016541.1	-1.2656	6.55E-07	2.37E-06	Elongation factor Tu, mitochondrial;
Px002640.1	-1.2858	0.00E+00	0.00E+00	Alpha-actinin, sarcomeric; similar with ACTN_DROME;
Px012129.1	-1.2949	1.78E-33	1.64E-32	Lysosomal Pro-X carboxypeptidase;
Px015632.1	-1.3238	2.97E-07	1.10E-06	Flap endonuclease 1; similar with FEN1_DROAN;
Px009149.1	-1.3366	4.40E-70	6.46E-69	116 kDa U5 small nuclear ribonucleoprotein component;
Px008876.1	-1.3446	1.07E-09	4.74E-09	Tubulin beta-4 chain; similar with TBB4_CAEEL;
Px010423.1	-1.3483	1.94E-09	8.45E-09	G/T mismatch-specific thymine DNA glycosylase;
Px017176.1	-1.3522	4.34E-23	3.21E-22	Probable ATP-dependent RNA helicase kurz;
Px010023.2	-1.3680	1.39E-22	1.02E-21	ATP-dependent RNA helicase DDX24;
Px000852.1	-1.3773	7.86E-06	2.57E-05	Probable phytanoyl-CoA dioxygenase;
Px009314.2	-1.3802	1.36E-12	7.02E-12	A disintegrin and metalloproteinase with thrombospondin motifs 16;
Px010117.1	-1.3859	3.21E-207	1.02E-205	Asparagine synthetase [glutamine-hydrolyzing];
Px005941.1	-1.3879	3.50E-09	1.50E-08	UDP-N-acetylglucosamine 1-carboxyvinyltransferase 2;
Px008352.1	-1.3967	7.24E-17	4.46E-16	SOX domain-containing protein dichaete;
Px004087.1	-1.3985	1.15E-50	1.37E-49	Signal peptidase complex subunit 2;
Px009337.1	-1.4082	8.00E-05	2.35E-04	M-phase inducer phosphatase; similar with MPIP_DROME;
Px014167.1	-1.4107	0.00E+00	0.00E+00	Titin; similar with TITIN_DROME;
Px001501.4	-1.4245	9.22E-22	6.65E-21	Ubiquitin carboxyl-terminal hydrolase;
Px013713.2	-1.4278	1.06E-42	1.13E-41	GMP reductase 1; similar with GMPR1_MOUSE;
Px006766.1	-1.4330	1.04E-119	2.20E-118	ATP-binding cassette sub-family F member 1;
Px011795.1	-1.4417	5.27E-29	4.51E-28	RuvB-like helicase 2; similar with RUVB2_DROPS;
Px017708.4	-1.4493	1.01E-40	1.05E-39	Long-chain-fatty-acid--CoA ligase 1;
Px009474.1	-1.4691	0.00E+00	0.00E+00	H/ACA ribonucleoprotein complex subunit 4;
Px000037.1	-1.4737	2.19E-29	1.89E-28	DNA replication licensing factor Mcm5;
Px005468.1	-1.4753	3.05E-04	8.27E-04	Alpha,alpha-trehalose-phosphate synthase [UDP-forming];
Px000572.1	-1.4791	1.12E-04	3.23E-04	Phosphatidylinositol-binding clathrin assembly protein LAP;
Px009530.1	-1.5142	5.85E-17	3.61E-16	DNA polymerase eta; similar with POLH_HUMAN;

Px016332.1	-1.5166	0.00E+00	0.00E+00	Calcium-transporting ATPase sarcoplasmic/endoplasmic reticulum type;
Px002987.1	-1.5181	0.00E+00	0.00E+00	Methionine aminopeptidase 2;
Px015198.2	-1.5238	2.14E-20	1.48E-19	NADH dehydrogenase [ubiquinone] 1 alpha subcomplex subunit 8;
Px013969.3	-1.5363	0.00E+00	0.00E+00	Twitchin; similar with UNC22_CAEEL;
Px010951.2	-1.5366	3.53E-143	8.64E-142	Lysosomal alpha-mannosidase;
Px014636.1	-1.5417	2.28E-04	6.26E-04	Trypsin-1; similar with TRY1_ANOGA;
Px013405.1	-1.5636	1.01E-18	6.66E-18	CAP isoform A; similar with C9DTM4_BOMMO;
Px002580.1	-1.6232	2.48E-05	7.70E-05	Kinase suppressor of Ras 2; similar with KSR2_HUMAN;
Px011825.1	-1.6306	5.34E-09	2.25E-08	Glucose dehydrogenase [acceptor];
Px004483.1	-1.6597	9.63E-08	3.71E-07	N-alpha-acetyltransferase 20, NatB catalytic subunit;
Px016787.1	-1.6669	1.01E-06	3.59E-06	Insulin-degrading enzyme; similar with IDE_HUMAN;
Px009430.1	-1.6972	1.08E-06	3.83E-06	Histone-arginine methyltransferase CARMER;
Px016603.1	-1.7020	0.00E+00	0.00E+00	Muscle M-line assembly protein unc-89;
Px003941.1	-1.7142	4.69E-05	1.42E-04	Probable phospholipid-transporting ATPase IA;
Px014610.1	-1.7464	1.84E-20	1.28E-19	Tubulin beta-2 chain; similar with TBB2_HOMAM;
Px001595.1	-1.7505	2.87E-215	9.40E-214	Peptidyl-prolyl cis-trans isomerase 1;
Px015524.1	-1.7807	3.80E-14	2.11E-13	Cyclin-dependent kinase 12;
Px007377.1	-1.9772	8.53E-07	3.05E-06	Glucose 1-dehydrogenase 1; similar with DHG1_BACME;
Px002199.1	-2.0641	1.94E-17	1.22E-16	4-coumarate--CoA ligase-like 5;
Px013590.1	-2.0927	3.30E-08	1.31E-07	Protein arginine N-methyltransferase 3;
Px009473.1	-2.1154	2.05E-08	8.25E-08	DNA-directed RNA polymerases I, II, and III subunit RPABC3;
Px011896.1	-2.2353	0.00E+00	0.00E+00	PAB-dependent poly(A)-specific ribonuclease subunit 2;
Px012510.1	-2.2884	1.67E-09	7.30E-09	N-glycosylase/DNA lyase; similar with OGG1_DROME;
Px015353.2	-2.4729	9.32E-07	3.32E-06	Fibroblast growth factor receptor 3;
Px009429.3	-2.5772	1.39E-110	2.81E-109	ATP-dependent RNA helicase DDX24;
Px017404.1	-2.6008	9.07E-06	2.95E-05	Fidgetin-like protein 1; similar with FIGL1_XENLA;
Px012212.1	-2.8769	4.13E-06	1.39E-05	Multidrug resistance protein homolog 49;
Px010814.1	-3.0382	1.61E-05	5.11E-05	ADAMTS-like protein 3; similar with ATL3_HUMAN;
Px000980.1	-3.3822	3.42E-05	1.05E-04	Catalase; similar with CATA_PONAB;
Px003126.1	-3.3822	3.42E-05	1.05E-04	Adenylate cyclase type 3; similar with ADCY3_MOUSE;
Px017388.1	-8.2367	1.78E-04	4.99E-04	Neither inactivation nor afterpotential protein G;
Px012074.1	#######	3.46E-04	9.33E-04	Acylphosphatase-2; similar with ACYP2_ANAPL;

GO:0008236

Px015905.1	13.4712	1.77E-71	2.63E-70	Transmembrane protease serine 6;
Px003000.1	12.5364	2.28E-42	2.43E-41	Transmembrane protease serine 6;
Px006453.1	10.3391	8.15E-10	3.63E-09	Transmembrane protease serine 6;
Px017505.1	9.7974	1.71E-04	4.81E-04	Plasma kallikrein; similar with KLKB1_HUMAN;
Px007670.1	6.2472	5.75E-63	7.92E-62	Proclotting enzyme; similar with PCE_TACTR;
Px005241.1	4.3794	1.43E-53	1.74E-52	Trypsin CFT-1; similar with TRYP_CHOFU;
Px005243.1	3.9876	0.00E+00	0.00E+00	Trypsin, alkaline A; similar with TRYA_MANSE;
Px015277.1	3.9698	7.98E-158	2.09E-156	Trypsin CFT-1; similar with TRYP_CHOFU;
Px010313.2	3.7896	2.43E-106	4.78E-105	Serine proteinase stubble; similar with STUB_DROME;
Px006623.1	3.7777	1.10E-26	9.00E-26	Transmembrane protease serine 9;
Px012813.1	3.6622	2.98E-06	1.02E-05	Serine proteinase stubble; similar with STUB_DROME;
Px008538.1	3.4202	3.15E-138	7.39E-137	Trypsin, alkaline B; similar with TRYB_MANSE;
Px005657.1	3.4122	1.95E-23	1.46E-22	Proclotting enzyme; similar with PCE_TACTR;
Px007621.1	3.3847	6.61E-21	4.65E-20	Trypsin CFT-1; similar with TRYP_CHOFU;
Px007619.2	3.0951	0.00E+00	0.00E+00	Trypsin, alkaline A; similar with TRYA_MANSE;
Px003479.1	2.9314	1.50E-17	9.44E-17	Protein rhomboid; similar with RHOM_DROME;
Px015280.1	2.9138	7.05E-10	3.15E-09	Trypsin CFT-1; similar with TRYP_CHOFU;
Px007618.1	2.7222	1.13E-272	4.41E-271	Trypsin CFT-1; similar with TRYP_CHOFU;
Px007674.2	2.5239	0.00E+00	0.00E+00	Collagenase; similar with COGS_HYPLI;
Px007675.1	2.5233	0.00E+00	0.00E+00	Collagenase; similar with COGS_HYPLI;

Px010489.1	2.4085	6.89E-66	9.73E-65	Trypsin, alkaline C; similar with TRYC_MANSE;
Px009395.1	2.3871	1.03E-17	6.47E-17	Proclotting enzyme; similar with PCE_TACTR;
Px007616.1	2.3746	1.09E-158	2.87E-157	Trypsin CFT-1; similar with TRYP_CHOFU;
Px015275.1	2.3286	0.00E+00	0.00E+00	Trypsin CFT-1; similar with TRYP_CHOFU;
Px006622.3	2.2728	9.45E-20	6.46E-19	Ovochymase-1; similar with OVCH1_HUMAN;
Px011796.5	2.0740	1.05E-51	1.26E-50	Putative aminopeptidase W07G4.4;
Px001833.1	2.0603	0.00E+00	0.00E+00	Serine protease easter; similar with EAST_DROME;
Px011097.1	1.9460	2.65E-10	1.22E-09	Serine proteinase stubble; similar with STUB_DROME;
Px008406.1	1.8639	2.77E-08	1.10E-07	Serine proteinase stubble; similar with STUB_DROME;
Px009715.1	1.5988	8.85E-16	5.24E-15	Mitochondrial inner membrane protease subunit 1;
Px016171.2	1.3471	1.79E-54	2.21E-53	Prolyl endopeptidase; similar with PPCE_MOUSE;
Px008596.1	1.0870	1.05E-14	5.94E-14	Transmembrane protease serine 9;
Px016983.3	1.0379	6.64E-09	2.78E-08	Serine proteinase stubble; similar with STUB_DROME;

GO:0017171

Px015905.1	13.4712	1.77E-71	2.63E-70	Transmembrane protease serine 6;
Px003000.1	12.5364	2.28E-42	2.43E-41	Transmembrane protease serine 6;
Px006453.1	10.3391	8.15E-10	3.63E-09	Transmembrane protease serine 6;
Px017505.1	9.7974	1.71E-04	4.81E-04	Plasma kallikrein; similar with KLKB1_HUMAN;
Px007670.1	6.2472	5.75E-63	7.92E-62	Proclotting enzyme; similar with PCE_TACTR;
Px005241.1	4.3794	1.43E-53	1.74E-52	Trypsin CFT-1; similar with TRYP_CHOFU;
Px005243.1	3.9876	0.00E+00	0.00E+00	Trypsin, alkaline A; similar with TRYA_MANSE;
Px015277.1	3.9698	7.98E-158	2.09E-156	Trypsin CFT-1; similar with TRYP_CHOFU;
Px010313.2	3.7896	2.43E-106	4.78E-105	Serine proteinase stubble; similar with STUB_DROME;
Px006623.1	3.7777	1.10E-26	9.00E-26	Transmembrane protease serine 9;
Px012813.1	3.6622	2.98E-06	1.02E-05	Serine proteinase stubble; similar with STUB_DROME;
Px008538.1	3.4202	3.15E-138	7.39E-137	Trypsin, alkaline B; similar with TRYB_MANSE;
Px005657.1	3.4122	1.95E-23	1.46E-22	Proclotting enzyme; similar with PCE_TACTR;
Px007621.1	3.3847	6.61E-21	4.65E-20	Trypsin CFT-1; similar with TRYP_CHOFU;
Px007619.2	3.0951	0.00E+00	0.00E+00	Trypsin, alkaline A; similar with TRYA_MANSE;
Px003479.1	2.9314	1.50E-17	9.44E-17	Protein rhomboid; similar with RHOM_DROME;
Px015280.1	2.9138	7.05E-10	3.15E-09	Trypsin CFT-1; similar with TRYP_CHOFU;
Px007618.1	2.7222	1.13E-272	4.41E-271	Trypsin CFT-1; similar with TRYP_CHOFU;
Px007674.2	2.5239	0.00E+00	0.00E+00	Collagenase; similar with COGS_HYPLI;
Px007675.1	2.5233	0.00E+00	0.00E+00	Collagenase; similar with COGS_HYPLI;
Px010489.1	2.4085	6.89E-66	9.73E-65	Trypsin, alkaline C; similar with TRYC_MANSE;
Px009395.1	2.3871	1.03E-17	6.47E-17	Proclotting enzyme; similar with PCE_TACTR;
Px007616.1	2.3746	1.09E-158	2.87E-157	Trypsin CFT-1; similar with TRYP_CHOFU;
Px015275.1	2.3286	0.00E+00	0.00E+00	Trypsin CFT-1; similar with TRYP_CHOFU;
Px006622.3	2.2728	9.45E-20	6.46E-19	Ovochymase-1; similar with OVCH1_HUMAN;
Px011796.5	2.0740	1.05E-51	1.26E-50	Putative aminopeptidase W07G4.4;
Px001833.1	2.0603	0.00E+00	0.00E+00	Serine protease easter; similar with EAST_DROME;
Px011097.1	1.9460	2.65E-10	1.22E-09	Serine proteinase stubble; similar with STUB_DROME;
Px008406.1	1.8639	2.77E-08	1.10E-07	Serine proteinase stubble; similar with STUB_DROME;
Px009715.1	1.5988	8.85E-16	5.24E-15	Mitochondrial inner membrane protease subunit 1;
Px016171.2	1.3471	1.79E-54	2.21E-53	Prolyl endopeptidase; similar with PPCE_MOUSE;
Px008596.1	1.0870	1.05E-14	5.94E-14	Transmembrane protease serine 9;
Px016983.3	1.0379	6.64E-09	2.78E-08	Serine proteinase stubble; similar with STUB_DROME;

GO:0070011

Px015905.1	13.4712	1.77E-71	2.63E-70	Transmembrane protease serine 6;
Px003000.1	12.5364	2.28E-42	2.43E-41	Transmembrane protease serine 6;
Px006453.1	10.3391	8.15E-10	3.63E-09	Transmembrane protease serine 6;
Px017505.1	9.7974	1.71E-04	4.81E-04	Plasma kallikrein; similar with KLKB1_HUMAN;

Px007670.1	6.2472	5.75E-63	7.92E-62	Proclotting enzyme; similar with PCE_TACTR;
Px000995.2	4.3915	0.00E+00	0.00E+00	Carboxypeptidase A4; similar with CBPA4_HUMAN;
Px005241.1	4.3794	1.43E-53	1.74E-52	Trypsin CFT-1; similar with TRYP_CHOFU;
Px005243.1	3.9876	0.00E+00	0.00E+00	Trypsin, alkaline A; similar with TRYA_MANSE;
Px015277.1	3.9698	7.98E-158	2.09E-156	Trypsin CFT-1; similar with TRYP_CHOFU;
Px010313.2	3.7896	2.43E-106	4.78E-105	Serine proteinase stubble; similar with STUB_DROME;
Px006623.1	3.7777	1.10E-26	9.00E-26	Transmembrane protease serine 9;
Px013732.1	3.7553	4.20E-15	2.42E-14	Proteasome subunit alpha type-7-1;
Px012813.1	3.6622	2.98E-06	1.02E-05	Serine proteinase stubble; similar with STUB_DROME;
Px000992.1	3.6006	0.00E+00	0.00E+00	Carboxypeptidase A1; similar with CBPA1_BOVIN;
Px008538.1	3.4202	3.15E-138	7.39E-137	Trypsin, alkaline B; similar with TRYB_MANSE;
Px005657.1	3.4122	1.95E-23	1.46E-22	Proclotting enzyme; similar with PCE_TACTR;
Px007621.1	3.3847	6.61E-21	4.65E-20	Trypsin CFT-1; similar with TRYP_CHOFU;
Px008277.1	3.3443	1.09E-141	2.62E-140	Membrane alanyl aminopeptidase;
Px015928.1	3.1647	2.57E-04	7.05E-04	Carboxypeptidase B; similar with CBPB_ASTFL;
Px007619.2	3.0951	0.00E+00	0.00E+00	Trypsin, alkaline A; similar with TRYA_MANSE;
Px015049.1	2.9693	5.52E-35	5.22E-34	Venom dipeptidyl peptidase 4; similar with VDDP4_VESVU;
Px003479.1	2.9314	1.50E-17	9.44E-17	Protein rhomboid; similar with RHOM_DROME;
Px015280.1	2.9138	7.05E-10	3.15E-09	Trypsin CFT-1; similar with TRYP_CHOFU;
Px000994.1	2.8631	0.00E+00	0.00E+00	Zinc carboxypeptidase A 1; similar with CBPA1_DROPS;
Px002993.1	2.7717	0.00E+00	0.00E+00	Zinc metalloproteinase nas-4; similar with NAS4_CAEEL;
Px005373.1	2.7565	1.52E-39	1.56E-38	Carboxypeptidase B; similar with CBPB_ASTFL;
Px007618.1	2.7222	1.13E-272	4.41E-271	Trypsin CFT-1; similar with TRYP_CHOFU;
Px016567.1	2.6458	1.14E-175	3.25E-174	Matrix metalloproteinase-14; similar with MMP14_RAT;
Px007674.2	2.5239	0.00E+00	0.00E+00	Collagenase; similar with COGS_HYPLI;
Px007675.1	2.5233	0.00E+00	0.00E+00	Collagenase; similar with COGS_HYPLI;
Px010489.1	2.4085	6.89E-66	9.73E-65	Trypsin, alkaline C; similar with TRYC_MANSE;
Px003467.1	2.3876	2.10E-18	1.36E-17	Matrix metalloproteinase-14; similar with MMP14_RABIT;
Px009395.1	2.3871	1.03E-17	6.47E-17	Proclotting enzyme; similar with PCE_TACTR;
Px007616.1	2.3746	1.09E-158	2.87E-157	Trypsin CFT-1; similar with TRYP_CHOFU;
Px001708.1	2.3708	0.00E+00	0.00E+00	Aminopeptidase N; similar with AMPN_PLUXY;
Px015275.1	2.3286	0.00E+00	0.00E+00	Trypsin CFT-1; similar with TRYP_CHOFU;
Px001705.1	2.3213	0.00E+00	0.00E+00	Membrane alanyl aminopeptidase;
Px006622.3	2.2728	9.45E-20	6.46E-19	Ovochymase-1; similar with OVCH1_HUMAN;
Px001990.2	2.2289	2.92E-104	5.69E-103	Carboxypeptidase B; similar with CBPB_ASTFL;
Px001633.1	2.2212	3.10E-80	5.00E-79	Angiotensin-converting enzyme;
Px011796.5	2.0740	1.05E-51	1.26E-50	Putative aminopeptidase W07G4.4;
Px001833.1	2.0603	0.00E+00	0.00E+00	Serine protease easter; similar with EAST_DROME;
Px002632.1	2.0480	2.47E-165	6.70E-164	Xaa-Pro dipeptidase; similar with PEPD_HUMAN;
Px011097.1	1.9460	2.65E-10	1.22E-09	Serine proteinase stubble; similar with STUB_DROME;
Px003754.1	1.9228	1.67E-147	4.13E-146	Membrane alanyl aminopeptidase;
Px006956.1	1.8788	1.02E-112	2.08E-111	Xaa-Pro dipeptidase; similar with PEPD_HUMAN;
Px008406.1	1.8639	2.77E-08	1.10E-07	Serine proteinase stubble; similar with STUB_DROME;
Px015778.1	1.7676	5.49E-08	2.14E-07	Sentrin-specific protease 8; similar with SENP8_HUMAN;
Px012643.1	1.7650	5.67E-95	1.02E-93	Angiotensin-converting enzyme; similar with ACE_MOUSE;
Px003753.1	1.7587	0.00E+00	0.00E+00	Membrane alanyl aminopeptidase;
Px006502.1	1.6424	1.34E-55	1.68E-54	Calpain-B; similar with CANB_DROME;
Px009715.1	1.5988	8.85E-16	5.24E-15	Mitochondrial inner membrane protease subunit 1;
Px011489.1	1.4705	1.48E-69	2.16E-68	Gamma-glutamyl hydrolase A; similar with GGHA_DICDI;
Px005726.1	1.4124	1.92E-12	9.85E-12	Xaa-Pro aminopeptidase 1;
Px005747.1	1.4025	6.57E-16	3.92E-15	Proteasome subunit alpha type-5;
Px016171.2	1.3471	1.79E-54	2.21E-53	Prolyl endopeptidase; similar with PPCE_MOUSE;
Px008337.1	1.2761	1.16E-111	2.35E-110	Leishmanolysin-like peptidase;
Px002715.1	1.2673	4.39E-154	1.12E-152	Cathepsin L; similar with CATL_DROME;

Px010436.1	1.1181	3.98E-92	7.03E-91	Venom serine carboxypeptidase; similar with VCP_APIME;
Px008596.1	1.0870	1.05E-14	5.94E-14	Transmembrane protease serine 9;
Px016983.3	1.0379	6.64E-09	2.78E-08	Serine proteinase stubble; similar with STUB_DROME;
Px001502.1	-1.0334	1.38E-18	9.02E-18	Ubiquitin carboxyl-terminal hydrolase 2;
Px013179.1	-1.1092	3.73E-04	9.98E-04	Insulin-degrading enzyme; similar with IDE_HUMAN;
Px000827.1	-1.1679	2.17E-07	8.16E-07	Ubiquitin carboxyl-terminal hydrolase 2;
Px000572.1	-1.4791	1.12E-04	3.23E-04	Phosphatidylinositol-binding clathrin assembly protein LAP;
Px002987.1	-1.5181	0.00E+00	0.00E+00	Methionine aminopeptidase 2;
Px016787.1	-1.6669	1.01E-06	3.59E-06	Insulin-degrading enzyme; similar with IDE_HUMAN;
Px010814.1	-3.0382	1.61E-05	5.11E-05	ADAMTS-like protein 3; similar with ATL3_HUMAN;

Table K. GO enrichment analysis of *B. bassiana* genes at 24 hpt_I.

GO ID	Gene numbers	P-value	FDR	GO term
**Cellular component**				
GO:0005622	469	2.85E-20	3.90E-17	intracellular
GO:0044424	455	2.79E-19	1.91E-16	intracellular part
GO:0005623	539	2.59E-18	8.87E-16	cell
GO:0044464	539	2.59E-18	8.87E-16	cell part
GO:0032991	198	3.64E-14	7.24E-12	macromolecular complex
GO:0005575	677	6.79E-14	1.16E-11	cellular_component
GO:0030529	101	2.24E-13	3.41E-11	ribonucleoprotein complex
GO:0043229	260	1.44E-07	8.56E-06	intracellular organelle
GO:0043226	261	1.78E-07	9.50E-06	organelle
GO:0043227	224	4.03E-07	1.58E-05	membrane-bounded organelle
GO:0043231	210	9.37E-07	3.56E-05	intracellular membrane-bounded organelle
GO:0044446	99	7.36E-06	2.65E-04	intracellular organelle part
GO:0043234	85	5.58E-05	1.82E-03	protein complex
GO:0044422	104	1.79E-04	5.44E-03	organelle part
GO:0044444	72	7.71E-04	2.25E-02	cytoplasmic part
GO:0005737	72	1.06E-03	3.02E-02	cytoplasm
GO:0000502	13	1.36E-03	3.79E-02	proteasome complex
GO:0005839	7	4.01E-03	7.73E-02	proteasome core complex
GO:0098588	15	5.88E-03	1.09E-01	bounding membrane of organelle
GO:0019866	34	6.04E-03	1.10E-01	organelle inner membrane
GO:0044432	8	8.82E-03	1.46E-01	endoplasmic reticulum part
**Molecular Function**				
GO:0005198	70	2.06E-14	5.64E-12	structural molecule activity
GO:0000166	276	1.39E-09	1.59E-07	nucleotide binding
GO:1901265	276	1.39E-09	1.59E-07	nucleoside phosphate binding
GO:0036094	283	2.10E-09	2.05E-07	small molecule binding
GO:0032559	157	5.35E-08	3.74E-06	adenyl ribonucleotide binding
GO:0030554	157	7.63E-08	4.97E-06	adenyl nucleotide binding
GO:0097159	357	1.80E-07	9.50E-06	organic cyclic compound binding
GO:0097367	190	1.99E-07	9.68E-06	carbohydrate derivative binding
GO:0032553	188	2.05E-07	9.68E-06	ribonucleotide binding
GO:0032555	188	2.05E-07	9.68E-06	purine ribonucleotide binding
GO:1901363	356	2.20E-07	1.00E-05	heterocyclic compound binding
GO:0017076	188	2.76E-07	1.11E-05	purine nucleotide binding
GO:0008135	27	7.42E-04	2.21E-02	translation factor activity, nucleic acid binding
GO:0016209	10	1.87E-03	4.87E-02	antioxidant activity
GO:0016879	24	2.38E-03	5.35E-02	ligase activity, forming carbon-nitrogen bonds
GO:0004601	7	3.23E-03	6.69E-02	peroxidase activity
GO:0016684	7	3.23E-03	6.69E-02	oxidoreductase activity
GO:0022890	16	4.92E-03	9.23E-02	inorganic cation transmembrane transporter activity
GO:0016831	8	7.18E-03	1.26E-01	carboxy-lyase activity
GO:0004175	26	7.88E-03	1.35E-01	endopeptidase activity
GO:0016861	8	8.91E-03	1.46E-01	intramolecular oxidoreductase activity
GO:0003723	30	9.36E-03	1.46E-01	RNA binding
**Biological process**				
GO:0071704	559	3.70E-14	7.24E-12	organic substance metabolic process
GO:0008152	788	2.48E-11	3.39E-09	metabolic process
GO:0044237	484	1.63E-09	1.72E-07	cellular metabolic process

GO:0044710	248	4.62E-09	4.09E-07	single-organism metabolic process
GO:0008150	1052	4.78E-09	4.09E-07	biological_process
GO:0010467	213	6.40E-09	5.15E-07	gene expression
GO:0019538	174	4.04E-08	3.08E-06	protein metabolic process
GO:0043170	358	5.46E-08	3.74E-06	macromolecule metabolic process
GO:0044281	186	8.94E-08	5.56E-06	small molecule metabolic process
GO:0044267	146	1.62E-07	9.25E-06	cellular protein metabolic process
GO:0009987	575	2.28E-07	1.01E-05	cellular process
GO:0044238	452	2.41E-07	1.03E-05	primary metabolic process
GO:0044763	277	2.76E-07	1.11E-05	single-organism cellular process
GO:0044699	334	1.97E-06	7.31E-05	single-organism process
GO:0006412	56	1.30E-05	4.56E-04	translation
GO:0006082	104	4.21E-05	1.44E-03	organic acid metabolic process
GO:0043436	103	5.59E-05	1.82E-03	oxoacid metabolic process
GO:1901564	118	9.09E-05	2.89E-03	organonitrogen compound metabolic process
GO:0019752	100	1.42E-04	4.43E-03	carboxylic acid metabolic process
GO:0006006	17	1.56E-03	4.26E-02	glucose metabolic process
GO:0009167	16	1.88E-03	4.87E-02	purine ribonucleoside monophosphate metabolic process
GO:0009168	16	1.88E-03	4.87E-02	purine ribonucleoside monophosphate biosynthetic process
GO:0006520	77	2.05E-03	5.05E-02	cellular amino acid metabolic process
GO:0006007	15	2.14E-03	5.05E-02	glucose catabolic process
GO:0019320	15	2.14E-03	5.05E-02	hexose catabolic process
GO:0044724	15	2.14E-03	5.05E-02	single-organism carbohydrate catabolic process
GO:0046365	15	2.14E-03	5.05E-02	monosaccharide catabolic process
GO:0055086	58	2.33E-03	5.35E-02	nucleobase-containing small molecule metabolic process
GO:0009056	62	2.38E-03	5.35E-02	catabolic process
GO:0006091	28	2.71E-03	5.98E-02	generation of precursor metabolites and energy
GO:0044723	31	2.80E-03	6.09E-02	single-organism carbohydrate metabolic process
GO:0044712	39	2.88E-03	6.15E-02	single-organism catabolic process
GO:0044260	237	3.28E-03	6.70E-02	cellular macromolecule metabolic process
GO:0044085	26	3.33E-03	6.71E-02	cellular component biogenesis
GO:0019318	21	3.45E-03	6.84E-02	hexose metabolic process
GO:0005996	22	4.00E-03	7.73E-02	monosaccharide metabolic process
GO:1901575	58	4.21E-03	8.01E-02	organic substance catabolic process
GO:0022613	22	6.25E-03	1.13E-01	ribonucleoprotein complex biogenesis
GO:0009199	34	7.00E-03	1.24E-01	ribonucleoside triphosphate metabolic process
GO:0009141	35	7.57E-03	1.31E-01	nucleoside triphosphate metabolic process
GO:0009126	16	9.18E-03	1.46E-01	purine nucleoside monophosphate metabolic process
GO:0009127	16	9.18E-03	1.46E-01	purine nucleoside monophosphate biosynthetic process
GO:0009156	16	9.19E-03	1.46E-01	ribonucleoside monophosphate biosynthetic process
GO:0009161	16	9.19E-03	1.46E-01	ribonucleoside monophosphate metabolic process
GO:0009201	12	9.35E-03	1.46E-01	ribonucleoside triphosphate biosynthetic process
GO:0009144	33	9.67E-03	1.47E-01	purine nucleoside triphosphate metabolic process
GO:0009205	33	9.67E-03	1.47E-01	purine ribonucleoside triphosphate metabolic process
GO:0019637	58	9.83E-03	1.48E-01	organophosphate metabolic process

Table L. GO enrichment analysis of *B. bassiana* genes at 36 hpt_I.

GO ID	Gene numbers	P-value	FDR	GO term
**Cellular component**				
GO:0005622	544	4.72E-29	6.46E-26	intracellular
GO:0044424	526	6.60E-27	4.52E-24	intracellular part
GO:0005623	618	7.15E-24	2.45E-21	cell
GO:0044464	618	7.15E-24	2.45E-21	cell part
GO:0005575	785	2.45E-20	6.71E-18	cellular_component
GO:0032991	230	3.28E-20	7.49E-18	macromolecular complex
GO:0030529	113	2.49E-16	4.86E-14	ribonucleoprotein complex
GO:0043226	300	1.49E-09	1.36E-07	organelle
GO:0043229	297	3.30E-09	2.66E-07	intracellular organelle
GO:0043227	256	1.13E-08	7.35E-07	membrane-bounded organelle
GO:0043231	241	1.88E-08	1.17E-06	intracellular membrane-bounded organelle
GO:0043234	101	1.15E-07	6.55E-06	protein complex
GO:0044422	125	1.84E-07	8.67E-06	organelle part
GO:0005737	89	6.61E-07	2.66E-05	cytoplasm
GO:0044446	112	7.49E-07	2.93E-05	intracellular organelle part
GO:0044444	88	9.78E-07	3.72E-05	cytoplasmic part
GO:0031090	73	1.06E-04	2.60E-03	organelle membrane
GO:0031967	54	2.80E-04	5.71E-03	organelle envelope
GO:0031975	54	2.80E-04	5.71E-03	envelope
GO:0030659	10	1.14E-03	1.86E-02	cytoplasmic vesicle membrane
GO:0044433	10	1.14E-03	1.86E-02	cytoplasmic vesicle part
GO:0019866	39	1.56E-03	2.45E-02	organelle inner membrane
GO:0030662	9	1.94E-03	3.01E-02	coated vesicle membrane
GO:0044429	32	1.97E-03	3.02E-02	mitochondrial part
GO:0005739	38	2.31E-03	3.47E-02	mitochondrion
GO:0012506	11	3.61E-03	4.90E-02	vesicle membrane
GO:0005634	22	4.49E-03	5.90E-02	nucleus
GO:0000502	13	6.17E-03	7.75E-02	proteasome complex
GO:0016469	20	6.39E-03	7.95E-02	proton-transporting two-sector ATPase complex
GO:0030135	10	6.47E-03	7.98E-02	coated vesicle
GO:0044455	8	8.54E-03	1.02E-01	mitochondrial membrane part
**Molecular Function**				
GO:0005198	73	2.92E-13	3.64E-11	structural molecule activity
GO:0097367	212	9.17E-08	5.46E-06	carbohydrate derivative binding
GO:0032559	172	1.23E-07	6.73E-06	adenyl ribonucleotide binding
GO:0032553	209	1.47E-07	7.47E-06	ribonucleotide binding
GO:0032555	209	1.47E-07	7.47E-06	purine ribonucleotide binding
GO:0030554	172	1.82E-07	8.67E-06	adenyl nucleotide binding
GO:0017076	209	2.08E-07	9.49E-06	purine nucleotide binding
GO:0003723	41	5.32E-07	2.28E-05	RNA binding
GO:0008135	33	2.66E-06	9.60E-05	translation factor activity, nucleic acid binding
GO:0036094	300	5.41E-06	1.90E-04	small molecule binding
GO:0000166	289	1.98E-05	6.31E-04	nucleotide binding
GO:1901265	289	1.98E-05	6.31E-04	nucleoside phosphate binding
GO:0097159	386	1.02E-04	2.53E-03	organic cyclic compound binding
GO:1901363	385	1.16E-04	2.79E-03	heterocyclic compound binding
GO:0016836	14	5.58E-04	1.00E-02	hydro-lyase activity
GO:0016651	12	2.55E-03	3.72E-02	oxidoreductase activity, acting on NAD(P)H
GO:0005515	43	2.88E-03	4.11E-02	protein binding

GO:0003954	9	3.13E-03	4.32E-02	NADH dehydrogenase activity
**Biological process**				
GO:0071704	629	3.20E-15	5.47E-13	organic substance metabolic process
GO:0043170	427	2.80E-14	4.26E-12	macromolecule metabolic process
GO:0010467	252	4.72E-14	6.46E-12	gene expression
GO:0019538	206	1.12E-12	1.27E-10	protein metabolic process
GO:0044267	173	1.23E-11	1.29E-09	cellular protein metabolic process
GO:0044237	548	1.07E-10	1.05E-08	cellular metabolic process
GO:0008152	876	2.56E-09	2.19E-07	metabolic process
GO:0008150	1191	5.20E-09	3.96E-07	biological_process
GO:0009987	657	6.44E-09	4.64E-07	cellular process
GO:0006412	67	7.89E-09	5.40E-07	translation
GO:0044238	506	4.07E-07	1.80E-05	primary metabolic process
GO:0044710	266	6.14E-07	2.55E-05	single-organism metabolic process
GO:0006091	36	1.36E-06	5.04E-05	generation of precursor metabolites and energy
GO:0044260	282	9.63E-06	3.29E-04	cellular macromolecule metabolic process
GO:0044281	198	1.58E-05	5.27E-04	small molecule metabolic process
GO:0044265	22	2.27E-05	7.06E-04	cellular macromolecule catabolic process
GO:0019941	19	2.69E-05	7.53E-04	modification-dependent protein catabolic process
GO:0030163	19	2.69E-05	7.53E-04	protein catabolic process
GO:0043632	19	2.69E-05	7.53E-04	modification-dependent macromolecule catabolic process
GO:0044257	19	2.69E-05	7.53E-04	cellular protein catabolic process
GO:0051603	19	2.69E-05	7.53E-04	proteolysis involved in cellular protein catabolic process
GO:0009056	74	4.66E-05	1.27E-03	catabolic process
GO:1901575	70	6.49E-05	1.74E-03	organic substance catabolic process
GO:0071840	65	7.05E-05	1.85E-03	cellular component organization or biogenesis
GO:0033036	46	8.02E-05	2.07E-03	macromolecule localization
GO:0044763	298	8.81E-05	2.23E-03	single-organism cellular process
GO:0022613	27	1.22E-04	2.87E-03	ribonucleoprotein complex biogenesis
GO:0044085	31	1.33E-04	3.08E-03	cellular component biogenesis
GO:0006508	24	1.49E-04	3.41E-03	proteolysis
GO:0043436	112	1.69E-04	3.79E-03	oxoacid metabolic process
GO:0006418	30	1.74E-04	3.84E-03	tRNA aminoacylation for protein translation
GO:0019752	110	2.06E-04	4.48E-03	carboxylic acid metabolic process
GO:0006082	112	2.45E-04	5.24E-03	organic acid metabolic process
GO:0009057	30	2.72E-04	5.71E-03	macromolecule catabolic process
GO:0043038	30	3.98E-04	7.89E-03	amino acid activation
GO:0043039	30	3.98E-04	7.89E-03	tRNA aminoacylation
GO:0044699	361	4.21E-04	8.23E-03	single-organism process
GO:0016485	24	4.33E-04	8.23E-03	protein processing
GO:0051604	24	4.33E-04	8.23E-03	protein maturation
GO:0008104	40	4.75E-04	8.68E-03	protein localization
GO:0015031	40	4.75E-04	8.68E-03	protein transport
GO:0045184	40	4.75E-04	8.68E-03	establishment of protein localization
GO:0007010	11	7.68E-04	1.37E-02	cytoskeleton organization
GO:0006520	87	8.10E-04	1.42E-02	cellular amino acid metabolic process
GO:0044249	162	9.23E-04	1.60E-02	cellular biosynthetic process
GO:0044248	49	1.02E-03	1.75E-02	cellular catabolic process
GO:1901576	162	1.12E-03	1.86E-02	organic substance biosynthetic process
GO:0009058	167	1.14E-03	1.86E-02	biosynthetic process
GO:0034660	39	1.35E-03	2.17E-02	ncRNA metabolic process
GO:0043933	19	1.41E-03	2.24E-02	macromolecular complex subunit organization
GO:0006006	18	2.22E-03	3.38E-02	glucose metabolic process

GO:0051128	9	2.37E-03	3.52E-02	regulation of cellular component organization
GO:0044723	34	2.50E-03	3.69E-02	single-organism carbohydrate metabolic process
GO:0019318	23	2.59E-03	3.74E-02	hexose metabolic process
GO:0055114	20	2.95E-03	4.16E-02	oxidation-reduction process
GO:1901564	124	3.03E-03	4.24E-02	organonitrogen compound metabolic process
GO:0005996	24	3.54E-03	4.85E-02	monosaccharide metabolic process
GO:0071822	17	3.66E-03	4.91E-02	protein complex subunit organization
GO:0015980	17	4.38E-03	5.83E-02	energy derivation by oxidation of organic compounds
GO:0006399	35	5.37E-03	6.99E-02	tRNA metabolic process
GO:0030029	8	5.47E-03	6.99E-02	actin filament-based process
GO:0030036	8	5.47E-03	6.99E-02	actin cytoskeleton organization
GO:1901137	19	5.72E-03	7.24E-02	carbohydrate derivative biosynthetic process
GO:0042254	18	7.08E-03	8.65E-02	ribosome biogenesis
GO:1902589	12	7.84E-03	9.50E-02	single-organism organelle organization
GO:0045333	14	8.15E-03	9.78E-02	cellular respiration
GO:0033043	7	9.72E-03	1.13E-01	regulation of organelle organization

Table M. GO enrichment analysis of *B. bassiana* genes at 48 hpt_I.

GO ID	Gene numbers	P-value	FDR	GO term
**Cellular component**				
GO:0005622	660	2.22E-31	3.04E-28	intracellular
GO:0044424	640	1.51E-29	1.03E-26	intracellular part
GO:0005623	762	1.90E-26	6.50E-24	cell
GO:0044464	762	1.90E-26	6.50E-24	cell part
GO:0032991	270	5.89E-23	1.61E-20	macromolecular complex
GO:0005575	984	1.06E-22	2.42E-20	cellular_component
GO:0030529	125	3.45E-16	5.91E-14	ribonucleoprotein complex
GO:0043226	381	1.41E-13	2.14E-11	organelle
GO:0043229	378	2.83E-13	3.87E-11	intracellular organelle
GO:0043227	322	2.56E-11	2.34E-09	membrane-bounded organelle
GO:0043231	302	1.00E-10	8.09E-09	intracellular membrane-bounded organelle
GO:0043234	124	4.87E-10	3.33E-08	protein complex
GO:0044444	103	2.58E-06	1.07E-04	cytoplasmic part
GO:0044422	146	3.27E-06	1.18E-04	organelle part
GO:0005737	103	5.83E-06	1.95E-04	cytoplasm
GO:0044446	129	3.88E-05	1.13E-03	intracellular organelle part
GO:0031090	88	4.98E-05	1.42E-03	organelle membrane
GO:0031967	63	5.74E-04	1.31E-02	organelle envelope
GO:0031975	63	5.74E-04	1.31E-02	envelope
GO:0019866	46	1.40E-03	2.77E-02	organelle inner membrane
GO:0005739	45	1.86E-03	3.44E-02	mitochondrion
GO:0012505	32	2.44E-03	4.27E-02	endomembrane system
GO:0043228	64	7.48E-03	1.08E-01	non-membrane-bounded organelle
GO:0043232	64	7.48E-03	1.08E-01	intracellular non-membrane-bounded organelle
GO:0044429	36	8.44E-03	1.19E-01	mitochondrial part
**Molecular Function**				
GO:0005198	78	9.21E-12	9.70E-10	structural molecule activity
GO:0097159	514	1.16E-09	6.90E-08	organic cyclic compound binding
GO:1901363	513	1.34E-09	7.63E-08	heterocyclic compound binding
GO:0036094	388	2.69E-08	1.42E-06	small molecule binding
GO:0000166	374	1.27E-07	5.45E-06	nucleotide binding
GO:1901265	374	1.27E-07	5.45E-06	nucleoside phosphate binding
GO:0097367	257	2.82E-06	1.08E-04	carbohydrate derivative binding
GO:0030554	207	5.69E-06	1.95E-04	adenyl nucleotide binding
GO:0017076	254	7.39E-06	2.41E-04	purine nucleotide binding
GO:0032559	206	1.03E-05	3.15E-04	adenyl ribonucleotide binding
GO:0032553	253	1.04E-05	3.15E-04	ribonucleotide binding
GO:0032555	253	1.04E-05	3.15E-04	purine ribonucleotide binding
GO:0003723	43	2.43E-04	6.29E-03	RNA binding
GO:0016757	32	5.26E-04	1.24E-02	transferase activity, transferring glycosyl groups
GO:0016616	23	6.65E-04	1.49E-02	oxidoreductase activity
GO:0003676	169	1.02E-03	2.14E-02	nucleic acid binding
GO:0008135	34	1.10E-03	2.28E-02	translation factor activity, nucleic acid binding
GO:0016879	31	1.14E-03	2.33E-02	ligase activity, forming carbon-nitrogen bonds
GO:0016614	31	2.39E-03	4.25E-02	oxidoreductase activity
GO:0016651	13	5.85E-03	8.90E-02	oxidoreductase activity, acting on NAD(P)H
GO:0008168	21	6.09E-03	9.06E-02	methyltransferase activity
**Biological process**				

GO:0071704	799	2.40E-19	4.69E-17	organic substance metabolic process
GO:0044710	352	1.51E-12	1.88E-10	single-organism metabolic process
GO:0044237	695	5.98E-12	6.82E-10	cellular metabolic process
GO:0009987	847	1.36E-11	1.33E-09	cellular process
GO:0044763	400	1.00E-10	8.09E-09	single-organism cellular process
GO:0043170	512	1.40E-10	1.06E-08	macromolecule metabolic process
GO:0044699	488	3.90E-10	2.81E-08	single-organism process
GO:0019538	241	5.27E-10	3.37E-08	protein metabolic process
GO:0044238	658	5.41E-10	3.37E-08	primary metabolic process
GO:0010467	289	2.34E-09	1.28E-07	gene expression
GO:0008150	1530	3.98E-08	2.02E-06	biological_process
GO:0008152	1112	5.12E-08	2.49E-06	metabolic process
GO:0044281	255	5.28E-08	2.49E-06	small molecule metabolic process
GO:0044267	198	5.47E-08	2.50E-06	cellular protein metabolic process
GO:0006082	145	2.85E-06	1.08E-04	organic acid metabolic process
GO:0019752	142	2.90E-06	1.08E-04	carboxylic acid metabolic process
GO:1901564	166	2.91E-06	1.08E-04	organonitrogen compound metabolic process
GO:0043436	144	3.46E-06	1.22E-04	oxoacid metabolic process
GO:0006520	113	1.24E-05	3.70E-04	cellular amino acid metabolic process
GO:0006412	71	6.41E-05	1.79E-03	translation
GO:0008104	48	1.78E-04	4.69E-03	protein localization
GO:0015031	48	1.78E-04	4.69E-03	protein transport
GO:0045184	48	1.78E-04	4.69E-03	establishment of protein localization
GO:0033036	53	3.06E-04	7.76E-03	macromolecule localization
GO:0022613	30	4.14E-04	1.03E-02	ribonucleoprotein complex biogenesis
GO:0006793	115	4.50E-04	1.10E-02	phosphorus metabolic process
GO:0071840	75	5.25E-04	1.24E-02	cellular component organization or biogenesis
GO:0006091	37	6.78E-04	1.50E-02	generation of precursor metabolites and energy
GO:0006796	112	7.88E-04	1.71E-02	phosphate-containing compound metabolic process
GO:0044711	64	1.00E-03	2.14E-02	single-organism biosynthetic process
GO:1901566	57	1.28E-03	2.57E-02	organonitrogen compound biosynthetic process
GO:0006006	21	1.42E-03	2.77E-02	glucose metabolic process
GO:0005996	29	1.49E-03	2.88E-02	monosaccharide metabolic process
GO:0044085	34	1.51E-03	2.88E-02	cellular component biogenesis
GO:0055086	79	1.64E-03	3.08E-02	nucleobase-containing small molecule metabolic process
GO:0044260	342	1.93E-03	3.53E-02	cellular macromolecule metabolic process
GO:0044723	41	2.25E-03	4.05E-02	single-organism carbohydrate metabolic process
GO:0006807	360	2.46E-03	4.27E-02	nitrogen compound metabolic process
GO:0019318	27	2.81E-03	4.81E-02	hexose metabolic process
GO:0044712	52	3.12E-03	5.27E-02	single-organism catabolic process
GO:0034660	46	3.24E-03	5.40E-02	ncRNA metabolic process
GO:1901605	46	3.28E-03	5.40E-02	alpha-amino acid metabolic process
GO:0006007	18	4.37E-03	6.87E-02	glucose catabolic process
GO:0019320	18	4.37E-03	6.87E-02	hexose catabolic process
GO:0044724	18	4.37E-03	6.87E-02	single-organism carbohydrate catabolic process
GO:0046365	18	4.37E-03	6.87E-02	monosaccharide catabolic process
GO:0019637	81	5.12E-03	7.96E-02	organophosphate metabolic process
GO:0006753	73	5.19E-03	7.99E-02	nucleoside phosphate metabolic process
GO:1901137	22	5.94E-03	8.93E-02	carbohydrate derivative biosynthetic process
GO:0042254	21	7.09E-03	1.04E-01	ribosome biogenesis
GO:0006399	42	8.31E-03	1.19E-01	tRNA metabolic process
GO:1901576	199	8.66E-03	1.21E-01	organic substance biosynthetic process
GO:0065008	21	8.80E-03	1.22E-01	regulation of biological quality
GO:0009058	205	9.78E-03	1.34E-01	biosynthetic process

GO:0044249	198	9.90E-03	1.34E-01	cellular biosynthetic process

Table N. Putative *B. bassiana* secretion proteins continuous expressed during infection.

Gene ID	RPKM 24 hpt_I	36 hpt_I	48 hpt_I	Annotation
BBA_08898		0.3741	0.5024	Peptidase cysteine/serine, trypsin
BBA_10053	0.2493	0.0226	0.0228	NIMA-interacting protein TinC
BBA_07199	1.3754	0.4406	0.8137	peptidyl-prolyl cis-trans isomerase B
BBA_02046		0.0734	0.1849	nucleotide exchange factor SIL1
BBA_00520	0.3607	0.1801	0.1979	heterokaryon incompatibility protein Het-C
BBA_07851		0.1627	0.4778	hypothetical protein BBA_07851
BBA_02025	0.2452	0.0159	0.3042	glycoside hydrolase family 31
BBA_03905	0.6326	0.7810	1.4114	hsp70-like protein
BBA_05172		0.0865	0.1161	glycosyl hydrolase family 47
BBA_08491	2.0279	0.0969	0.1301	GEgh16 protein
BBA_01337	0.2395		0.2190	hypothetical protein BBA_01337
BBA_06454		0.0404	0.2442	glycosyltransferase family 17
BBA_04983	0.0431	0.0392		Heterokaryon incompatibility Het-C
BBA_03039		0.0769	0.4131	flavin containing amine oxidoreductase
BBA_09468	0.0640		0.0488	WSC domain-containing protein
BBA_06777	0.1111	0.0504	0.2031	nuclease S1
BBA_04223		0.0529	0.0533	gamma-glutamyltranspeptidase
BBA_08434		0.1046	0.1054	lipase class 2
BBA_06623	0.1344	0.0610	0.2150	disulfide isomerase
BBA_06290	0.0669	0.0304		amidohydrolase-like protein
BBA_10292	1.3236	0.3433	1.0373	cytochrome c oxidase assembly factor
BBA_00303	0.6472	0.0452	0.2276	endoglucanase, putative
BBA_00564		0.0931	0.0938	hypothetical protein BBA_00564
BBA_08504		0.7236	0.2429	hypothetical protein BBA_08504
BBA_08440		0.1872	0.4525	Molecular chaperone, heat shock protein, Hsp40
BBA_08183	0.0570	0.0259	0.2086	laccase 2
BBA_01026	0.0471	0.0856		hypothetical protein BBA_01026
BBA_04314	0.0223		0.0817	alpha-N-acetylglucosaminidase, putative
BBA_06203	0.5704		0.7823	hypothetical protein BBA_06203
BBA_03834	0.1530		0.0699	glycosyl hydrolase family 16
BBA_00968	0.5724		0.5233	hypothetical protein BBA_00968
BBA_01346	1.2539	0.5391	0.3620	hypothetical protein BBA_01346
BBA_02865		0.0459	0.0462	serine carboxypeptidase
BBA_02608	0.9755	0.5535	0.3345	biotrophy-associated secreted protein 2
BBA_07659	0.4418		1.0649	sulphydryl oxidase
BBA_07690	0.1580	0.2869	0.1445	hypothetical protein BBA_07690
BBA_05303	0.2343	0.1063		subtilase-like protein
BBA_00039	1.3571	1.2319	0.4963	hypothetical protein BBA_00039
BBA_07333	1.8698	1.4548	2.6865	hypothetical protein BBA_07333
BBA_03440	0.4808	0.4364		hypothetical protein BBA_03440
BBA_06599	8.2690	5.9259	8.7542	hydrophobin-like protein
BBA_05017	0.6708	0.1853	0.0800	amidase-like protein
BBA_06201		0.5875	0.2959	hypothetical protein BBA_06201
BBA_01227		0.1222	0.3693	hypothetical protein BBA_01227
BBA_06224	1.3764	0.1209	1.0555	ice nucleation protein
BBA_04972	0.1355	0.0492		eukaryotic aspartyl protease
BBA_07277	0.0562	0.0510		hypothetical protein BBA_07277
BBA_00736	0.4808	1.3093	0.7327	hypothetical protein BBA_00736
BBA_02068	0.2770	0.0419	0.0633	calcineurin-like phosphoesterase
BBA_05746		0.0583	0.0587	FAD binding domain protein

BBA_04066		0.0248	0.0249	PLC-like phosphodiesterase
BBA_01335	0.0713	0.1942		GPI anchored glycoprotein, putative
BBA_06564	1.9083	0.7874	0.6345	hypothetical protein BBA_06564
BBA_02374	0.1062		0.0485	metalloprotease-like protein
BBA_10203	0.2134	0.1937	0.4181	brefeldin A resistance protein
BBA_02148	0.9297	0.7596	0.8501	hypothetical protein BBA_02148
BBA_08623	0.3205		0.4396	hypothetical protein BBA_08623
BBA_06297	0.2057	0.1120	0.0376	chitinase-like protein
BBA_00080	0.3825	0.1736	0.6120	hypothetical protein BBA_00080
BBA_10055	0.3418	0.0955	0.0481	putative hexosaminidase
BBA_07560	1.0825	0.1787	0.1800	hypothetical protein BBA_07560
BBA_06589	0.1623	0.2947		5'/3'-nucleotidase SurE
BBA_08723	0.7317	0.1107	0.1115	Epl1 protein
BBA_08695		0.0335	1.6852	serin endopeptidase
BBA_09070	0.3698	0.5036	1.3526	hypothetical protein BBA_09070
BBA_04526	0.0792	0.0359		cellulase-like protein
BBA_08224	2.8347	2.6140	1.0285	hypothetical protein BBA_08224
BBA_02727		0.0382	0.0769	Chymotrypsin-like protein
BBA_09296		0.1328	0.4014	hypothetical protein BBA_09296
BBA_01690		0.2777	0.2797	phosphatidylinositol/phosphatidylglycerol transfer protein
BBA_02781	5.4593	2.2990	1.3379	WSC domain-containing protein
BBA_09728	0.6350	1.8014	3.2659	hypothetical protein BBA_09728
BBA_01324		0.7767	3.3902	hypothetical protein BBA_01324
BBA_08022	0.4315	0.3314	1.0622	protein disulfide isomerase
BBA_07494	0.0166		0.1212	SPX domain-containing protein
BBA_02121	8.1336	3.1824	1.6668	hypothetical protein BBA_02121
BBA_02631	0.3914	1.4210	0.2684	hypothetical protein BBA_02631
BBA_07874		0.0460	0.0464	endoplasmic Reticulum Oxidoreductin 1
BBA_07427	0.6556	1.9838		hypothetical protein BBA_07427
BBA_10272		0.0519	0.0732	UDP-glucose:glycoprotein glucosyltransferase
BBA_04722	0.0557	0.0506		hypothetical protein BBA_04722
BBA_07048	0.3506	0.5304	0.4274	hypothetical protein BBA_07048
BBA_00745	0.3629	0.2882	0.5391	disulfide-isomerase erp38
BBA_05929	0.1265	0.0766	0.1542	WSC domain-containing protein
BBA_09033	0.2550	0.8101	2.2147	hypothetical protein BBA_09033
BBA_02095	0.4674		0.4274	hypothetical protein BBA_02095
BBA_04446	0.2524		0.4616	PCI domain-containing protein
BBA_02230		0.0422	0.0213	chitinase-like protein
BBA_08789	0.8121	0.3276	0.3300	phosphorylcholine phosphatase
BBA_06292	0.0774		0.1415	subtilase-like protein
BBA_02999	1.2020	0.7794	0.4710	hydrophobin-like protein
BBA_07148		0.1736	0.1748	hypothetical protein BBA_07148
BBA_01712		0.0491	0.8905	hypothetical protein BBA_01712
BBA_07293	2.2724	1.0035	0.4492	hypothetical protein BBA_07293
BBA_00246	0.2019	0.8554	0.1846	cutinase-like protein
BBA_02446	0.1654	0.0375	0.3024	ice nucleation protein
BBA_09760	0.1689	0.1342	0.3282	Catalase-peroxidase
BBA_05552		0.0832	0.0838	glycosyltransferase
BBA_02206	0.1096		0.0501	carboxypeptidase S1
BBA_06720	2.0005	1.4955		hypothetical protein BBA_06720
BBA_09155	3.1346	0.7488	0.3017	hypothetical protein BBA_09155
BBA_07501		0.3433	0.6915	hypothetical protein BBA_07501
BBA_03268	0.2003		0.3663	hypothetical protein BBA_03268
BBA_08776	14.8951	4.6446	1.5594	hypothetical protein BBA_08776

BBA_09878	0.1809	0.3285	0.3309	hypothetical protein BBA_09878
BBA_10020		0.0589	0.1482	DnaJ domain-containing protein
BBA_03898	0.7212	0.5456	0.7693	small secreted protein
BBA_02140	0.1150	0.1392		hypothetical protein BBA_02140
BBA_05894	0.0663		0.0606	eukaryotic aspartyl protease
BBA_06527	0.3473	0.1146		feruloyl esterase B precursor
BBA_10328	0.6339		0.1054	hypothetical protein BBA_10328
BBA_04700	0.6350	0.1441	0.8709	hypothetical protein BBA_04700
BBA_08078	0.1923	0.2619	0.0879	hypothetical protein BBA_08078
BBA_04640	1.2886	0.9977	1.0743	glucanosyltransferase-like protein
BBA_08080	0.2047		0.0374	hypothetical protein BBA_08080
BBA_06650	0.0694	0.0945		putative aspartic endopeptidase
BBA_07745	0.0982	0.1783	1.0776	Glycoside hydrolase, catalytic core
BBA_00386	1.2945	0.6409	0.9684	FKBP-type peptidyl-prolyl cis-trans isomerase
BBA_05577	0.2329	0.3171	0.3194	SCP-like extracellular protein
BBA_09501		0.0471	0.0950	subtilisin-like protease PR1F
BBA_05436	1.3794	1.3356	0.2522	hypothetical protein BBA_05436
BBA_00698		0.0887	0.2680	restless-like transposase
BBA_08214	0.0839	0.0762		cell wall glucanosyltransferase Mwg1
BBA_08602	0.3983		0.3642	LysM domain-containing protein
BBA_05281	0.8742	0.7935	0.1998	hypothetical protein BBA_05281
BBA_08573	2.2653	0.5141	0.2219	extracellular serine-rich protein
BBA_07600	0.5048		0.1539	hypothetical protein BBA_07600
BBA_10113	0.1784	0.2699		surface antigen
BBA_02352	19.4091	12.2767	23.9760	hypothetical protein BBA_02352
BBA_05808	0.1261	0.2288	0.5186	cell wall protein
BBA_05264	2.0653	1.2498	0.2098	chitinase-like protein
BBA_01553	0.1972	0.1193		hypothetical protein BBA_01553
BBA_00943	1.2995	0.4718	0.6535	hypothetical protein BBA_00943
BBA_07943	0.0813	0.1476		glycerophosphoryl diester phosphodiesterase
BBA_07637		0.3917	0.1973	hypothetical protein BBA_07637
BBA_03015	0.1237	0.7862	0.7919	class I hydrophobin
BBA_05879	0.0545	0.0494		aorsin-like protein
BBA_09685	0.4308	0.6778	0.3413	metalloprotease-like protein
BBA_08600		0.0310	0.2497	1,3-beta-glucanosyltransferase gel2
BBA_02379		0.3990	1.0334	adhesin protein Mad2
BBA_02602	2.6097	0.4084	2.0570	cell wall protein
BBA_03207	0.0619	0.0281		amidase-like protein
BBA_02045	0.3357	0.7238	0.5755	exo-beta-1,3-glucanase, putative
BBA_07227	0.0668		0.0611	malate dehydrogenase
BBA_08200	0.0768	0.0698	0.7026	p24 protein, involved in membrane trafficking
BBA_05689	2.1472	0.3173	0.9588	extracellular dioxygenase
BBA_08480	0.1463	0.2657	1.4048	hypothetical protein BBA_08480
BBA_06750	1.9633	2.2913	1.5386	hypothetical protein BBA_06750
BBA_03016		0.0736	0.1112	peptidase family protein
BBA_05283	0.0870		0.0795	peptidase family M28
BBA_04710	0.2475	1.0109	0.7919	Mmc protein
BBA_09621	0.1088	0.0593	0.0796	glycosyl hydrolase, putative
BBA_09427	0.1594	0.2025	0.1166	serine carboxypeptidase
BBA_07648	1.1144	0.8093	0.1019	hypothetical protein BBA_07648
BBA_04098	0.1451	0.3951	0.1326	hypothetical protein BBA_04098
BBA_06139	0.0389		0.2132	GPI anchored protein
BBA_09500		0.0915	0.2073	cuticle-degrading serine protease
BBA_02548	0.0495	0.0899	0.0905	twin-arginine translocation pathway signal

BBA_01466	0.0648	0.1177	0.2372	beta-glucosidase
BBA_09280	3.2475	1.7420	0.4049	hypothetical protein BBA_09280
BBA_04521		0.1107	0.1115	hypothetical protein BBA_04521
BBA_08621		0.1538	0.1033	Axe2-like protein
BBA_05603	0.0230		0.0841	Catalase-like domain, heme-dependent
BBA_00766	0.1310	0.2378		ribonuclease Trv, RNase Trv
BBA_01529	0.1167	0.1059	0.2667	prenylcysteine oxidase
BBA_05615	1.0090	0.5909	0.2381	metalloprotease-like protein
BBA_08498	0.1325		0.0606	endonuclease/exonuclease/phosphatase family
BBA_03412	1.3166	0.5976	0.3010	putative cell wall glycoprotein
BBA_01910	0.5636		0.5153	hypothetical protein BBA_01910
BBA_02309	0.1717		0.1570	serine proteinase inhibitor IA-2
BBA_08416		0.0604	0.0304	peptidase family M28
BBA_02852	0.1242	0.0282	0.0568	penicillin-binding protein
BBA_01658	0.2293	0.2497	0.2515	CAS1-like protein
BBA_09242	1.0684	0.3233	1.3025	ecm33-like protein
BBA_09422	0.1691	0.1535		hypothetical protein BBA_09422
BBA_07621	0.4476	0.0813	0.0818	hypothetical protein BBA_07621
BBA_08791	0.0793	0.0480	0.0242	alkaline serine protease AorO
BBA_01654	0.0709	0.1286	0.0324	oxalate decarboxylase family bicupin
BBA_10058	0.0524	0.0476	0.5752	hypothetical protein BBA_10058
BBA_08030	0.1923	0.1746	0.5275	hypothetical protein BBA_08030
BBA_00702	0.1222	0.1663	0.8936	lipase, putative
BBA_00850		0.0266	0.1072	putative SERINE-TYPE CARBOXYPEPTIDASE F PRECURSOR
BBA_07009	0.4518	0.4101	0.7228	alkaline foam protein B precursor
BBA_07997	0.9466	0.2387		hypothetical protein BBA_07997
BBA_02239	0.6390	0.0967	0.0779	eukaryotic aspartyl protease
BBA_03990	0.1683	0.4583	0.9539	beta-1,3-glucanosyltransferase
BBA_02529	2.6571	1.7866	2.1595	DNase1 protein
BBA_03668	0.1169	0.2122	0.6945	hypothetical protein BBA_03668
BBA_09144	0.3060	0.2777	0.5595	GPI anchored protein, putative
BBA_07623	0.0500	0.1362	0.2743	trehalase-like protein
BBA_02459		0.3070	0.1546	hypothetical protein BBA_02459
BBA_06776	0.0687	0.1247		hypothetical protein BBA_06776
BBA_07249	0.0310	0.1409		ECM14-like protein
BBA_04792	1.7126	0.7435		hypothetical protein BBA_04792
BBA_07358		0.2869	0.2889	hypothetical protein BBA_07358
BBA_05770		0.0505	0.1400	serine/threonine kinase IREI
BBA_09173	9.8164	3.1824	1.2822	hypothetical protein BBA_09173
BBA_08970	1.8889	1.8705	2.6690	hypothetical protein BBA_08970
BBA_01020	0.3970	0.0832	0.2234	glucosidase II beta subunit-like protein
BBA_01743	1.3932	0.7987	1.0392	glucan 1,3-beta-glucosidase
BBA_08863	2.2263	1.7237	0.1796	metalloprotease 1
BBA_02001	0.3725	0.2766	0.4334	WSC domain-containing protein
BBA_09753	0.6233	0.3772		hypothetical protein BBA_09753
BBA_10330	0.0390		0.3562	septation protein SUN4
BBA_08433	0.1291	1.2502	0.3542	quercetin 2,3-dioxygenase
BBA_07674		0.0477	0.0480	Peptidase S33, tripeptidyl-peptidase
BBA_10249		0.0391	0.1379	kynurenine 3-monooxygenase
BBA_02780		0.3250	0.4092	hypothetical protein BBA_02780
BBA_01863	0.9349	0.0943	0.8548	hypothetical protein BBA_01863
BBA_02361	0.5296	0.5875	0.8608	FMN-binding split barrel-related protein
BBA_07594	0.4888	0.1268	1.0853	hypothetical protein BBA_07594
BBA_09671		0.1490	0.1501	hypothetical protein BBA_09671

BBA_08402		0.0298	0.0902	monooxygenase-like protein
BBA_06749	0.7496	0.4083	0.1371	chitinase 18-11
BBA_04424		0.0455	0.0458	hypothetical protein BBA_04424
BBA_04903	0.3211	0.0583		rasp f 7 allergen
BBA_01961	0.0712	0.0646		hypothetical protein BBA_01961
BBA_04753	0.0287	0.2082	0.8912	Concanavalin A-like lectin/glucanase
BBA_06706	0.0835		0.0764	Chitin-binding, domain 3
BBA_02312	0.1590	0.2165	0.0969	eukaryotic aspartyl protease
BBA_08015	0.1307	0.0949		protein phosphatase type 1 complex subunit Hex2/Reg1
BBA_02532	0.1580	0.0717		S-layer protein
BBA_02290	0.1137		0.0693	eukaryotic aspartyl protease
BBA_07868	0.3580		0.2455	hypothetical protein BBA_07868
BBA_02253	0.1215	0.1103		cation efflux family protein
BBA_05906	0.8931	0.1280		serine-threonine rich protein
BBA_08497	0.0581	0.0528	0.0531	Glycoside hydrolase, family 20
BBA_00325	1.0409		0.6345	ribosomal protein L36
BBA_07620		0.1057	0.1065	polysaccharide deacetylase
BBA_07256		0.0339	0.0682	secretory pathway protein Sec39
BBA_03717	0.4286	0.0819	0.1031	beta-1,3-glucanase precursor
BBA_01022	0.0533	0.0242		WSC domain-containing protein
BBA_00307	0.7928	0.0654	0.4283	Protein of unknown function DUF2401, secretory
BBA_03167	1.5776	0.3472	1.8796	glycosyl hydrolase 53 domain-containing protein
BBA_08492	0.5832	0.0756		extracellular serine-rich protein
BBA_05976	0.2732	0.0248		Peptidase S33, tripeptidyl-peptidase
BBA_05194		0.0680	0.0228	molybdenum cofactor synthesis domain-containing protein
BBA_07920		0.0298	0.0902	Zinc finger CCCH type domain containing protein
BBA_08283	2.0948	1.0775	1.4684	hypothetical protein BBA_08283
BBA_06304		0.0800	0.0806	lipocalin-like domain-containing protein
BBA_04181	0.0508		0.0930	S1/P1 nuclease
BBA_03246	0.9935		0.2271	cell wall protein
BBA_01048		0.0844	0.2550	oxidoreductase domain-containing protein
BBA_07645		0.1501	0.3402	exopolyphosphatase-like protein
BBA_02925		0.1535	0.5412	peptidase family M28
